# An efficient and ecofriendly synthesis of highly functionalized pyridones *via* a one-pot three-component reaction†

**DOI:** 10.1039/c8ra05690k

**Published:** 2018-07-30

**Authors:** Hajar Hosseini, Mohammad Bayat

**Affiliations:** Department of Chemistry, Faculty of Science, Imam Khomeini International University Qazvin Iran bayat_mo@yahoo.com m.bayat@sci.ikiu.ac.ir +98(28)33780040

## Abstract

A simple and convenient protocol has been developed for the synthesis of *N*-amino-3-cyano-2-pyridone derivatives by a one-pot reaction of cyanoacetohydrazide, activated nitrile substrates (malononitrile, ethyl cyanoacetate, cyanoacetamide) and aromatic aldehydes in the presence of piperidine in water or a mixture of water and ethanol. The sequence of cascade reactions includes Knoevenagel condensation, Michael addition, intramolecular cyclization, imine-enamine tautomerization and oxidative aromatization. The main advantages of this procedure are availability of starting compounds, simple procedure, mild conditions, easy purification of products and the use of water or water/ethanol as green solvents.

## Abstract 2

Synthesis of *N*-amino-3-cyano-2-pyridone derivatives in green solvent with simple procedure and high purity *via* a one-pot operation.

## Graphical abstract

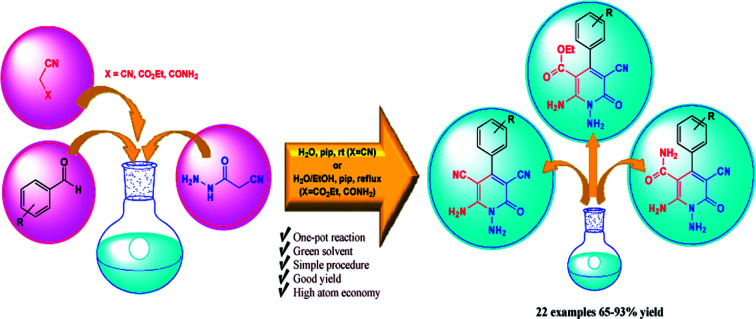

## Introduction

The chemistry and applications of pyridine structures have recently attracted a lot of attention due to their uses as synthetic intermediates and their biological importance as agrochemicals,^1–3^ pharmaceuticals,^4–8^ dye intermediates,^9,10^ insecticides, adhesives,^11^ antifungals, antibacterials,^12–14^ antidepressant agents,^15,16^ and antitumor agents.^17^ In fact the pyridine ring has been found in more than 7000 drugs which are already in existence.^18^

Furthermore, 2-pyridones are a unique category of pharmacophores which exhibit several biological activities such as antitumoral,^19^ antimalarial,^20^ analgesic,^21^ and anti-HIV.^22^

**Scheme 1:**
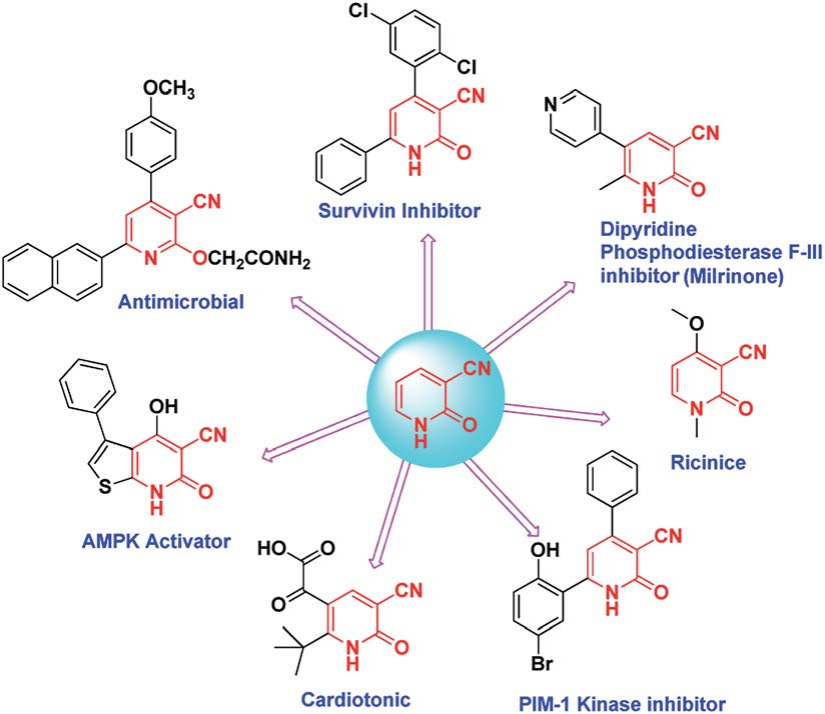
Biodynamic activities of different derivatives of 3-cyano-2-pyridone.

Also cyanopyridines are important intermediates for the synthesis of nicotinamide, nicotinic acid and isonicotinic acid. 3-Cyano-2-pyridones are one of the biodynamic cyanopyridine derivatives (Scheme 1).^23^ The significance of 3-cyano-2-pyridone frameworks in the past few decades is undeniable. For example they are the structural basis of the alkaloid ricinine, the first known alkaloid containing a cyano group. Milrinone is a 3-cyano-2-pyridone derivative that has been used for the treatment of congestive heart failure.^24,25^ Another derivative 3-cyano-2-pyridone has shown anticancer activity which might be due to the interference of the molecule with PDE-3,^26^ PIM-1 kinase,^27^ and survivin protein.^28^

**Scheme 2:**
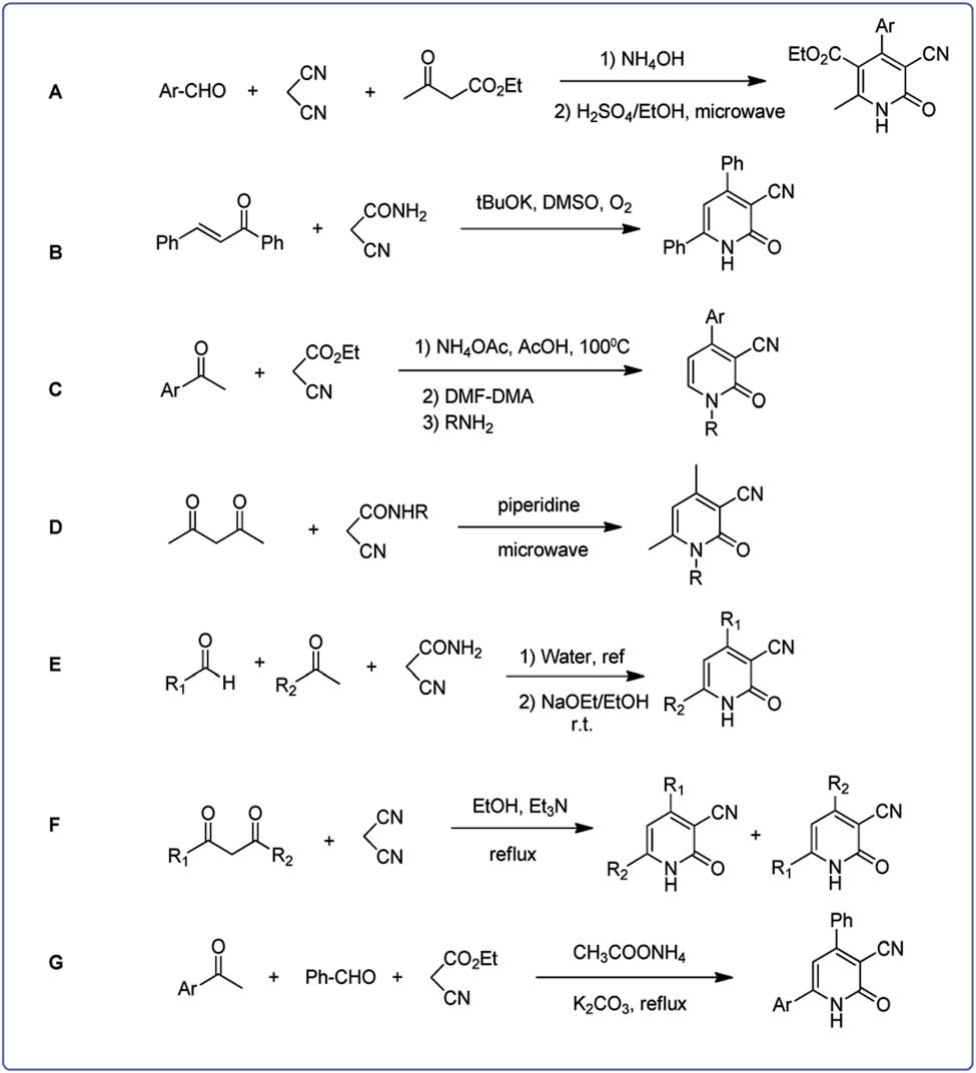
Summary of previous studies of 3-cyano-2-pyridones synthesis.

Due to the great importance of this skeleton, the development of efficient and environmentally benign methodologies for the synthesis of diverse functionalized 2-pyridones is still highly desired. There are some strategies for the synthesis of 3-cyano-2-pyridone derivatives which already have been reported, most of them proceed through the regioselective cyclocondensation of an acetonitrile derivative (cyanoacetate ester, cyanoacetamide or malononitrile) with a suitable carbonyl substrate in a [3 + 3] mode. In fact, Michael addition of acetonitrile derivatives to an appropriate carbonyl substrate (1,3-dicarbonyl or α,β-unsaturated compound) and subsequent hydrolytic cyclization followed by oxidative aromatization leads to the corresponding 3-cyano-2-pyridones. Here, we outline some of the most efficient methods (Scheme 2).

Reaction of malononitrile, ethyl cyanoacetate and aromatic aldehydes in a two-step reaction led to corresponding 3-cyano-2-pyridones (A).^29^ In another work the reaction of various enones with cyanoacetamide, led to 3-cyano-2-pyridones by operating in DMSO and in the presence of excess *t*-BuOK under an oxygen atmosphere (B).^30^ The Knoevenagel product of aromatic ketones and ethyl acetoacetate reacted with *N*,*N*-dimethylformamide-dimethylacetal (DMF-DMA) to produce enaminonitriles. By adding various types of primary nucleophilic amines to enaminonitrile, 3-cyano-2-pyridone derivatives were formed (C).^31^

Condensation of acetylacetone and corresponding *N*-substituted cyanoacetamide using microwave irradiation led to pyridones (D).^32^ In a two-step reaction, firstly acrylamides were obtained *via* the reaction of aromatic aldehydes and cyanoacetamide in water, then acrylamides were reacted with enolates to produce corresponding 2-pyridones (E).^33^

Also 3-cyano-2-pyridinone derivatives were prepared in the reaction of 1,3-dicarbonyl compounds with malononitrile followed by cycloaddition and isomerization (F).^34^ In another study a one-pot reaction of acetophenone derivatives, malononitrile or ethyl cyanoacetate, an aldehyde, and ammonium acetate in the presence of K_2_CO_3_ led to the corresponding structures (G).^35^

Many of the established procedures carried out under harsh reaction conditions. On the other hand, one of the goals in modern synthetic organic chemistry includes the use of safer, easier and more effective methods.

## Results and discussion

Here we report a one-pot three-component reaction between cyanoacetohydrazide, acetonitrile derivatives (malononitrile, ethyl cyanoacetate, cyanoacetamide) and aromatic aldehydes. This strategy led to the *N*-amino-3-cyano-2-pyridones in good yields *via* a multicomponent reaction. Multicomponent reactions (MCRs) are extremely convergent one-pot processes, in which three or more reagents are combined sequentially to create complex products, with almost all the atoms coming from the starting reagents.^36–39^

The reaction of cyanoacetohydrazide, malononitrile or ethyl cyanoacetate and aldehydes has been reported in 1984 and 1997 with different procedure and there are no complete spectral data about the structures of products.^40,41^ Furthermore, synthesis of pyridones using of both cyanoacetamide and cyanoacetohydrazide has not been reported so far.

We succeeded in synthesizing three categories of highly functionalized 2-pyridone structures containing 1,6-diamino-4-aryl-2-oxo-1,2-dihydropyridine-3,5-dicarbonitrile, ethyl 1,6-diamino-4-aryl-3-cyano-2-oxo-1,2-dihydropyridine-5-carboxylate and 1,6-diamino-4-aryl-3-cyano-2-oxo-1,2-dihydropyridine-5-carboxamide.

**Scheme 3:**
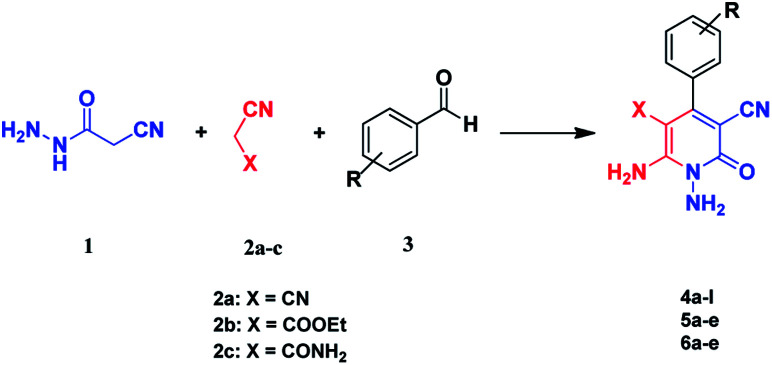
Synthetic scheme for generation of products 4, 5, 6.

Continuing our research on multi-component reactions using cyanoacetohydrazide, we will describe in this paper a very efficient and environmentally benign strategy for the synthesis of *N*-amino-3-cyano-2-pyridone derivatives by a one-pot three component reaction of cyanoacetohydrazide 1, acetonitrile derivatives 2 (malononitrile 2a, ethyl cyanoacetate 2b, cyanoacetamide 2c) and aromatic aldehydes 3 (Scheme 3).

For the better study of reactions and procedures and more detailed examination of structures, we divide the reactions into three class based on malononitrile 2a, ethyl cyanoacetate 2b and cyanoacetamide 2c.

As the first group of reactions, we used cyanoacetohydrazide 1, malononitrile 2a and aromatic aldehydes 3a–l in different conditions. The best results were obtained in water in the presence of piperidine as catalyst at room temperature. We could synthesize 12 pyridone derivatives with various aldehydes. In all of these reactions, 1,6-diamino-4-aryl-3,5-dicyano-2-pyridone 4a–l were obtained with high efficiency and relatively short time (Table 1). The structures of compounds 4a–l were derived from their IR, ^1^H NMR, ^13^C NMR spectroscopic and mass spectrometric data (see the ESI†).

**Table 1:** Synthesis of 1,6-diamino-4-aryl-2-oxo-1,2-dihydropyridine-3,5-dicarbonitrile 4a–l^a^

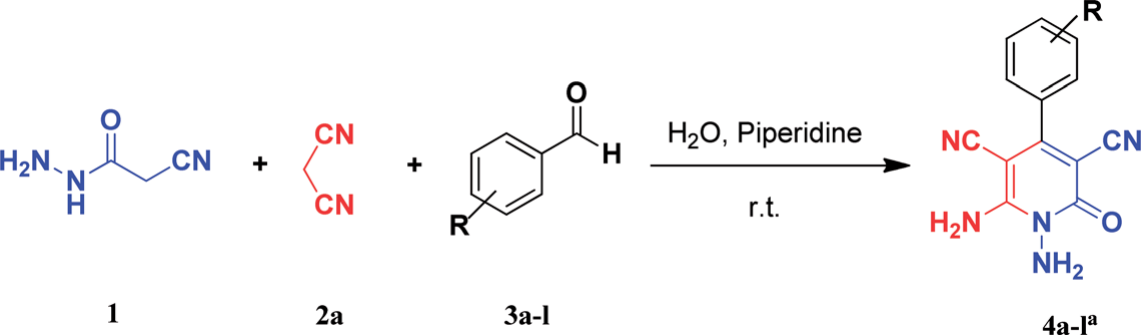					
Entry	Aromatic aldehyde	Product	Time (h)	Yield (%)	M.p. (°C)
1	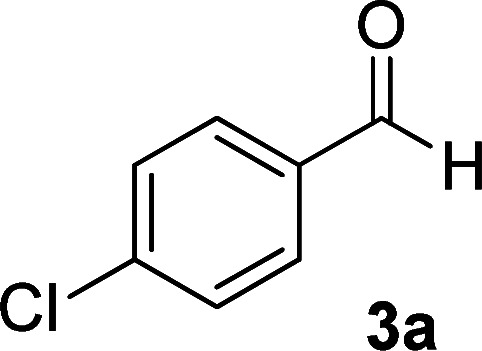	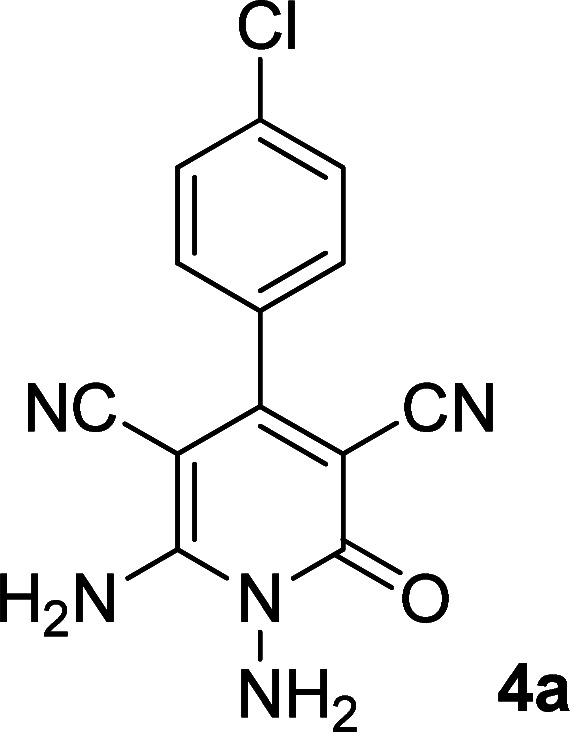	12	93	340 (dec.)^41^
2	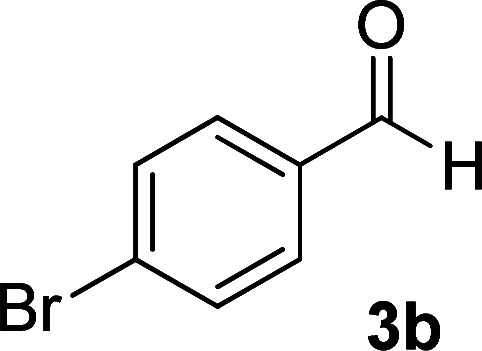	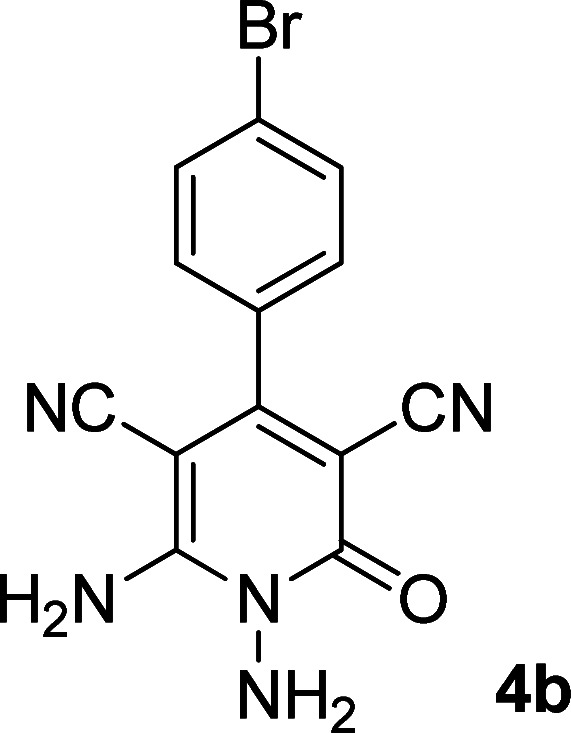	12	88	355 (dec.)
3	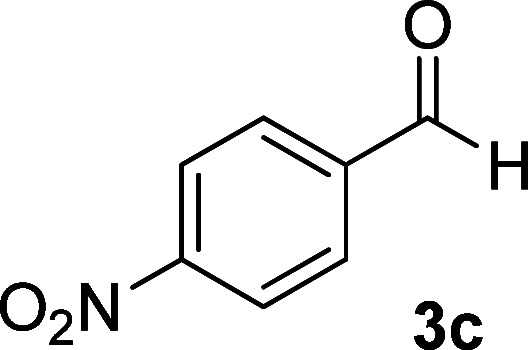	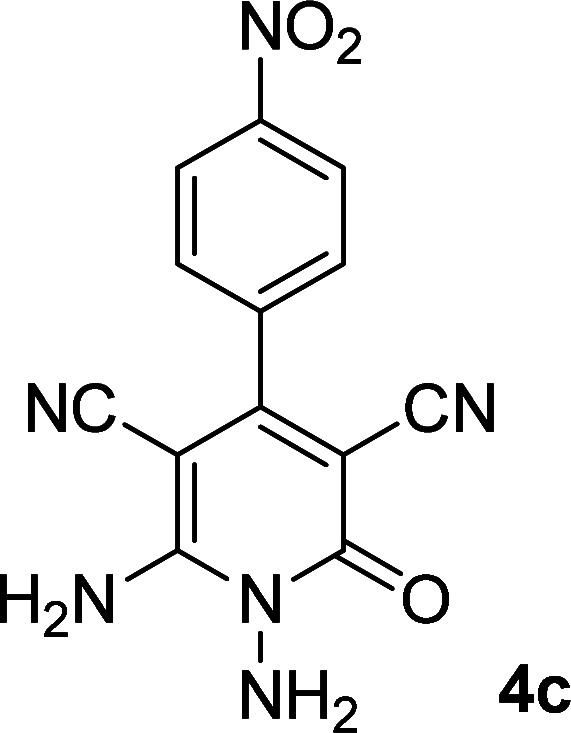	12	91	360 (dec.)^41^
4	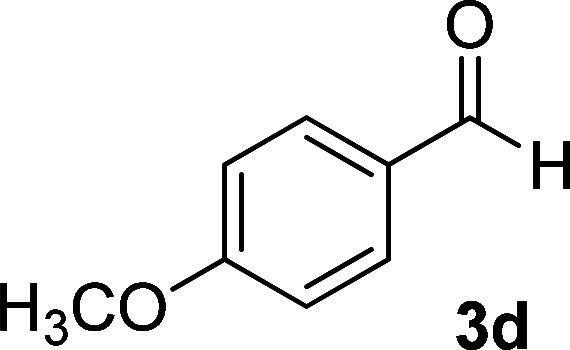	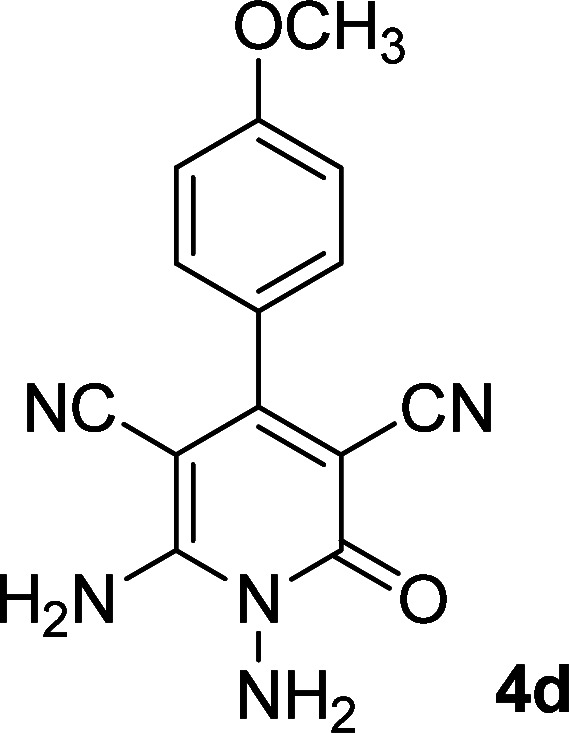	16	83	321–323 (ref. 41)
5	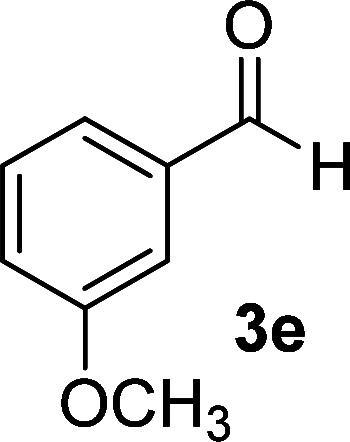	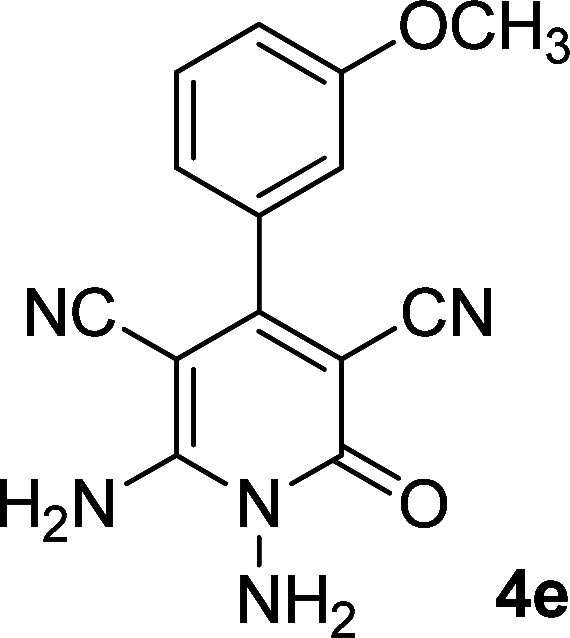	15	85	265–267
6	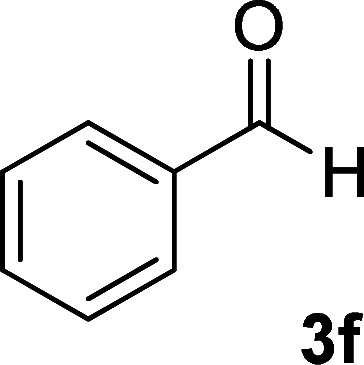	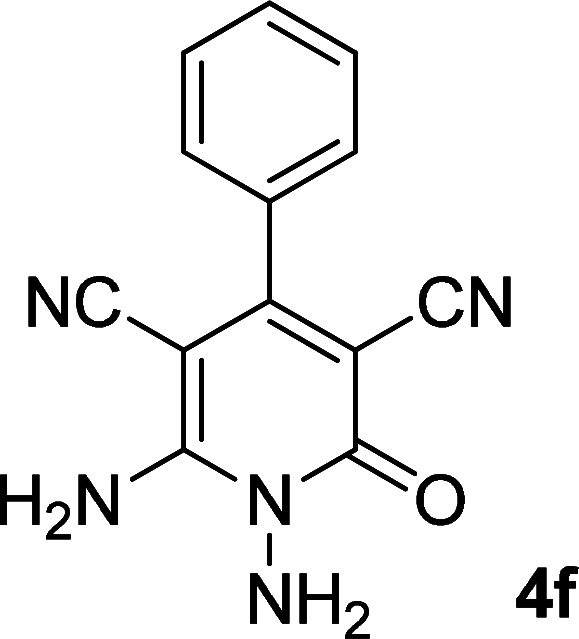	12	90	332–334 (ref. 41)
7	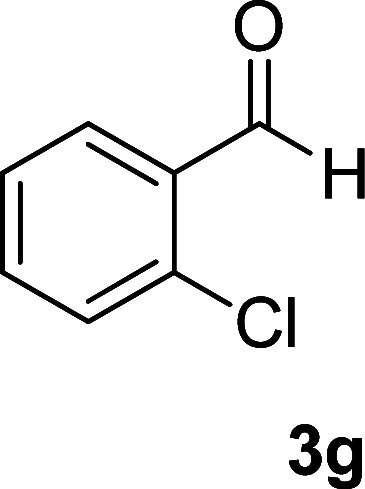	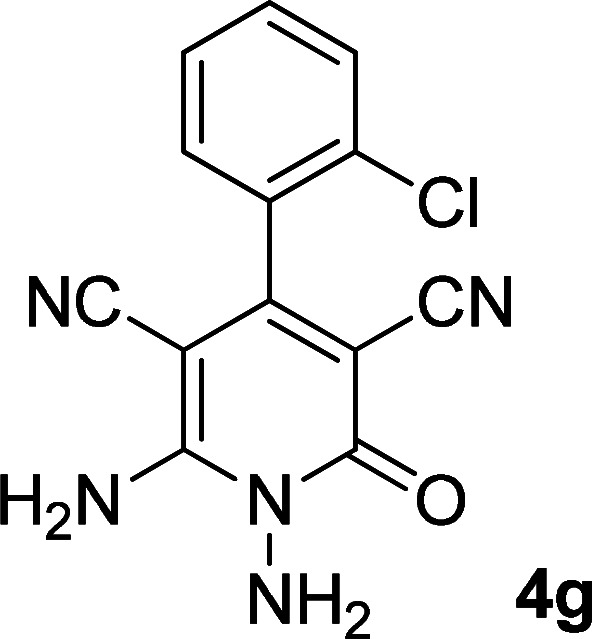	11	87	305–307 (ref. 41)
8	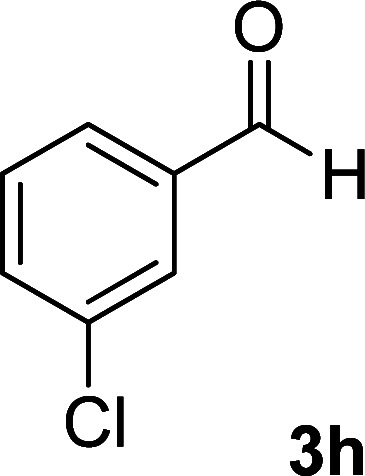	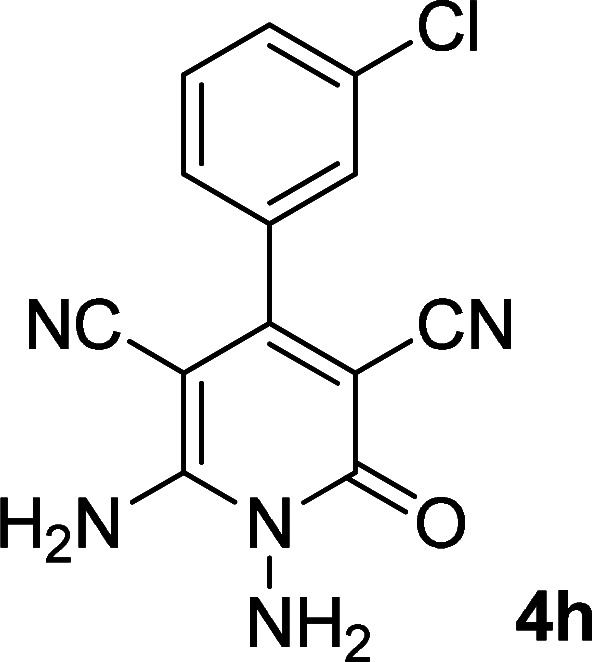	12	85	292–294
9	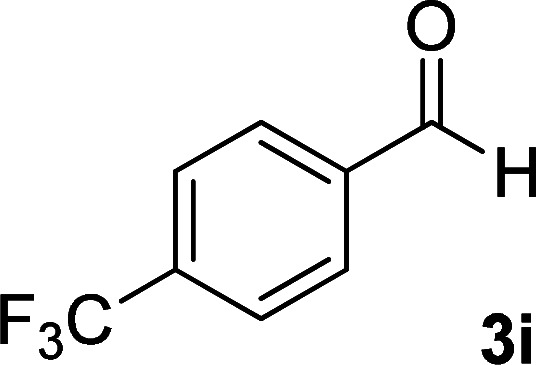	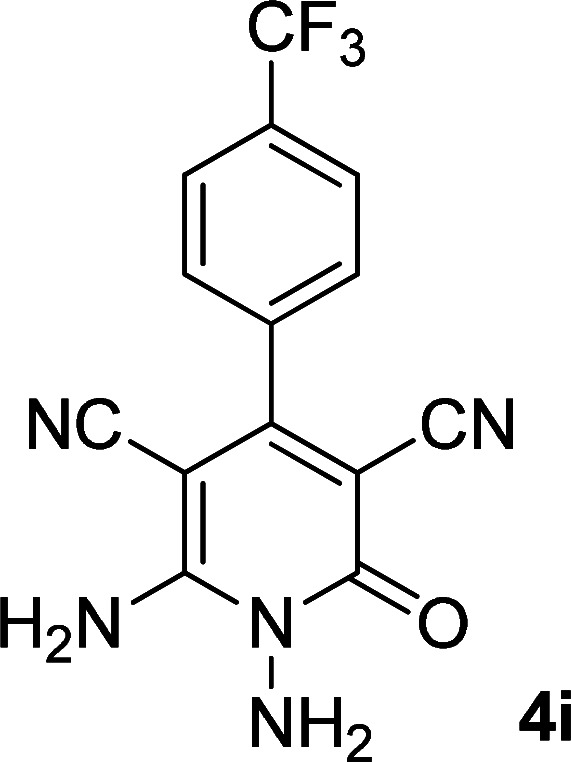	13	80	365 (dec.)
10	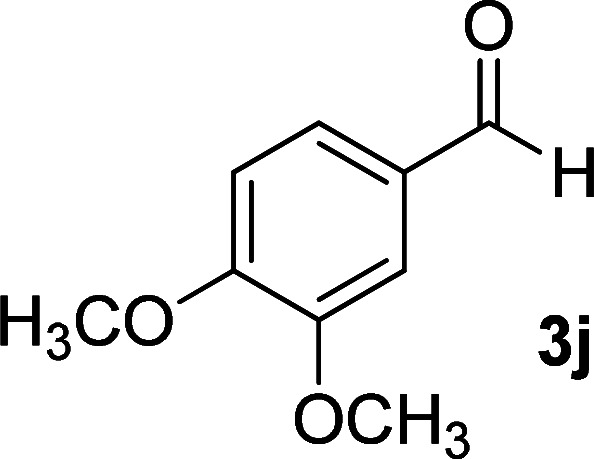	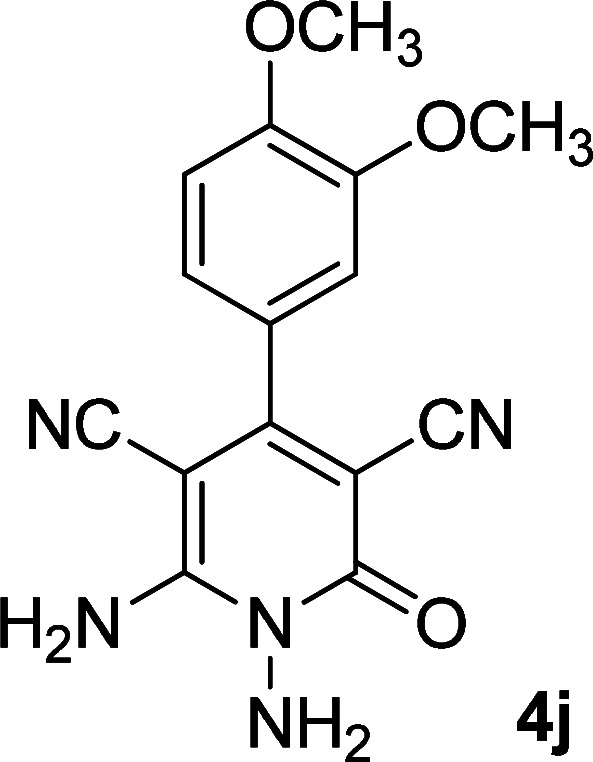	17	75	290–292 (ref. 41)
11	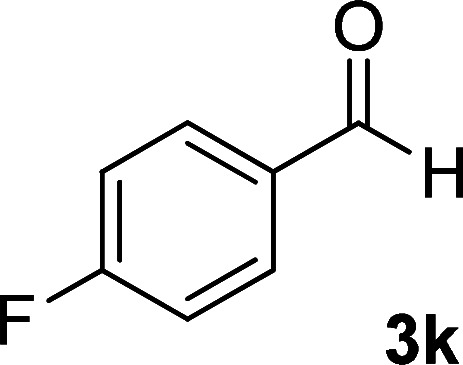	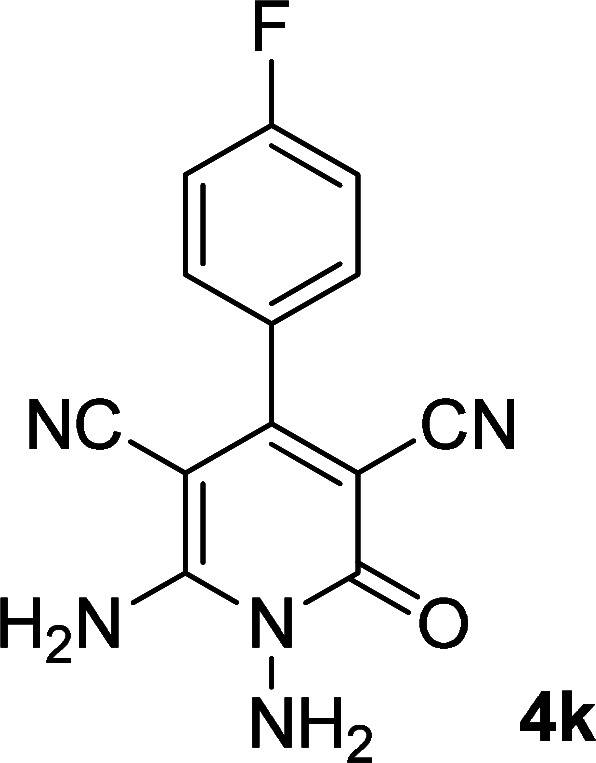	11	83	338–340
12	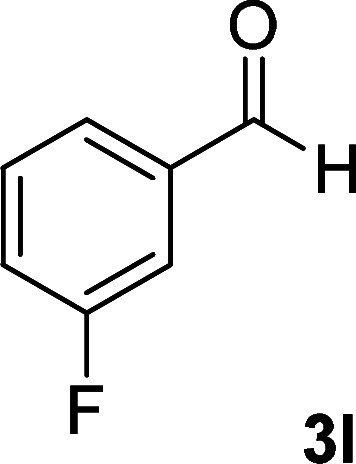	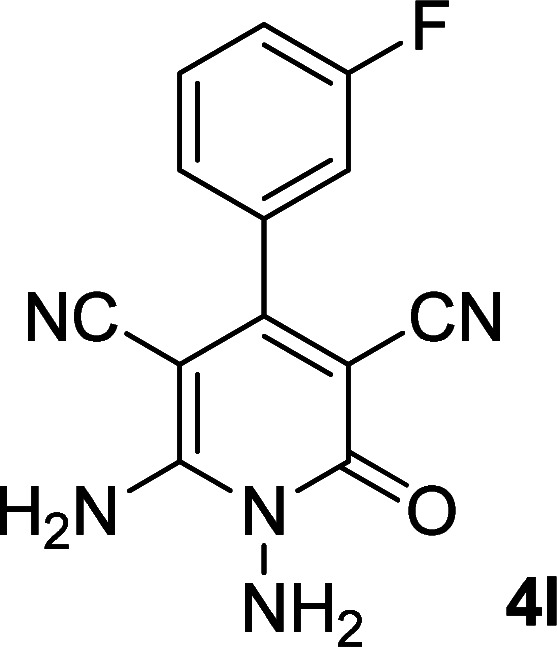	13	78	324–326

a All reactions were carried out with cyanoacetohydrazide 1 (1 mmol), malononitrile 2a (1 mmol), aromatic aldehydes 3 (1 mmol) and piperidine (0.02 mmol) in water (10 ml).

By comparing the reaction rates in the table above, it is found that for aldehydes with an electron withdrawing group on the ring (nitro and halogens), the reaction rate is the highest and with electron donating groups (methoxy), the speed is the lowest.

**Fig. 1:**
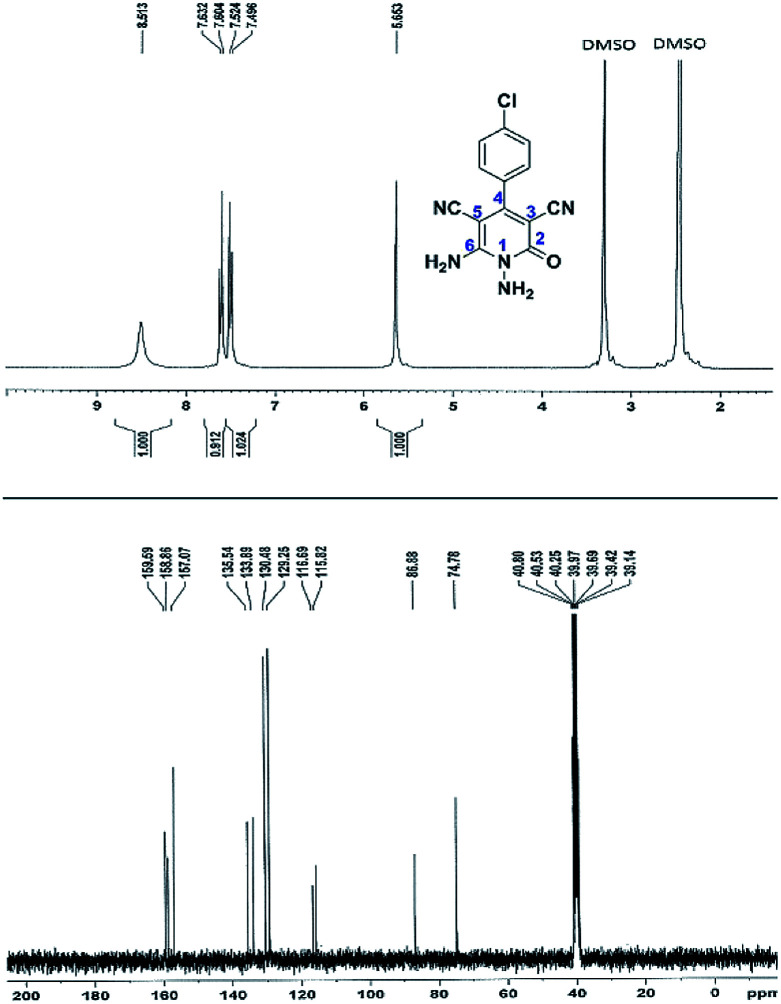
The ^1^H and ^13^C NMR spectrums of 4a.

Here we investigate the ^1^H and ^13^C NMR spectra of product 4a. As shown in Fig. 1, the ^1^H NMR spectrum of 4a showed two signals at *δ* 5.65 and 8.51 ppm identified as N–NH_2_ and NH_2_ groups respectively. These peaks were exchangeable with D_2_O. The four protons of aromatic ring appeared at *δ* 7.50 and 7.62 ppm as two doublets. The ^1^H-decoupled ^13^C NMR spectrum of 4a indicated 11 distinct resonances in accordance to desired structure. Two signals of C-5 and C-3 of pyridone ring were observed at *δ* 74.7 and 86.8 ppm respectively. The signals at *δ* 115.8 and 116.6 ppm were related to two nitrile groups. The signals at *δ* 129.2, 130.4, 133.8 and 135.5 ppm were assigned to carbons of aryl ring. The carbons of C-4 and C-6 appeared at *δ* 157.0 and 158.8 ppm. The carbonyl group (C-2) was observed at *δ* 159.5 ppm (Fig. 1).

The mass spectrum of 4a displayed the molecular-ion peak at *m*/*z* 285 (100%) in agreement with the proposed structure. The IR spectrum of this compound showed absorption bands at 3390, 3283 due to the NH_2_ groups, stretching vibration of nitrile groups at 2216, strong absorption of carbonyl group at 1623, N–H bending band at 1525, stretching vibration of C=C of aromatic ring at 1465 and C–N band at 1227 cm^−1^.

According to the above findings, we used ethyl cyanoacetate instead of malononitrile. As a result, ethyl 1,6-diamino-4-aryl-3-cyano-2-pyridone-5-carboxylate derivatives 5a–e were synthesized *via* the reaction of cyanoacetohydrazide 1, ethyl cyanoacetate 2b and the five aldehydes 3 (Table 2). The best results in this type of reactions were obtained in the mixture of water and ethanol (1 : 1, v/v) in the presence of piperidine at reflux conditions (these reactions in water led to the hydrazone products without participation of ethyl cyanoacetate).

**Table 2:** Synthesis of ethyl 1,6-diamino-4-aryl-3-cyano-2-oxo-1,2-dihydropyridine-5-carboxylate 5a–e^a^

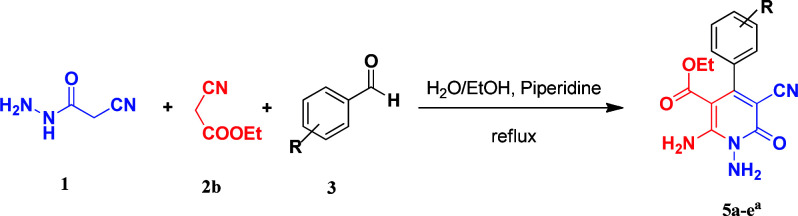					
Entry	Aromatic aldehyde	Product	Time (h)	Yield (%)	M.p. (°C)
1	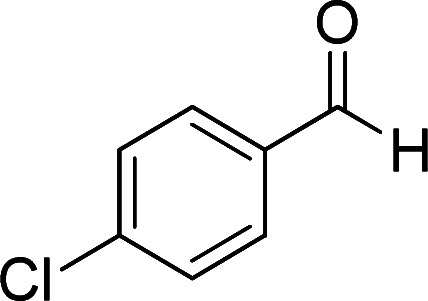	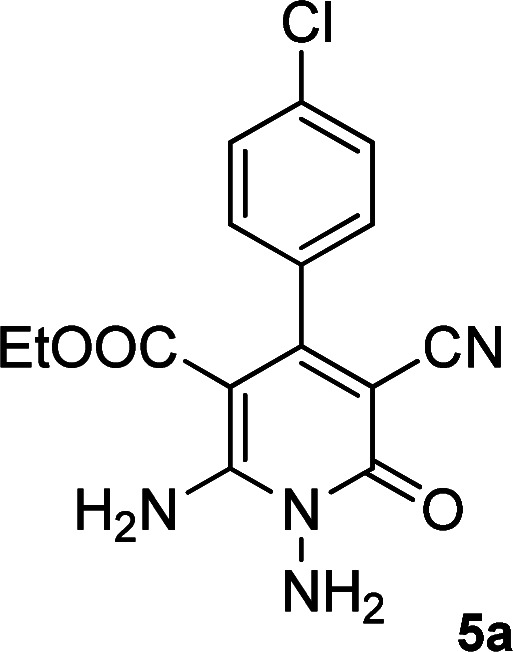	20	87	245–247
2	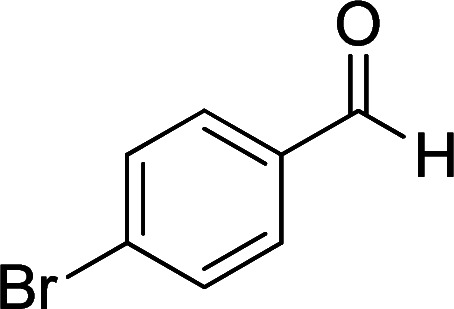	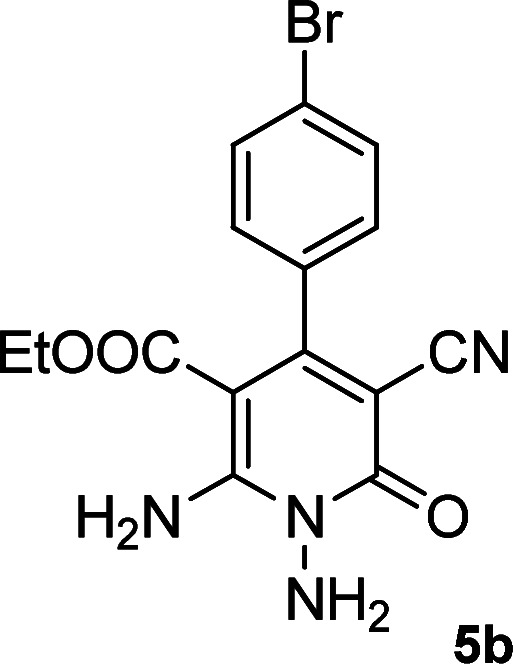	20	85	280–282
3	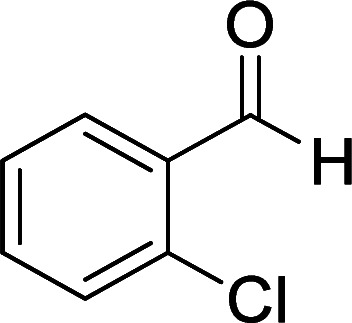	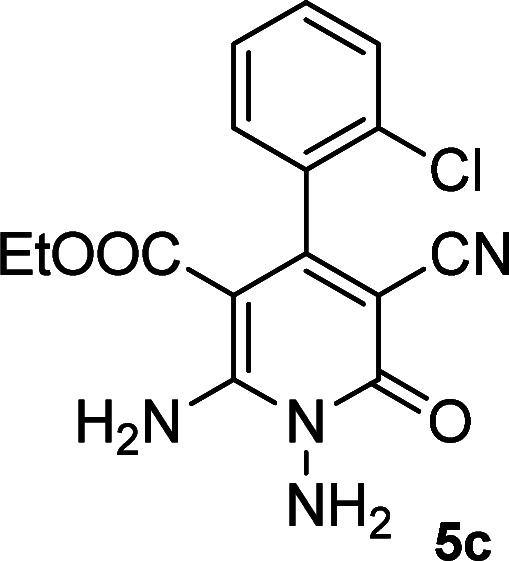	22	80	322–324
4	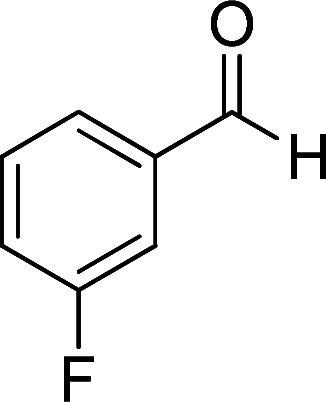	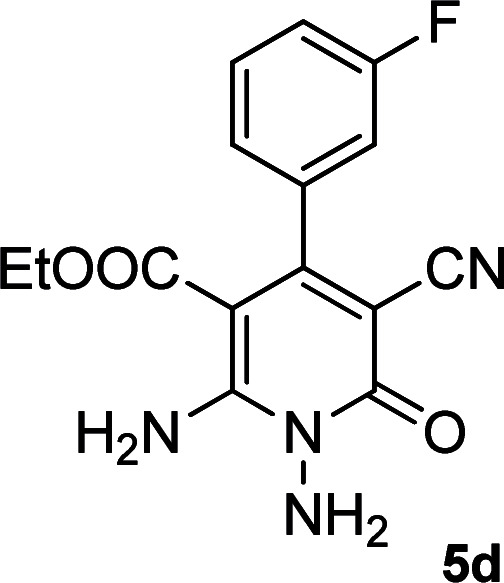	24	75	218–220
5	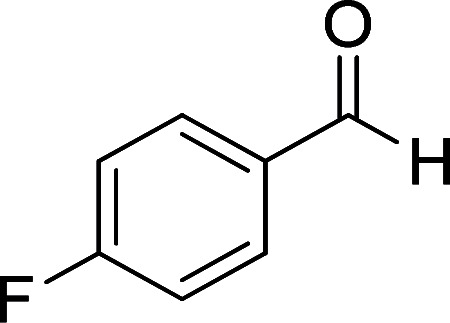	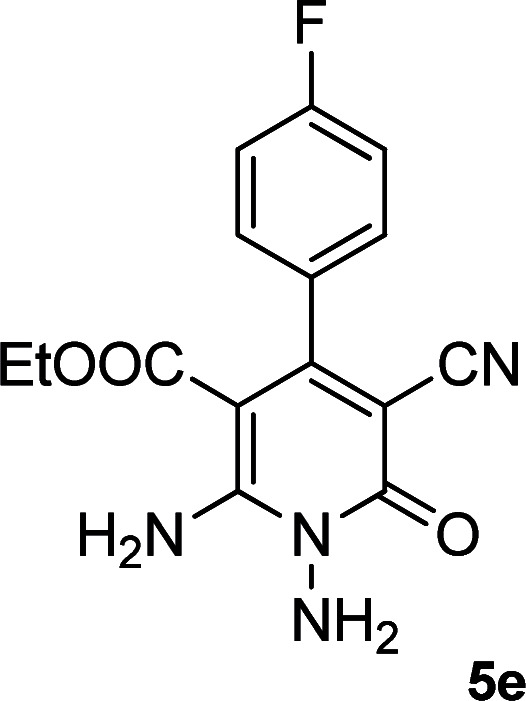	24	75	237–239

a All reactions were carried out with cyanoacetohydrazide 1 (1 mmol), ethyl cyanoacetate 2b (1 mmol), aromatic aldehydes 3 (1 mmol) and piperidine (0.02 mmol) in water/EtOH (5 : 5 mL).

The reaction with other aromatic aldehydes was carried out similar to the high work, but did not result in the expected products and the reaction mixture showed several spots in TLC (the desired product was not isolable).

It is interesting to note that when methyl cyanoacetate was used instead of ethyl cyanoacetate in the above reaction, the same ethyl carboxylate products 5a–e were obtained.

**Fig. 2:**
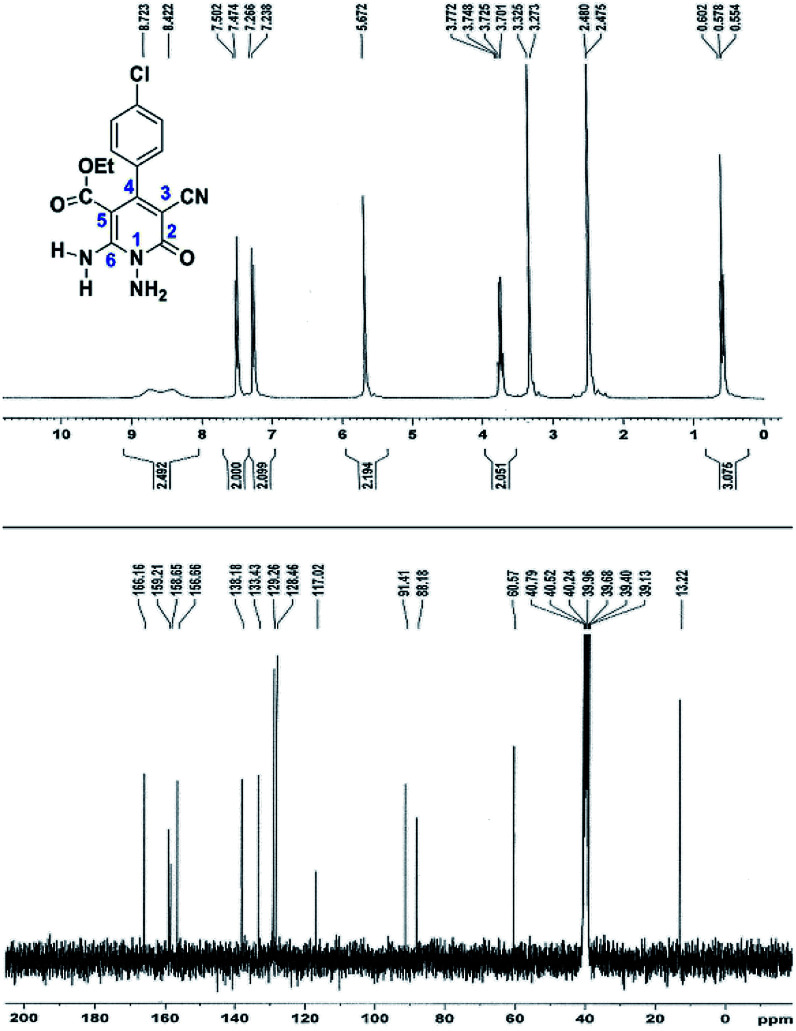
The ^1^H and ^13^C NMR spectrums of 5a.

The spectral analysis of IR, ^1^H NMR, ^13^C NMR and mass spectrums of 5a–e confirmed the formation of expected structures (see the ESI†). For example the ^1^H NMR spectrum of 5a showed a triplet at *δ* 0.57 and a quartet at *δ* 3.73 ppm related to the CH_3_ and CH_2_ groups. Protons of N–NH_2_ group was observed at *δ* 5.56 ppm. The signals of aromatic ring specified at *δ* 7.25 and 7.48 ppm as two doublets. Two broad signals related to the NH_2_ group (position 6) appeared at *δ* 8.42 and 8.72 ppm separately as a result of intramolecular hydrogen bonding. The ^1^H-decoupled ^13^C NMR spectrum of 5a displayed 13 distinct signals in accordance with expected structure. The carbons of methyl and methylene groups appeared at *δ* 13.2 and 60.5 ppm respectively. The signals of C-3 and C-5 of pyridone ring were observed at *δ* 88.1 and 91.4 ppm. The nitrile group was assigned at *δ* 117.0 The signals at *δ* 129.2, 130.4, 133.8 and 135.5 ppm were related to carbons of aryl ring. The carbons of C-4 and C-6 appeared at *δ* 156.6 and 158.6 ppm. Two carbonyl groups (C-2 and CO_2_Et) were observed at *δ* 159.2 and 166.16 ppm respectively (Fig. 2).

The mass spectrum of 5a afforded a molecular-ion peak at *m*/*z* 332 (100%) in conformity with the proposed product. The IR spectrum of this compound indicated absorption bands due to NH_2_ groups broadly at 3387 and 3266, stretching vibrations of aliphatic C–H bands at 2984, 2923, one vibration of nitrile group at 2215, strong absorptions of carbonyl groups at 1686 and 1651, N–H bending band at 1536, stretching vibration of C=C of aromatic ring at 1473 and C–N stretching band at 1207 cm^−1^.

In the third section, we used cyanoacetamide as activated nitrile compound. As a result, the reaction of cyanoacetohydrazide 1, cyanoacetamide 2c and some aromatic aldehydes 3 led to 1,6-diamino-4-aryl-3-cyano-2-pyridone-5-carboxamide derivatives 6a–e (Table 3). It was investigated that the best results were obtained in the mixture of water and ethanol (1 : 2, v/v) in the presence of piperidine at reflux conditions (similar to the previous reactions, when water was used alone, the hydrazone compounds were formed as only products).

**Table 3:** Synthesis of 1,6-diamino-4-aryl-3-cyano-2-oxo-1,2-dihydropyridine-5-carboxamide 6a–e^a^

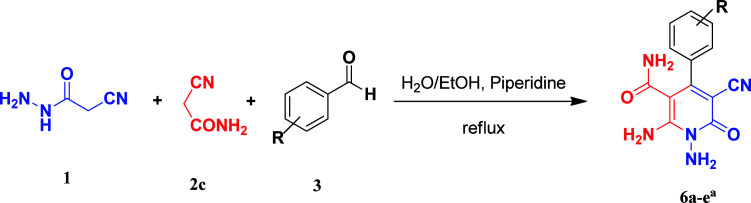					
Entry	Aromatic aldehyde	Product	Time (h)	Yield (%)	M.p. (°C)
1	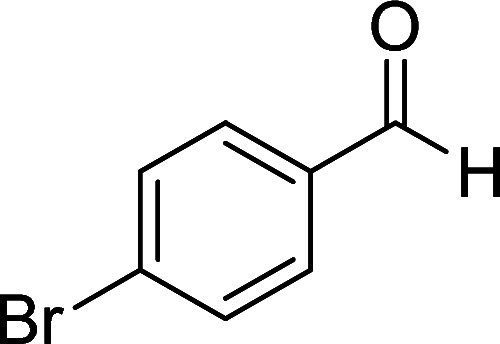	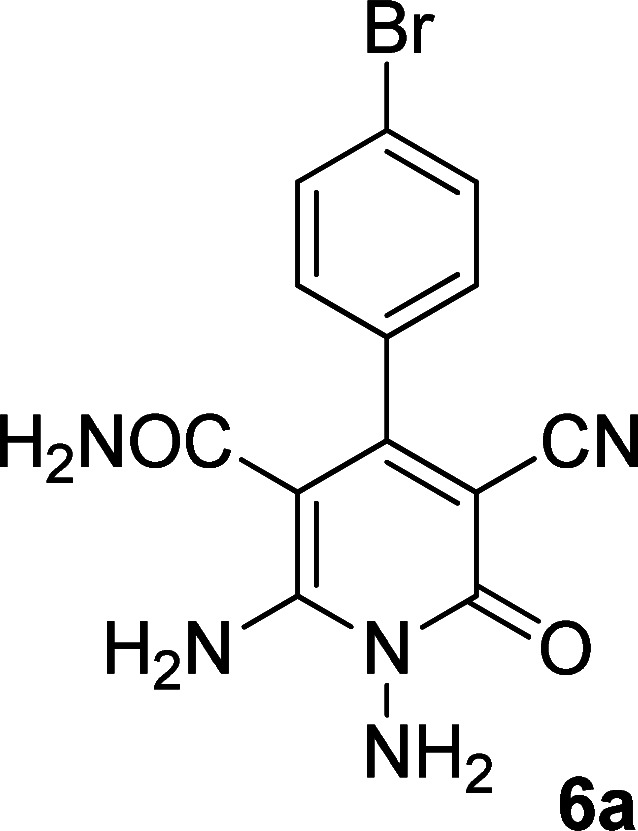	20	80	245–247
2	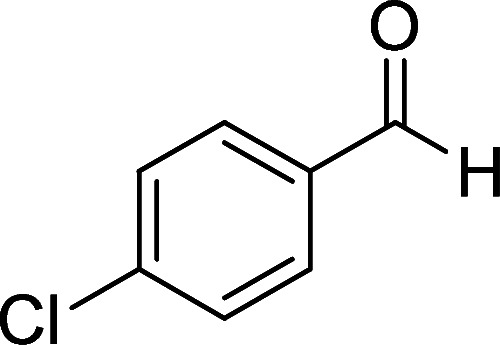	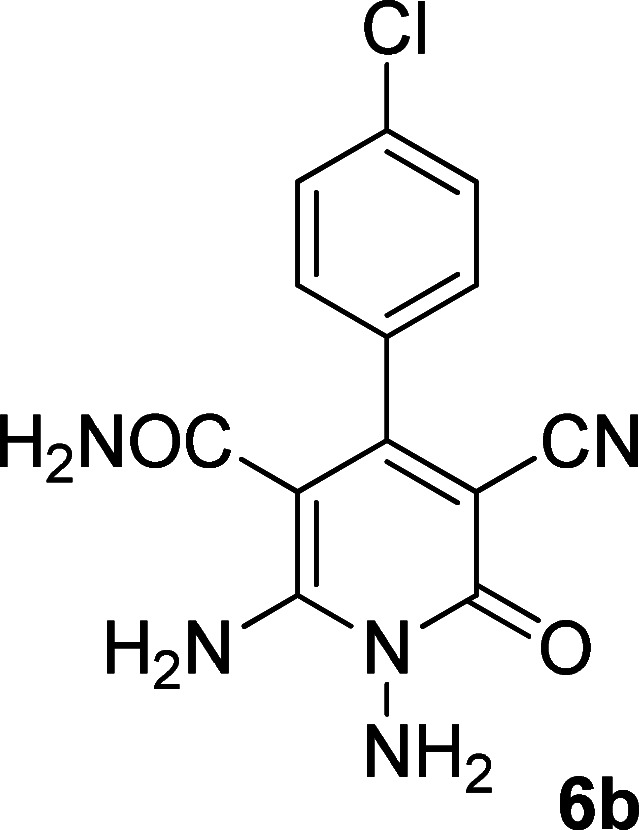	20	78	280–282
3	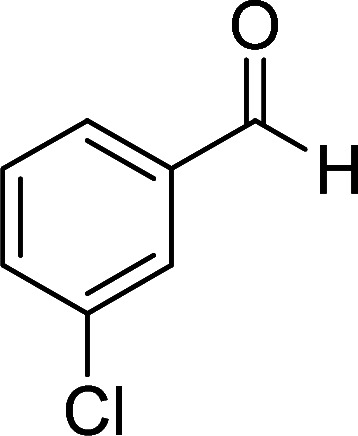	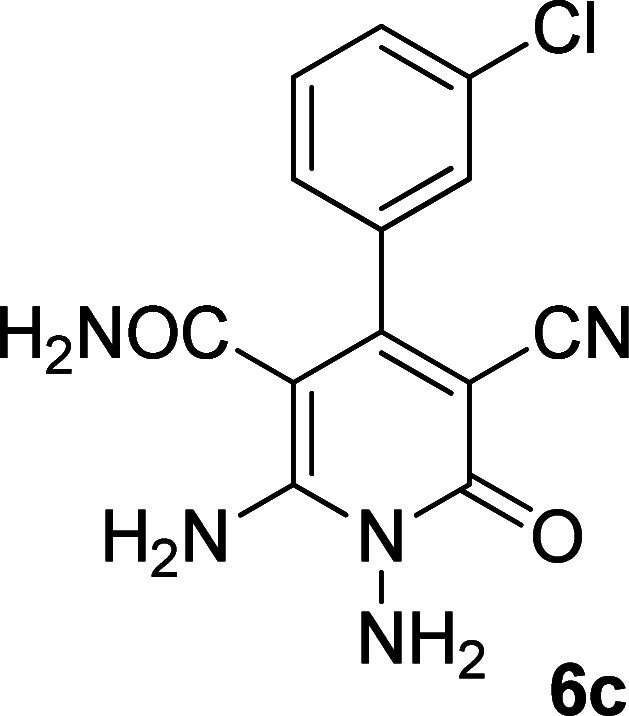	22	75	322–324
4	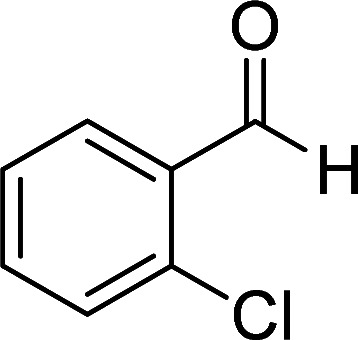	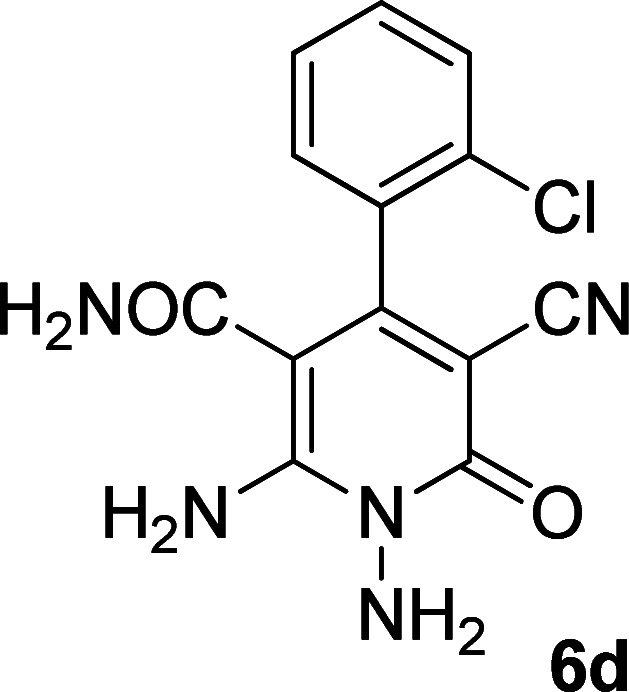	24	70	218–220
5	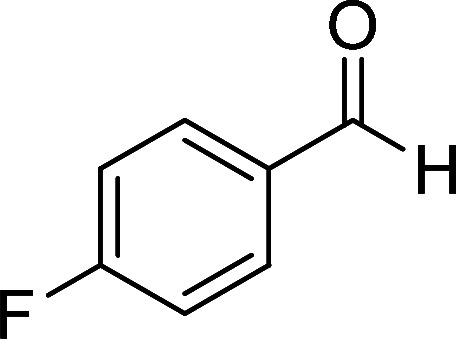	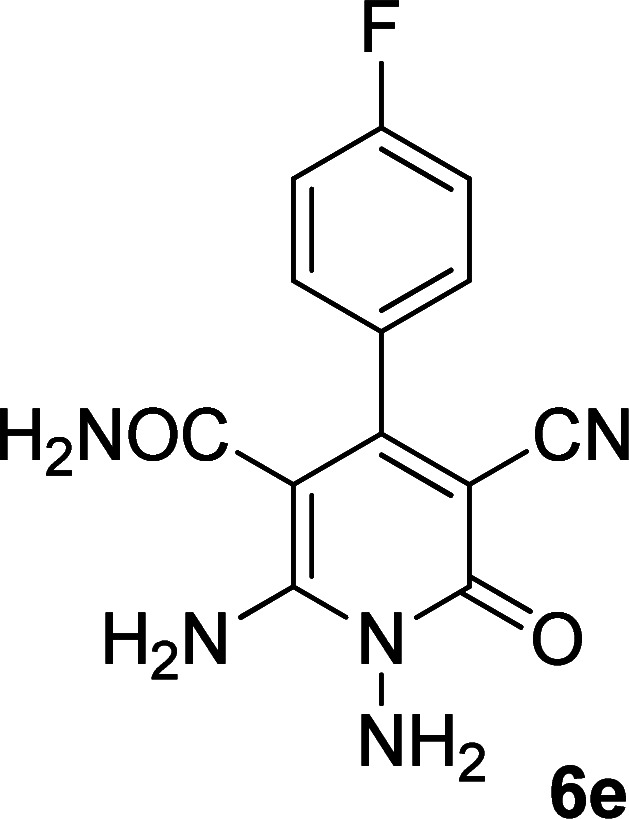	24	65	237–239

a All reactions were carried out with cyanoacetohydrazide 1 (1 mmol), cyanoacetamide 2c (1 mmol), aromatic aldehydes 3 (1 mmol) and piperidine (0.02 mmol) in water/EtOH (3 : 6 mL).

This reaction with other aromatic aldehydes was carried out similar to the first work, but did not led to the pure products and the reaction mixture contained several spots in TLC (the expected product was not separable).

**Fig. 3:**
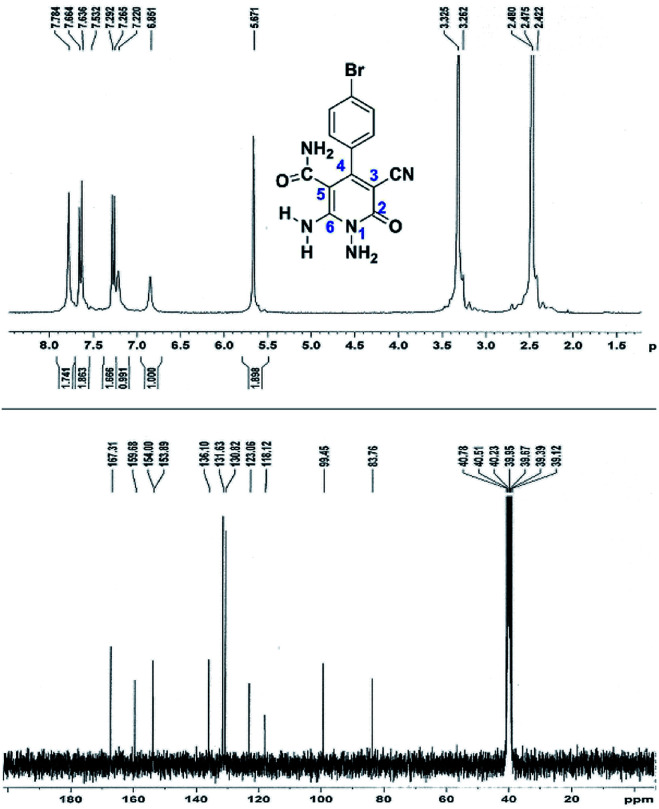
The ^1^H and ^13^C NMR spectrums of 6a.

The structures of products 6a–e were deduced from their IR, ^1^H NMR, ^13^C NMR spectroscopic and mass spectrometric data (see the ESI†). Here we study the ^1^H and ^13^C NMR spectra of 6a. As seen in Fig. 3, the ^1^H NMR spectrum of 6a indicated a singlet at *δ* 5.67 ppm identified as the N–NH_2_ group. Two singlet broad signals at *δ* 6.85 and 7.22 ppm were assigned to the amino group (on C-6). Two doublets at *δ* 7.28 and 7.65 ppm were related to aryl ring. The amidic NH_2_ group appeared at *δ* 7.78 ppm. The ^1^H-decoupled ^13^C NMR spectrum of 6a showed 11 resonances which confirmed the assumed structure. The signals of C-3 and C-5 were observed at *δ* 83.7 and 99.4 ppm respectively. The nitrile group appeared at *δ* 118.1 ppm. The signals at *δ* 123.0, 130.8, 131.6 and 136.1 were related to aryl ring. The carbons of C-4 and C-6 were observed at *δ* 153.8 and 154.0 ppm. Two carbonyl groups (C-2 and CONH_2_) were assigned at *δ* 159.6 and 167.3 ppm respectively (Fig. 3).

The mass spectrum of 6a showed the molecular-ion peak at *m*/*z* 347 (100%) that was in accordance with the desired structure. The IR spectrum of compound 6a showed absorption broad bands at 3394, 3290 related to the NH_2_ groups, stretching vibrations of nitrile group at 2205, strong absorption of carbonyl groups at 1662 and 1612, N–H bending band at 1571, stretching vibration of C=C of aromatic ring at 1463 and C–N stretching band at 1213 cm^−1^.

**Scheme 4:**
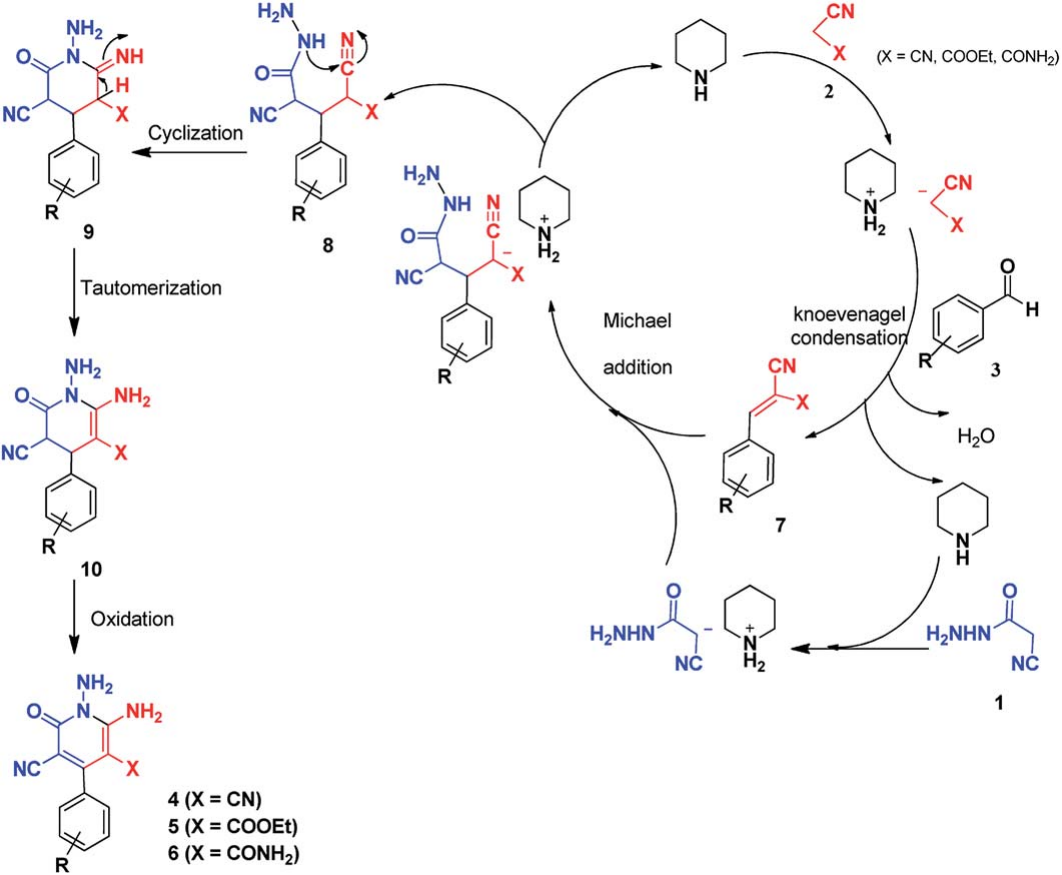
Proposed mechanism for the formation of products 4, 5, 6.

A plausible mechanism for the formation of 2-pyridones 4, 5 and 6 is depicted in Scheme 4. Initial condensation of activated nitriles 2 with aromatic aldehydes 3 in the presence of piperidine, leads to the Knoevenagel products 7. The methylene group of cyanoacetohydrazide loses proton with the catalyst so subsequent Michael addition of cyanoacetohydrazide 1 to adduct 7 affords intermediate 8 which undergoes intramolecular cyclisation *via* nucleophilic addition of –NH to nitrile group to give the corresponding 1-amino-6-imino-2-piperidinone 9. Successive imine–enamine tautomerization followed by dehydrogenation affords desired products (Scheme 4).

It is necessary to mention this point that cyanoacetohydrazide could conceivably lead to condensation at the hydrazide group with aldehydes, but even when the hydrazone structures were synthesized and added to compounds 2, the same pyridone products were obtained. In fact, the most important part of these reactions was the prevention of hydrazone formation, which was controlled with the mentioned conditions.

## Experimental

### Materials

All commercially available reagents and other solvents were purchased and used without further purification. The NMR spectra were recorded with a Bruker DRX-300 Avance instrument (300 MHz for ^1^H and 75.4 MHz for ^13^C) with DMSO-*d*_6_ as solvent. Chemical shifts are given in ppm (*δ*), and coupling constant (*J*) are reported in hertz (Hz). Melting points were measured with an electrotherma1 9100 apparatus. Mass spectra were recorded with an Agilent 5975C VL MSD with Triple-Axis Detector operating at an ionization potential of 70 eV. IR spectra were measured with, Bruker Tensor 27 spectrometer (*ν̄* in cm^−1^). Elemental analyses for C, H and N were performed using a PerkinElmer 2004 series [II] CHN elemental analyzer.

### General procedure for the synthesis of 1,6-diamino-4-aryl-2-oxo-1,2-dihydropyridine-3,5-dicarbonitrile (4a–l)

The stoichiometric mixture of cyanoacetohydrazide 1 (1 mmol, 0.99 g), malononitrile 2a (1 mmol, 0.66 g), aromatic aldehyde 3a–l (1 mmol) and piperidine (20% mol, 0.019 ml) in H_2_O (10 ml) was stirred at room temperature. The progress of the reaction was monitored by TLC using ethyl acetate/*n*-hexane (1 : 1). After completion of the reaction, the precipitated product was collected by filtration and washed with warm ethanol to give the pure products 4a–l.

#### 1,6-Diamino-4-(4-chlorophenyl)-2-oxo-1,2-dihydropyridine-3,5-dicarbonitrile (4a)

White solid; yield: 0.265 g (93%); mp: 340 °C (dec.); IR (KBr) (*ν*_max_/cm^−1^): 3390, 3283, 3197, 2216, 1623, 1525, 1465, 1227, 841, 766; ^1^H NMR (300 MHz, DMSO): *δ* 5.65 (s, 2H, N–NH_2_), 7.51 (d, *J* = 8.4 Hz, 2H, ArH), 7.62 (d, *J* = 8.4 Hz, 2H, ArH), 8.51 (brs, 2H, NH_2_); ^13^C{^1^H} NMR (75.4 MHz, DMSO): *δ* 74.7 (*C*–CN), 86.8 (*C*–CN), 115.8 (CN), 116.6 (CN), 129.2, 130.4, 133.8, 135.5 (Ar), 157 (C-4), 158.8 (C–NH_2_), 159.5 (C=O); MS (EI, 70 eV): *m*/*z* (%) = 287 (35) [M + 2]^+^, 286 (18) [M + 1]^+^, 285 (100) [M]^+^, 256 (19), 241 (20), 205 (13), 193 (7), 161 (10), 138 (5), 111 (4), 88 (3), 75 (6), 58 (3), 43 (2); anal. calcd for C_13_H_8_ClN_5_O: C, 54.65; H, 2.82; N, 24.51. Found: C, 54.65; H, 2.71; N, 24.39.

#### 1,6-Diamino-4-(4-bromophenyl)-2-oxo-1,2-dihydropyridine-3,5-dicarbonitrile (4b)

White solid; yield: 0.289 g (88%); mp: 355 °C (dec.); IR (KBr) (*ν*_max_/cm^−1^): 3450, 3396, 3258, 2212, 1600, 1522, 1466, 1297, 829, 613; ^1^H NMR (300 MHz, DMSO): *δ* 5.64 (s, 2H, N–NH_2_), 7.43 (d, *J* = 8.1 Hz, 2H, ArH), 7.59 (d, *J* = 8.1 Hz, 2H, ArH), 8.50 (brs, 2H, NH_2_); ^13^C{^1^H} NMR (75.4 MHz, DMSO): *δ* 74.7 (*C*–CN), 86.7 (*C*–CN), 115.8 (CN), 116.6 (CN), 124.3, 130.6, 132.1, 134.2 (Ar), 157 (C-4), 158.8 (C–NH_2_), 159.5 (C=O); MS (EI, 70 eV): *m*/*z* (%) = 331 (98) [M + 2]^+^, 330 (21) [M + 1]^+^, 329 (100) [M]^+^, 300 (12), 285 (17), 258 (6), 222 (14), 205 (26), 193 (14), 165 (12), 152 (13), 126 (14), 100 (9), 88 (7), 75 (10), 58 (6), 43 (3); anal. calcd for C_13_H_8_BrN_5_O: C, 47.29; H, 2.44; N, 21.21. Found: C, 47.26; H, 2.28; N, 21.07.

#### 1,6-Diamino-4-(4-nitrophenyl)-2-oxo-1,2-dihydropyridine-3,5-dicarbonitrile (4c)

Yellow solid; yield: 0.269 g (91%); mp: 360 °C (dec.); ^1^H NMR (300 MHz, DMSO): *δ* 5.50 (s, 2H, N–NH_2_), 7.84 (d, *J* = 9 Hz, 2H, ArH), 8.06 (brs, 2H, NH_2_); 8.44 (d, *J* = 9 Hz, 2H, ArH), ^13^C{^1^H} NMR (75.4 MHz, DMSO): *δ* 74.6 (*C*–CN), 86.7 (*C*–CN), 115.5 (CN), 116.4 (CN), 124.3, 130.2, 141.3, 148.9 (Ar), 157.0 (C-4), 158 (C–NH_2_), 159.4 (C=O); MS (EI, 70 eV): *m*/*z* (%) = 297 (17) [M + 1]^+^, 296 (100) [M]^+^, 267 (11), 252 (14), 230 (3), 205 (4), 193 (5), 165 (8), 152 (7), 126 (5), 100 (4), 88 (4), 58 (3), 43 (2); anal. calcd for C_13_H_8_N_6_O_3_: C, 52.71; H, 2.72; N, 28.37. Found: C, 52.64; H, 2.53; N, 28.19.

#### 1,6-Diamino-4-(4-methoxyphenyl)-2-oxo-1,2-dihydropyridine-3,5-dicarbonitrile (4d)

Pale yellow solid; yield: 0.233 g (83%); mp: 321–323 °C; IR (KBr) (*ν*_max_/cm^−1^): 3393, 3316, 3264, 2211, 1640, 1513, 1419, 1256, 1023, 837; ^1^H NMR (300 MHz, DMSO): *δ* 3.81 (s, 3H, OCH_3_), 5.62 (s, 2H, N–NH_2_), 7.07 (d, *J* = 8.7 Hz, 2H, ArH), 7.43 (d, *J* = 8.7 Hz, 2H, ArH), 8.40 (brs, 2H, NH_2_); ^13^C{^1^H} NMR (75.4 MHz, DMSO): *δ* 55.79 (OCH_3_), 74.7 (*C*–CN), 86.7 (*C*–CN), 114.4 (CN), 116.2 (CN), 117, 126.9, 130.3, 157.7 (Ar), 159.7 (C-4), 159.8 (C–NH_2_), 161.1 (C=O); MS (EI, 70 eV): *m*/*z* (%) = 282 (18) [M + 1]^+^, 281 (100) [M]^+^, 266 (4), 252 (18), 237 (27), 210 (13), 195 (8), 180 (6), 167 (5), 140 (3), 114 (9), 88 (6), 58 (4), 43 (7); anal. calcd for C_14_H_11_N_5_O_2_: C, 59.78; H, 3.94; N, 24.90. Found: C, 59.39; H, 3.79; N, 24.85.

#### 1,6-Diamino-4-(3-methoxyphenyl)-2-oxo-1,2-dihydropyridine-3,5-dicarbonitrile (4e)

White solid; yield: 0.238 g (85%); mp: 265–267 °C; IR (KBr) (*ν*_max_/cm^−1^): 3425, 3314, 3194, 2954, 2205, 1682, 1617, 1526, 1464, 1292, 1159, 884; ^1^H NMR (300 MHz, DMSO): *δ* 3.78 (s, 3H, OCH_3_), 5.57 (s, 2H, N–NH_2_), 6.99–7.10 (m, 3H, ArH), 7.44 (t, *J* = 8.1 Hz, 1H, ArH), 8.44 (brs, 2H, NH_2_); ^13^C{^1^H} NMR (75.4 MHz, DMSO): *δ* 55.78 (OCH_3_), 74.7 (*C*–CN), 86.8 (*C*–CN), 114.1 (CN), 115.8 (CN), 116, 116.7, 120.5, 130.3, 136.2, 157 (Ar), 159.4 (C-4), 159.6 (C–NH_2_), 159.7 (C=O); MS (EI, 70 eV): *m*/*z* (%) = 282 (18) [M + 1]^+^, 281 (100) [M]^+^, 280 (22), 265 (5), 252 (18), 237 (16), 210 (3), 195 (7), 180 (6), 167 (5), 140 (6), 114 (6), 88 (5), 63 (5), 43 (2); anal. calcd for C_14_H_11_N_5_O_2_: C, 59.78; H, 3.94; N, 24.90. Found: C, 59.60; H, 3.84; N, 24.85.

#### 1,6-Diamino-4-phenyl-2-oxo-1,2-dihydropyridine-3,5-dicarbonitrile (4f)

White solid; yield: 0.225 g (90%); mp: 332–334 °C; IR (KBr) (*ν*_max_/cm^−1^): 3444, 3397, 3240, 2212, 1630, 1525, 1469, 1219, 858, 739; ^1^H NMR (300 MHz, DMSO): *δ* 5.64 (s, 2H, N–NH_2_), 7.36–7.52 (m, 5H, ArH), 8.45 (brs, 2H, NH_2_); ^13^C{^1^H} NMR (75.4 MHz, DMSO): *δ* 74.7 (*C*–CN), 86.8 (*C*–CN), 115.9 (CN), 116.8 (CN), 128.4, 129, 130.6, 135 (Ar), 157.1 (C-4), 159.7 (C–NH_2_), 160 (C=O); MS (EI, 70 eV): *m*/*z* (%) = 252 (18) [M + 1]^+^, 251 (100) [M]^+^, 236 (2), 222 (21), 207 (26), 194 (7), 180 (17), 165 (8), 127 (14), 88 (5), 77 (12), 51 (10), 43 (4); anal. calcd for C_13_H_9_N_5_O: C, 62.15; H, 3.61; N, 27.87. Found: C, 61.79; H, 3.56; N, 27.62.

#### 1,6-Diamino-4-(2-chlorophenyl)-2-oxo-1,2-dihydropyridine-3,5-dicarbonitrile (4g)

White solid; yield: 0.247 g (87%); mp: 305–307 °C; IR (KBr) (*ν*_max_/cm^−1^): 3411, 3305, 3205, 2213, 1678, 1561, 1466, 1228, 880, 757; ^1^H NMR (300 MHz, DMSO): *δ* 5.66 (s, 2H, N–NH_2_), 7.41–7.66 (m, 4H, ArH), 8.59 (brs, 2H, NH_2_); ^13^C{^1^H} NMR (75.4 MHz, DMSO): *δ* 75.3 (*C*–CN), 87.5 (*C*–CN), 115.1 (CN), 116 (CN), 128.2, 130.1, 130.2, 131, 132.1, 134.2 (Ar), 157 (C-4), 157.7 (C–NH_2_), 159.5 (C=O); MS (EI, 70 eV): *m*/*z* (%) = 287 (37) [M + 2]^+^, 286 (30) [M + 1]^+^, 285 (100) [M]^+^, 276 (3), 250 (23), 222 (4), 205 (13), 193 (10), 161 (12), 138 (10), 111 (5), 88 (6), 67 (3), 58 (4), 43 (2); anal. calcd for C_13_H_8_ClN_5_O: C, 54.65; H, 2.82; N, 24.51. Found: C, 55.14; H, 2.45; N, 24.31.

#### 1,6-Diamino-4-(3-chlorophenyl)-2-oxo-1,2-dihydropyridine-3,5-dicarbonitrile (4h)

White solid; yield: 0.242 g (85%); mp: 292–294 °C; ^1^H NMR (300 MHz, DMSO): *δ* 5.66 (s, 2H, N–NH_2_), 7.42–7.62 (m, 4H, ArH), 8.52 (brs, 2H, NH_2_); ^13^C{^1^H} NMR (75.4 MHz, DMSO): *δ* 74.8 (*C*–CN), 86.9 (*C*–CN), 115.7 (CN), 116.6 (CN), 127.2, 128.2, 130.5, 131.1, 133.6, 137 (Ar), 157 (C-4), 158.4 (C–NH_2_), 159.5 (C=O); anal. calcd for C_13_H_8_ClN_5_O: C, 54.65; H, 2.82; N, 24.51. Found: C, 55.53; H, 2.67; N, 24.50.

#### 1,6-Diamino-2-oxo-4-(4-(trifluoromethyl)phenyl)-1,2-dihydropyridine-3,5-dicarbonitrile (4i)

Pale brown solid; yield: 0.255 g (80%); mp: 290–293 °C; ^1^H NMR (300 MHz, DMSO): *δ* 5.61 (s, 2H, N–NH_2_), 7.72 (d, *J* = 8.1 Hz, 2H, ArH), 7.93 (d, *J* = 8.1 Hz, 2H, ArH), 8.57 (brs, 2H, NH_2_); ^13^C{^1^H} NMR (75.4 MHz, DMSO): *δ* 74.7 (*C*–CN), 86.8 (*C*–CN), 115.6 (CN), 116.5 (CN), 122.5 (CF_3_), 126.1, 129.5, 131.03, 139.1, 135.5 (Ar), 157.1 (C-4), 158.6 (C–NH_2_), 159.5 (C=O); MS (EI, 70 eV): *m*/*z* (%) = 320 (17) [M + 1]^+^, 319 (100) [M]^+^, 300 (12), 275 (24), 248 (10), 195 (11), 176 (10), 145 (9), 111 (4), 99 (6), 88 (5), 69 (15), 57 (8), 43 (11); anal. calcd for C_14_H_8_F_3_N_5_O: C, 52.67; H, 2.53; N, 21.94. Found: C, 52.35; H, 2.81; N, 21.67.

#### 1,6-Diamino-4-(3,4-dimethoxyphenyl)-2-oxo-1,2-dihydropyridine-3,5-dicarbonitrile (4j)

Pale yellow solid; yield: 0.233 g (75%); mp: 290–292 °C; IR (KBr) (*ν*_max_/cm^−1^): 3402, 3291, 2966, 2845, 2215, 1673, 1613, 152, 1464, 1269, 1153, 1019, 866, 762; ^1^H NMR (300 MHz, DMSO): *δ* 3.77 (s, 3H, OCH_3_), 3.81 (s, 3H, OCH_3_), 5.64 (s, 2H, N–NH_2_), 7.02–7.10 (m, 3H, ArH), 8.40 (brs, 2H, NH_2_); ^13^C{^1^H} NMR (75.4 MHz, DMSO): *δ* 56 (OCH_3_), 56.1 (OCH_3_), 74.7 (*C*–CN), 86.7 (*C*–CN), 111.84, 112.4 (Ar), 116.2 (CN), 117.1 (CN), 121.6, 126.9, 148.6, 150.7 (Ar), 157.1 (C-4), 159.7 (C–NH_2_), 159.8 (C=O); MS (EI, 70 eV): *m*/*z* (%) = 312 (24) [M + 1]^+^, 311 (100) [M]^+^, 296 (7), 268 (13), 237 (5), 209 (4), 181 (4), 151 (10), 127 (3), 101 (3), 88 (2), 77 (3), 58 (2), 43 (3); anal. calcd for C_15_H_13_N_5_O_3_: C, 57.87; H, 4.21; N, 22.50. Found: C, 57.60; H, 4.20; N, 22.22.

#### 1,6-Diamino-4-(4-fluorophenyl)-2-oxo-1,2-dihydropyridine-3,5-dicarbonitrile (4k)

White solid; yield: 0.223 g (83%); mp: 338–340 °C; IR (KBr) (*ν*_max_/cm^−1^):3390, 3287, 3196, 2211, 1627, 1516, 1463, 1224, 1157, 945, 848, 616; ^1^H NMR (300 MHz, DMSO): *δ* 5.65 (s, 2H, N–NH_2_), 7.35–7.57 (m, 4H, ArH), 8.49 (brs, 2H, NH_2_); ^13^C{^1^H} NMR (75.4 MHz, DMSO): *δ* 74.9 (*C*–CN), 87 (*C*–CN), 115.9 (CN), 116 (CN), 116.5, 131.1, 131.4, 163.4 (Ar), 157 (C-4), 159 (C–NH_2_), 159.6 (C=O); anal. calcd for C_13_H_8_FN_5_O: C, 57.99; H, 2.99; N, 26.01. Found: C, 57.95, 2.89; 25.87.

#### 1,6-Diamino-4-(3-fluorophenyl)-2-oxo-1,2-dihydropyridine-3,5-dicarbonitrile (4l)

White solid; yield: 0.209 g (78%); mp: 340 °C (dec.); IR (KBr) (*ν*_max_/cm^−1^): 3382, 3298, 3206, 2215, 1619, 1527, 1465, 1244, 1019, 965, 884, 761; ^1^H NMR (300 MHz, DMSO): *δ* 5.66 (s, 2H, N–NH_2_), 7.29–7.62 (m, 4H, ArH), 8.52 (brs, 2H, NH_2_); ^13^C{^1^H} NMR (75.4 MHz, DMSO): *δ* 74.8 (*C*–CN), 86.9 (*C*–CN), 115.6 (Ar), 115.8 (CN), 116.5 (CN), 117.5, 124.7, 131.4, 137.1, 162.1 (Ar), 157 (C-4), 158.5 (C–NH_2_), 159.5 (C=O); anal. calcd for C_13_H_8_FN_5_O: C, 57.99; H, 2.99; N, 26.01. Found: C, 56.73; H, 3.83; N, 24.11.

### General procedure for the synthesis of ethyl 1,6-diamino-4-aryl-3-cyano-2-oxo-1,2-dihydropyridine-5-carboxylate (5a–e)

The stoichiometric mixture of cyanoacetohydrazide 1 (1 mmol, 0.99 g), ethyl cyanoacetate 2b (1 mmol, 0.113 g), aromatic aldehyde (1 mmol) and piperidine (20% mol, 0.019 ml) in a mixture of H_2_O and ethanol (5 : 5 ml) was stirred at 80 °C. Upon completion the reaction as monitored by TLC (using ethyl acetate/*n*-hexane (1 : 1)), the precipitated product was collected by filtration and washed with H_2_O/EtOH to give the pure products 5a–e.

#### Ethyl 1,6-diamino-4-(4-chlorophenyl)-3-cyano-2-oxo-1,2-dihydropyridine-5-carboxylate (5a)

White solid; yield: 0.288 g (87%); mp: 245–247 °C; IR (KBr) (*ν*_max_/cm^−1^): 3387, 3226, 2984, 2923, 2215, 1686, 1651, 1585, 1473, 1207, 1092, 839, 618; ^1^H NMR (300 MHz, DMSO): *δ* 0.57 (t, *J* = 7.2 Hz, 3H, CH_3_), 3.73 (q, *J* = 7.2 Hz, 2H, CH_2_), 5.67 (s, 2H, N–NH_2_), 7.25 (d, *J* = 8.4 Hz, 2H, ArH), 7.49 (d, *J* = 8.4 Hz, 2H, ArH), 8.42 (brs, 1H, NH_2_), 8.72 (brs, 1H, NH_2_); ^13^C{^1^H} NMR (75.4 MHz, DMSO): *δ* 13.2 (CH_3_), 60.5 (O–CH_2_), 88.1 (*C*–CN), 91.4 (*C*–CO_2_Et), 117 (CN), 128.4, 129.2, 133.4, 138.1 (Ar), 156.6 (C-4), 158.6 (C–NH_2_), 159.2 (C=O), 166.1 (CO_2_Et); MS (EI, 70 eV): *m*/*z* (%) = 334 (35) [M + 2]^+^, 333 (21) [M + 1]^+^, 332 (100) [M]^+^, 303 (5), 286 (13), 251 (13), 229 (21), 187 (5), 162 (12), 138 (7), 111 (4), 75 (4), 58 (3), 43 (5); anal. calcd for C_15_H_13_ClN_4_O_3_: C, 54.14; H, 3.94; N, 16.84. Found: C, 53.86; H, 3.74; N, 16.47.

#### Ethyl 1,6-diamino-4-(4-bromophenyl)-3-cyano-2-oxo-1,2-dihydropyridine-5-carboxylate (5b)

White solid; yield: 0.319 g (85%); mp: 245–247 °C; IR (KBr) (*ν*_max_/cm^−1^): 3391, 3270, 2216, 1686, 1588, 1537, 1471, 1207, 1091, 878, 768, 615; ^1^H NMR (300 MHz, DMSO): *δ* 0.57 (t, *J* = 6.9 Hz, 3H, CH_3_), 3.73 (q, *J* = 6.9 Hz, 2H, CH_2_), 5.66 (s, 2H, N–NH_2_), 7.19 (d, *J* = 8.1 Hz, 2H, ArH), 7.62 (d, *J* = 8.1 Hz, 2H, ArH), 8.42 (brs, 1H, NH_2_), 8.72 (brs, 1H, NH_2_); ^13^C{^1^H} NMR (75.4 MHz, DMSO): *δ* 13.1 (CH_3_), 60.5 (O–CH_2_), 88 (*C*–CN), 91.3 (*C*–CO_2_Et), 117 (CN), 121.9, 129.5, 131.3, 138.5 (Ar), 156.6 (C-4), 158.6 (C–NH_2_), 159.2 (C=O), 166.1 (CO_2_Et); MS (EI, 70 eV): *m*/*z* (%) = 379 (19) [M+2]^+^, 378 (100) [M+1]^+^, 377 (21) [M]^+^, 376 (99), 349 (4), 332 (9), 304 (9), 275 (18), 223 (18), 194 (14), 152 (8), 127 (12), 100 (5), 88 (5), 43 (3); anal. calcd for C_15_H_13_BrN_4_O_3_: C, 47.76; H, 3.47; N, 14.85. Found: C, 47.43; H, 3.75; N, 14.53.

#### Ethyl 1,6-diamino-4-(2-chlorophenyl)-3-cyano-2-oxo-1,2-dihydropyridine-5-carboxylate (5c)

White solid; yield: 0.265 g (80%); mp: 322–324 °C; IR (KBr) (*ν*_max_/cm^−1^): 3386, 3269, 2214, 1653, 1584, 1456, 1312, 1213, 1006, 800, 621; ^1^H NMR (300 MHz, DMSO): *δ* 0.55 (t, *J* = 6.9 Hz, 3H, CH_3_), 3.73 (q, *J* = 6.9 Hz, 2H, CH_2_), 5.69 (s, 2H, N–NH_2_), 7.24–7.52 (m, 4H, ArH), 8.47 (brs, 1H, NH_2_), 9.00 (brs, 1H, NH_2_); ^13^C{^1^H} NMR (75.4 MHz, DMSO): *δ* 13.2 (CH_3_), 60.5 (O–CH_2_), 88.5 (*C*–CN), 91 (*C*–CO_2_Et), 116.4 (CN), 127.4, 129, 129.3, 130.2, 130.7, 138.4 (Ar), 157 (C-4), 159.2 (C–NH_2_), 159.2 (C=O), 165.8 (CO_2_Et); MS (EI, 70 eV): *m*/*z* (%) = 334 (6) [M + 2]^+^, 333 (3) [M + 1]^+^, 332 (17) [M]^+^, 297 (100), 269 (95), 253 (12), 224 (11), 174 (6), 138 (7), 113 (3), 99 (3), 75 (3), 58 (2), 43 (2); anal. calcd for C_15_H_13_ClN_4_O_3_: C, 54.14; H, 3.94; N, 16.84. Found: C, 56.05; H, 4.04; N, 16.71.

#### Ethyl 1,6-diamino-4-(3-fluorophenyl)-3-cyano-2-oxo-1,2-dihydropyridine-5-carboxylate (5d)

White solid; yield: 0.237 g (75%); mp: 218–220 °C; IR (KBr) (*ν*_max_/cm^−1^): 3390, 3268, 2217, 1687, 1652, 1537, 1456, 1310, 1208, 1096, 884; ^1^H NMR (300 MHz, DMSO): *δ* 0.57 (t, *J* = 7.2 Hz, 3H, CH_3_), 3.74 (q, *J* = 7.2 Hz, 2H, CH_2_), 5.68 (s, 2H, N–NH_2_), 7.05 (d, *J* = 7.8 Hz, 1H, ArH), 7.14 (d, *J* = 9.6 Hz, 1H, ArH), 7.25 (t, *J* = 8.4 Hz, 1H, ArH), 7.45 (q, *J* = 8.1 Hz, 1H, ArH), 8.43 (brs, 1H, NH_2_), 8.78 (brs, 1H, NH_2_); ^13^C{^1^H} NMR (75.4 MHz, DMSO): *δ* 13.2 (CH_3_), 60.4 (O–CH_2_), 88.2 (*C*–CN), 91.3 (*C*–CO_2_Et), 116.9 (CN), 114.5, 115.3, 123.6, 130.5, 141.4, 163.8 (Ar), 156.7 (C-4), 158.3 (C–NH_2_), 159.1 (C=O), 166.1 (CO_2_Et); MS (EI, 70 eV): *m*/*z* (%) = 317 (19) [M + 1]^+^, 316 (100) [M]^+^, 287 (4), 270 (30), 213 (26), 171 (6), 146 (13), 111 (2), 75 (3), 58 (2), 43 (2); anal. calcd for C_15_H_13_FN_4_O_3_: C, 56.96; H, 4.14; N, 17.71. Found: C, 56.42; H, 4.28; N, 17.34.

#### Ethyl 1,6-diamino-4-(4-fluorophenyl)-3-cyano-2-oxo-1,2-dihydropyridine-5-carboxylate (5e)

White solid; yield: 0.237 g (75%); mp: 237–239 °C; ^1^H NMR (300 MHz, DMSO): *δ* 0.59 (t, *J* = 7.2 Hz, 3H, CH_3_), 3.73 (q, *J* = 7.2 Hz, 2H, CH_2_), 5.66 (s, 2H, N–NH_2_), 7.26 (d, *J* = 7.8 Hz, 4H, ArH), 8.55 (br, 2H, NH_2_); ^13^C{^1^H} NMR (75.4 MHz, DMSO): *δ* 13.2 (CH_3_), 60.5 (O–CH_2_), 88.2 (*C*–CN), 91.7 (*C*–CO_2_Et), 117.1 (CN), 115.4, 129.5, 135.5, 163.2 (Ar), 156.5 (C-4), 158.9 (C–NH_2_), 159.3 (C=O), 166.2 (CO_2_Et); anal. calcd for C_15_H_13_FN_4_O_3_: C, 56.96; H, 4.14; N, 17.71. Found: C, 56.14; H, 4.08; N, 17.61.

### General procedure for the synthesis of 1,6-diamino-4-aryl-3-cyano-2-oxo-1,2-dihydropyridine-5-carboxamide (6a–e)

The stoichiometric mixture of cyanoacetohydrazide 1 (1 mmol, 0.99 g), cyanoacetamide 2c (1 mmol, 0.084 g), aromatic aldehyde (1 mmol) and piperidine (20% mol, 0.019 ml) in a mixture of H_2_O and ethanol (3 : 6 ml) was stirred at 80 °C. After completion of the reaction that was monitored by TLC (using ethyl acetate/*n*-hexane (1 : 1)), the precipitated product was collected by filtration and washed with H_2_O/EtOH to give the pure products 6a–e.

#### 1,6-Diamino-4-(4-bromophenyl)-3-cyano-2-oxo-1,2-dihydropyridine-5-carboxamide (6a)

Brown solid; yield: 0.278 g (80%); mp: 245–247 °C; IR (KBr) (*ν*_max_/cm^−1^): 3394, 3290, 2205, 1662, 1612, 1571, 1463, 1399, 1267, 886, 579; ^1^H NMR (300 MHz, DMSO): *δ* 5.67 (s, 2H, N–NH_2_), 6.85 (brs, 1H, NH_2_), 7.22 (brs, 1H, NH_2_), 7.28 (d, *J* = 8.4 Hz, 2H, ArH), 7.65 (d, *J* = 8.4 Hz, 2H, ArH), 7.78 (s, 2H, CONH_2_); ^13^C{^1^H} NMR (75.4 MHz, DMSO): *δ* 83.7 (*C*–CN), 99.4 (*C*–CONH_2_), 118.1 (CN), 123, 130.8, 131.6, 136.1 (Ar), 153.8 (C-4), 154 (C–NH_2_), 159.6 (C=O), 167.3 (CONH_2_); MS (EI, 70 eV): *m*/*z* (%) = 350 (16) [M + 2]^+^, 349 (99) [M + 1]^+^, 348 (64) [M]^+^, 347 (100), 346 (51), 332 (5), 301 (5), 275 (4), 251 (43), 237 (8), 205 (16), 194 (24), 180 (9), 152 (9), 140 (18), 113 (8), 100 (9), 88 (8), 63 (9), 44 (22); anal. calcd for C_13_H_10_BrN_5_O_2_: C, 44.85; H, 2.90; N, 20.12. Found: C, 41.97; H, 3.38; N, 17.42.

#### 1,6-Diamino-4-(4-chlorophenyl)-3-cyano-2-oxo-1,2-dihydropyridine-5-carboxamide (6b)

Pale brown solid; yield: 0.236 g (78%); mp: 280–282 °C; IR (KBr) (*ν*_max_/cm^−1^): 3386, 3301, 2208, 1635, 1574, 1461, 1398, 1268, 880, 585; ^1^H NMR (300 MHz, DMSO): *δ* 5.67 (s, 2H, N–NH_2_), 6.83 (brs, 1H, NH_2_), 7.21 (brs, 1H, NH_2_), 7.35 (d, *J* = 8.4 Hz, 2H, ArH), 7.51 (d, *J* = 8.4 Hz, 2H, ArH), 7.78 (brs, 2H, CONH_2_); ^13^C{^1^H} NMR (75.4 MHz, DMSO): *δ* 83.8 (*C*–CN), 99.4 (*C*–CONH_2_), 118.1 (CN), 128.7, 130.5, 134.3, 135.7 (Ar), 153.8 (C-4), 154 (C–NH_2_), 159.6 (C=O), 167.3 (CONH_2_); anal. calcd for C_13_H_10_ClN_5_O_2_: C, 51.41; H, 3.32; N, 23.06. Found: C, 51.87; H, 3.54; N, 22.57.

#### 1,6-Diamino-4-(3-chlorophenyl)-3-cyano-2-oxo-1,2-dihydropyridine-5-carboxamide (6c)

White solid; yield: 0.227 g (75%); mp: 322–324 °C; ^1^H NMR (300 MHz, DMSO): *δ* 5.67 (s, 2H, N–NH_2_), 6.91 (brs, 1H, NH_2_), 7.26 (brs, 1H, NH_2_), 7.28–7.48 (m, 4H, ArH), 7.79 (s, 2H, CONH_2_); ^13^C{^1^H} NMR (75.4 MHz, DMSO): *δ* 83.7 (*C*–CN), 99.5 (*C*–CONH_2_), 118 (CN), 127.4, 128.4, 129.4, 130.5, 133.1, 138.8 (Ar), 153.3 (C-4), 153.9 (C–NH_2_), 159.6 (C=O), 167.2 (CONH_2_); anal. calcd for C_13_H_10_ClN_5_O_2_: C, 51.41; H, 3.32; N, 23.06.

#### 1,6-Diamino-4-(2-chlorophenyl)-3-cyano-2-oxo-1,2-dihydropyridine-5-carboxamide (6d)

Pale brown solid; yield: 0.212 g (70%); mp: 218–220 °C; ^1^H NMR (300 MHz, DMSO): *δ* 5.69 (s, 2H, N–NH_2_), 6.37 (br, 1H, NH_2_), 7.27 (br, 1H, NH_2_), 7.42–7.54 (m, 4H, ArH), 8.05 (s, 2H, CONH_2_); ^13^C{^1^H} NMR (75.4 MHz, DMSO): *δ* 84.7 (*C*–CN), 99.2 (*C*–CONH_2_), 117.4 (CN), 127.4, 130, 130.6, 131.1, 131.4, 135.8 (Ar), 152.4 (C-4), 154.6 (C–NH_2_), 159.4 (C=O), 166.8 (CONH_2_); anal. calcd for C_13_H_10_ClN_5_O_2_: C, 51.41; H, 3.32; N, 23.06.

#### 1,6-Diamino-4-(4-fluorophenyl)-3-cyano-2-oxo-1,2-dihydropyridine-5-carboxamide (6e)

Pale brown solid; yield: 0.186 g (65%); mp: 237–239 °C; ^1^H NMR (300 MHz, DMSO): *δ* 5.66 (s, 2H, N–NH_2_), 6.77 (brs, 1H, NH_2_), 7.21 (brs, 1H, NH_2_), 7.25–7.37 (m, 4H, ArH), 7.77 (s, 2H, CONH_2_); ^13^C{^1^H} NMR (75.4 MHz, DMSO): *δ* 84.1 (*C*–CN), 99.5 (*C*–CONH_2_), 118.1 (CN), 115.6, 130.9, 131, 133.2 (Ar), 154 (C-4), 154.1 (C–NH_2_), 159.7 (C=O), 167.4 (CONH_2_); MS (EI, 70 eV): *m*/*z* (%) = 288 (9) [M + 1]^+^, 287 (51) [M]^+^, 286 (21), 270 (17), 241 (10), 205 (26), 177 (12), 146 (18), 122 (100), 109 (24), 95 (25), 75 (13), 57 (11), 44 (16); anal. calcd for C_13_H_10_FN_5_O_2_: C, 54.36; H, 3.51; N, 24.38. Found: C, 56.09; H, 4.15; N, 19.43.

## Conclusion

We have reported a novel and green one-pot synthesis of three classes of polysubstituted pyridine systems, 1,6-diamino-4-aryl-3,5-dicyano-2-pyridone, ethyl 1,6-diamino-4-aryl-3-cyano-2-pyridone-5-carboxylate and 1,6-diamino-4-aryl-3-cyano-2-pyridone-5-carboxamide derivatives, *via* a three-component reaction between cyanoacetohydrazide, activated nitrile compounds and aromatic aldehydes. The present process includes some important advantages like easy operation mild reaction conditions, facile accessibility of reactants, simple workup procedure, the use of water or water/ethanol as green reaction medium, high atom economy and good to high yields.

## Conflicts of interest

The authors declare no competing financial interest.

## Supplementary Material

RA-008-C8RA05690K-s001
