# Supplementary material for: An efficient and ecofriendly synthesis of highly functionalized pyridones via a one-pot three-component reaction
Source: RSC Adv. 2018 Jul 30;8(48):27131–43. doi: 10.1039/c8ra05690k (PMC9092425; doi:10.1039/c8ra05690k)
Supplement: RA-008-C8RA05690K-s001 [file RA-008-C8RA05690K-s001.pdf]

Electronic Supplementary Material (ESI) for RSC Advances.
This journal is © The Royal Society of Chemistry 2018

Supporting Information

An Efficient and Ecofriendly Synthesis of Highly Functionalized Pyridones via a One-pot Three-Component Reaction

Hajar Hosseini, Mohammad Bayat*

Department of Chemistry, Faculty of Science, Imam Khomeini International University, Qazvin, Iran

bayat_mo@yahoo.com

The Table of Contents

Title	Page
Title, author's name, address and table of contents	1-2
Experimental Section; General remarks	2
**Figure 1.** Structure of all products **4a-l, 5a-e, 6a-e**	3
^{1}H and ^{13}C NMR and IR and Mass spectrums of **4a**	4-7
^{1}H and ^{13}C NMR and IR and Mass spectrums of **4b**	8-11
^{1}H and D_2O exchange and ^{13}C NMR and Mass spectrums of **4c**	12-14
^{1}H and D_2O exchange and ^{13}C NMR and IR and Mass spectrums of **4d**	15-18
^{1}H and ^{13}C NMR and IR and Mass spectrums of **4e**	19-22
^{1}H and ^{13}C NMR and IR and Mass spectrums of **4f**	23-26
^{1}H and ^{13}C NMR and IR and Mass spectrums of **4g**	27-30
^{1}H and ^{13}C NMR spectrums of **4h**	31-32
^{1}H and ^{13}C NMR and Mass spectrums of **4i**	33-35
^{1}H and ^{13}C NMR and IR and Mass spectrums of **4j**	36-39
^{1}H and ^{13}C NMR and IR spectrums of **4k**	40-42

^{1}H and ^{13}C NMR and IR spectrums of **4l**	43-45
^{1}H and D_2O exchange and ^{13}C NMR and IR and Mass spectrums of **5a**	46-49
^{1}H and ^{13}C NMR and IR and Mass spectrums of **5b**	50-53
^{1}H and ^{13}C NMR and IR and Mass spectrums of **5c**	54-57
^{1}H and ^{13}C NMR and IR and Mass spectrums of **5d**	58-61
^{1}H and ^{13}C NMR spectrums of **5e**	62-63
^{1}H and ^{13}C NMR and IR and Mass spectrums of **6a**	64-67
^{1}H and ^{13}C NMR and IR spectrums of **6b**	68-70
^{1}H and ^{13}C NMR spectrums of **6c**	71-72
^{1}H and ^{13}C NMR spectrums of **6d**	73-74
^{1}H and ^{13}C NMR and Mass spectrums of **6e**	75-77

Experimental Section

General remarks:

All commercially available reagents and other solvents were purchased and used without further purification. The NMR spectra were recorded with a Bruker DRX-300 Avance instrument instrument (300 MHz for ^{1}H and 75.4 MHz for ^{13}C) with DMSO-d_6 as solvent. Chemical shifts are given in ppm (δ), and coupling constant (*J*) are reported in hertz (Hz). Melting points were measured with an electrotherma1 9100 apparatus. Mass spectra were recorded with an Agilent 5975C VL MSD with Triple-Axis Detector operating at an ionization potential of 70 eV. IR spectra were measured with, Bruker Tensor 27 spectrometer ($\bar{\upsilon}$ in cm^{-1}). Elemental analyses for C, H and N were performed using a PerkinElmer 2004 series [II] CHN elemental analyzer.. All NMR spectra at room temperature were determined in DMSO-d_6. Chemical shifts are reported in parts per million (*δ*). Coupling constants (*J* values) are reported in hertz (Hz), and spin multiplicities are indicated by the following symbols: s (singlet), d (doublet), t (triplet), q (quartet), m (multiplet). All chemicals were purchased and were used without further purification.

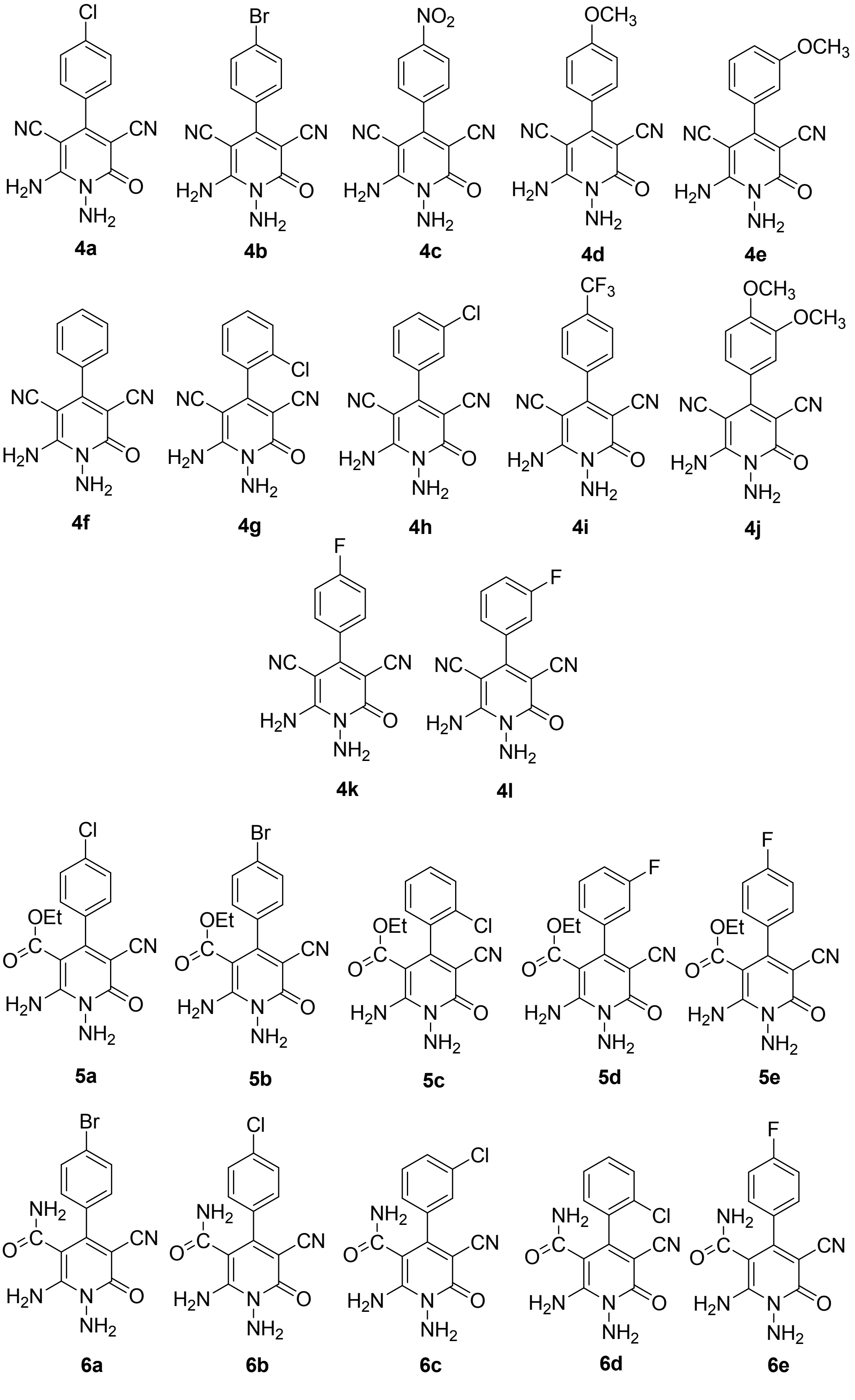

Figure 1. Structure of all products **4a-l, 5a-e, 6a-e.**

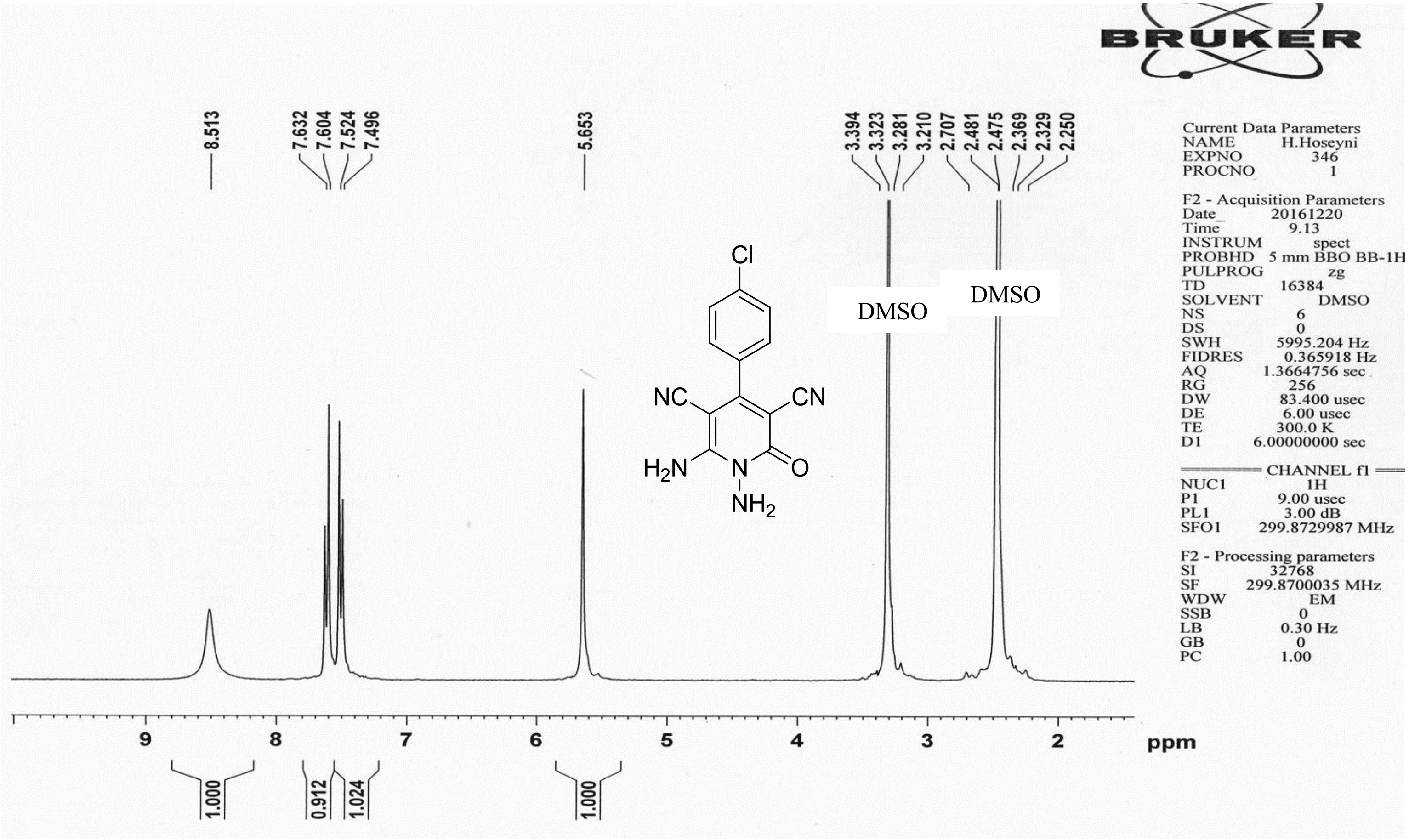
BRUKER
8.513
7.632
7.604
7.524
7.496
5.653
3.394
3.323
3.281
3.210
2.707
2.481
2.475
2.369
2.329
2.250
DMSO
DMSO
Cl
NC
CN
H2N
N
O
NH2
Current Data Parameters
NAME H.Hoseyni
EXPNO 346
PROCNO 1
F2 - Acquisition Parameters
Date_ 20161220
Time 9.13
INSTRUM spect
PROBHD 5 mm BBO BB-1H
PULPROG zg
TD 16384
SOLVENT DMSO
NS 6
DS 0
SWH 5995.204 Hz
FIDRES 0.365918 Hz
AQ 1.3664756 sec
RG 256
DW 83.400 usec
DE 6.00 usec
TE 300.0 K
D1 6.00000000 sec
CHANNEL f1
NUC1 1H
P1 9.00 usec
PL1 3.00 dB
SFO1 299.8729987 MHz
F2 - Processing parameters
SI 32768
SF 299.8700035 MHz
WDW EM
SSB 0
LB 0.30 Hz
GB 0
PC 1.00
9
8
7
6
5
4
3
2
ppm
1.000
0.912
1.024
1.000

^{1}H NMR of 4a

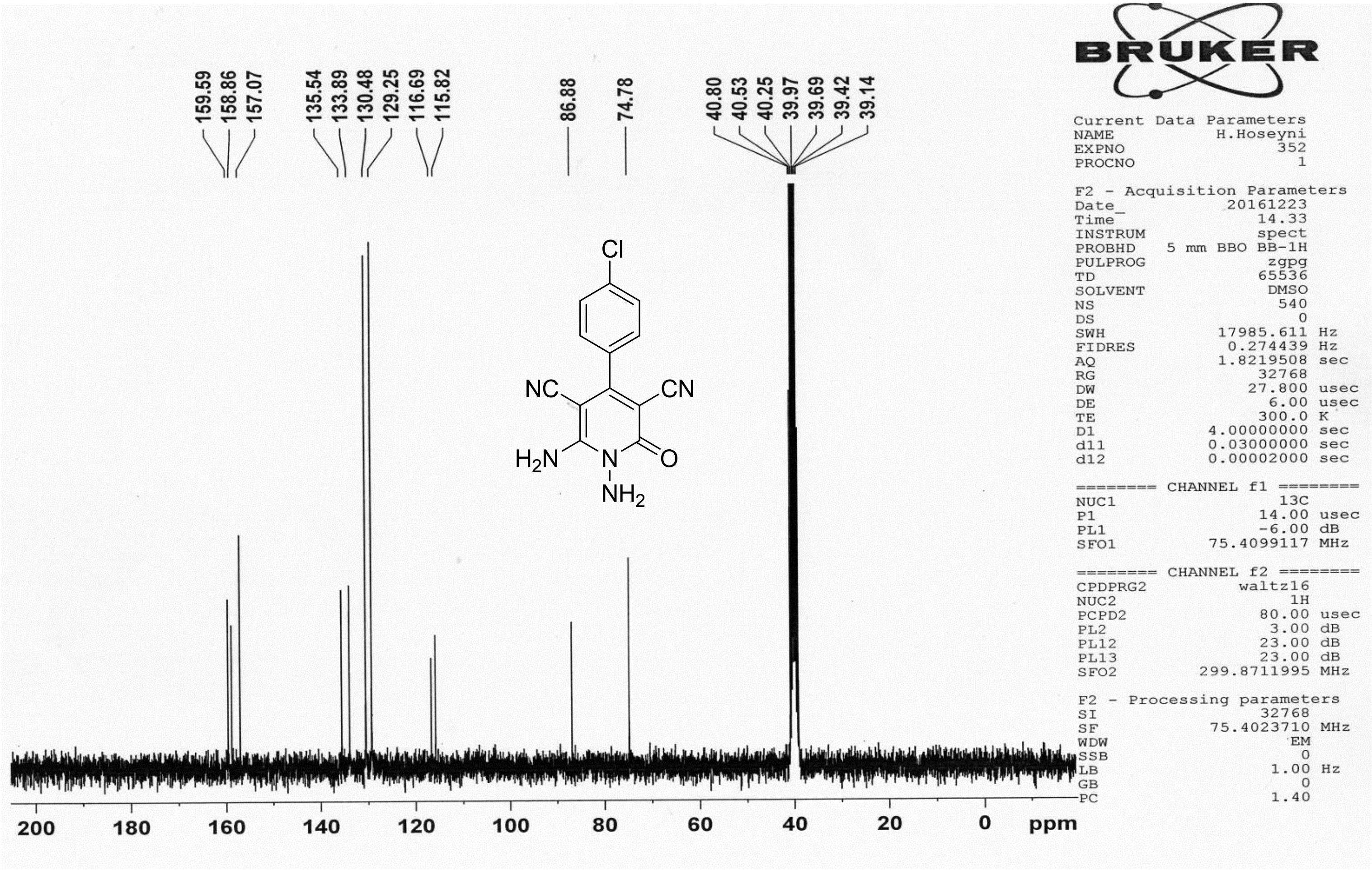

159.59
158.86
157.07
135.54
133.89
130.48
129.25
116.69
115.82
86.88
74.78
40.80
40.53
40.25
39.97
39.69
39.42
39.14
BRUKER
Current Data Parameters
NAME H.Hoseyni
EXPNO 352
PROCNO 1
F2 - Acquisition Parameters
Date_ 20161223
Time 14.33
INSTRUM spect
PROBHD 5 mm BBO BB-1H
PULPROG zgpg
TD 65536
SOLVENT DMSO
NS 540
DS 0
SWH 17985.611 Hz
FIDRES 0.274439 Hz
AQ 1.8219508 sec
RG 32768
DW 27.800 usec
DE 6.00 usec
TE 300.0 K
D1 4.00000000 sec
d11 0.03000000 sec
d12 0.00002000 sec
======== CHANNEL f1 ========
NUC1 13C
P1 14.00 usec
PL1 -6.00 dB
SFO1 75.4099117 MHz
======== CHANNEL f2 ========
CPDPRG2 waltz16
NUC2 1H
PCPD2 80.00 usec
PL2 3.00 dB
PL12 23.00 dB
PL13 23.00 dB
SFO2 299.8711995 MHz
F2 - Processing parameters
SI 32768
SF 75.4023710 MHz
WDW EM
SSB 0
LB 1.00 Hz
GB 0
PC 1.40
Cl
NC
CN
H2N
N
O
NH2
200 180 160 140 120 100 80 60 40 20 0 ppm

^{13}C NMR of 4a

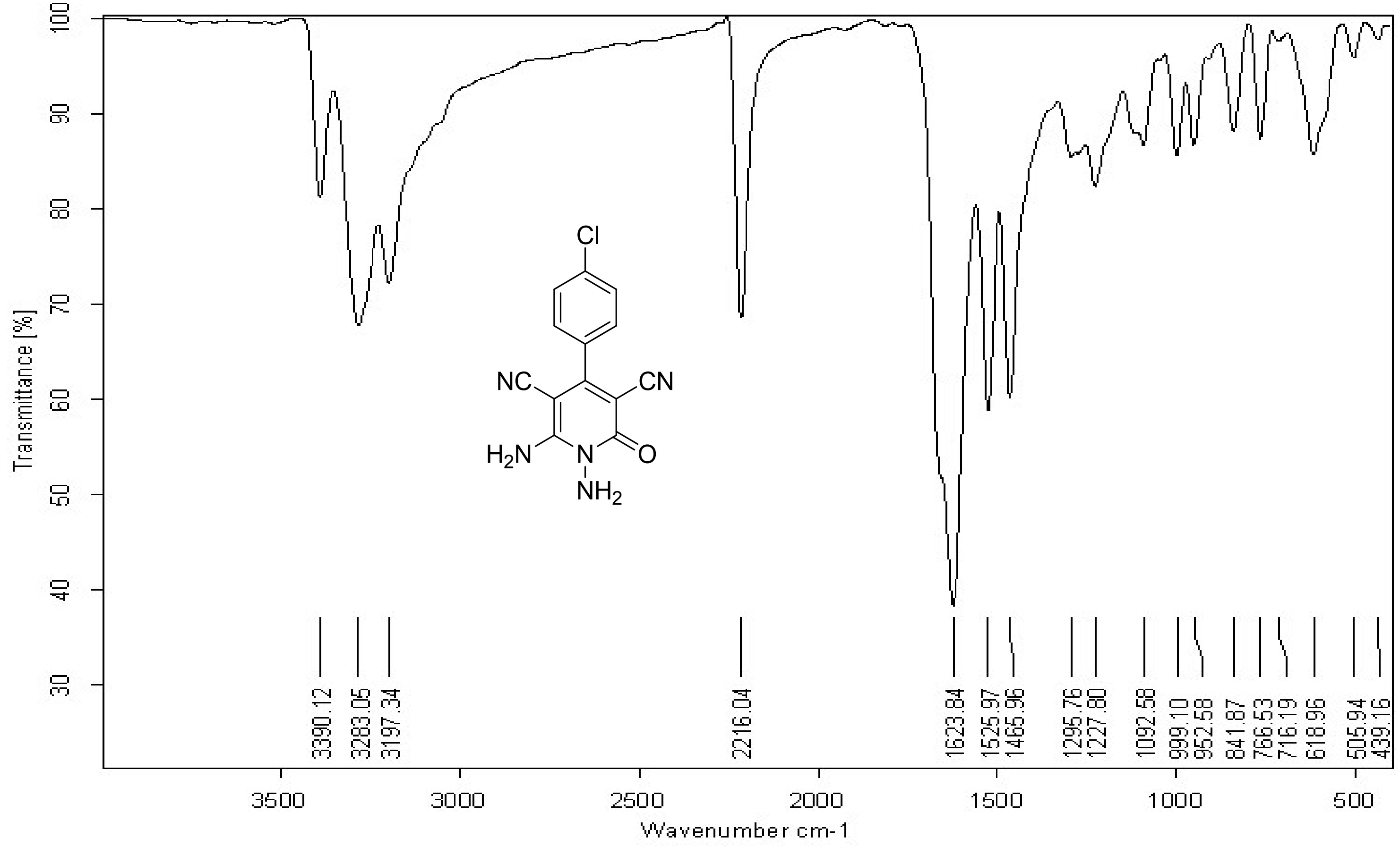

Transmittance [%]
Wavenumber cm-1
Cl
NC
CN
H_2N
N
O
NH_2
3390.12
3283.05
3197.34
2216.04
1623.84
1525.97
1465.96
1295.76
1227.80
1092.58
999.10
952.58
841.87
766.53
716.19
618.96
505.94
439.16

IR of 4a

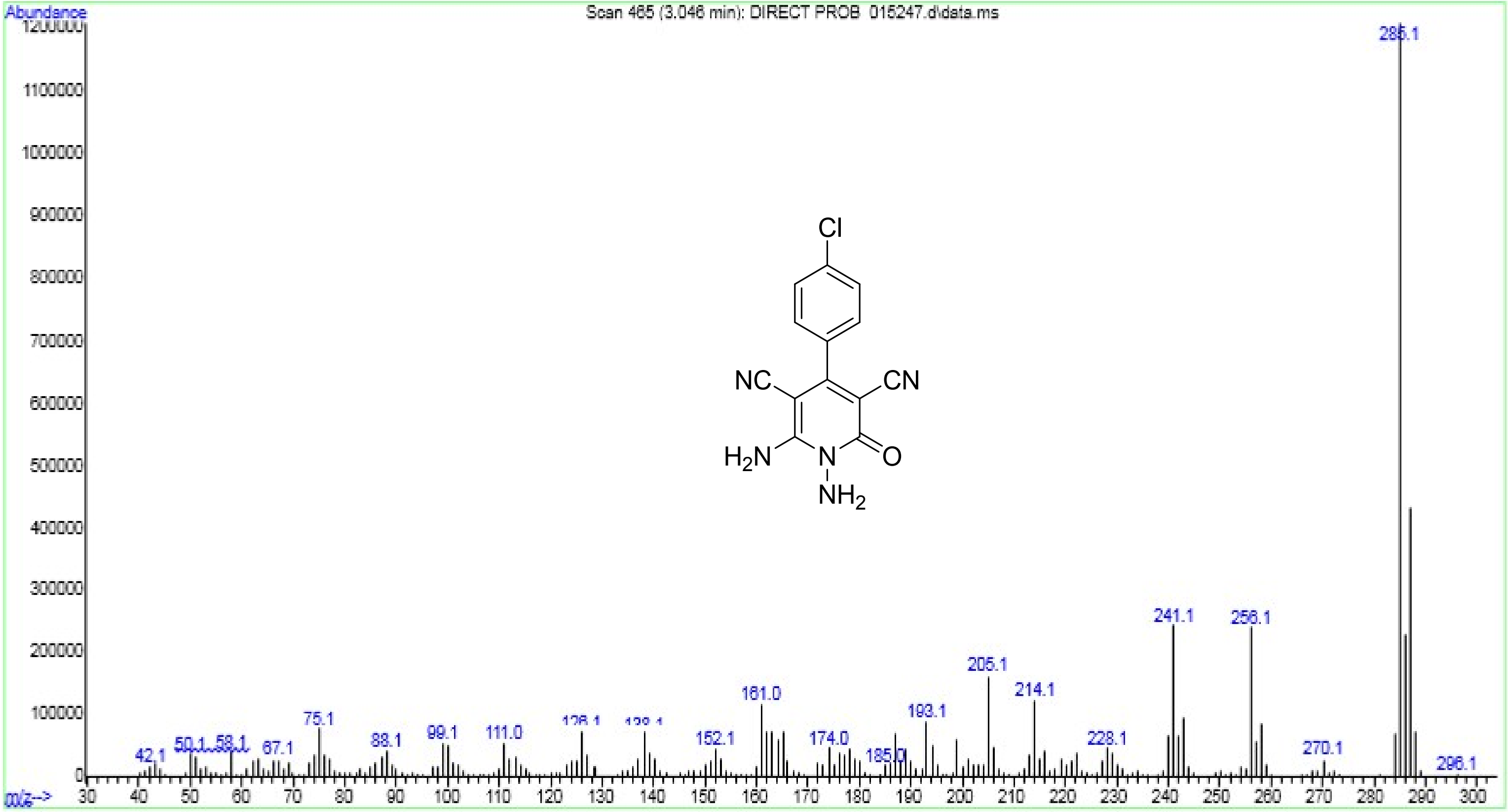
Abundance
Scan 465 (3.046 min): DIRECT PROB 015247.d\data.ms
285.1
241.1
256.1
205.1
214.1
161.0
193.1
174.0
152.1
228.1
270.1
296.1
185.0
111.0
99.1
88.1
75.1
67.1
58.1
50.1
42.1
m/z-->
Cl
NC
CN
H_2N
N
O
NH_2

MS of 4a

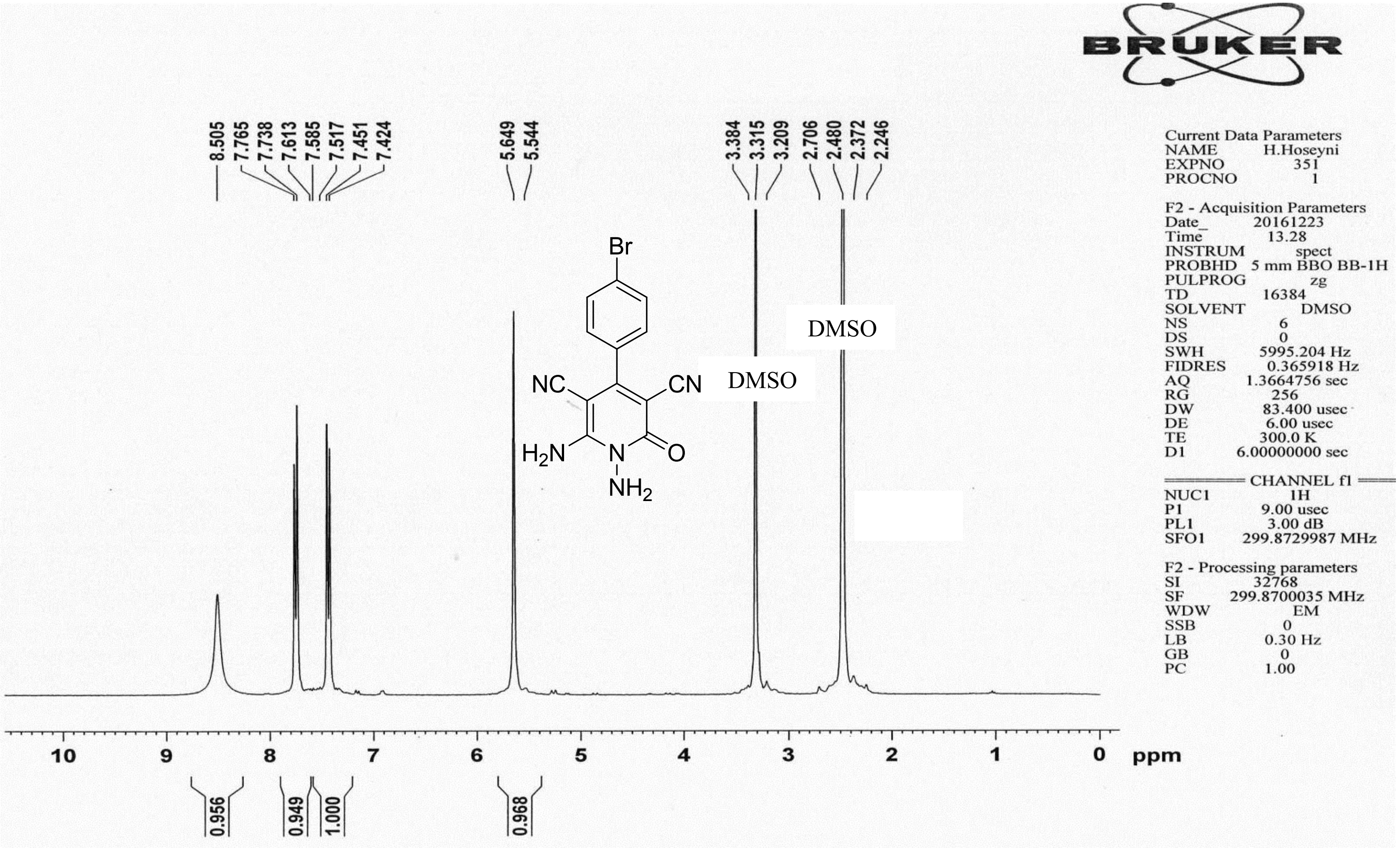
BRUKER
8.505
7.765
7.738
7.613
7.585
7.517
7.451
7.424
5.649
5.544
3.384
3.315
3.209
2.706
2.480
2.372
2.246
Br
NC
CN
H2N
N
O
NH2
DMSO
DMSO
Current Data Parameters
NAME H.Hoseyni
EXPNO 351
PROCNO 1
F2 - Acquisition Parameters
Date_ 20161223
Time 13.28
INSTRUM spect
PROBHD 5 mm BBO BB-1H
PULPROG zg
TD 16384
SOLVENT DMSO
NS 6
DS 0
SWH 5995.204 Hz
FIDRES 0.365918 Hz
AQ 1.3664756 sec
RG 256
DW 83.400 usec
DE 6.00 usec
TE 300.0 K
D1 6.00000000 sec
CHANNEL f1
NUC1 1H
P1 9.00 usec
PL1 3.00 dB
SFO1 299.8729987 MHz
F2 - Processing parameters
SI 32768
SF 299.8700035 MHz
WDW EM
SSB 0
LB 0.30 Hz
GB 0
PC 1.00
10
9
8
7
6
5
4
3
2
1
0
ppm
0.956
0.949
1.000
0.968

^{1}H NMR of 4b

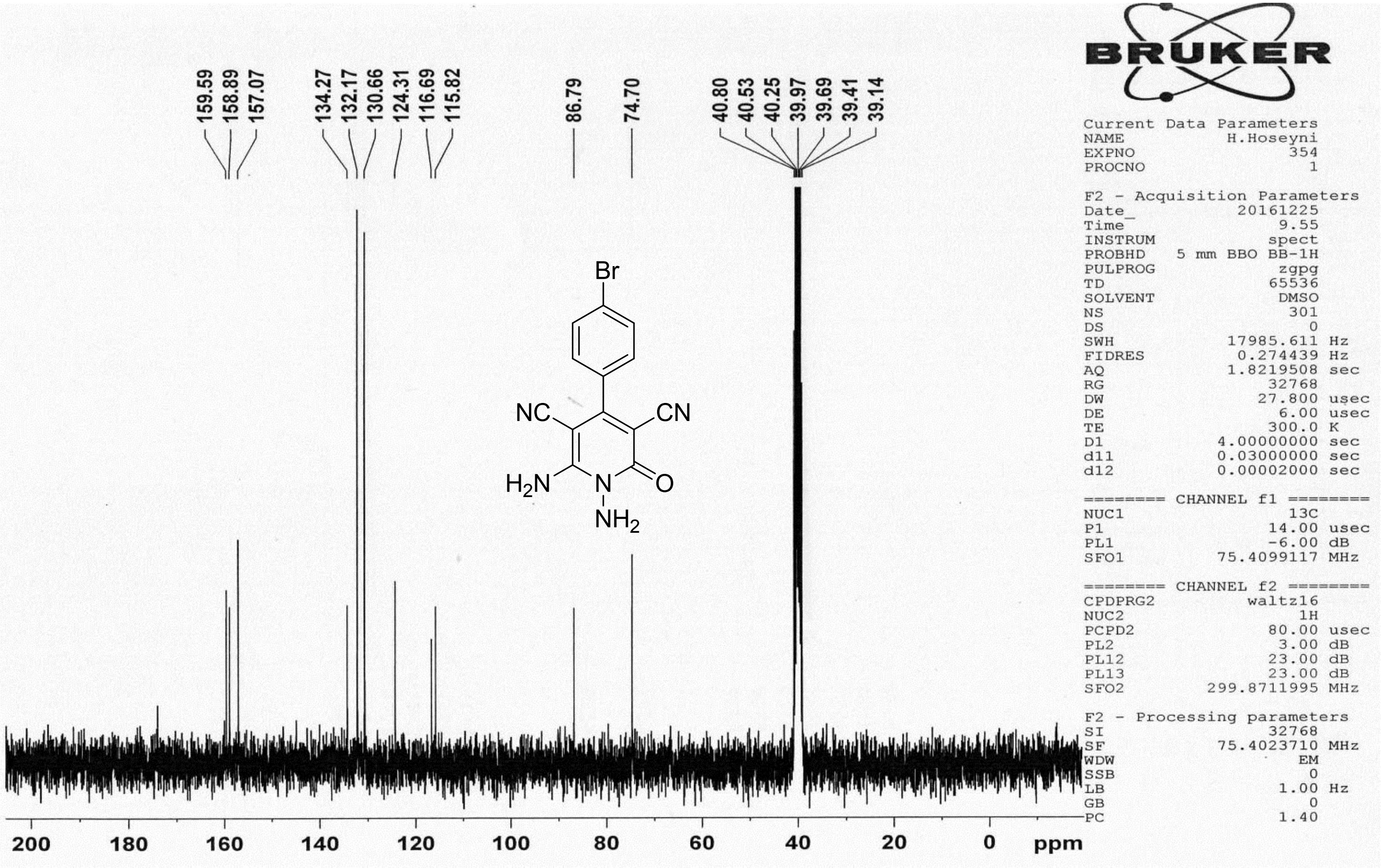
159.59
158.89
157.07
134.27
132.17
130.66
124.31
116.69
115.82
86.79
74.70
40.80
40.53
40.25
39.97
39.69
39.41
39.14
BRUKER
Current Data Parameters
NAME H.Hoseyni
EXPNO 354
PROCNO 1
F2 - Acquisition Parameters
Date_ 20161225
Time 9.55
INSTRUM spect
PROBHD 5 mm BBO BB-1H
PULPROG zgpg
TD 65536
SOLVENT DMSO
NS 301
DS 0
SWH 17985.611 Hz
FIDRES 0.274439 Hz
AQ 1.8219508 sec
RG 32768
DW 27.800 usec
DE 6.00 usec
TE 300.0 K
D1 4.00000000 sec
d11 0.03000000 sec
d12 0.00002000 sec
======== CHANNEL f1 ========
NUC1 13C
P1 14.00 usec
PL1 -6.00 dB
SFO1 75.4099117 MHz
======== CHANNEL f2 ========
CPDPRG2 waltz16
NUC2 1H
PCPD2 80.00 usec
PL2 3.00 dB
PL12 23.00 dB
PL13 23.00 dB
SFO2 299.8711995 MHz
F2 - Processing parameters
SI 32768
SF 75.4023710 MHz
WDW EM
SSB 0
LB 1.00 Hz
GB 0
PC 1.40
Br
NC
CN
H2N
N
O
NH2
200
180
160
140
120
100
80
60
40
20
0
ppm

^{13}C NMR of 4b

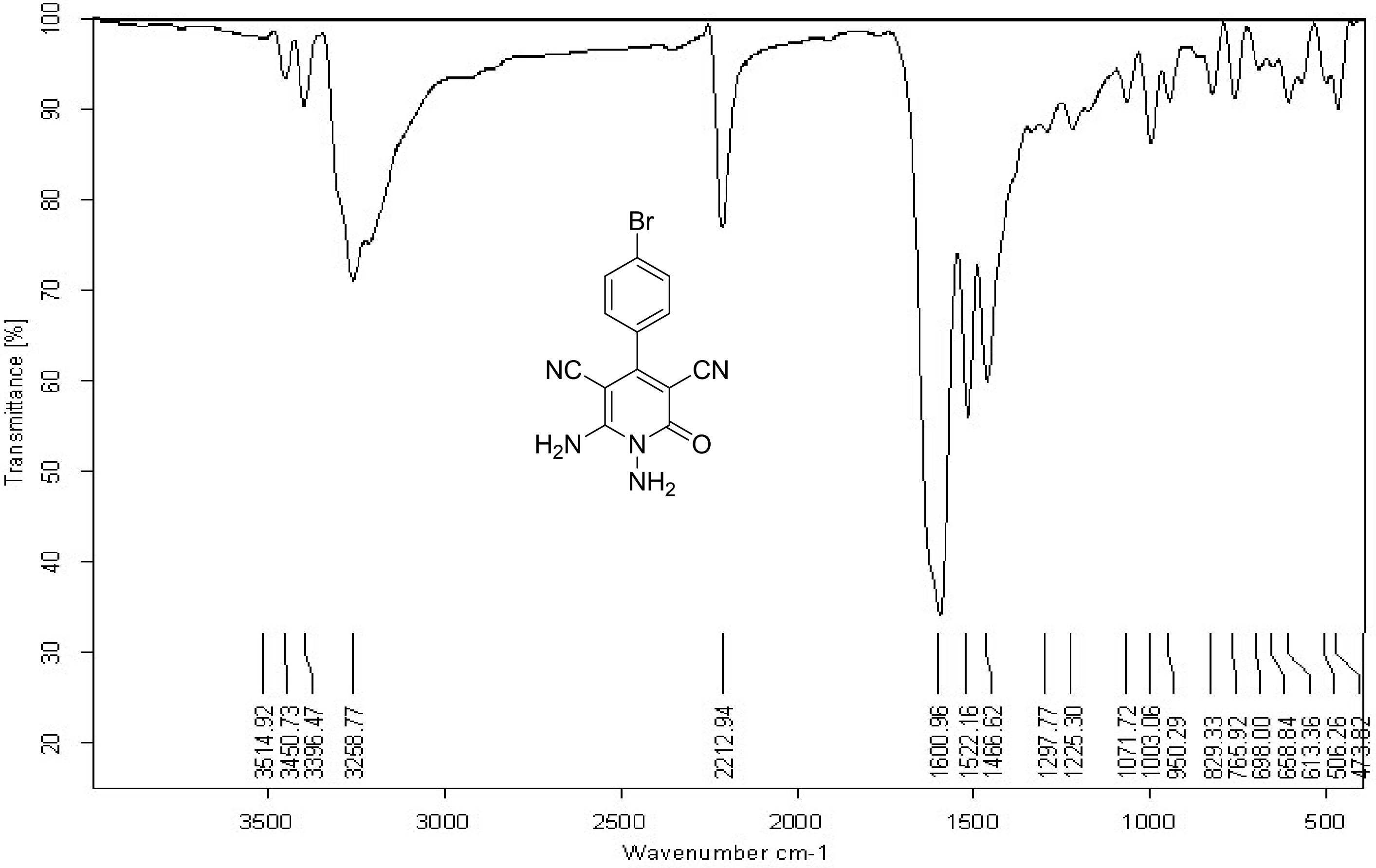
Transmittance [%]
Wavenumber cm-1
Br
NC
CN
H2N
N
O
NH2
3514.92
3450.73
3396.47
3258.77
2212.94
1600.96
1522.16
1466.62
1297.77
1225.30
1071.72
1003.06
950.29
829.33
765.92
698.00
658.84
613.36
506.26
473.82

IR of 4b

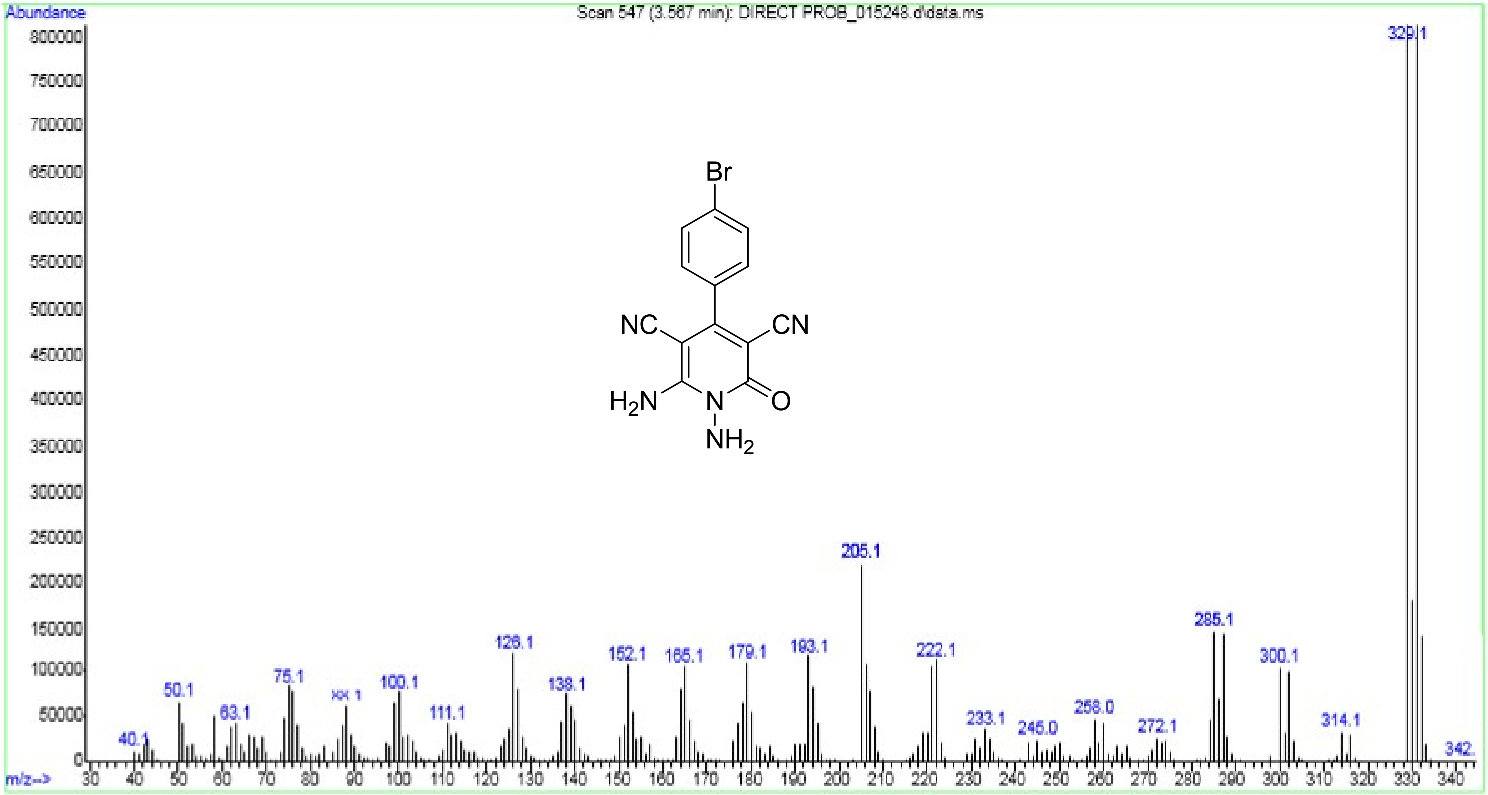
Abundance
Scan 547 (3.567 min): DIRECT PROB_015248.d\data.ms
m/z-->
Br
NC
CN
H2N
N
O
NH2
329.1
205.1
285.1
300.1
314.1
342.
126.1
152.1
165.1
179.1
193.1
222.1
233.1
245.0
258.0
272.1
138.1
100.1
111.1
75.1
63.1
50.1
40.1

MS of 4b

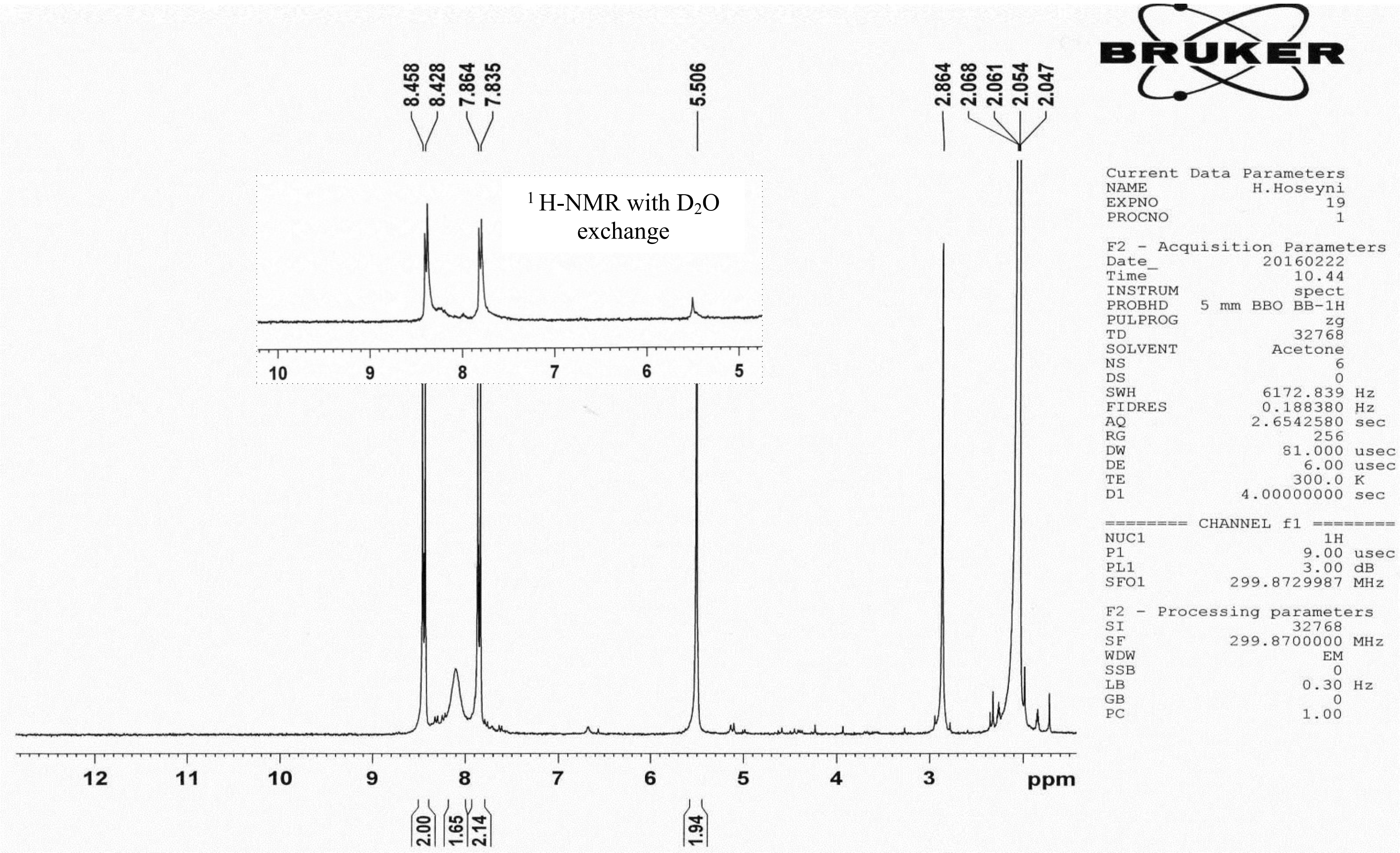
BRUKER
8.458
8.428
7.864
7.835
5.506
2.864
2.068
2.061
2.054
2.047
1 H-NMR with D2O exchange
10 9 8 7 6 5
12 11 10 9 8 7 6 5 4 3 ppm
2.00
1.65
2.14
1.94
Current Data Parameters
NAME H.Hoseyni
EXPNO 19
PROCNO 1
F2 - Acquisition Parameters
Date_ 20160222
Time 10.44
INSTRUM spect
PROBHD 5 mm BBO BB-1H
PULPROG zg
TD 32768
SOLVENT Acetone
NS 6
DS 0
SWH 6172.839 Hz
FIDRES 0.188380 Hz
AQ 2.6542580 sec
RG 256
DW 81.000 usec
DE 6.00 usec
TE 300.0 K
D1 4.00000000 sec
======== CHANNEL f1 ========
NUC1 1H
P1 9.00 usec
PL1 3.00 dB
SFO1 299.8729987 MHz
F2 - Processing parameters
SI 32768
SF 299.8700000 MHz
WDW EM
SSB 0
LB 0.30 Hz
GB 0
PC 1.00

^{1}H NMR of 4c

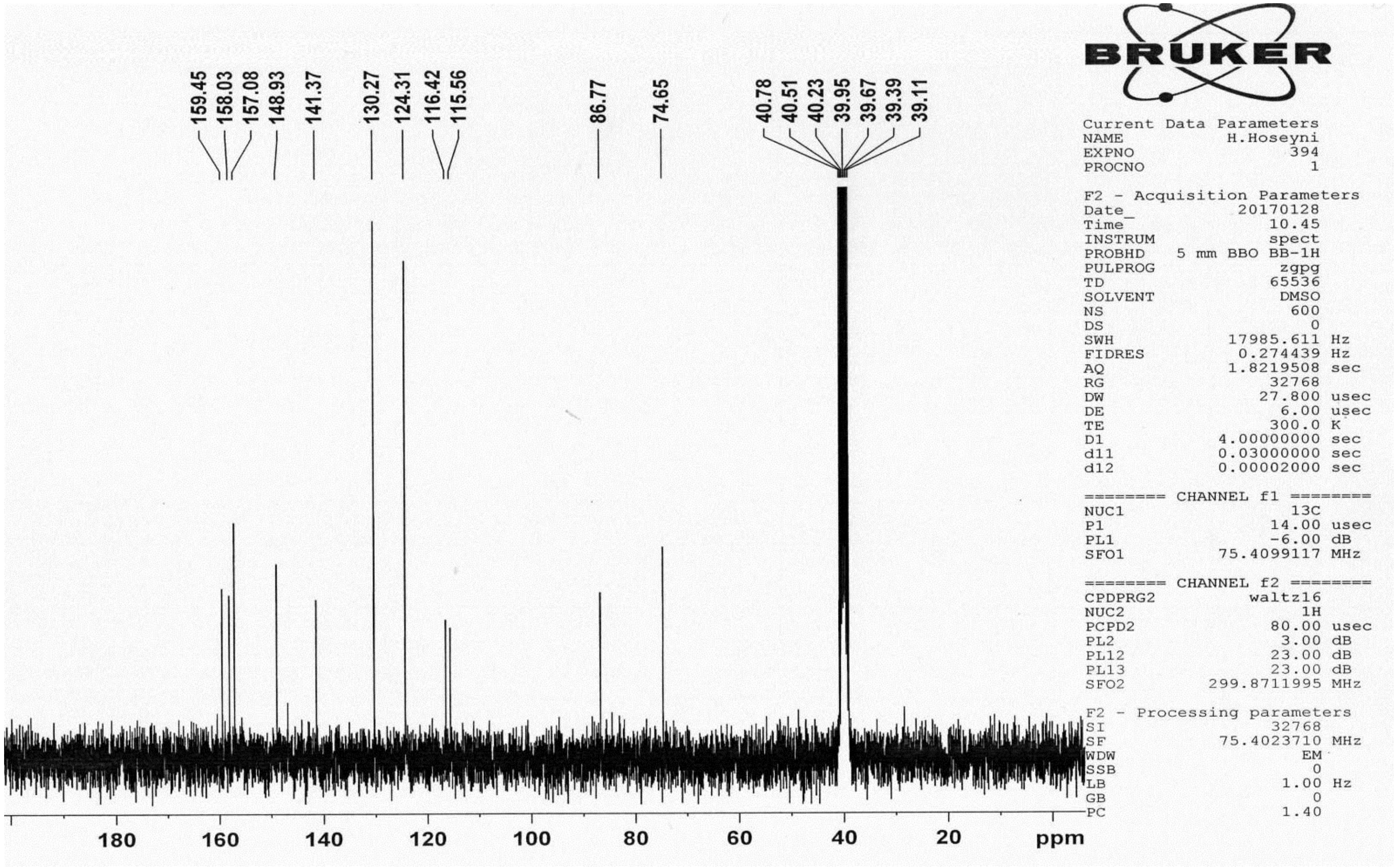
159.45
158.03
157.08
148.93
141.37
130.27
124.31
116.42
115.56
86.77
74.65
40.78
40.51
40.23
39.95
39.67
39.39
39.11
BRUKER
Current Data Parameters
NAME H.Hoseyni
EXPNO 394
PROCNO 1
F2 - Acquisition Parameters
Date_ 20170128
Time 10.45
INSTRUM spect
PROBHD 5 mm BBO BB-1H
PULPROG zgpg
TD 65536
SOLVENT DMSO
NS 600
DS 0
SWH 17985.611 Hz
FIDRES 0.274439 Hz
AQ 1.8219508 sec
RG 32768
DW 27.800 usec
DE 6.00 usec
TE 300.0 K
D1 4.00000000 sec
d11 0.03000000 sec
d12 0.00002000 sec
======== CHANNEL f1 ========
NUC1 13C
P1 14.00 usec
PL1 -6.00 dB
SFO1 75.4099117 MHz
======== CHANNEL f2 ========
CPDPRG2 waltz16
NUC2 1H
PCPD2 80.00 usec
PL2 3.00 dB
PL12 23.00 dB
PL13 23.00 dB
SFO2 299.8711995 MHz
F2 - Processing parameters
SI 32768
SF 75.4023710 MHz
WDW EM
SSB 0
LB 1.00 Hz
GB 0
PC 1.40
180
160
140
120
100
80
60
40
20
ppm

^{13}C NMR of 4c

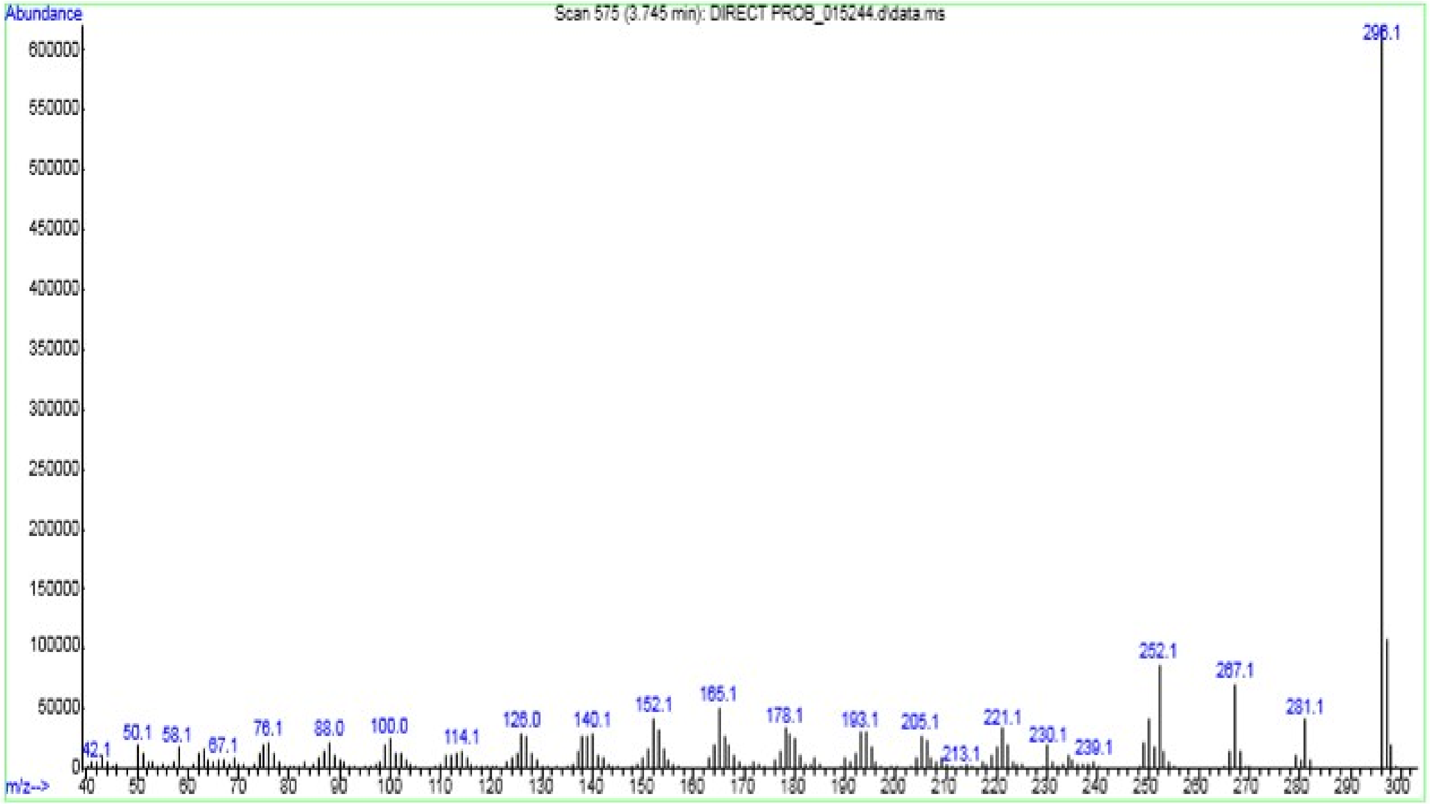
Abundance
Scan 575 (3.745 min): DIRECT PROB_015244.d\data.ms
m/z-->
296.1
252.1
267.1
281.1
165.1
152.1
178.1
193.1
205.1
221.1
230.1
239.1
213.1
126.0
140.1
114.1
100.0
88.0
76.1
67.1
58.1
50.1
42.1

MS of 4c

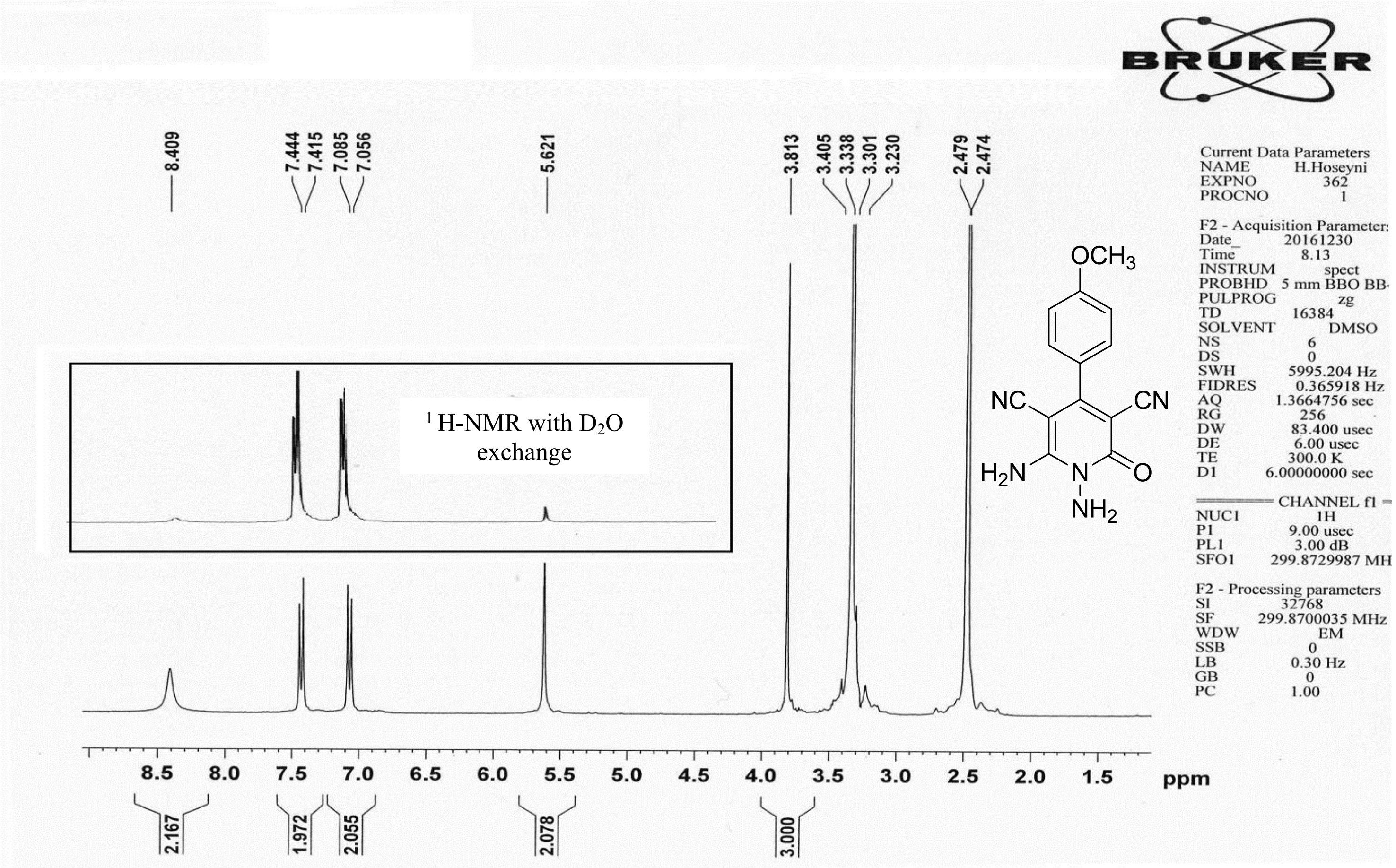
BRUKER
8.409
7.444
7.415
7.085
7.056
5.621
3.813
3.405
3.338
3.301
3.230
2.479
2.474
Current Data Parameters
NAME H.Hoseyni
EXPNO 362
PROCNO 1
F2 - Acquisition Parameters
Date_ 20161230
Time 8.13
INSTRUM spect
PROBHD 5 mm BBO BB-
PULPROG zg
TD 16384
SOLVENT DMSO
NS 6
DS 0
SWH 5995.204 Hz
FIDRES 0.365918 Hz
AQ 1.3664756 sec
RG 256
DW 83.400 usec
DE 6.00 usec
TE 300.0 K
D1 6.00000000 sec
CHANNEL f1
NUC1 1H
P1 9.00 usec
PL1 3.00 dB
SFO1 299.8729987 MH
F2 - Processing parameters
SI 32768
SF 299.8700035 MHz
WDW EM
SSB 0
LB 0.30 Hz
GB 0
PC 1.00
1 H-NMR with D2O exchange
ppm
2.167
1.972
2.055
2.078
3.000

^{1}H NMR of 4d

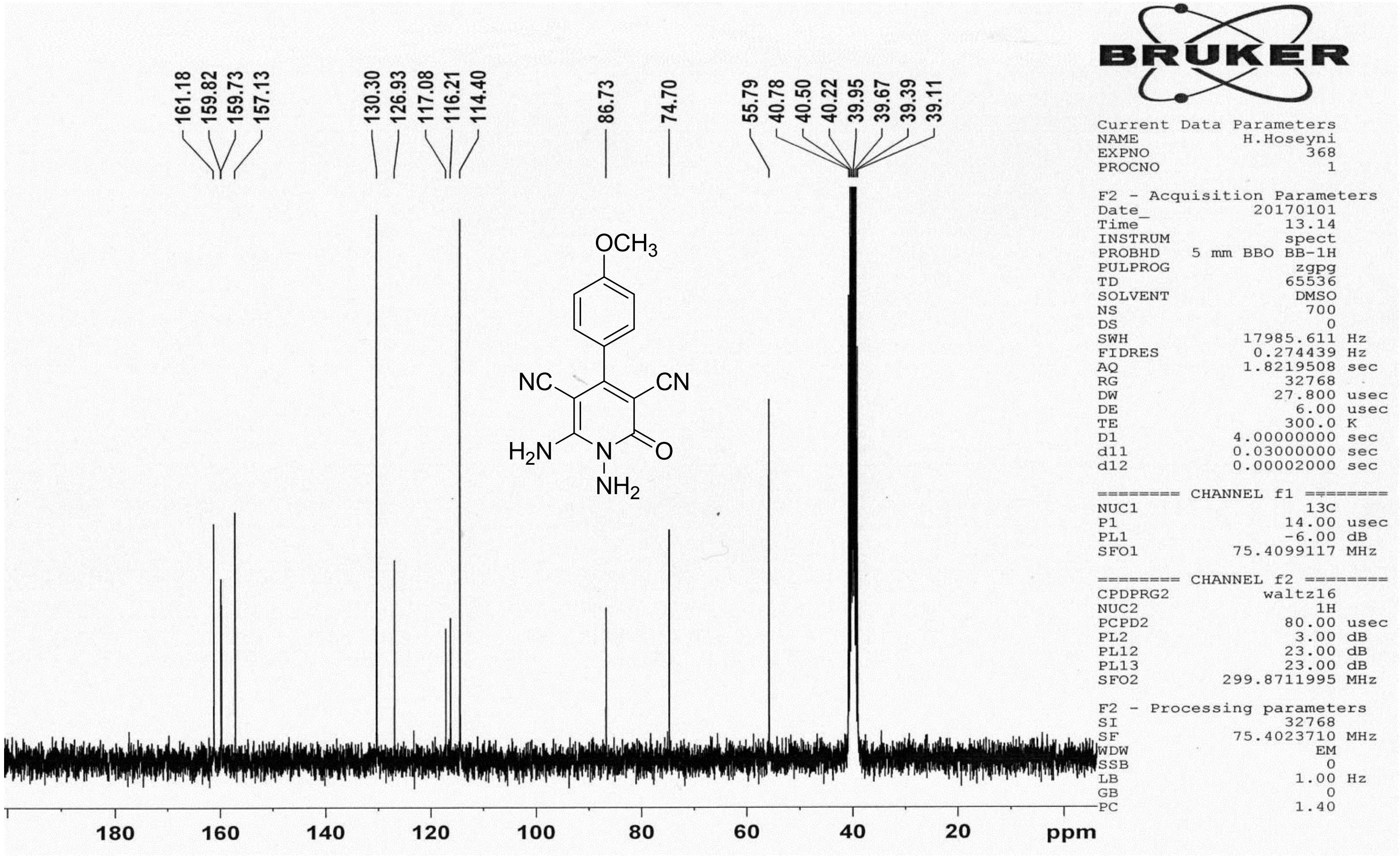
161.18
159.82
159.73
157.13
130.30
126.93
117.08
116.21
114.40
86.73
74.70
55.79
40.78
40.50
40.22
39.95
39.67
39.39
39.11
BRUKER
Current Data Parameters
NAME H.Hoseyni
EXPNO 368
PROCNO 1
F2 - Acquisition Parameters
Date_ 20170101
Time 13.14
INSTRUM spect
PROBHD 5 mm BBO BB-1H
PULPROG zgpg
TD 65536
SOLVENT DMSO
NS 700
DS 0
SWH 17985.611 Hz
FIDRES 0.274439 Hz
AQ 1.8219508 sec
RG 32768
DW 27.800 usec
DE 6.00 usec
TE 300.0 K
D1 4.00000000 sec
d11 0.03000000 sec
d12 0.00002000 sec
======== CHANNEL f1 ========
NUC1 13C
P1 14.00 usec
PL1 -6.00 dB
SFO1 75.4099117 MHz
======== CHANNEL f2 ========
CPDPRG2 waltz16
NUC2 1H
PCPD2 80.00 usec
PL2 3.00 dB
PL12 23.00 dB
PL13 23.00 dB
SFO2 299.8711995 MHz
F2 - Processing parameters
SI 32768
SF 75.4023710 MHz
WDW EM
SSB 0
LB 1.00 Hz
GB 0
PC 1.40
OCH3
NC
CN
H2N
N
O
NH2
180
160
140
120
100
80
60
40
20
ppm

^{13}C NMR of 4d

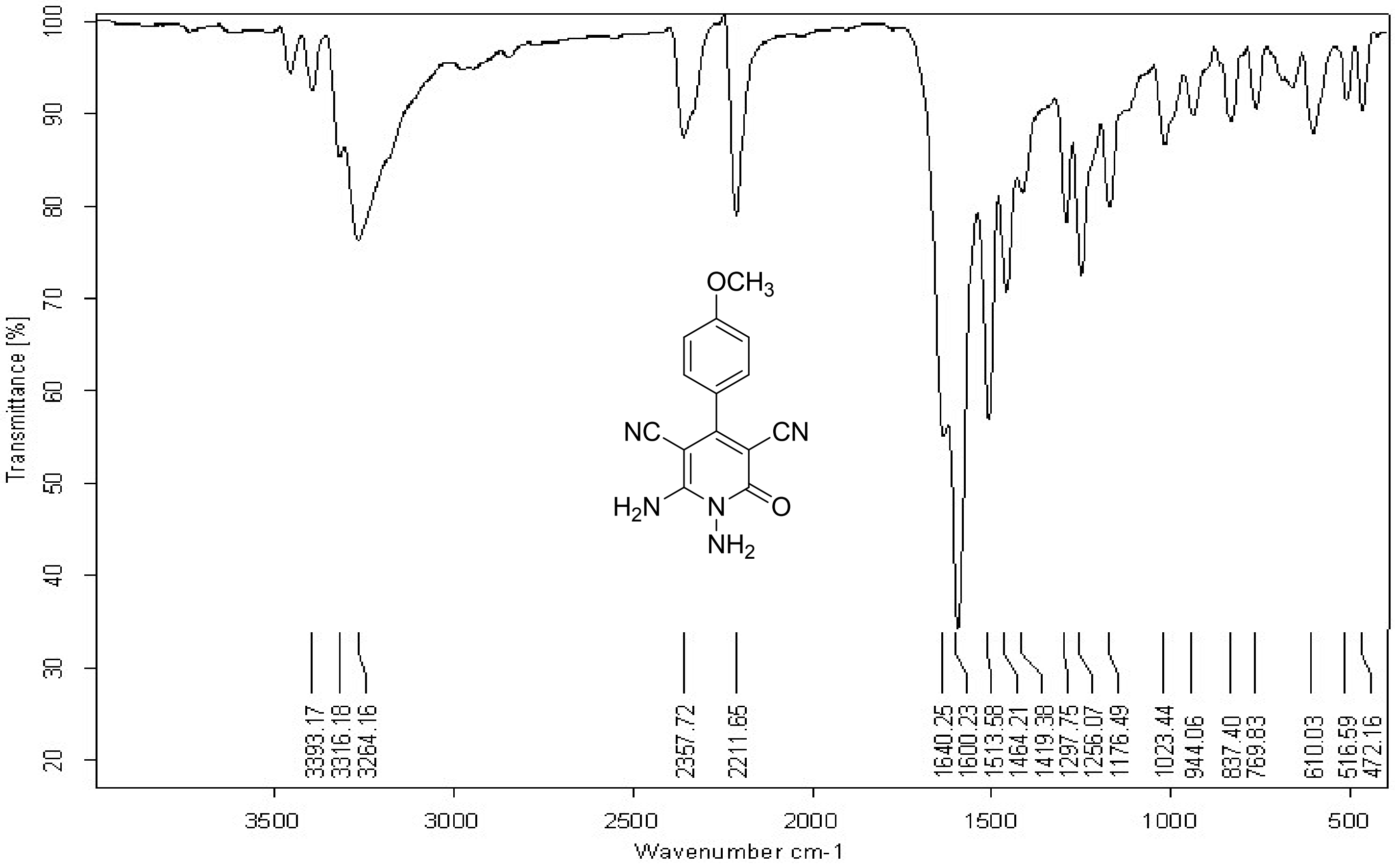

100
90
80
70
60
50
40
30
20
Transmittance [%]
3393.17
3316.18
3264.16
2357.72
2211.65
1640.25
1600.23
1513.58
1464.21
1419.38
1297.75
1256.07
1176.49
1023.44
944.06
837.40
769.83
610.03
516.59
472.16
3500
3000
2500
2000
1500
1000
500
Wavenumber cm-1
OCH3
NC
CN
H2N
N
O
NH2

IR of 4d

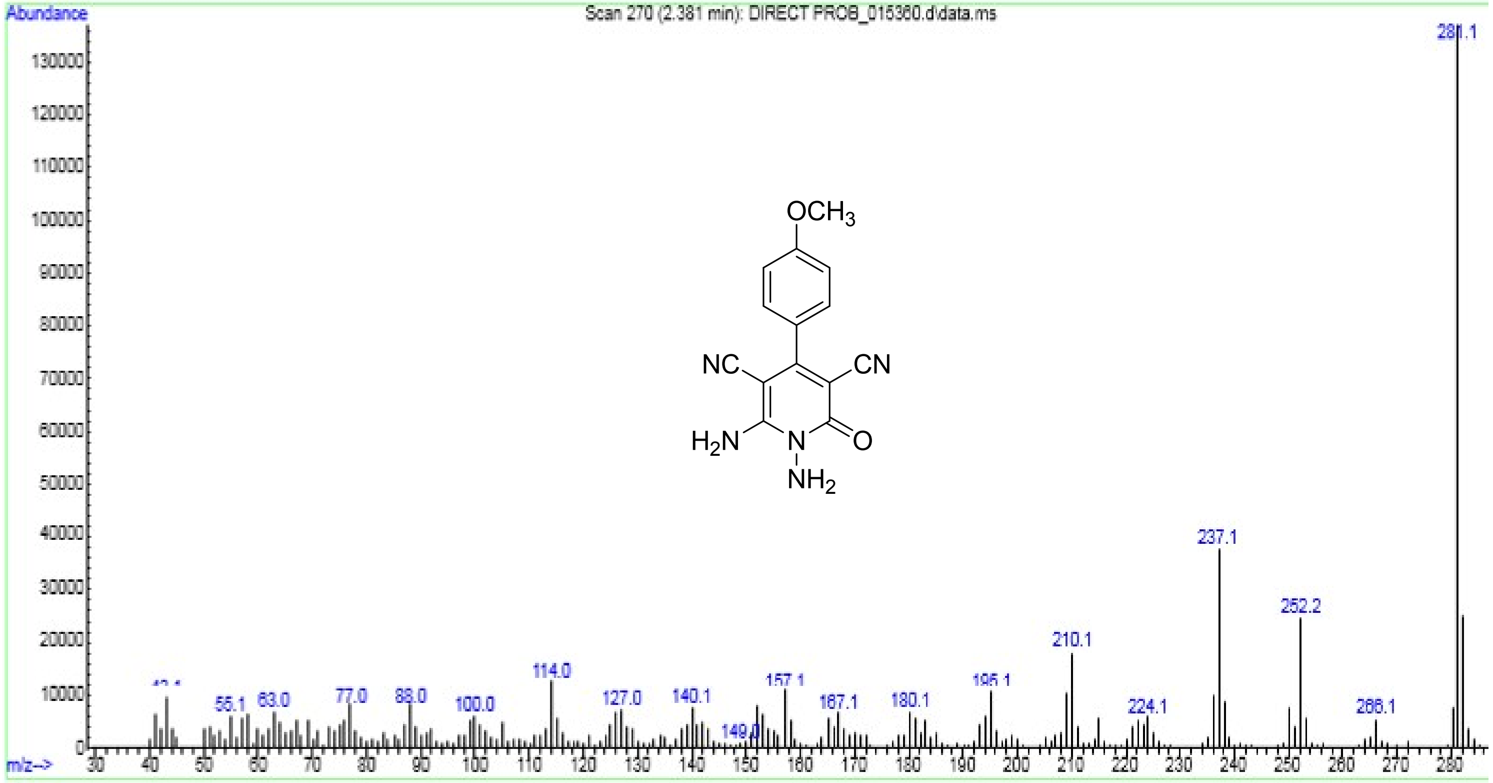
Abundance
Scan 270 (2.381 min): DIRECT PROB_015360.d\data.ms
OCH3
NC
CN
H2N
N
O
NH2
281.1
237.1
252.2
210.1
224.1
266.1
195.1
180.1
167.1
157.1
149.0
140.1
127.0
114.0
100.0
88.0
77.0
63.0
55.1
m/z-->

MS of 4d

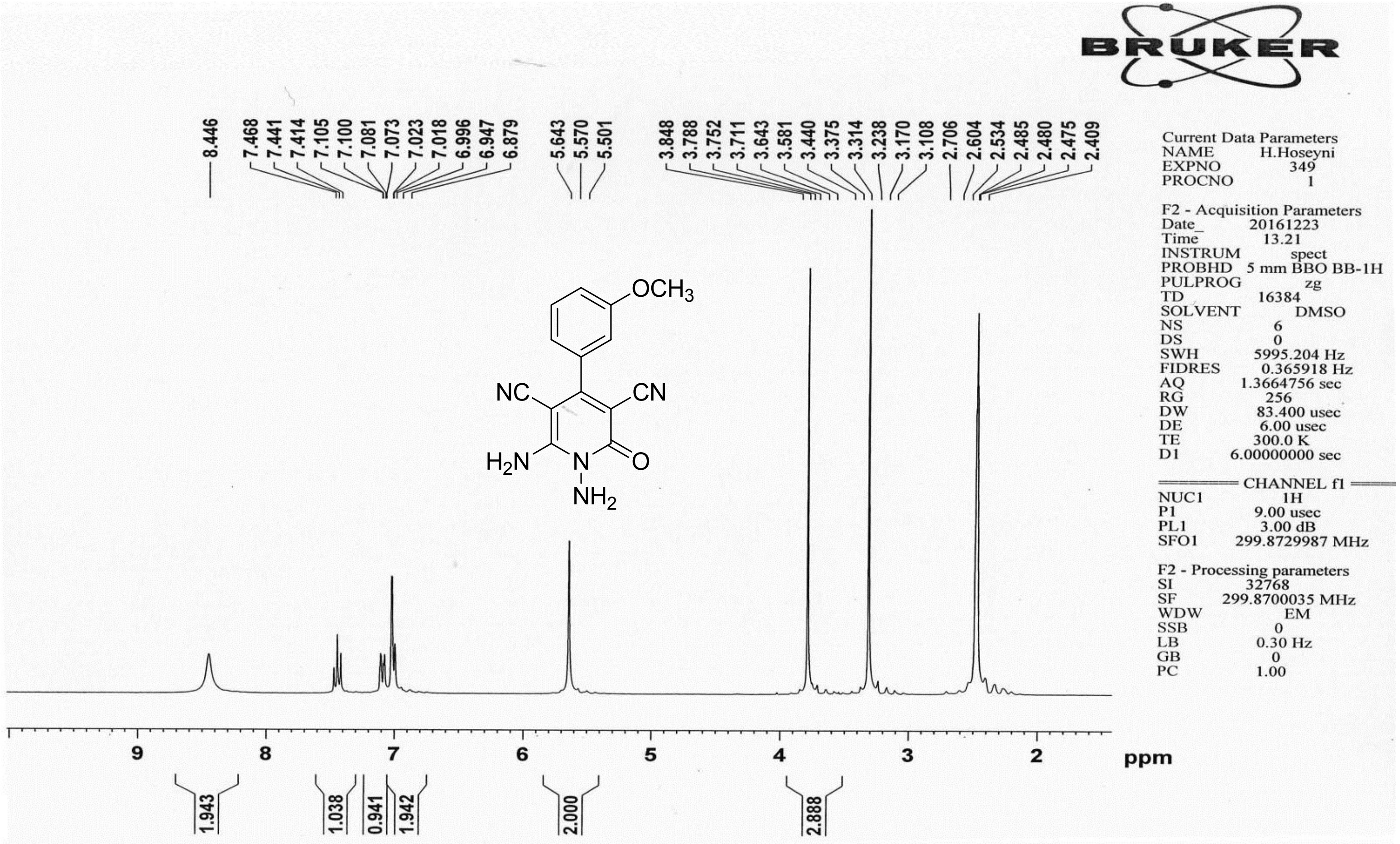

^{1}H NMR of 4e

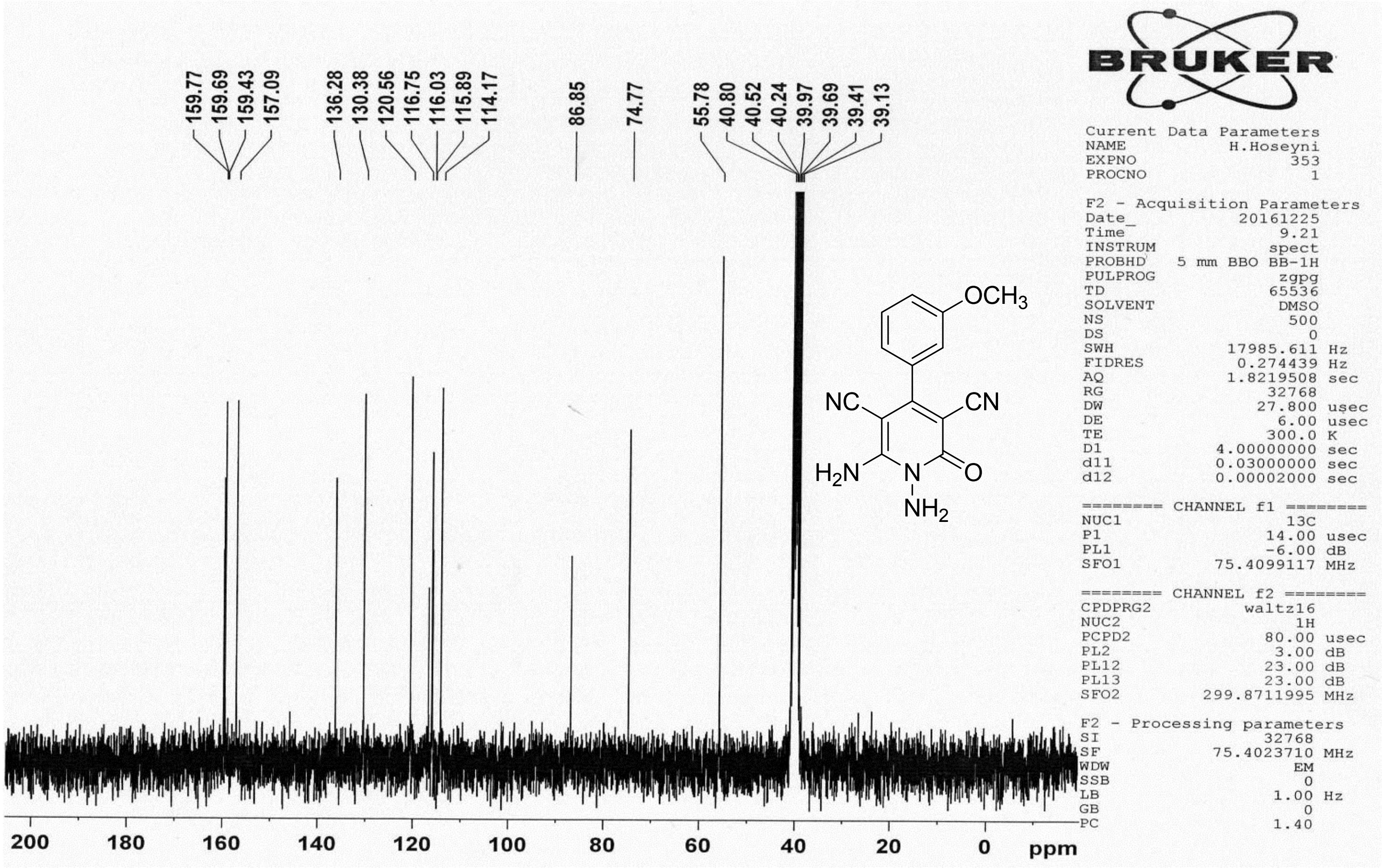
159.77
159.69
159.43
157.09
136.28
130.38
120.56
116.75
116.03
115.89
114.17
86.85
74.77
55.78
40.80
40.52
40.24
39.97
39.69
39.41
39.13
BRUKER
Current Data Parameters
NAME H.Hoseyni
EXPNO 353
PROCNO 1
F2 - Acquisition Parameters
Date_ 20161225
Time 9.21
INSTRUM spect
PROBHD 5 mm BBO BB-1H
PULPROG zgpg
TD 65536
SOLVENT DMSO
NS 500
DS 0
SWH 17985.611 Hz
FIDRES 0.274439 Hz
AQ 1.8219508 sec
RG 32768
DW 27.800 usec
DE 6.00 usec
TE 300.0 K
D1 4.00000000 sec
d11 0.03000000 sec
d12 0.00002000 sec
======== CHANNEL f1 ========
NUC1 13C
P1 14.00 usec
PL1 -6.00 dB
SFO1 75.4099117 MHz
======== CHANNEL f2 ========
CPDPRG2 waltz16
NUC2 1H
PCPD2 80.00 usec
PL2 3.00 dB
PL12 23.00 dB
PL13 23.00 dB
SFO2 299.8711995 MHz
F2 - Processing parameters
SI 32768
SF 75.4023710 MHz
WDW EM
SSB 0
LB 1.00 Hz
GB 0
PC 1.40
OCH3
NC
CN
H2N
N
O
NH2
200
180
160
140
120
100
80
60
40
20
0
ppm

^{13}C NMR of 4e

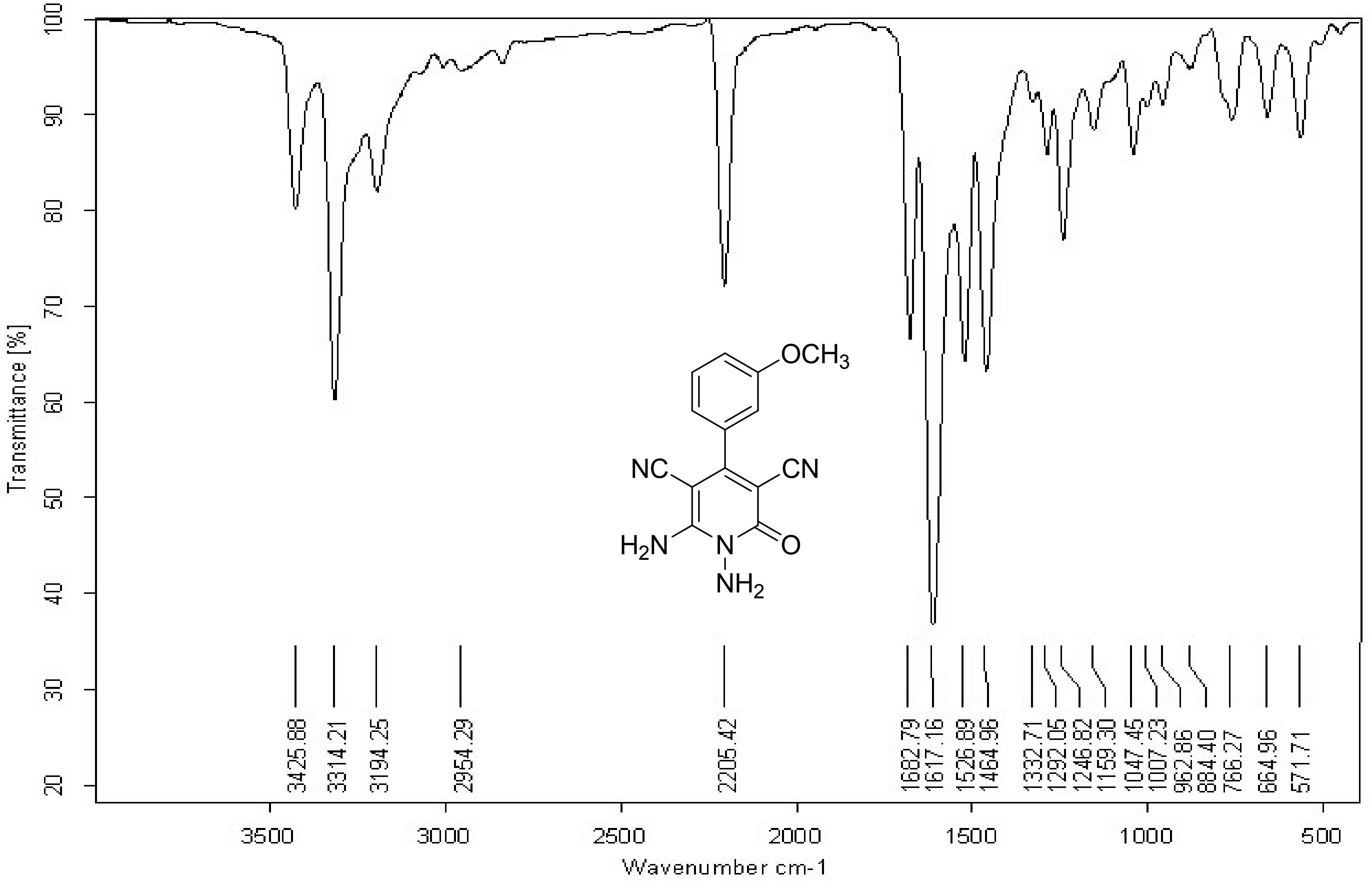

Transmittance [%]
Wavenumber cm-1
OCH3
NC
CN
H2N
N
O
NH2
3425.88
3314.21
3194.25
2954.29
2205.42
1682.79
1617.16
1526.89
1464.96
1332.71
1292.05
1246.82
1159.30
1047.45
1007.23
962.86
884.40
766.27
664.96
571.71

IR of 4e

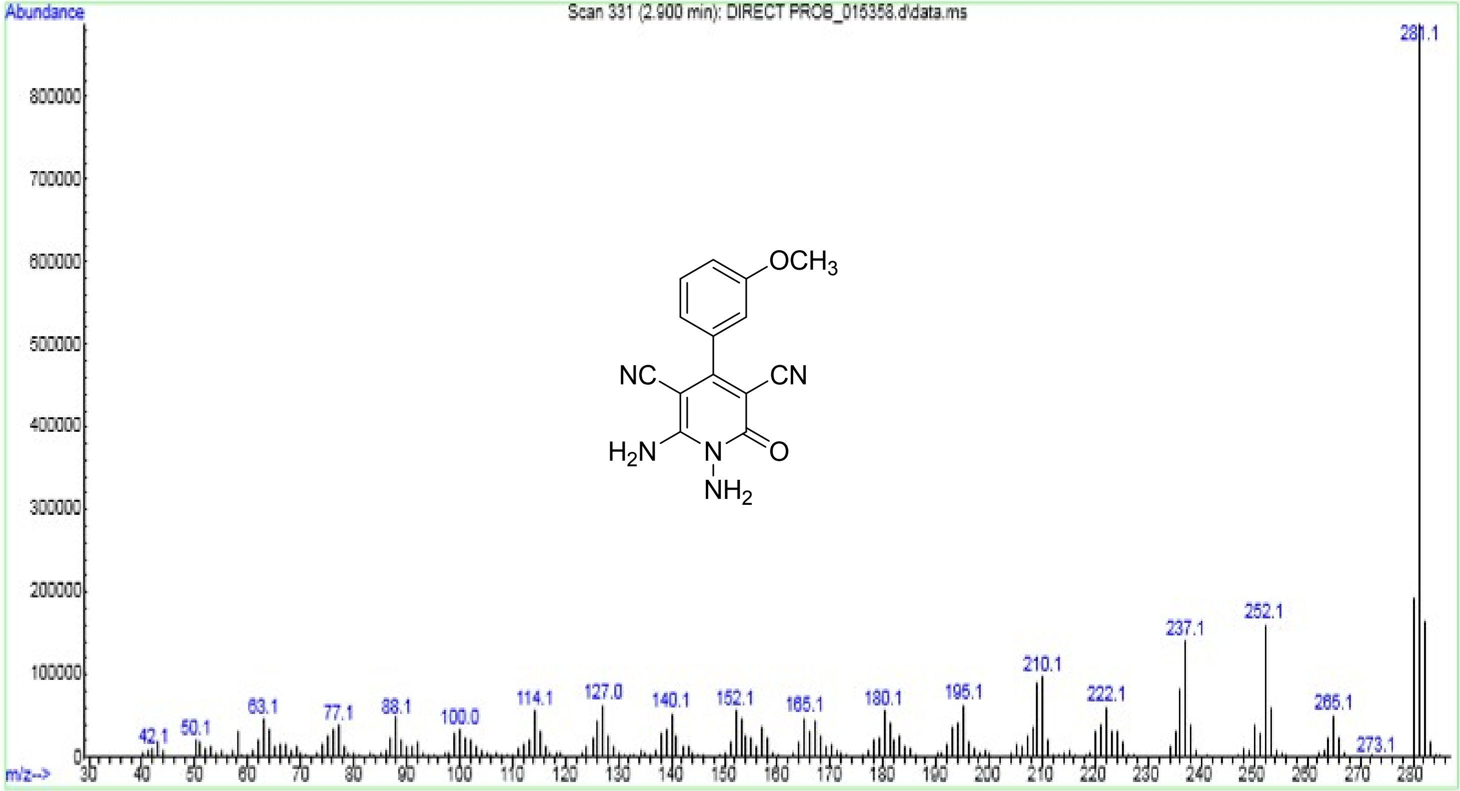
Abundance
Scan 331 (2.900 min): DIRECT PROB_015358.d\data.ms
OCH3
NC
CN
H2N
N
O
NH2
m/z-->
42.1
50.1
63.1
77.1
88.1
100.0
114.1
127.0
140.1
152.1
165.1
180.1
195.1
210.1
222.1
237.1
252.1
265.1
273.1

MS of 4e

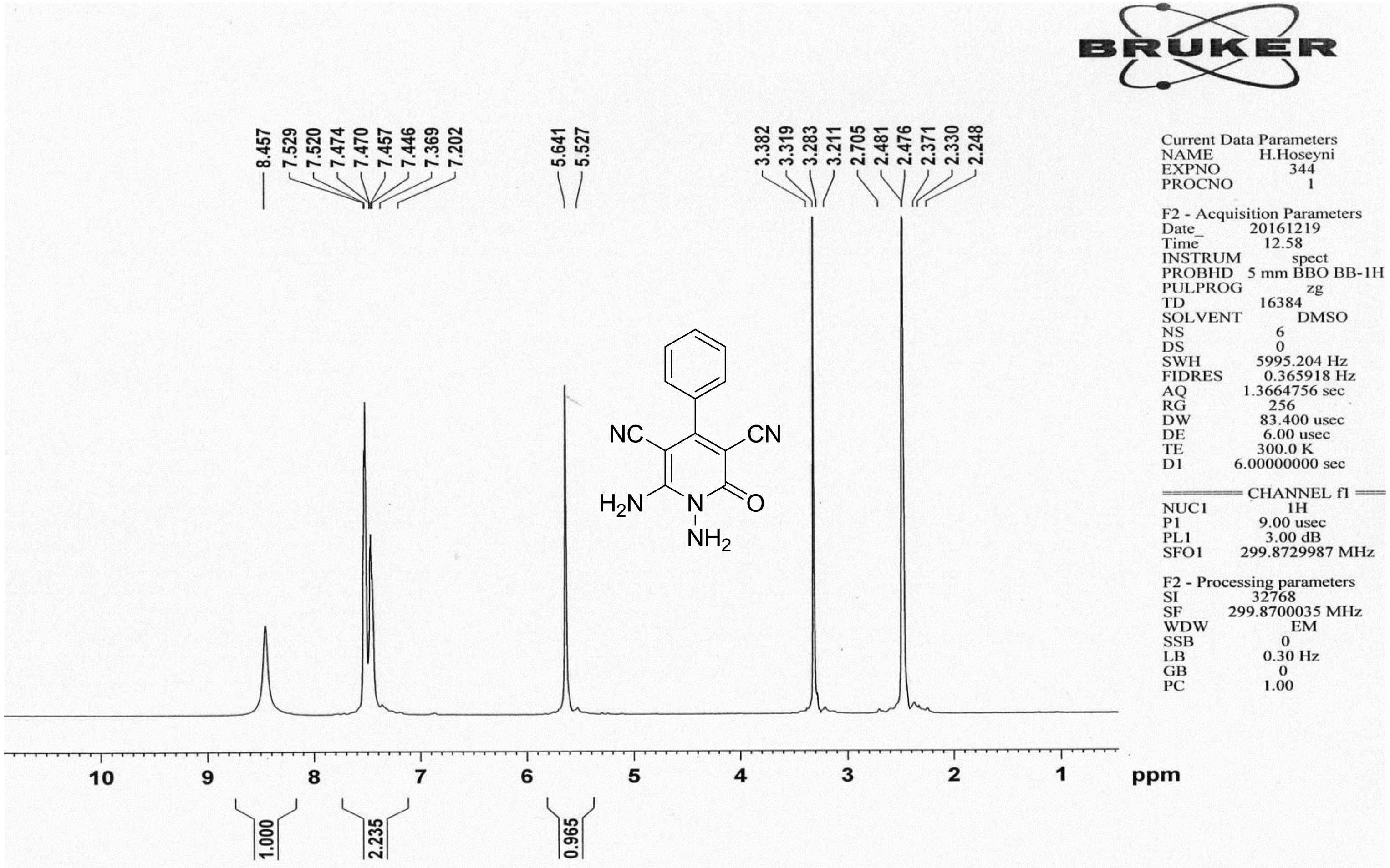
BRUKER
8.457
7.529
7.520
7.474
7.470
7.457
7.446
7.369
7.202
5.641
5.527
3.382
3.319
3.283
3.211
2.705
2.481
2.476
2.371
2.330
2.248
Current Data Parameters
NAME H.Hoseyni
EXPNO 344
PROCNO 1
F2 - Acquisition Parameters
Date_ 20161219
Time 12.58
INSTRUM spect
PROBHD 5 mm BBO BB-1H
PULPROG zg
TD 16384
SOLVENT DMSO
NS 6
DS 0
SWH 5995.204 Hz
FIDRES 0.365918 Hz
AQ 1.3664756 sec
RG 256
DW 83.400 usec
DE 6.00 usec
TE 300.0 K
D1 6.00000000 sec
CHANNEL f1
NUC1 1H
P1 9.00 usec
PL1 3.00 dB
SFO1 299.8729987 MHz
F2 - Processing parameters
SI 32768
SF 299.8700035 MHz
WDW EM
SSB 0
LB 0.30 Hz
GB 0
PC 1.00
NC
CN
H2N
N
O
NH2
10
9
8
7
6
5
4
3
2
1
ppm
1.000
2.235
0.965

^{1}H NMR of 4f

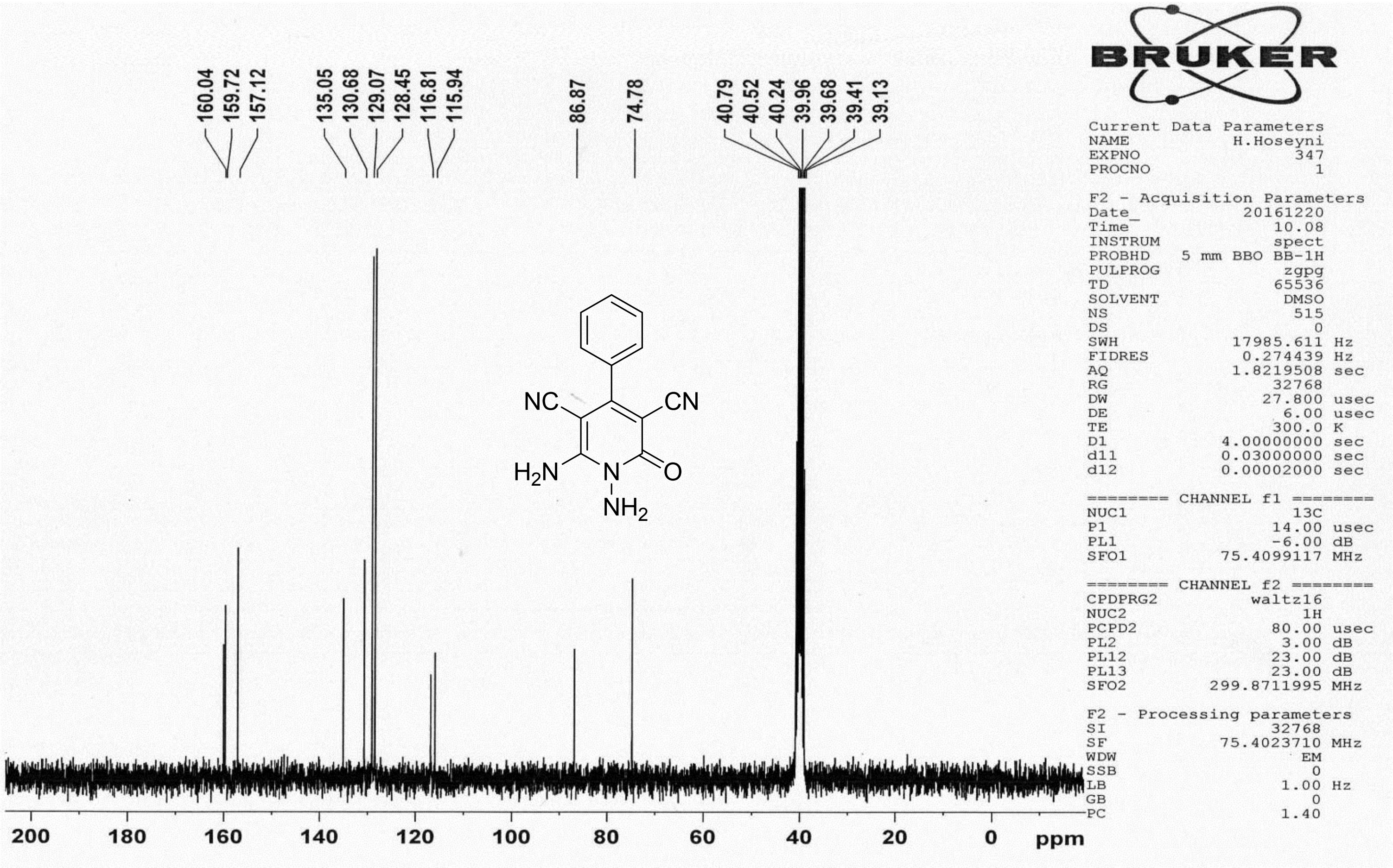

160.04
159.72
157.12
135.05
130.68
129.07
128.45
116.81
115.94
86.87
74.78
40.79
40.52
40.24
39.96
39.68
39.41
39.13
NC
CN
H2N
N
O
NH2
BRUKER
Current Data Parameters
NAME H.Hoseyni
EXPNO 347
PROCNO 1
F2 - Acquisition Parameters
Date_ 20161220
Time 10.08
INSTRUM spect
PROBHD 5 mm BBO BB-1H
PULPROG zgpg
TD 65536
SOLVENT DMSO
NS 515
DS 0
SWH 17985.611 Hz
FIDRES 0.274439 Hz
AQ 1.8219508 sec
RG 32768
DW 27.800 usec
DE 6.00 usec
TE 300.0 K
D1 4.00000000 sec
d11 0.03000000 sec
d12 0.00002000 sec
======== CHANNEL f1 ========
NUC1 13C
P1 14.00 usec
PL1 -6.00 dB
SFO1 75.4099117 MHz
======== CHANNEL f2 ========
CPDPRG2 waltz16
NUC2 1H
PCPD2 80.00 usec
PL2 3.00 dB
PL12 23.00 dB
PL13 23.00 dB
SFO2 299.8711995 MHz
F2 - Processing parameters
SI 32768
SF 75.4023710 MHz
WDW EM
SSB 0
LB 1.00 Hz
GB 0
PC 1.40
200 180 160 140 120 100 80 60 40 20 0 ppm

^{13}C NMR of 4f

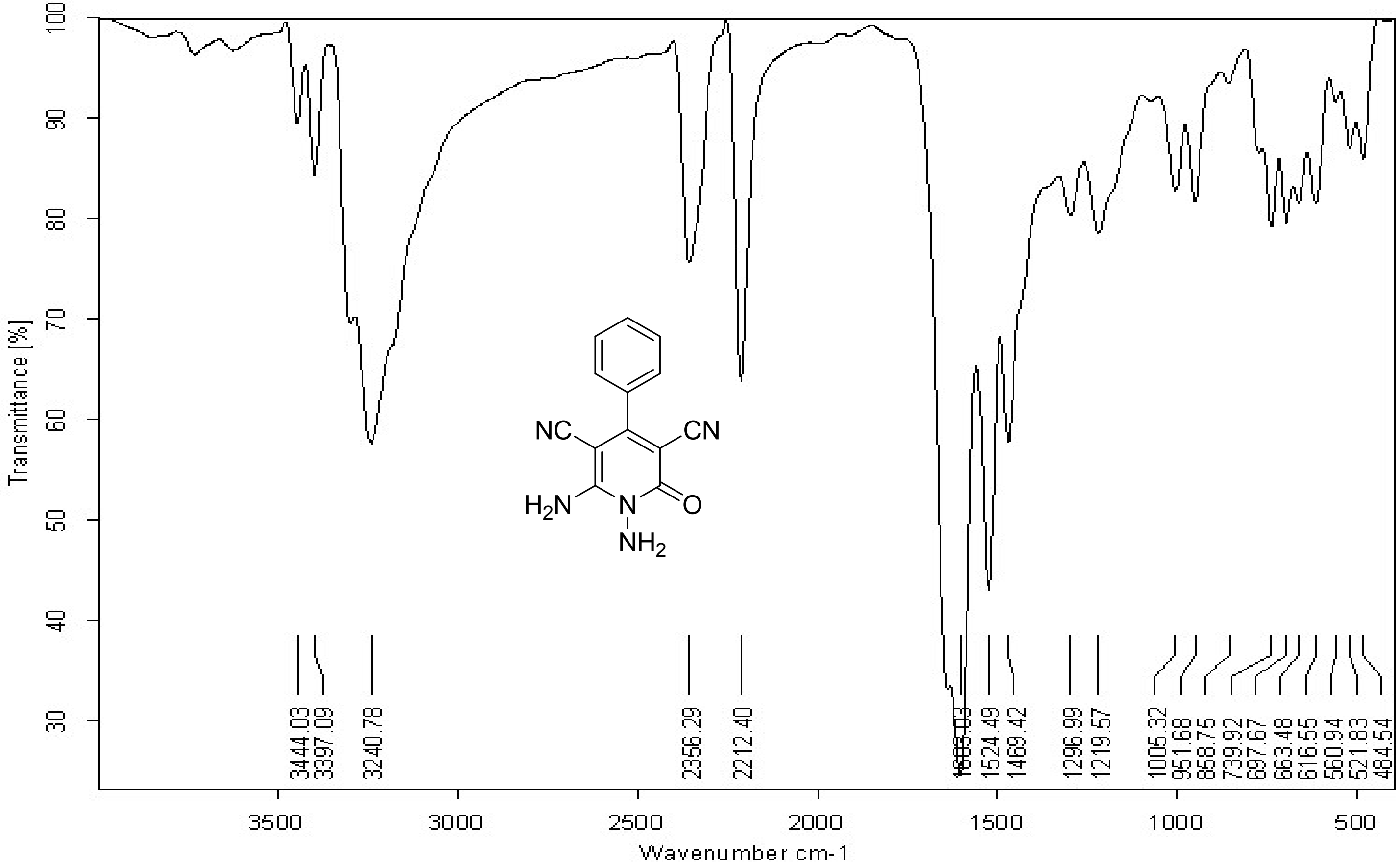
Transmittance [%]
100
90
80
70
60
50
40
30
NC
CN
H_2N
N
O
NH_2
3444.03
3397.09
3240.78
2356.29
2212.40
1524.49
1469.42
1296.99
1219.57
1005.32
951.68
858.75
739.92
697.67
663.48
616.55
560.94
521.83
484.54
3500
3000
2500
2000
1500
1000
500
Wavenumber cm-1

IR of 4f

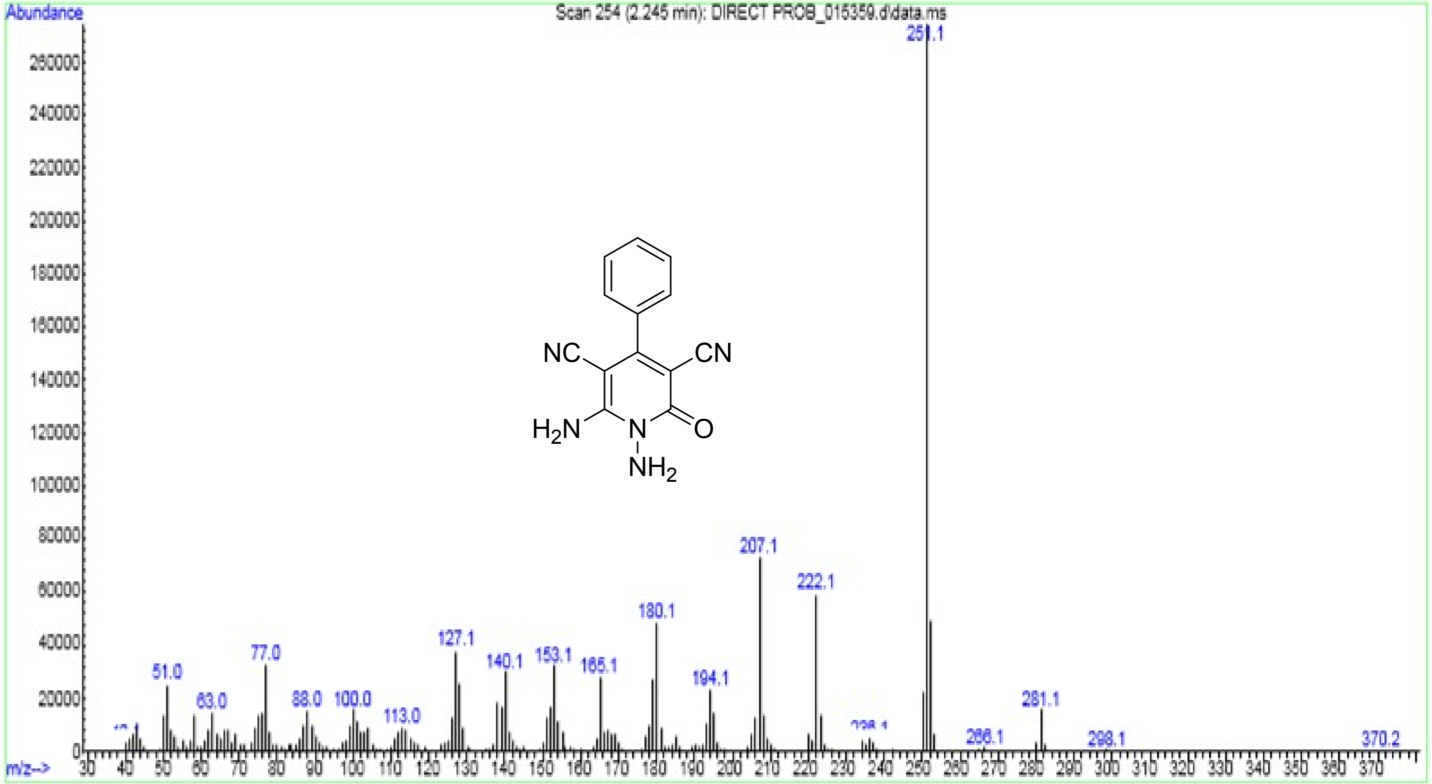
Abundance
Scan 254 (2.245 min): DIRECT PROB_015359.d\data.ms
260000
240000
220000
200000
180000
160000
140000
120000
100000
80000
60000
40000
20000
0
m/z-->
NC
CN
H2N
N
O
NH2
51.0
63.0
77.0
88.0
100.0
113.0
127.1
140.1
153.1
165.1
180.1
194.1
207.1
222.1
266.1
281.1
298.1
370.2

MS of 4f

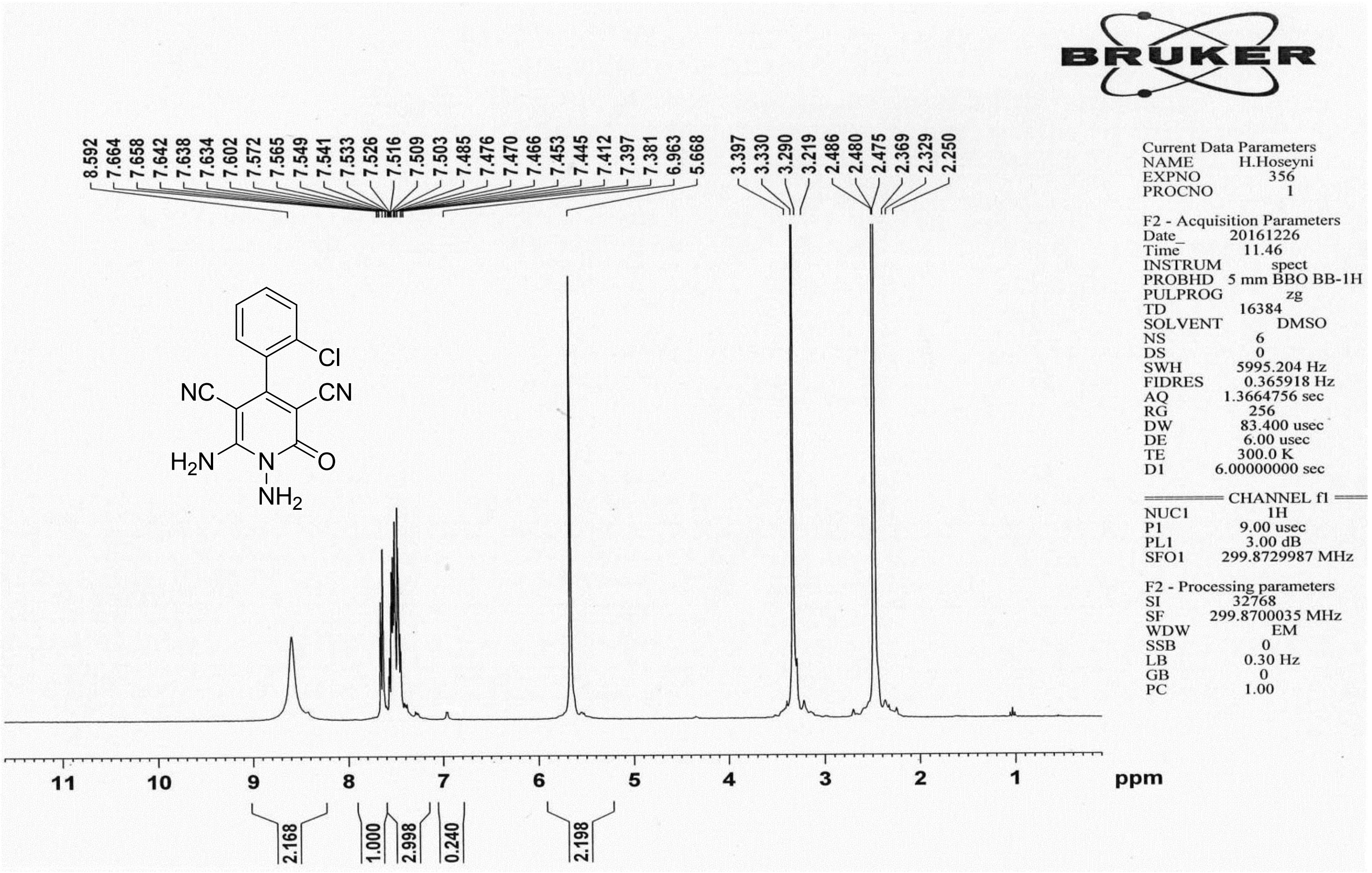
BRUKER
8.592
7.664
7.658
7.642
7.638
7.634
7.602
7.572
7.565
7.549
7.541
7.533
7.526
7.516
7.509
7.503
7.485
7.476
7.470
7.466
7.453
7.445
7.412
7.397
7.381
6.963
5.668
3.397
3.330
3.290
3.219
2.486
2.480
2.475
2.369
2.329
2.250
Cl
NC
CN
H2N
N
O
NH2
Current Data Parameters
NAME H.Hoseyni
EXPNO 356
PROCNO 1
F2 - Acquisition Parameters
Date_ 20161226
Time 11.46
INSTRUM spect
PROBHD 5 mm BBO BB-1H
PULPROG zg
TD 16384
SOLVENT DMSO
NS 6
DS 0
SWH 5995.204 Hz
FIDRES 0.365918 Hz
AQ 1.3664756 sec
RG 256
DW 83.400 usec
DE 6.00 usec
TE 300.0 K
D1 6.00000000 sec
CHANNEL f1
NUC1 1H
P1 9.00 usec
PL1 3.00 dB
SFO1 299.8729987 MHz
F2 - Processing parameters
SI 32768
SF 299.8700035 MHz
WDW EM
SSB 0
LB 0.30 Hz
GB 0
PC 1.00
11
10
9
8
7
6
5
4
3
2
1
ppm
2.168
1.000
2.998
0.240
2.198

^{1}H NMR of 4g

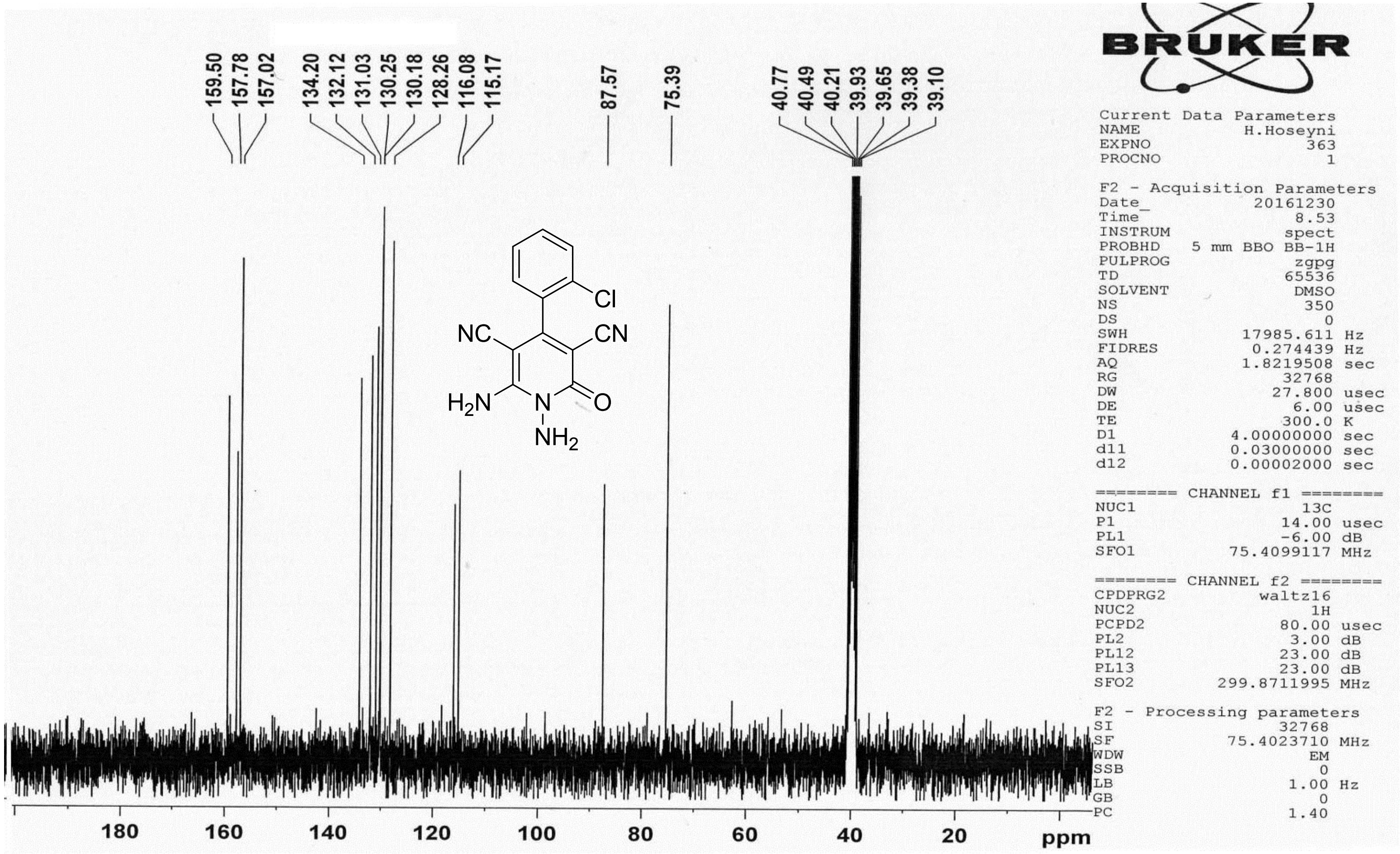
159.50
157.78
157.02
134.20
132.12
131.03
130.25
130.18
128.26
116.08
115.17
87.57
75.39
40.77
40.49
40.21
39.93
39.65
39.38
39.10
NC
CN
Cl
H_2N
N
O
NH_2
BRUKER
Current Data Parameters
NAME H.Hoseyni
EXPNO 363
PROCNO 1
F2 - Acquisition Parameters
Date_ 20161230
Time 8.53
INSTRUM spect
PROBHD 5 mm BBO BB-1H
PULPROG zgpg
TD 65536
SOLVENT DMSO
NS 350
DS 0
SWH 17985.611 Hz
FIDRES 0.274439 Hz
AQ 1.8219508 sec
RG 32768
DW 27.800 usec
DE 6.00 usec
TE 300.0 K
D1 4.00000000 sec
d11 0.03000000 sec
d12 0.00002000 sec
======== CHANNEL f1 ========
NUC1 13C
P1 14.00 usec
PL1 -6.00 dB
SFO1 75.4099117 MHz
======== CHANNEL f2 ========
CPDPRG2 waltz16
NUC2 1H
PCPD2 80.00 usec
PL2 3.00 dB
PL12 23.00 dB
PL13 23.00 dB
SFO2 299.8711995 MHz
F2 - Processing parameters
SI 32768
SF 75.4023710 MHz
WDW EM
SSB 0
LB 1.00 Hz
GB 0
PC 1.40
180
160
140
120
100
80
60
40
20
ppm

^{13}C NMR of 4g

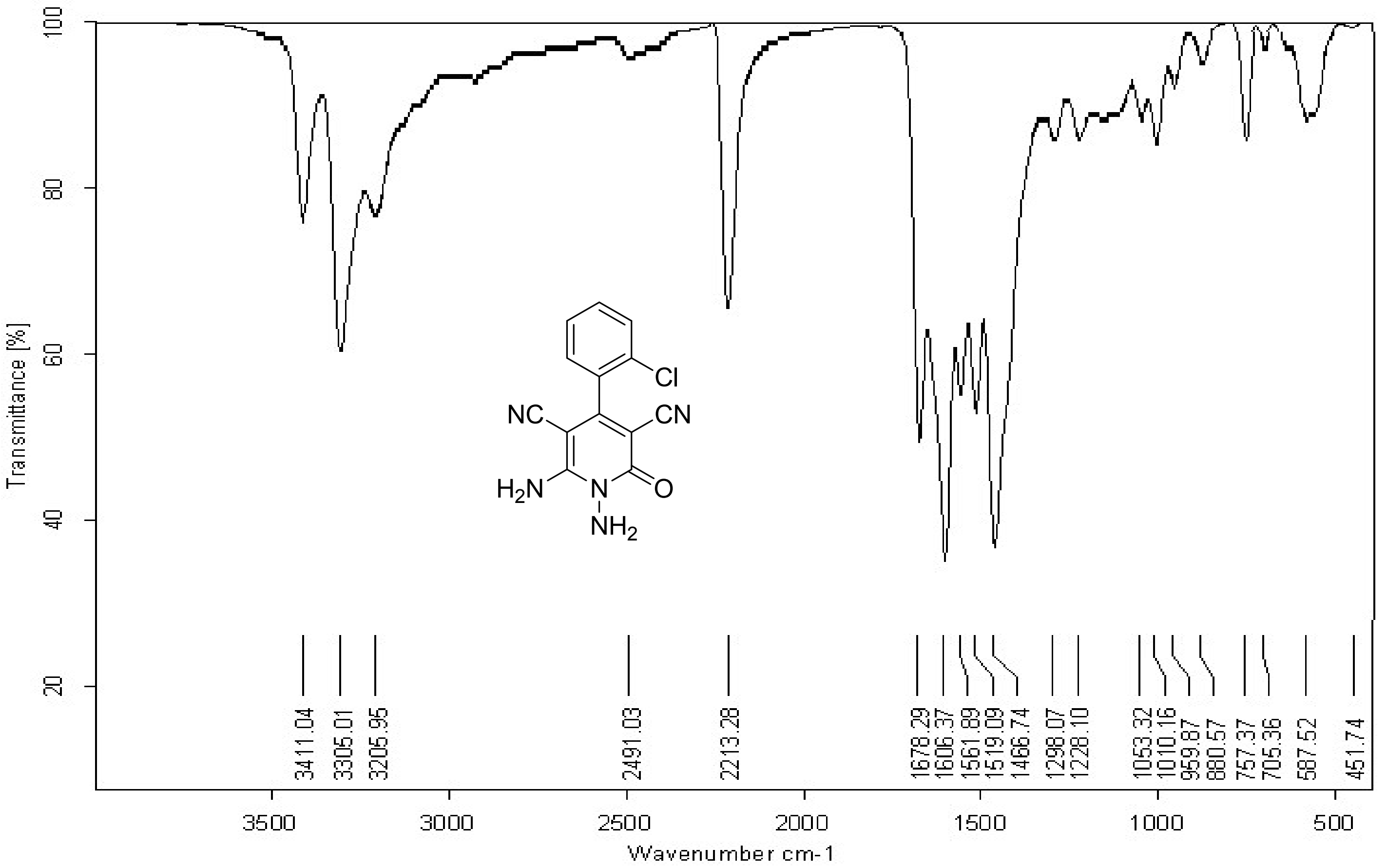
Transmittance [%]
100
80
60
40
20
3411.04
3305.01
3205.95
2491.03
2213.28
1678.29
1606.37
1561.89
1519.09
1466.74
1298.07
1228.10
1053.32
1010.16
959.87
880.57
757.37
705.36
587.52
451.74
3500
3000
2500
2000
1500
1000
500
Wavenumber cm-1
Cl
NC
CN
H_2N
N
O
NH_2

IR of 4g

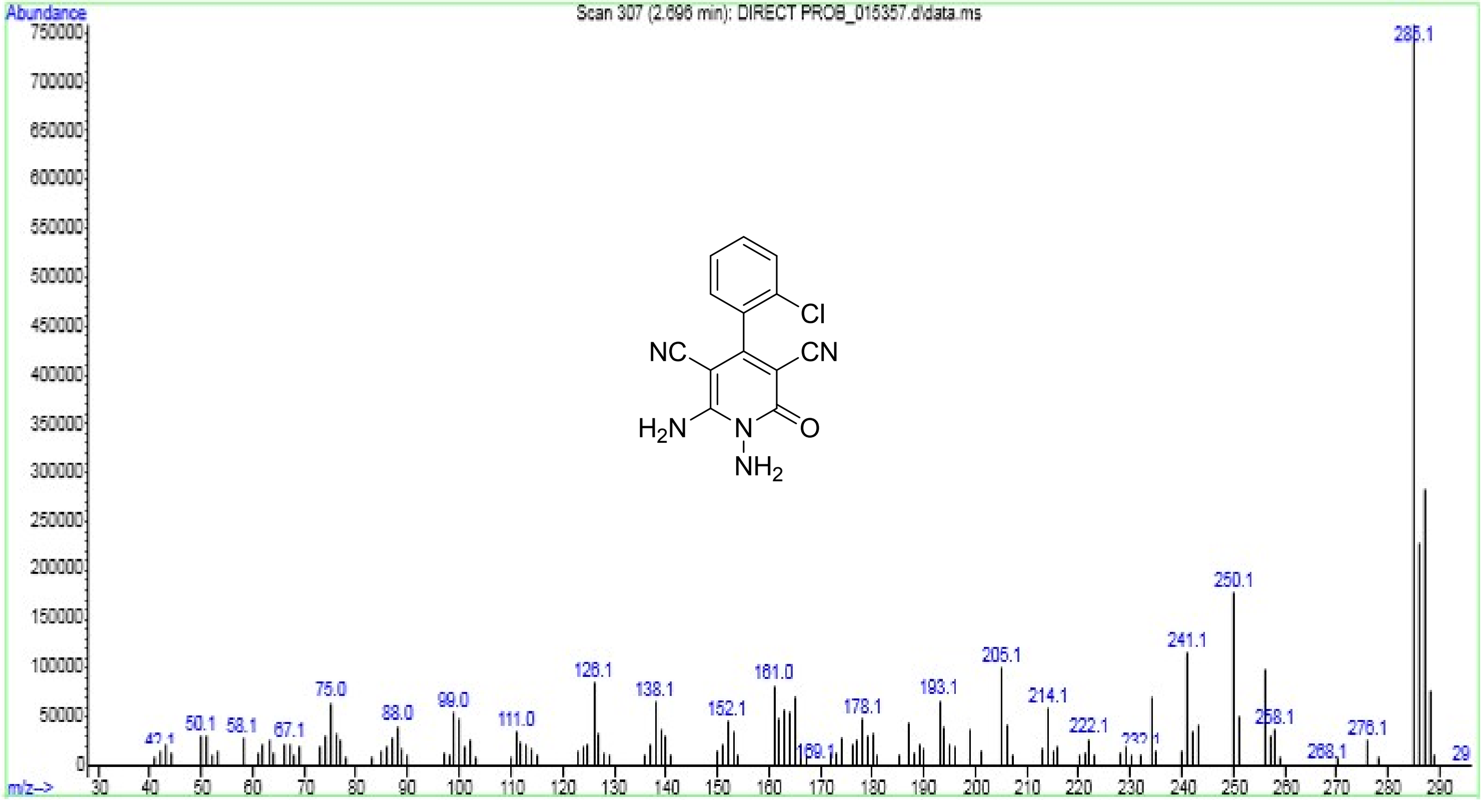
Abundance
Scan 307 (2.696 min): DIRECT PROB_015357.d\data.ms
m/z-->
285.1
250.1
241.1
205.1
193.1
161.0
126.1
138.1
152.1
178.1
214.1
258.1
276.1
222.1
268.1
169.1
111.0
99.0
88.0
75.0
67.1
58.1
50.1
Cl
NC
CN
H2N
N
O
NH2

MS of 4g

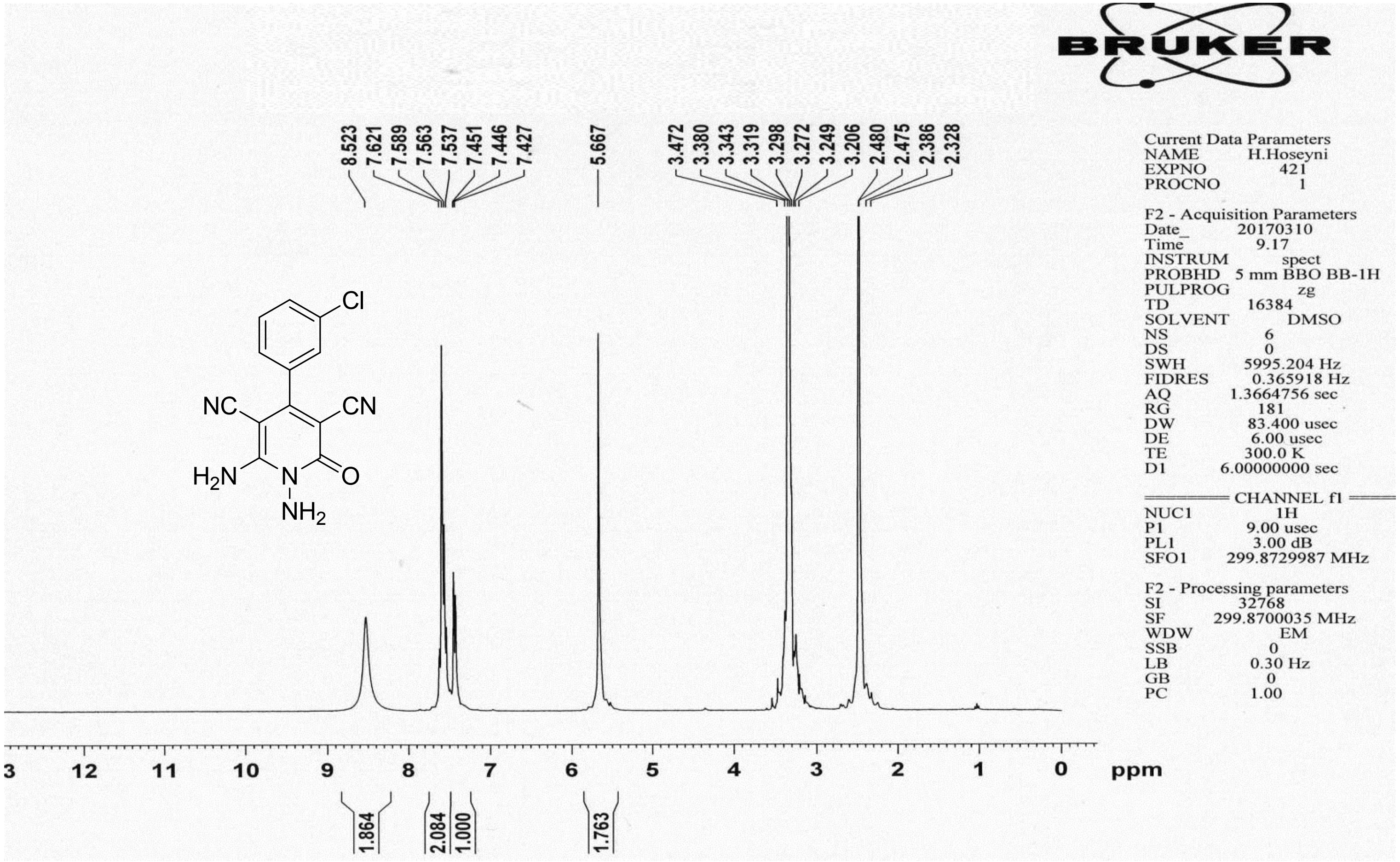

BRUKER
8.523
7.621
7.589
7.563
7.537
7.451
7.446
7.427
5.667
3.472
3.380
3.343
3.319
3.298
3.272
3.249
3.206
2.480
2.475
2.386
2.328
Cl
NC
CN
H2N
N
O
NH2
Current Data Parameters
NAME H.Hoseyni
EXPNO 421
PROCNO 1
F2 - Acquisition Parameters
Date_ 20170310
Time 9.17
INSTRUM spect
PROBHD 5 mm BBO BB-1H
PULPROG zg
TD 16384
SOLVENT DMSO
NS 6
DS 0
SWH 5995.204 Hz
FIDRES 0.365918 Hz
AQ 1.3664756 sec
RG 181
DW 83.400 usec
DE 6.00 usec
TE 300.0 K
D1 6.00000000 sec
CHANNEL f1
NUC1 1H
P1 9.00 usec
PL1 3.00 dB
SFO1 299.8729987 MHz
F2 - Processing parameters
SI 32768
SF 299.8700035 MHz
WDW EM
SSB 0
LB 0.30 Hz
GB 0
PC 1.00
3 12 11 10 9 8 7 6 5 4 3 2 1 0 ppm
1.864
2.084
1.000
1.763

^{1}H NMR of 4h

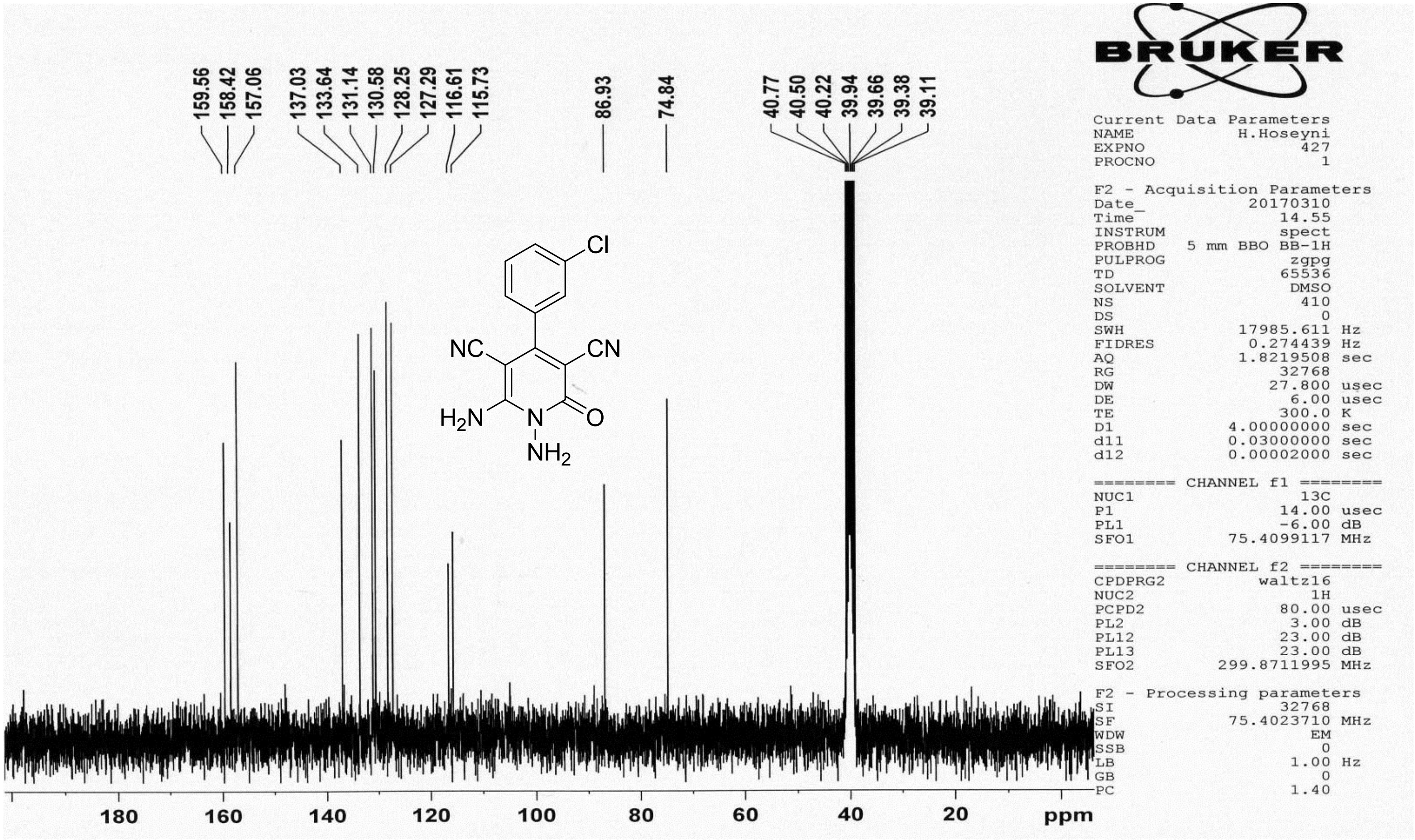
159.56
158.42
157.06
137.03
133.64
131.14
130.58
128.25
127.29
116.61
115.73
86.93
74.84
40.77
40.50
40.22
39.94
39.66
39.38
39.11
BRUKER
Current Data Parameters
NAME H.Hoseyni
EXPNO 427
PROCNO 1
F2 - Acquisition Parameters
Date_ 20170310
Time 14.55
INSTRUM spect
PROBHD 5 mm BBO BB-1H
PULPROG zgpg
TD 65536
SOLVENT DMSO
NS 410
DS 0
SWH 17985.611 Hz
FIDRES 0.274439 Hz
AQ 1.8219508 sec
RG 32768
DW 27.800 usec
DE 6.00 usec
TE 300.0 K
D1 4.00000000 sec
d11 0.03000000 sec
d12 0.00002000 sec
======== CHANNEL f1 ========
NUC1 13C
P1 14.00 usec
PL1 -6.00 dB
SFO1 75.4099117 MHz
======== CHANNEL f2 ========
CPDPRG2 waltz16
NUC2 1H
PCPD2 80.00 usec
PL2 3.00 dB
PL12 23.00 dB
PL13 23.00 dB
SFO2 299.8711995 MHz
F2 - Processing parameters
SI 32768
SF 75.4023710 MHz
WDW EM
SSB 0
LB 1.00 Hz
GB 0
PC 1.40
180
160
140
120
100
80
60
40
20
ppm

^{13}C NMR of 4h

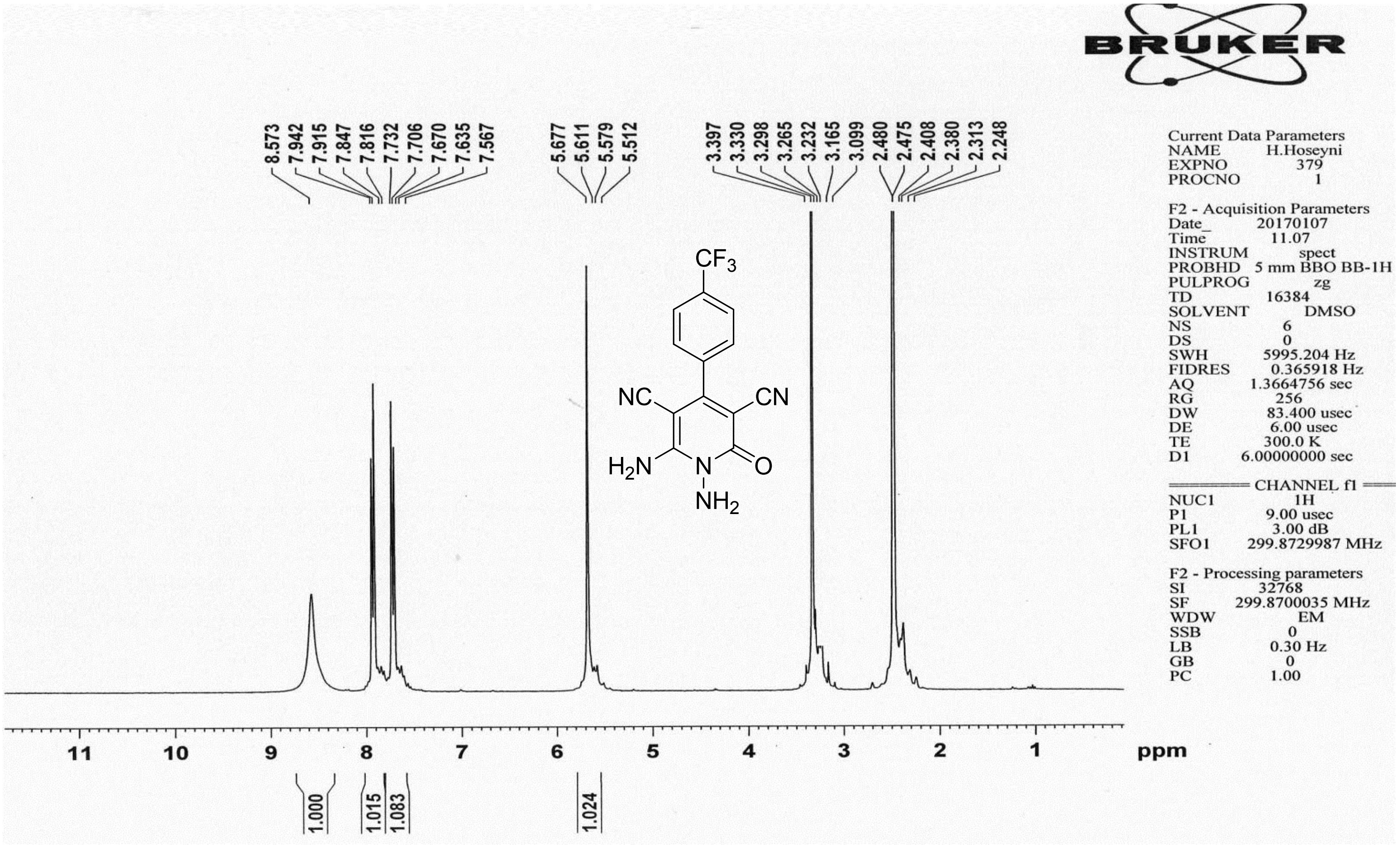
BRUKER
8.573
7.942
7.915
7.847
7.816
7.732
7.706
7.670
7.635
7.567
5.677
5.611
5.579
5.512
3.397
3.330
3.298
3.265
3.232
3.165
3.099
2.480
2.475
2.408
2.380
2.313
2.248
CF3
NC
CN
H2N
N
O
NH2
Current Data Parameters
NAME H.Hoseyni
EXPNO 379
PROCNO 1
F2 - Acquisition Parameters
Date_ 20170107
Time 11.07
INSTRUM spect
PROBHD 5 mm BBO BB-1H
PULPROG zg
TD 16384
SOLVENT DMSO
NS 6
DS 0
SWH 5995.204 Hz
FIDRES 0.365918 Hz
AQ 1.3664756 sec
RG 256
DW 83.400 usec
DE 6.00 usec
TE 300.0 K
D1 6.00000000 sec
CHANNEL f1
NUC1 1H
P1 9.00 usec
PL1 3.00 dB
SFO1 299.8729987 MHz
F2 - Processing parameters
SI 32768
SF 299.8700035 MHz
WDW EM
SSB 0
LB 0.30 Hz
GB 0
PC 1.00
11
10
9
8
7
6
5
4
3
2
1
ppm
1.000
1.015
1.083
1.024

^{1}H NMR of 4i

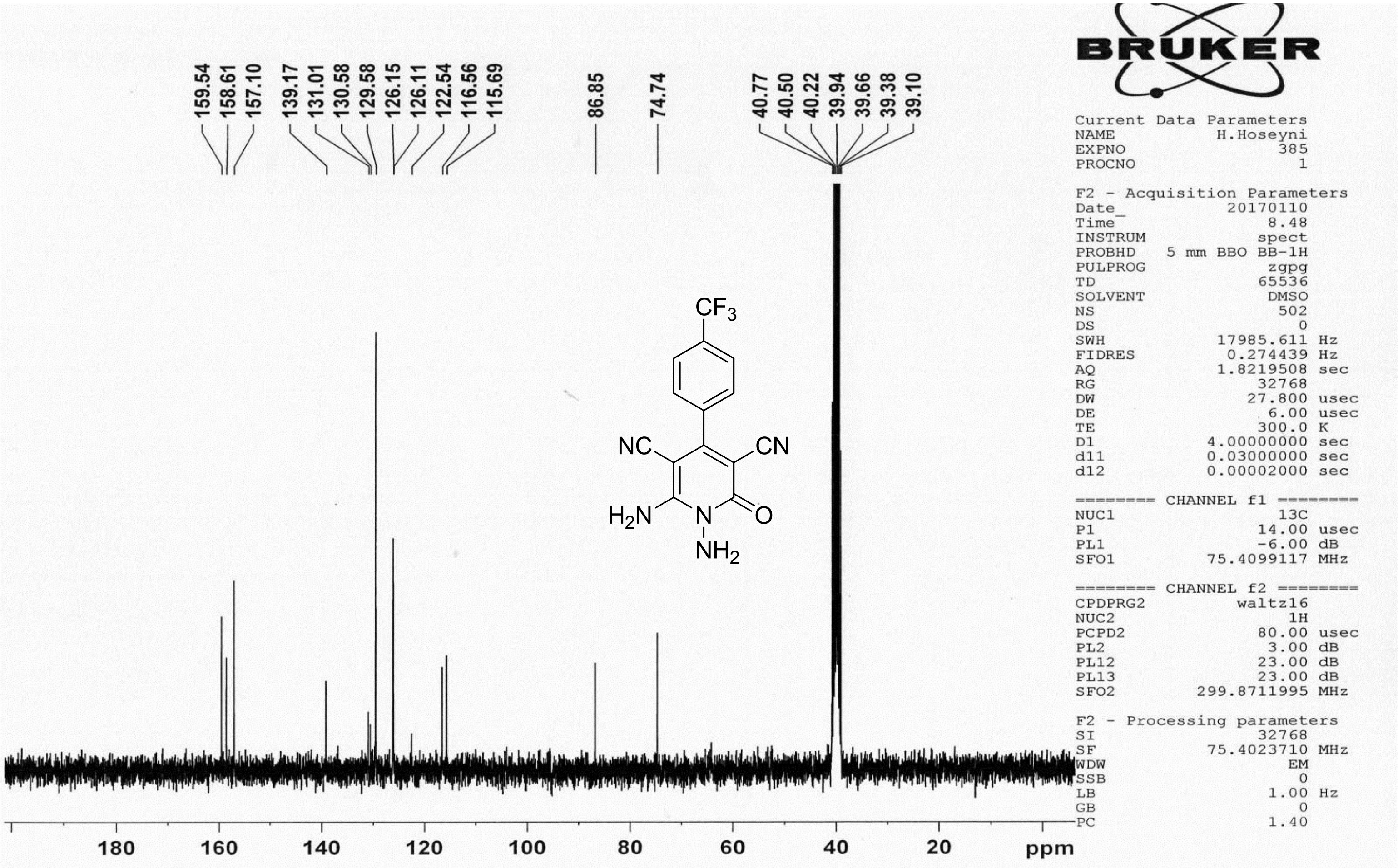
159.54
158.61
157.10
139.17
131.01
130.58
129.58
126.15
126.11
122.54
116.56
115.69
86.85
74.74
40.77
40.50
40.22
39.94
39.66
39.38
39.10
BRUKER
Current Data Parameters
NAME H.Hoseyni
EXPNO 385
PROCNO 1
F2 - Acquisition Parameters
Date_ 20170110
Time 8.48
INSTRUM spect
PROBHD 5 mm BBO BB-1H
PULPROG zgpg
TD 65536
SOLVENT DMSO
NS 502
DS 0
SWH 17985.611 Hz
FIDRES 0.274439 Hz
AQ 1.8219508 sec
RG 32768
DW 27.800 usec
DE 6.00 usec
TE 300.0 K
D1 4.00000000 sec
d11 0.03000000 sec
d12 0.00002000 sec
======== CHANNEL f1 ========
NUC1 13C
P1 14.00 usec
PL1 -6.00 dB
SFO1 75.4099117 MHz
======== CHANNEL f2 ========
CPDPRG2 waltz16
NUC2 1H
PCPD2 80.00 usec
PL2 3.00 dB
PL12 23.00 dB
PL13 23.00 dB
SFO2 299.8711995 MHz
F2 - Processing parameters
SI 32768
SF 75.4023710 MHz
WDW EM
SSB 0
LB 1.00 Hz
GB 0
PC 1.40
CF3
NC
CN
H2N
N
O
NH2
180
160
140
120
100
80
60
40
20
ppm

^{13}C NMR of 4i

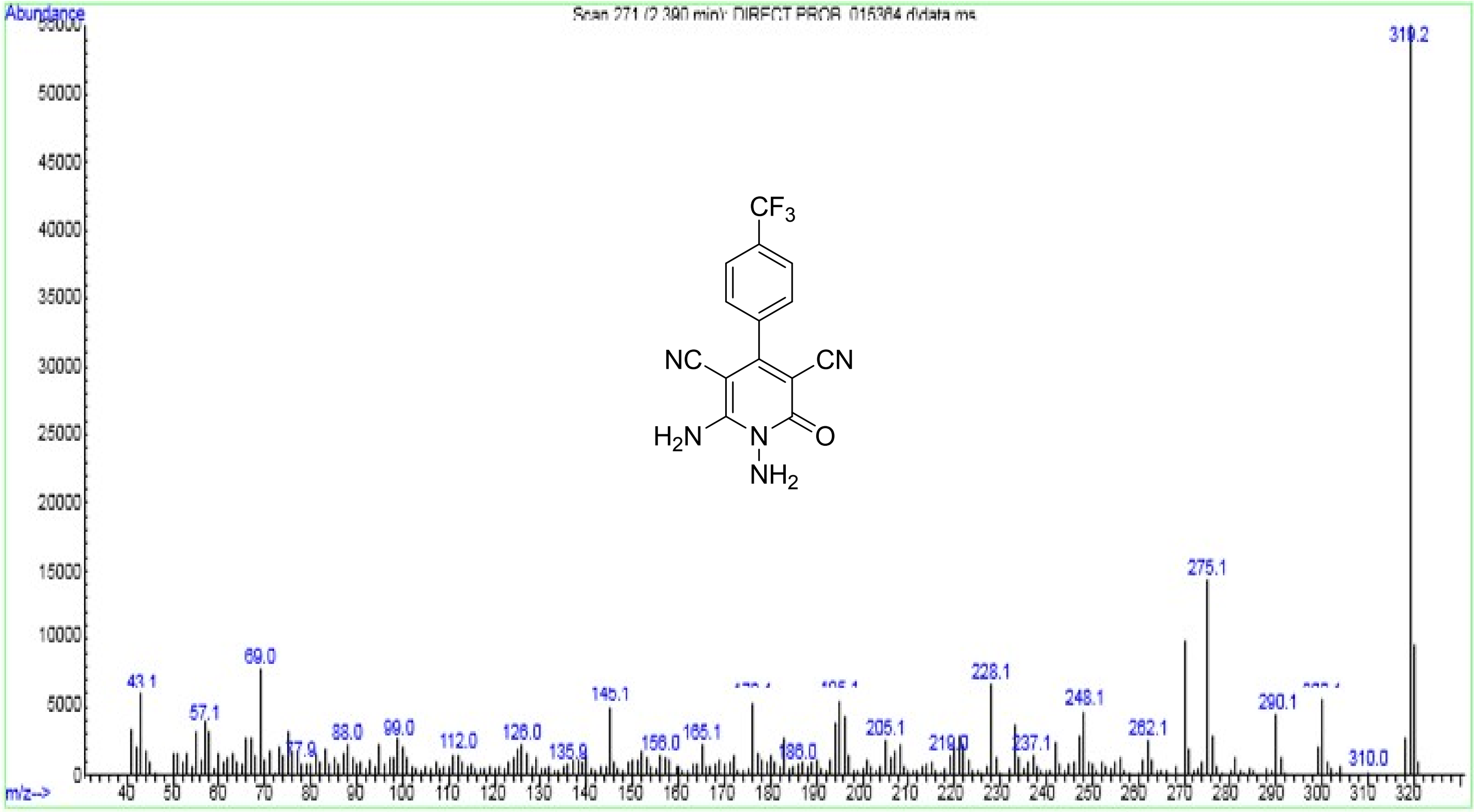
Abundance
Scan 271 (2.390 min): DIRECT PROB. 015384 d\data.ms
m/z-->
CF3
NC
CN
H2N
N
O
NH2
319.2
275.1
228.1
248.1
290.1
262.1
205.1
145.1
165.1
156.0
69.0
43.1
57.1
88.0
99.0
112.0
126.0
135.9
186.0
219.0
237.1
310.0

MS of 4i

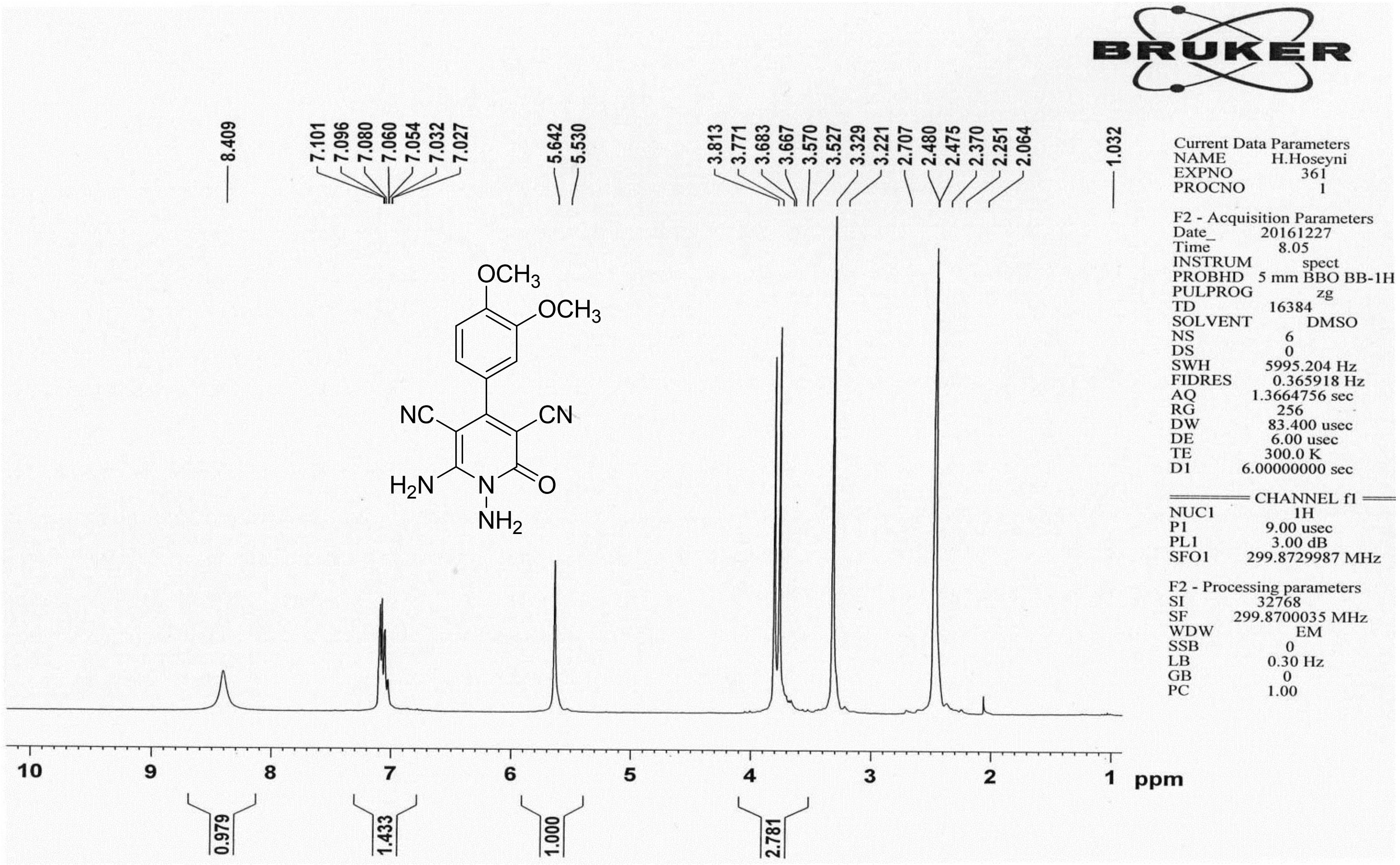
BRUKER
8.409
7.101
7.096
7.080
7.060
7.054
7.032
7.027
5.642
5.530
3.813
3.771
3.683
3.667
3.570
3.527
3.329
3.221
2.707
2.480
2.475
2.370
2.251
2.064
1.032
OCH3
OCH3
NC
CN
H2N
N
O
NH2
Current Data Parameters
NAME H.Hoseyni
EXPNO 361
PROCNO 1
F2 - Acquisition Parameters
Date_ 20161227
Time 8.05
INSTRUM spect
PROBHD 5 mm BBO BB-1H
PULPROG zg
TD 16384
SOLVENT DMSO
NS 6
DS 0
SWH 5995.204 Hz
FIDRES 0.365918 Hz
AQ 1.3664756 sec
RG 256
DW 83.400 usec
DE 6.00 usec
TE 300.0 K
D1 6.00000000 sec
CHANNEL f1
NUC1 1H
P1 9.00 usec
PL1 3.00 dB
SFO1 299.8729987 MHz
F2 - Processing parameters
SI 32768
SF 299.8700035 MHz
WDW EM
SSB 0
LB 0.30 Hz
GB 0
PC 1.00
10
9
8
7
6
5
4
3
2
1
ppm
0.979
1.433
1.000
2.781

^{1}H NMR of 4j

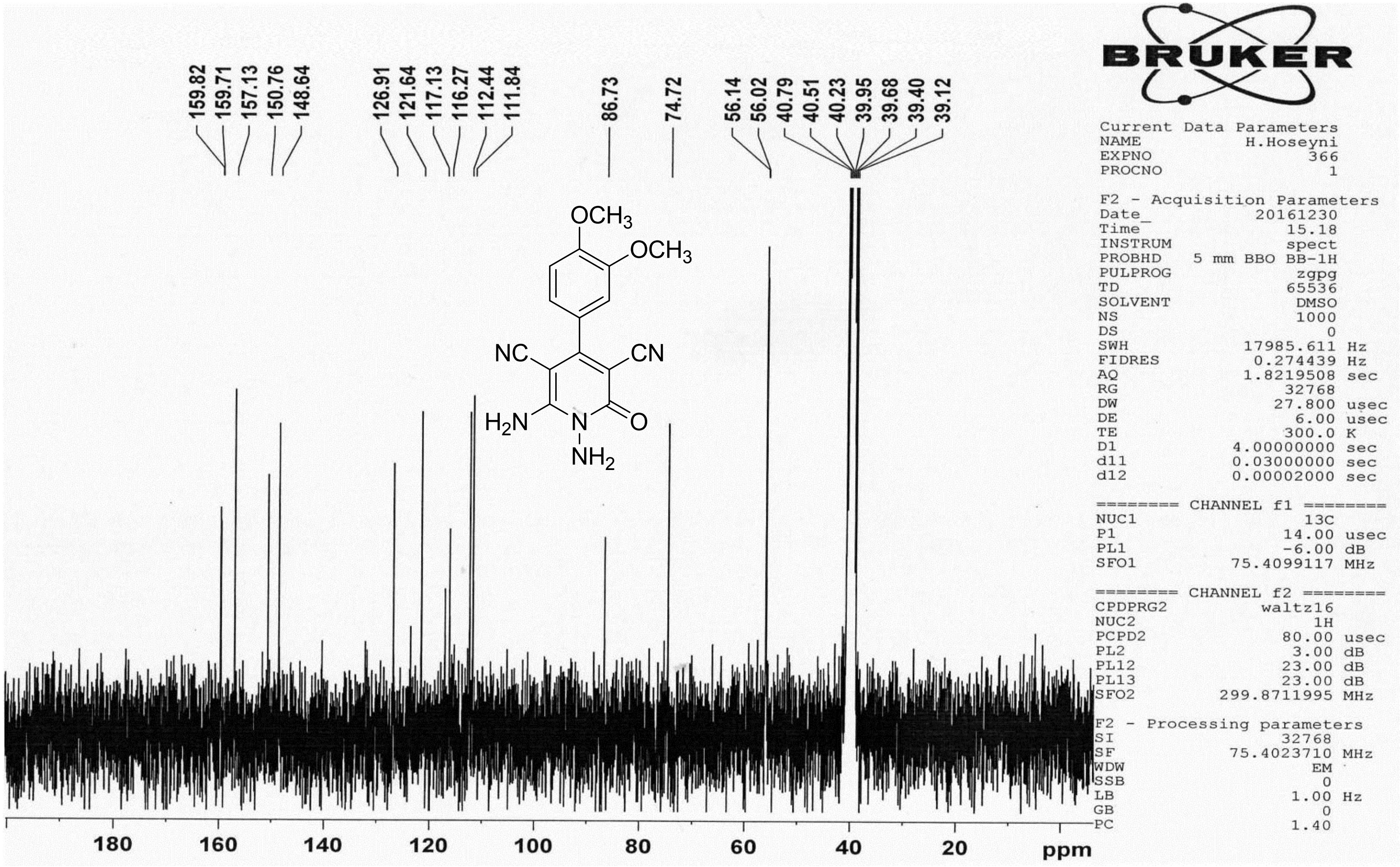
159.82
159.71
157.13
150.76
148.64
126.91
121.64
117.13
116.27
112.44
111.84
86.73
74.72
56.14
56.02
40.79
40.51
40.23
39.95
39.68
39.40
39.12
BRUKER
Current Data Parameters
NAME H.Hoseyni
EXPNO 366
PROCNO 1
F2 - Acquisition Parameters
Date_ 20161230
Time 15.18
INSTRUM spect
PROBHD 5 mm BBO BB-1H
PULPROG zgpg
TD 65536
SOLVENT DMSO
NS 1000
DS 0
SWH 17985.611 Hz
FIDRES 0.274439 Hz
AQ 1.8219508 sec
RG 32768
DW 27.800 usec
DE 6.00 usec
TE 300.0 K
D1 4.00000000 sec
d11 0.03000000 sec
d12 0.00002000 sec
======== CHANNEL f1 ========
NUC1 13C
P1 14.00 usec
PL1 -6.00 dB
SFO1 75.4099117 MHz
======== CHANNEL f2 ========
CPDPRG2 waltz16
NUC2 1H
PCPD2 80.00 usec
PL2 3.00 dB
PL12 23.00 dB
PL13 23.00 dB
SFO2 299.8711995 MHz
F2 - Processing parameters
SI 32768
SF 75.4023710 MHz
WDW EM
SSB 0
LB 1.00 Hz
GB 0
PC 1.40
OCH3
OCH3
NC
CN
H2N
N
O
NH2
180
160
140
120
100
80
60
40
20
ppm

^{13}C NMR of 4j

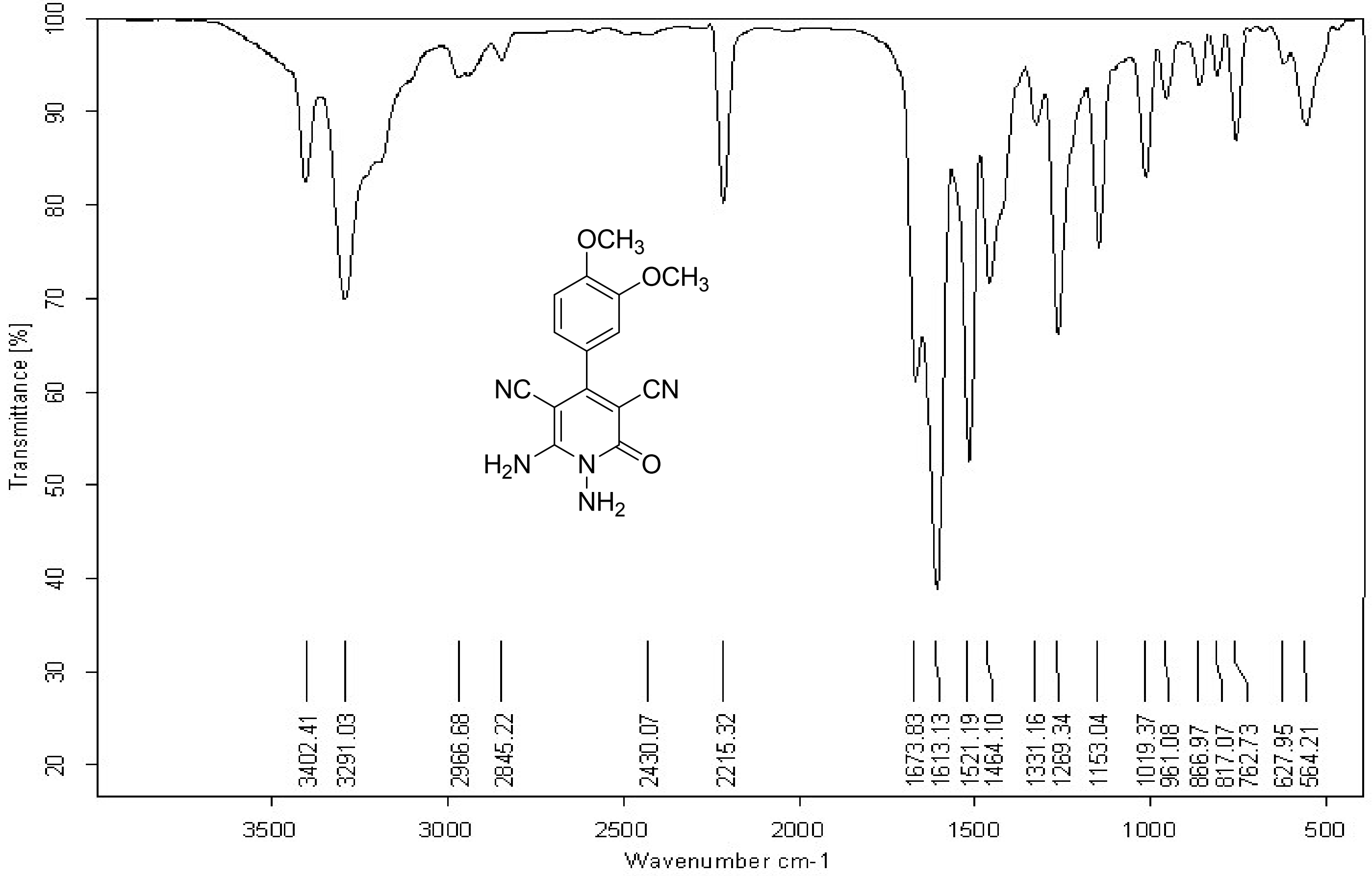
OCH3
OCH3
NC
CN
H2N
N
O
NH2
Transmittance [%]
Wavenumber cm-1
3402.41
3291.03
2966.68
2845.22
2430.07
2215.32
1673.83
1613.13
1521.19
1464.10
1331.16
1269.34
1153.04
1019.37
961.08
866.97
817.07
762.73
627.95
564.21

IR of 4j

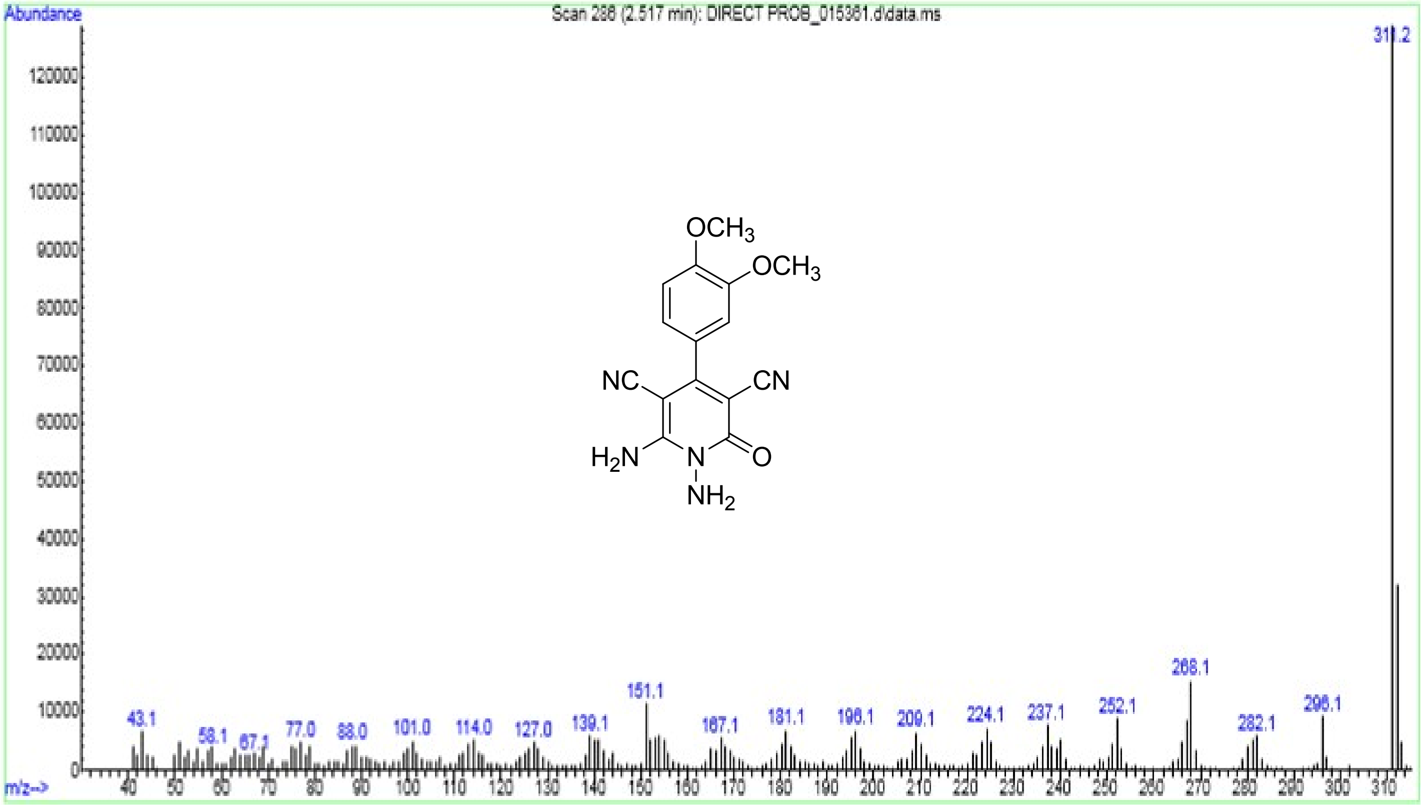
Abundance
Scan 288 (2.517 min): DIRECT PROB_015361.d\data.ms
m/z-->
43.1
58.1
67.1
77.0
88.0
101.0
114.0
127.0
139.1
151.1
167.1
181.1
196.1
209.1
224.1
237.1
252.1
268.1
282.1
296.1
OCH3
OCH3
NC
CN
H2N
N
O
NH2

MS of 4j

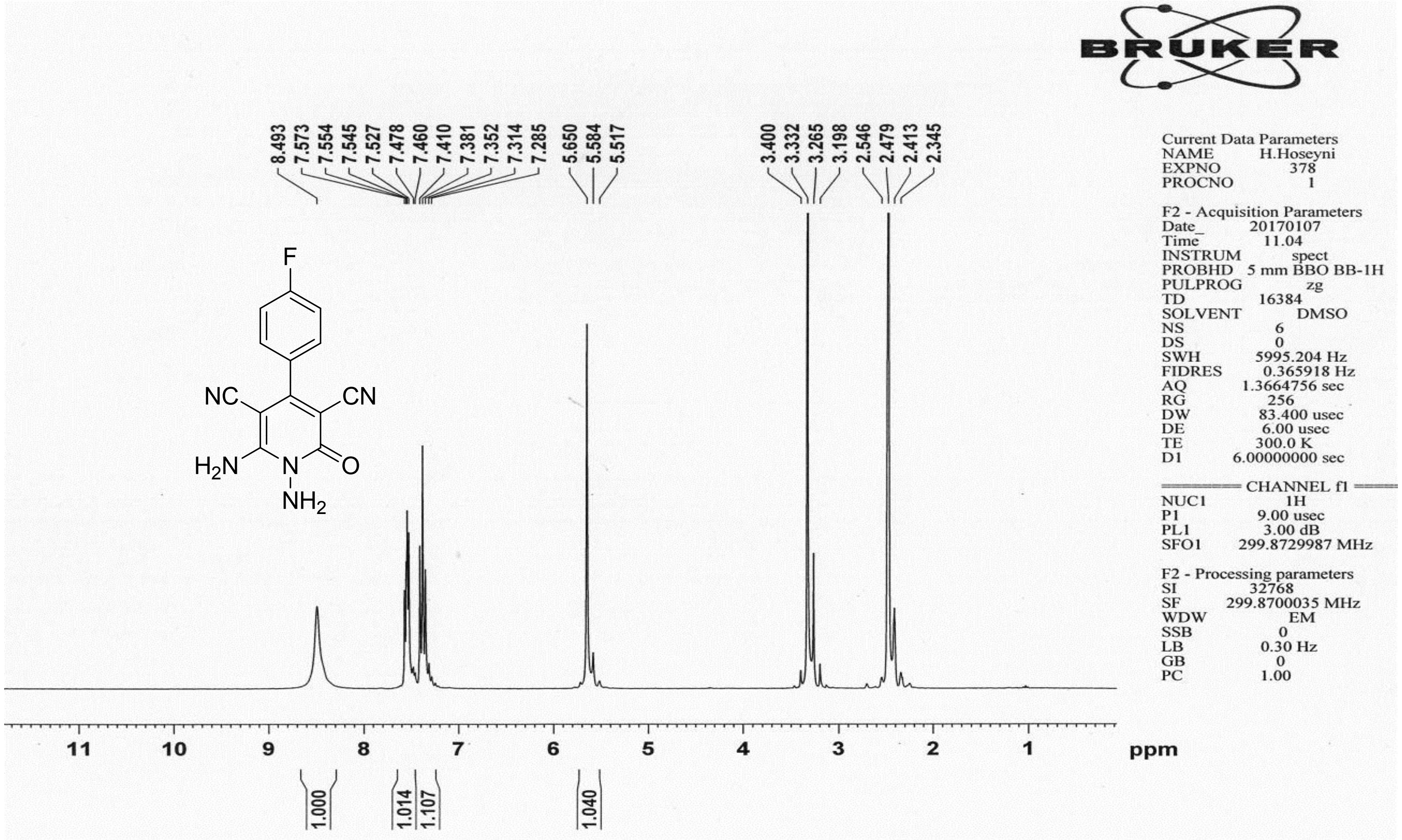
BRUKER
8.493
7.573
7.554
7.545
7.527
7.478
7.460
7.410
7.381
7.352
7.314
7.285
5.650
5.584
5.517
3.400
3.332
3.265
3.198
2.546
2.479
2.413
2.345
F
NC
CN
H2N
N
O
NH2
Current Data Parameters
NAME H.Hoseyni
EXPNO 378
PROCNO 1
F2 - Acquisition Parameters
Date_ 20170107
Time 11.04
INSTRUM spect
PROBHD 5 mm BBO BB-1H
PULPROG zg
TD 16384
SOLVENT DMSO
NS 6
DS 0
SWH 5995.204 Hz
FIDRES 0.365918 Hz
AQ 1.3664756 sec
RG 256
DW 83.400 usec
DE 6.00 usec
TE 300.0 K
D1 6.00000000 sec
CHANNEL f1
NUC1 1H
P1 9.00 usec
PL1 3.00 dB
SFO1 299.8729987 MHz
F2 - Processing parameters
SI 32768
SF 299.8700035 MHz
WDW EM
SSB 0
LB 0.30 Hz
GB 0
PC 1.00
11
10
9
8
7
6
5
4
3
2
1
ppm
1.000
1.014
1.107
1.040

^{1}H NMR of 4k

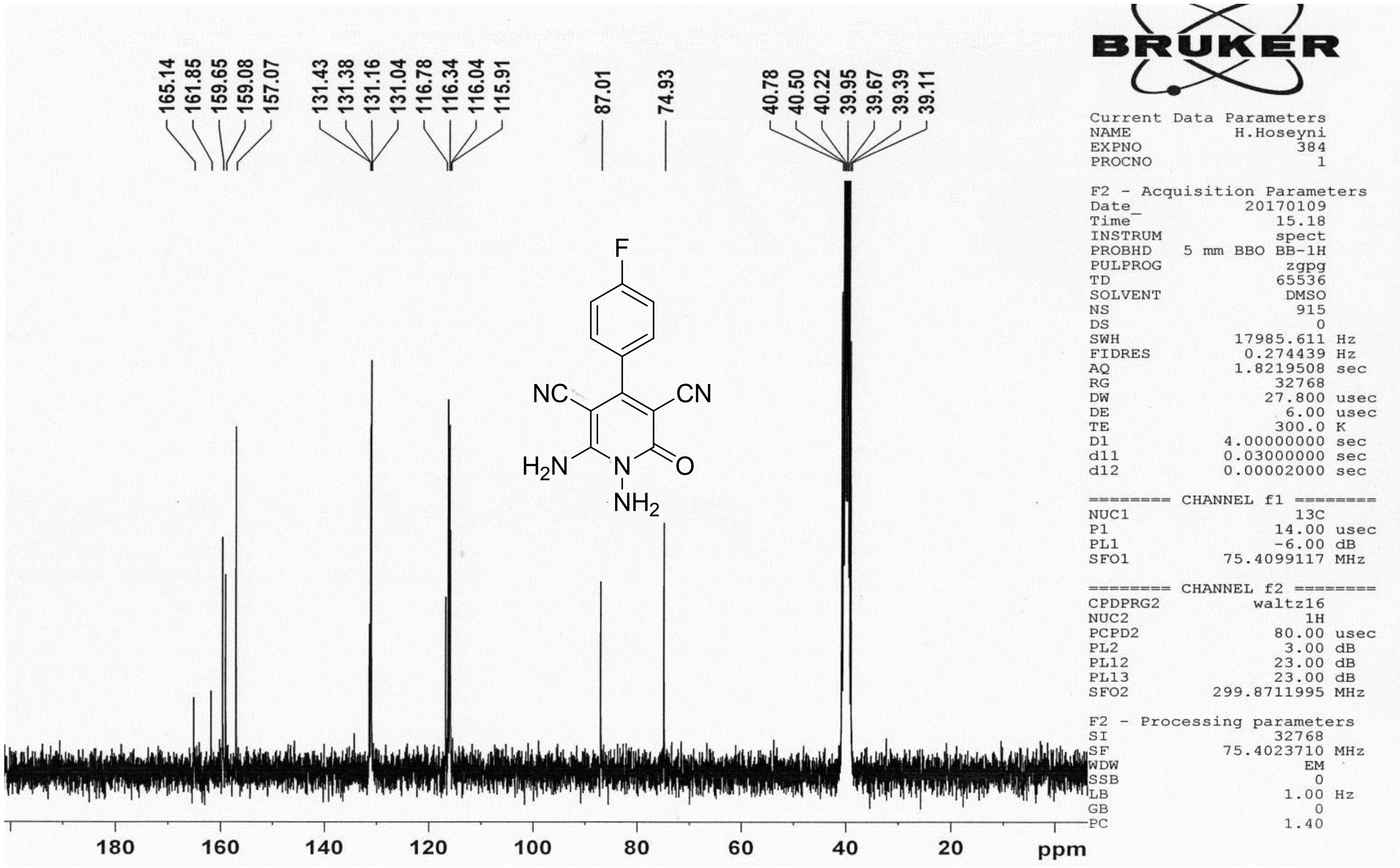
165.14
161.85
159.65
159.08
157.07
131.43
131.38
131.16
131.04
116.78
116.34
116.04
115.91
87.01
74.93
40.78
40.50
40.22
39.95
39.67
39.39
39.11
F
NC
CN
H2N
N
O
NH2
BRUKER
Current Data Parameters
NAME H.Hoseyni
EXPNO 384
PROCNO 1
F2 - Acquisition Parameters
Date_ 20170109
Time 15.18
INSTRUM spect
PROBHD 5 mm BBO BB-1H
PULPROG zgpg
TD 65536
SOLVENT DMSO
NS 915
DS 0
SWH 17985.611 Hz
FIDRES 0.274439 Hz
AQ 1.8219508 sec
RG 32768
DW 27.800 usec
DE 6.00 usec
TE 300.0 K
D1 4.00000000 sec
d11 0.03000000 sec
d12 0.00002000 sec
======== CHANNEL f1 ========
NUC1 13C
P1 14.00 usec
PL1 -6.00 dB
SFO1 75.4099117 MHz
======== CHANNEL f2 ========
CPDPRG2 waltz16
NUC2 1H
PCPD2 80.00 usec
PL2 3.00 dB
PL12 23.00 dB
PL13 23.00 dB
SFO2 299.8711995 MHz
F2 - Processing parameters
SI 32768
SF 75.4023710 MHz
WDW EM
SSB 0
LB 1.00 Hz
GB 0
PC 1.40
180
160
140
120
100
80
60
40
20
ppm

^{13}C NMR of 4k

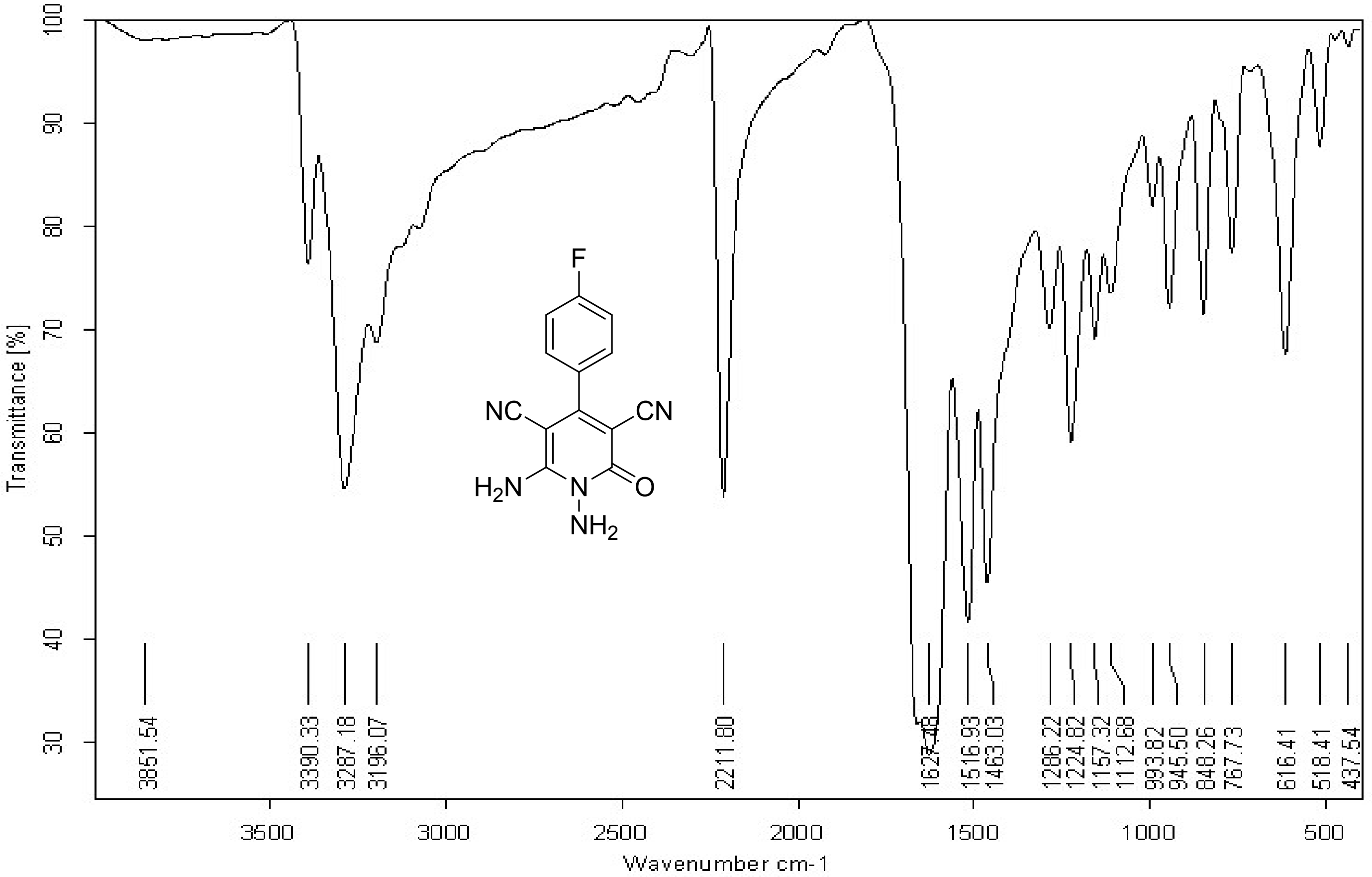
Transmittance [%]
Wavenumber cm-1
3851.54
3390.33
3287.18
3196.07
2211.80
1627.48
1516.93
1463.03
1286.22
1224.82
1157.32
1112.68
993.82
945.50
848.26
767.73
616.41
518.41
437.54
F
NC
CN
H2N
N
O
NH2

IR of 4k

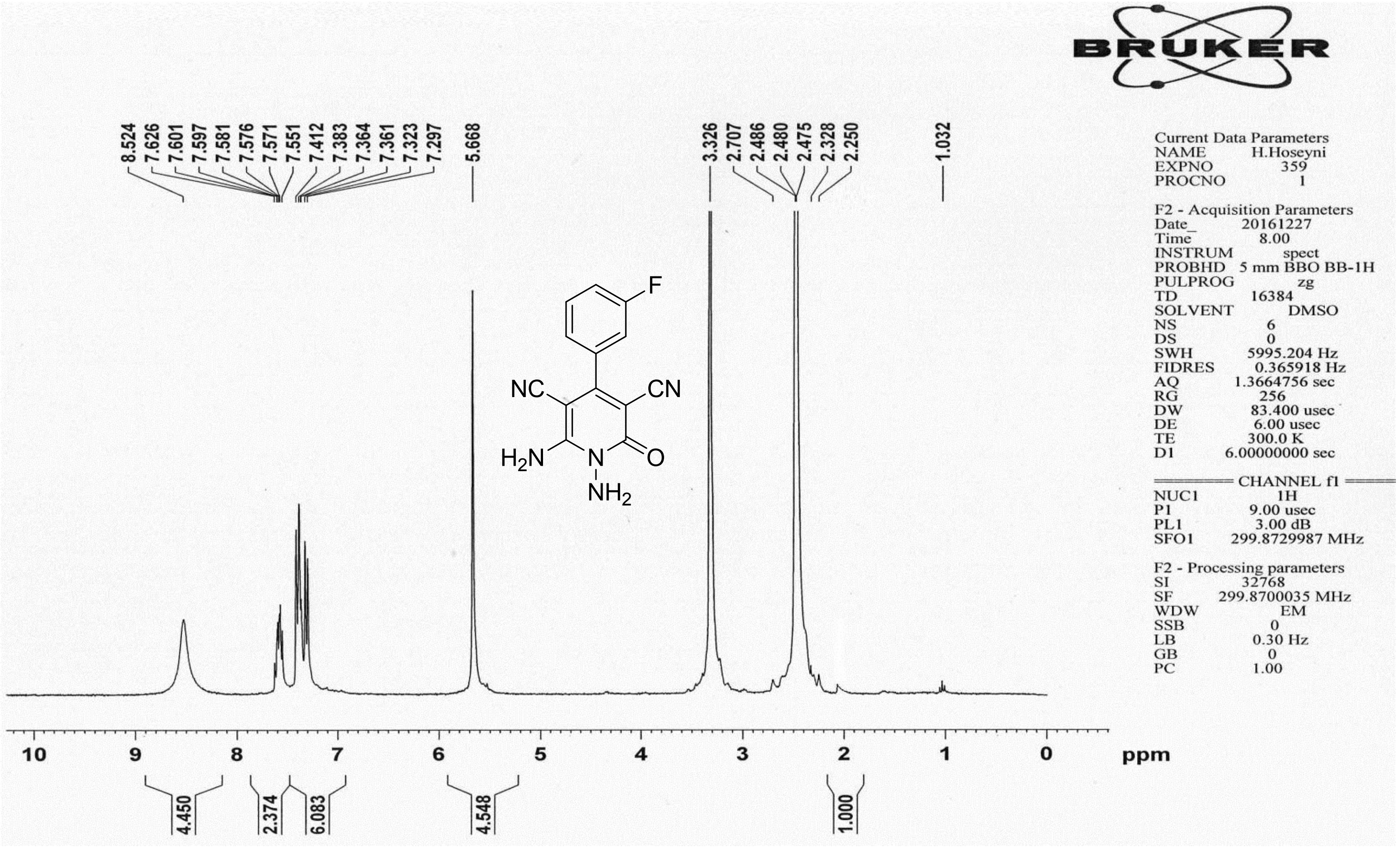
BRUKER
8.524
7.626
7.601
7.597
7.581
7.576
7.571
7.551
7.412
7.383
7.364
7.361
7.323
7.297
5.668
3.326
2.707
2.486
2.480
2.475
2.328
2.250
1.032
F
NC
CN
H2N
N
O
NH2
Current Data Parameters
NAME H.Hoseyni
EXPNO 359
PROCNO 1
F2 - Acquisition Parameters
Date_ 20161227
Time 8.00
INSTRUM spect
PROBHD 5 mm BBO BB-1H
PULPROG zg
TD 16384
SOLVENT DMSO
NS 6
DS 0
SWH 5995.204 Hz
FIDRES 0.365918 Hz
AQ 1.3664756 sec
RG 256
DW 83.400 usec
DE 6.00 usec
TE 300.0 K
D1 6.00000000 sec
CHANNEL f1
NUC1 1H
P1 9.00 usec
PL1 3.00 dB
SFO1 299.8729987 MHz
F2 - Processing parameters
SI 32768
SF 299.8700035 MHz
WDW EM
SSB 0
LB 0.30 Hz
GB 0
PC 1.00
10
9
8
7
6
5
4
3
2
1
0
ppm
4.450
2.374
6.083
4.548
1.000

^{1}H NMR of 4l

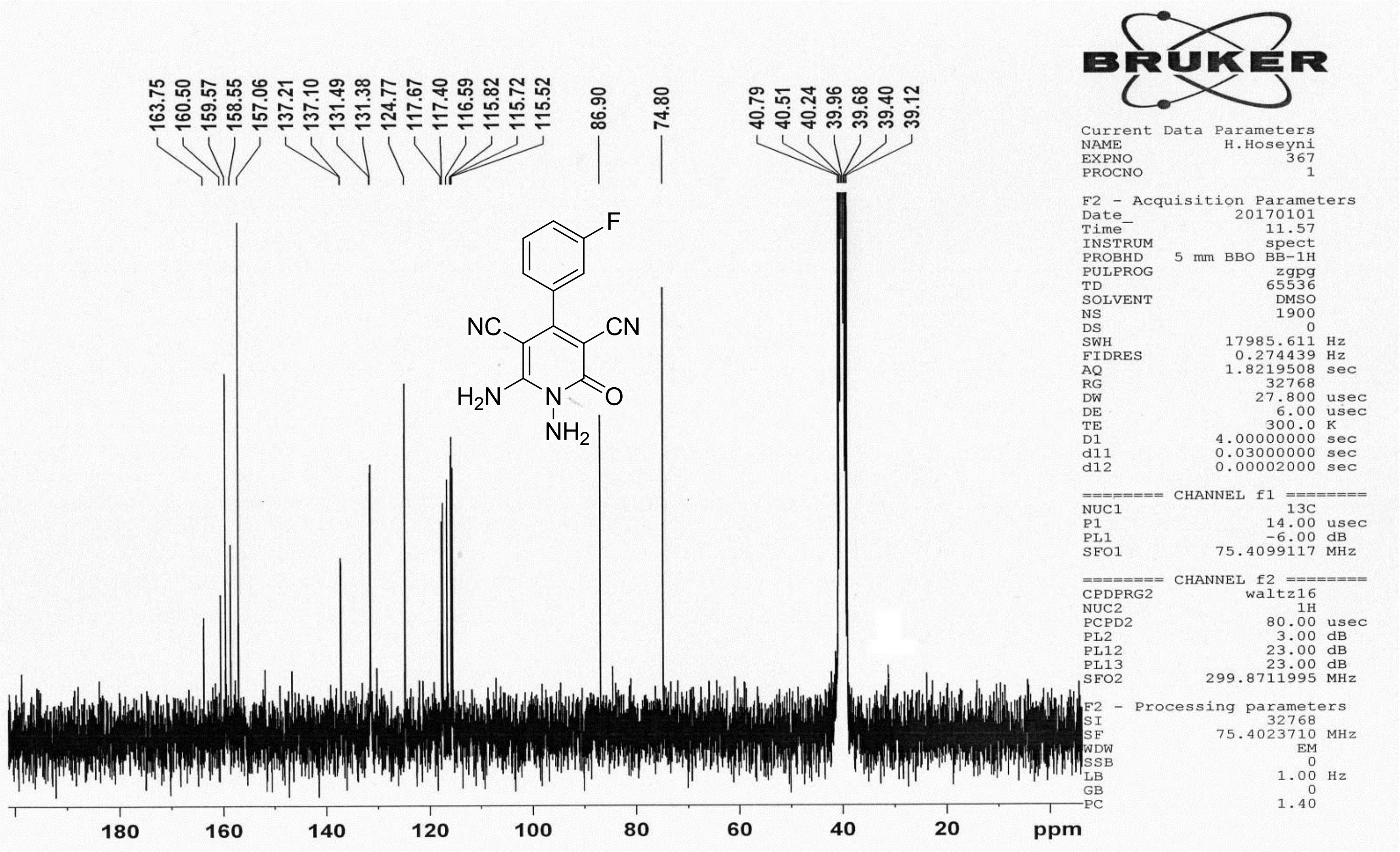
163.75
160.50
159.57
158.55
157.06
137.21
137.10
131.49
131.38
124.77
117.67
117.40
116.59
115.82
115.72
115.52
86.90
74.80
40.79
40.51
40.24
39.96
39.68
39.40
39.12
BRUKER
Current Data Parameters
NAME H.Hoseyni
EXPNO 367
PROCNO 1
F2 - Acquisition Parameters
Date_ 20170101
Time 11.57
INSTRUM spect
PROBHD 5 mm BBO BB-1H
PULPROG zgpg
TD 65536
SOLVENT DMSO
NS 1900
DS 0
SWH 17985.611 Hz
FIDRES 0.274439 Hz
AQ 1.8219508 sec
RG 32768
DW 27.800 usec
DE 6.00 usec
TE 300.0 K
D1 4.00000000 sec
d11 0.03000000 sec
d12 0.00002000 sec
======== CHANNEL f1 ========
NUC1 13C
P1 14.00 usec
PL1 -6.00 dB
SFO1 75.4099117 MHz
======== CHANNEL f2 ========
CPDPRG2 waltz16
NUC2 1H
PCPD2 80.00 usec
PL2 3.00 dB
PL12 23.00 dB
PL13 23.00 dB
SFO2 299.8711995 MHz
F2 - Processing parameters
SI 32768
SF 75.4023710 MHz
WDW EM
SSB 0
LB 1.00 Hz
GB 0
PC 1.40
180
160
140
120
100
80
60
40
20
ppm
F
NC
CN
H2N
N
O
NH2

^{13}C NMR of 4l

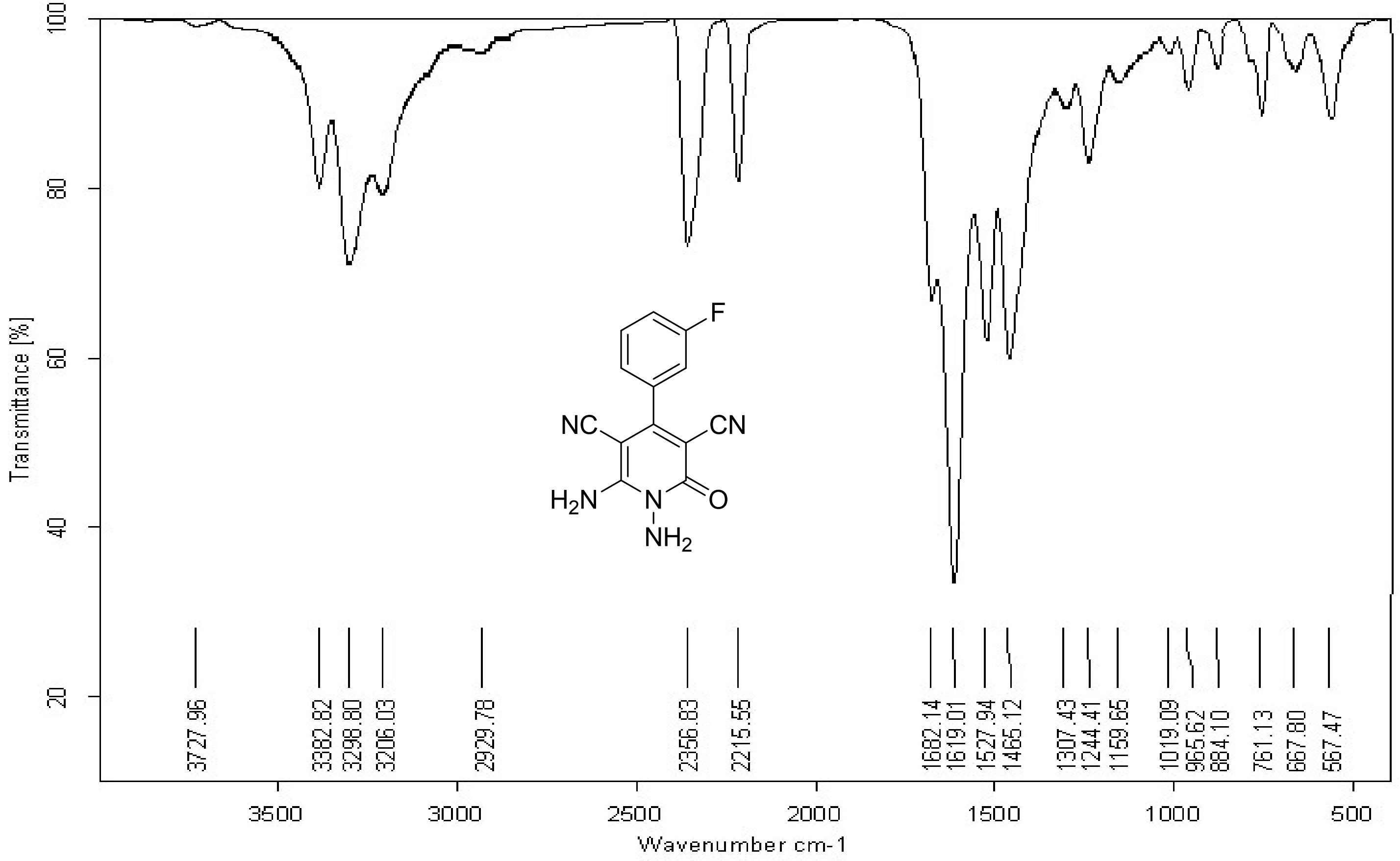
100
80
60
40
20
Transmittance [%]
F
NC
CN
H_2N
N
O
NH_2
3727.96
3382.82
3298.80
3206.03
2929.78
2356.83
2215.55
1682.14
1619.01
1527.94
1465.12
1307.43
1244.41
1159.65
1019.09
965.62
884.10
761.13
667.80
567.47
3500
3000
2500
2000
1500
1000
500
Wavenumber cm-1

IR of 4l

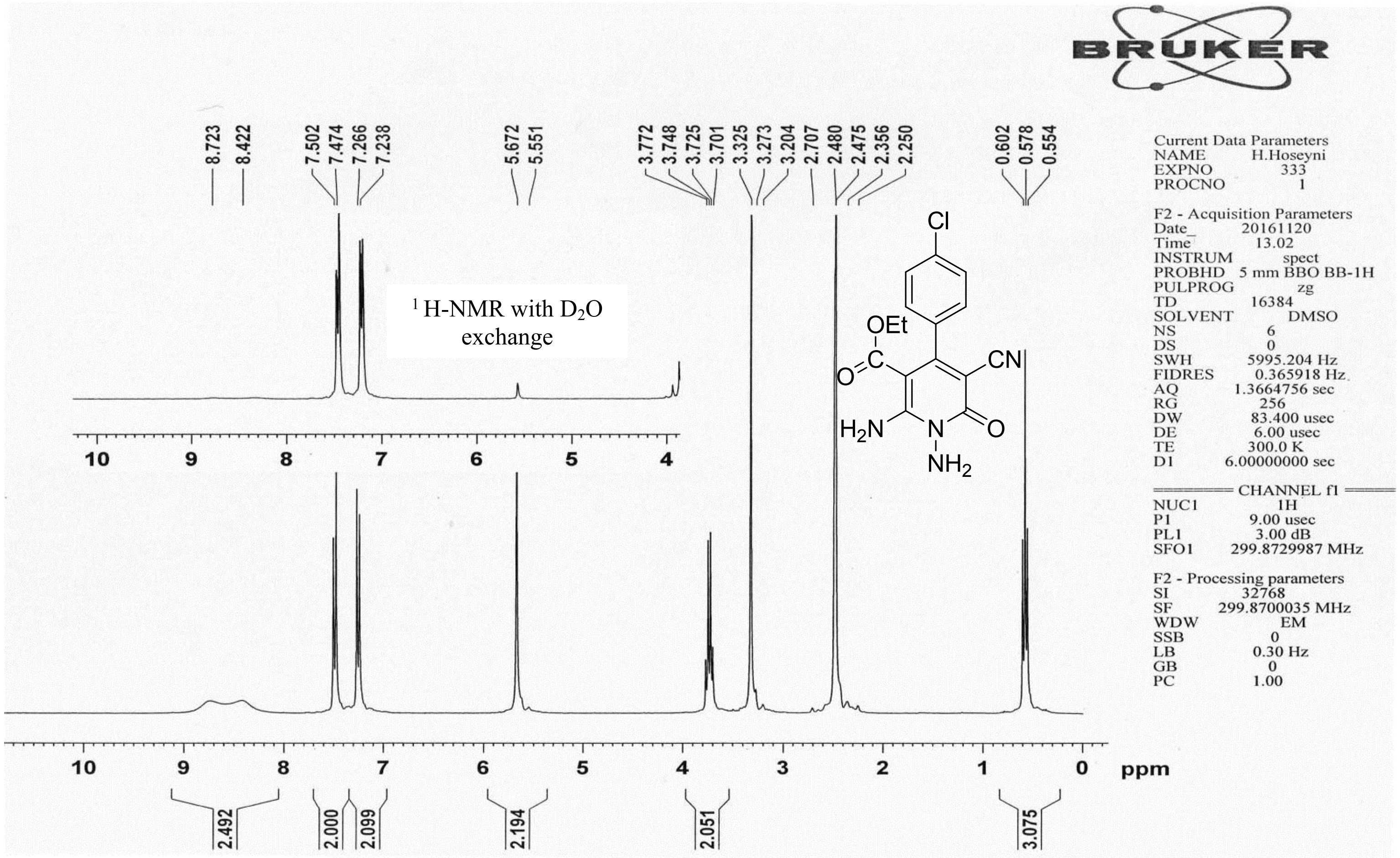
BRUKER
8.723
8.422
7.502
7.474
7.266
7.238
5.672
5.551
3.772
3.748
3.725
3.701
3.325
3.273
3.204
2.707
2.480
2.475
2.356
2.250
0.602
0.578
0.554
Current Data Parameters
NAME H.Hoseyni
EXPNO 333
PROCNO 1
F2 - Acquisition Parameters
Date_ 20161120
Time 13.02
INSTRUM spect
PROBHD 5 mm BBO BB-1H
PULPROG zg
TD 16384
SOLVENT DMSO
NS 6
DS 0
SWH 5995.204 Hz
FIDRES 0.365918 Hz
AQ 1.3664756 sec
RG 256
DW 83.400 usec
DE 6.00 usec
TE 300.0 K
D1 6.00000000 sec
CHANNEL f1
NUC1 1H
P1 9.00 usec
PL1 3.00 dB
SFO1 299.8729987 MHz
F2 - Processing parameters
SI 32768
SF 299.8700035 MHz
WDW EM
SSB 0
LB 0.30 Hz
GB 0
PC 1.00
1 H-NMR with D2O exchange
Cl
OEt
O
C
CN
H2N
N
O
NH2
ppm
2.492
2.000
2.099
2.194
2.051
3.075

^{1}H NMR of 5a

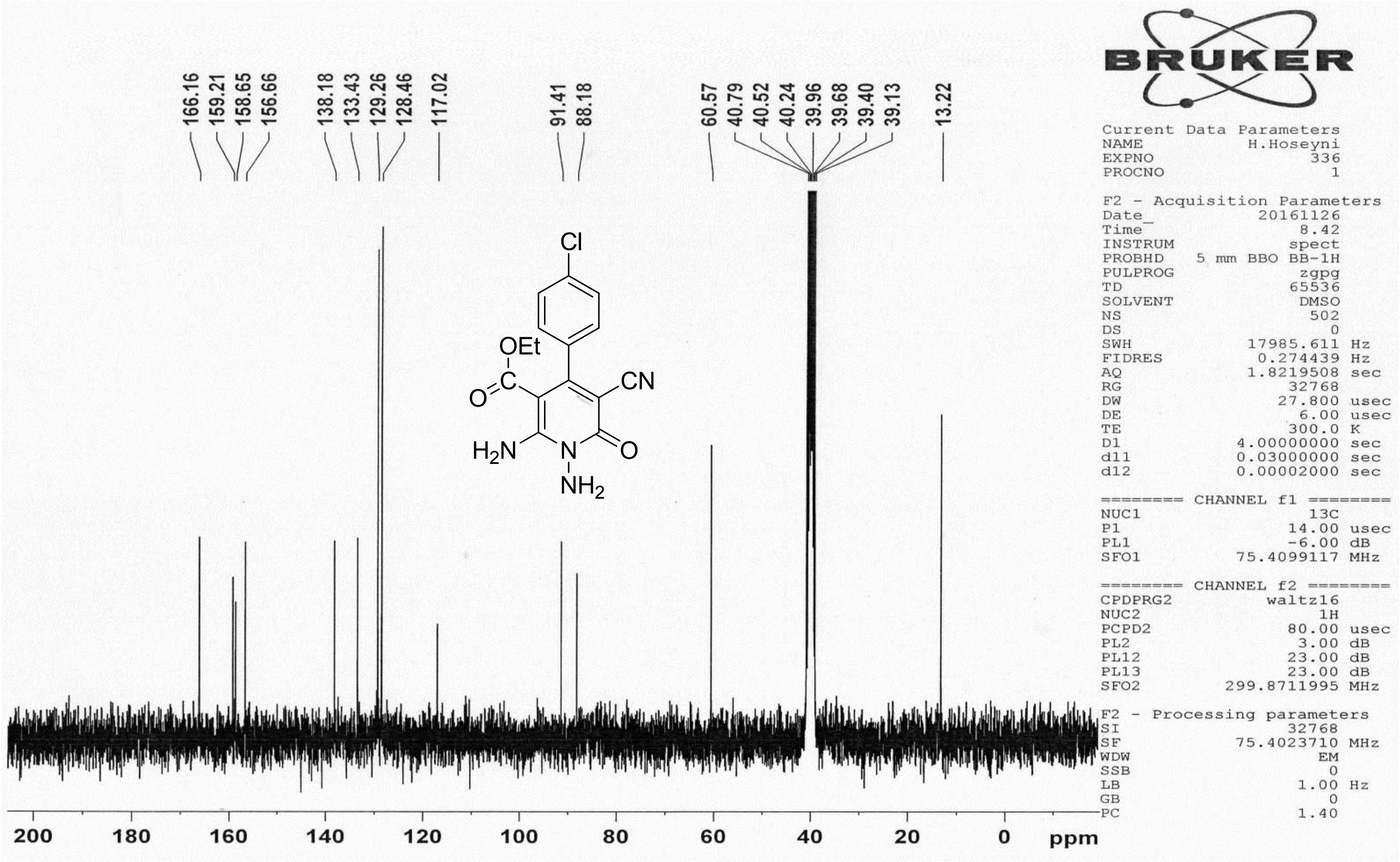

166.16
159.21
158.65
156.66
138.18
133.43
129.26
128.46
117.02
91.41
88.18
60.57
40.79
40.52
40.24
39.96
39.68
39.40
39.13
13.22
BRUKER
Cl
OEt
C
O
CN
H2N
N
O
NH2
Current Data Parameters
NAME H.Hoseyni
EXPNO 336
PROCNO 1
F2 - Acquisition Parameters
Date_ 20161126
Time 8.42
INSTRUM spect
PROBHD 5 mm BBO BB-1H
PULPROG zgpg
TD 65536
SOLVENT DMSO
NS 502
DS 0
SWH 17985.611 Hz
FIDRES 0.274439 Hz
AQ 1.8219508 sec
RG 32768
DW 27.800 usec
DE 6.00 usec
TE 300.0 K
D1 4.00000000 sec
d11 0.03000000 sec
d12 0.00002000 sec
======== CHANNEL f1 ========
NUC1 13C
P1 14.00 usec
PL1 -6.00 dB
SFO1 75.4099117 MHz
======== CHANNEL f2 ========
CPDPRG2 waltz16
NUC2 1H
PCPD2 80.00 usec
PL2 3.00 dB
PL12 23.00 dB
PL13 23.00 dB
SFO2 299.8711995 MHz
F2 - Processing parameters
SI 32768
SF 75.4023710 MHz
WDW EM
SSB 0
LB 1.00 Hz
GB 0
PC 1.40
200 180 160 140 120 100 80 60 40 20 0 ppm

^{13}C NMR of 5a

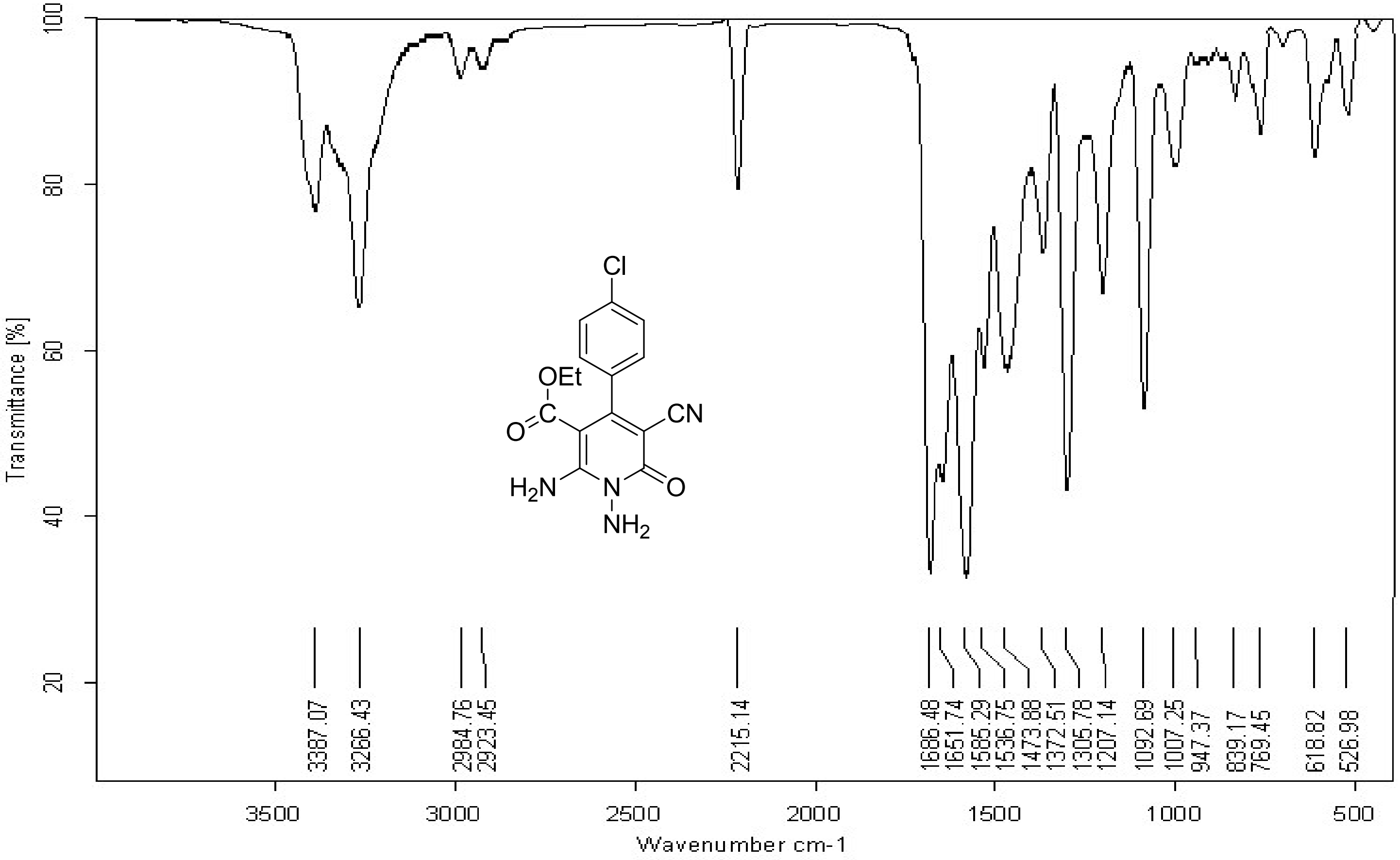
Transmittance [%]
100
80
60
40
20
Wavenumber cm-1
3500
3000
2500
2000
1500
1000
500
3387.07
3266.43
2984.76
2923.45
2215.14
1686.48
1651.74
1585.29
1536.75
1473.88
1372.51
1305.78
1207.14
1092.69
1007.25
947.37
839.17
769.45
618.82
526.98
Cl
OEt
C
O
CN
H2N
N
O
NH2

IR of 5a

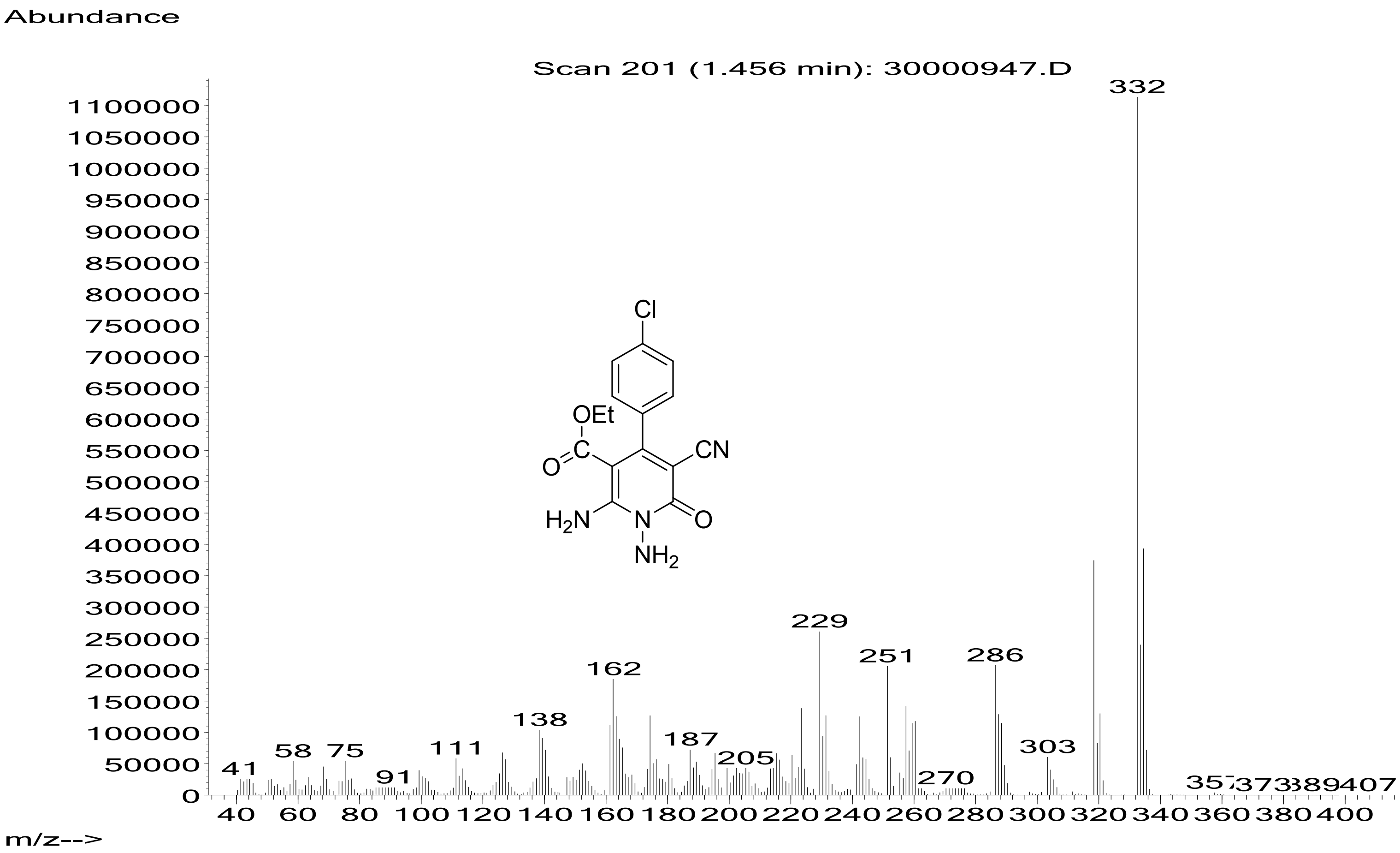
Abundance
Scan 201 (1.456 min): 30000947.D
332
229
251
286
162
138
187
205
303
270
41
58
75
91
111
357
373
389
407
1100000
1050000
1000000
950000
900000
850000
800000
750000
700000
650000
600000
550000
500000
450000
400000
350000
300000
250000
200000
150000
100000
50000
0
40 60 80 100 120 140 160 180 200 220 240 260 280 300 320 340 360 380 400
m/z-->
Cl
OEt
C
O
CN
H_2N
N
O
NH_2

MS of 5a

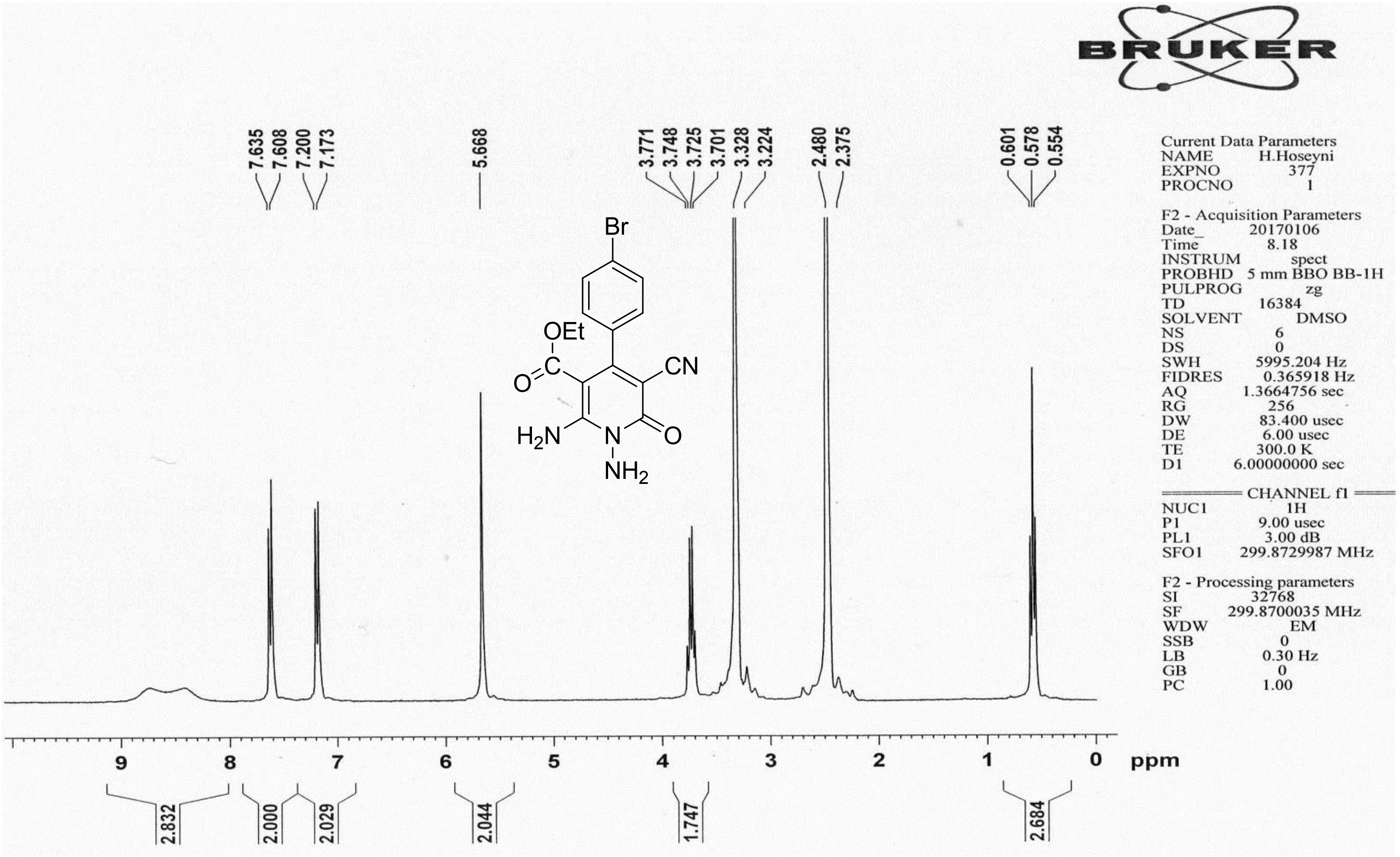
BRUKER
7.635
7.608
7.200
7.173
5.668
3.771
3.748
3.725
3.701
3.328
3.224
2.480
2.375
0.601
0.578
0.554
Br
OEt
CN
O
C
H2N
N
O
NH2
Current Data Parameters
NAME H.Hoseyni
EXPNO 377
PROCNO 1
F2 - Acquisition Parameters
Date_ 20170106
Time 8.18
INSTRUM spect
PROBHD 5 mm BBO BB-1H
PULPROG zg
TD 16384
SOLVENT DMSO
NS 6
DS 0
SWH 5995.204 Hz
FIDRES 0.365918 Hz
AQ 1.3664756 sec
RG 256
DW 83.400 usec
DE 6.00 usec
TE 300.0 K
D1 6.00000000 sec
CHANNEL f1
NUC1 1H
P1 9.00 usec
PL1 3.00 dB
SFO1 299.8729987 MHz
F2 - Processing parameters
SI 32768
SF 299.8700035 MHz
WDW EM
SSB 0
LB 0.30 Hz
GB 0
PC 1.00
9 8 7 6 5 4 3 2 1 0 ppm
2.832
2.000
2.029
2.044
1.747
2.684

^{1}H NMR of 5b

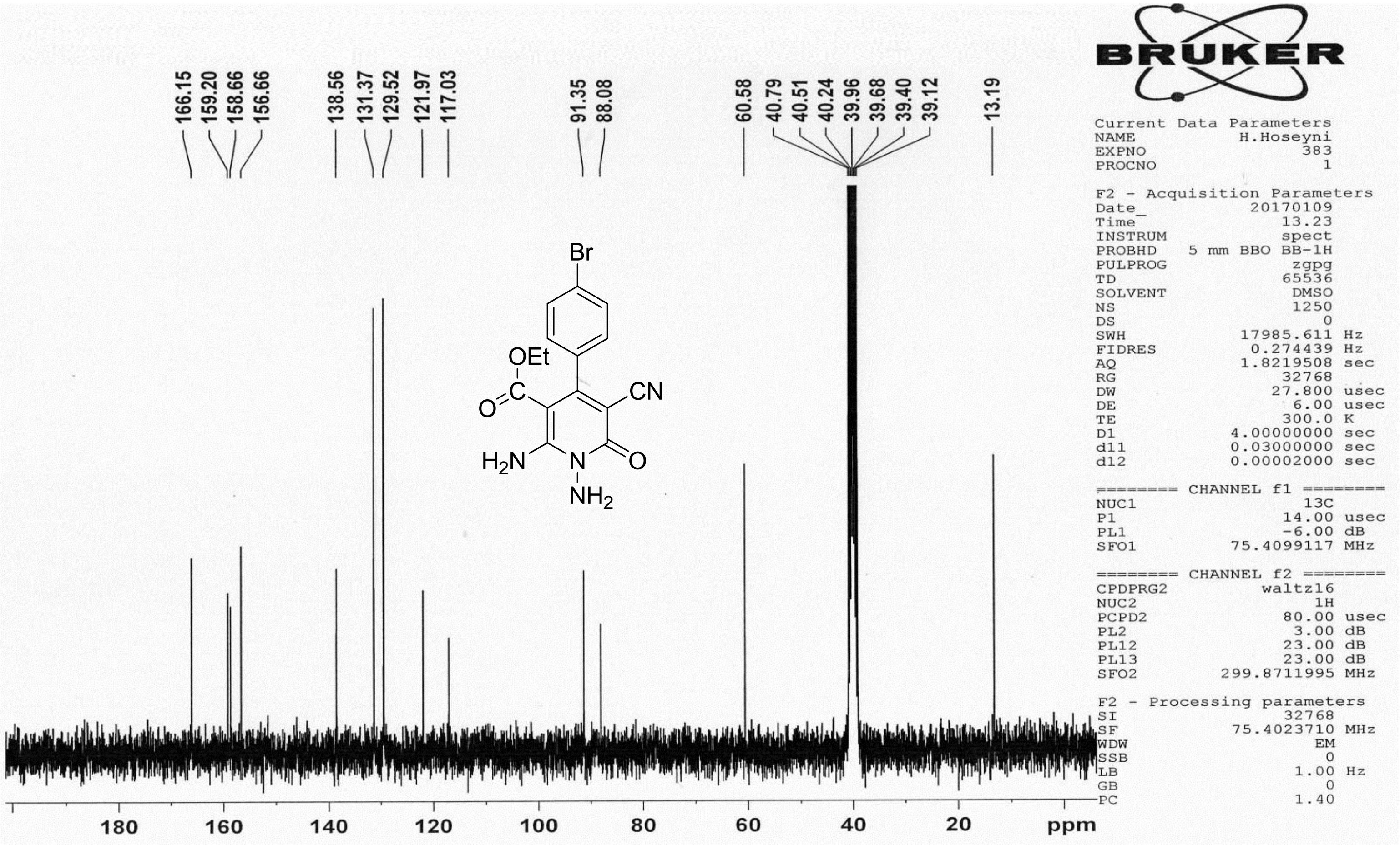

166.15
159.20
158.66
156.66
138.56
131.37
129.52
121.97
117.03
91.35
88.08
60.58
40.79
40.51
40.24
39.96
39.68
39.40
39.12
13.19
Br
OEt
CN
O
C
H2N
N
O
NH2
BRUKER
Current Data Parameters
NAME H.Hoseyni
EXPNO 383
PROCNO 1
F2 - Acquisition Parameters
Date_ 20170109
Time 13.23
INSTRUM spect
PROBHD 5 mm BBO BB-1H
PULPROG zgpg
TD 65536
SOLVENT DMSO
NS 1250
DS 0
SWH 17985.611 Hz
FIDRES 0.274439 Hz
AQ 1.8219508 sec
RG 32768
DW 27.800 usec
DE 6.00 usec
TE 300.0 K
D1 4.00000000 sec
d11 0.03000000 sec
d12 0.00002000 sec
======== CHANNEL f1 ========
NUC1 13C
P1 14.00 usec
PL1 -6.00 dB
SFO1 75.4099117 MHz
======== CHANNEL f2 ========
CPDPRG2 waltz16
NUC2 1H
PCPD2 80.00 usec
PL2 3.00 dB
PL12 23.00 dB
PL13 23.00 dB
SFO2 299.8711995 MHz
F2 - Processing parameters
SI 32768
SF 75.4023710 MHz
WDW EM
SSB 0
LB 1.00 Hz
GB 0
PC 1.40
180
160
140
120
100
80
60
40
20
ppm

^{13}C NMR of 5b

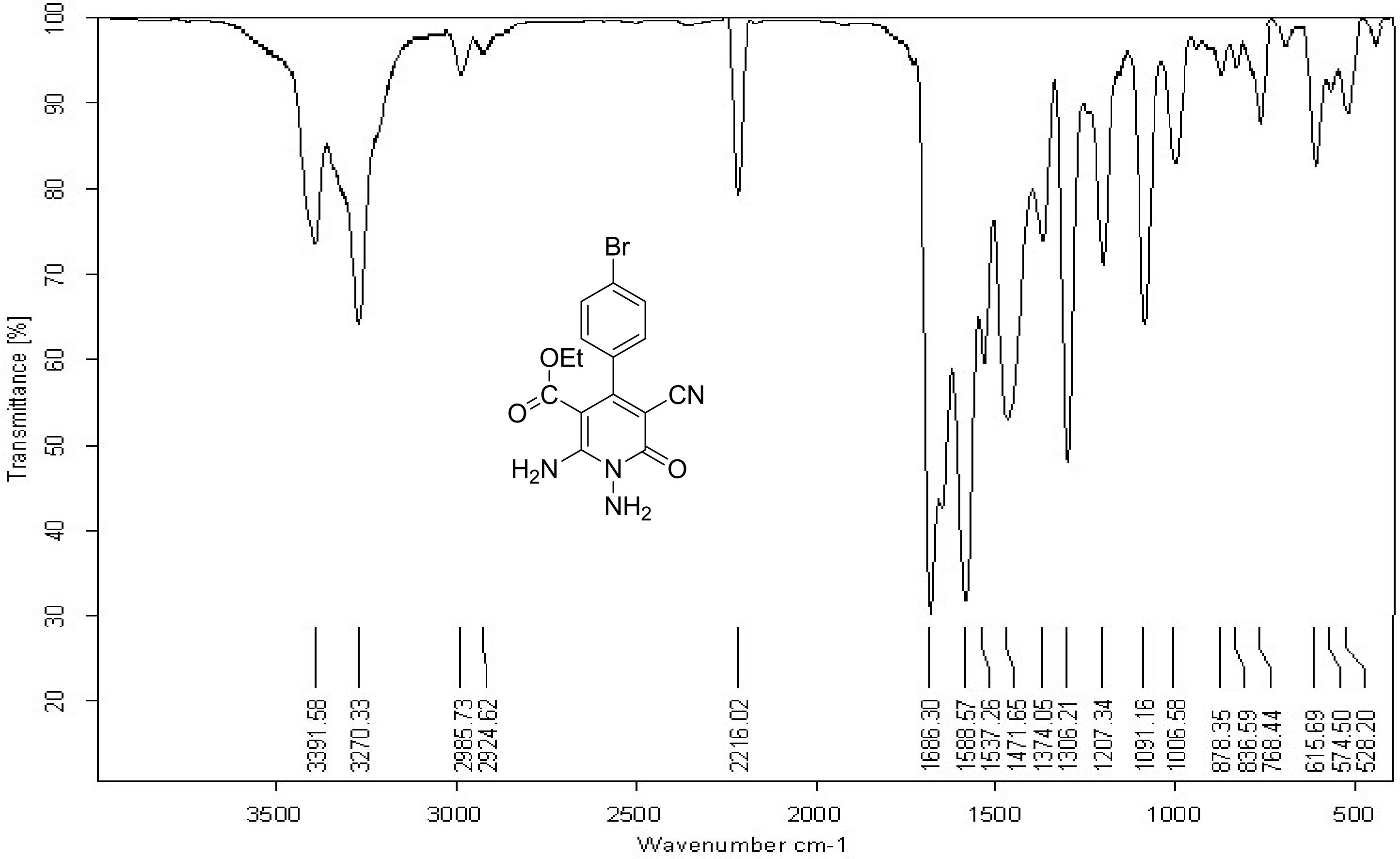

Transmittance [%]
100
90
80
70
60
50
40
30
20
3391.58
3270.33
2985.73
2924.62
2216.02
1686.30
1588.57
1537.26
1471.65
1374.05
1306.21
1207.34
1091.16
1006.58
878.35
836.59
768.44
615.69
574.50
528.20
3500
3000
2500
2000
1500
1000
500
Wavenumber cm-1
Br
OEt
CN
H2N
NH2

IR of 5b

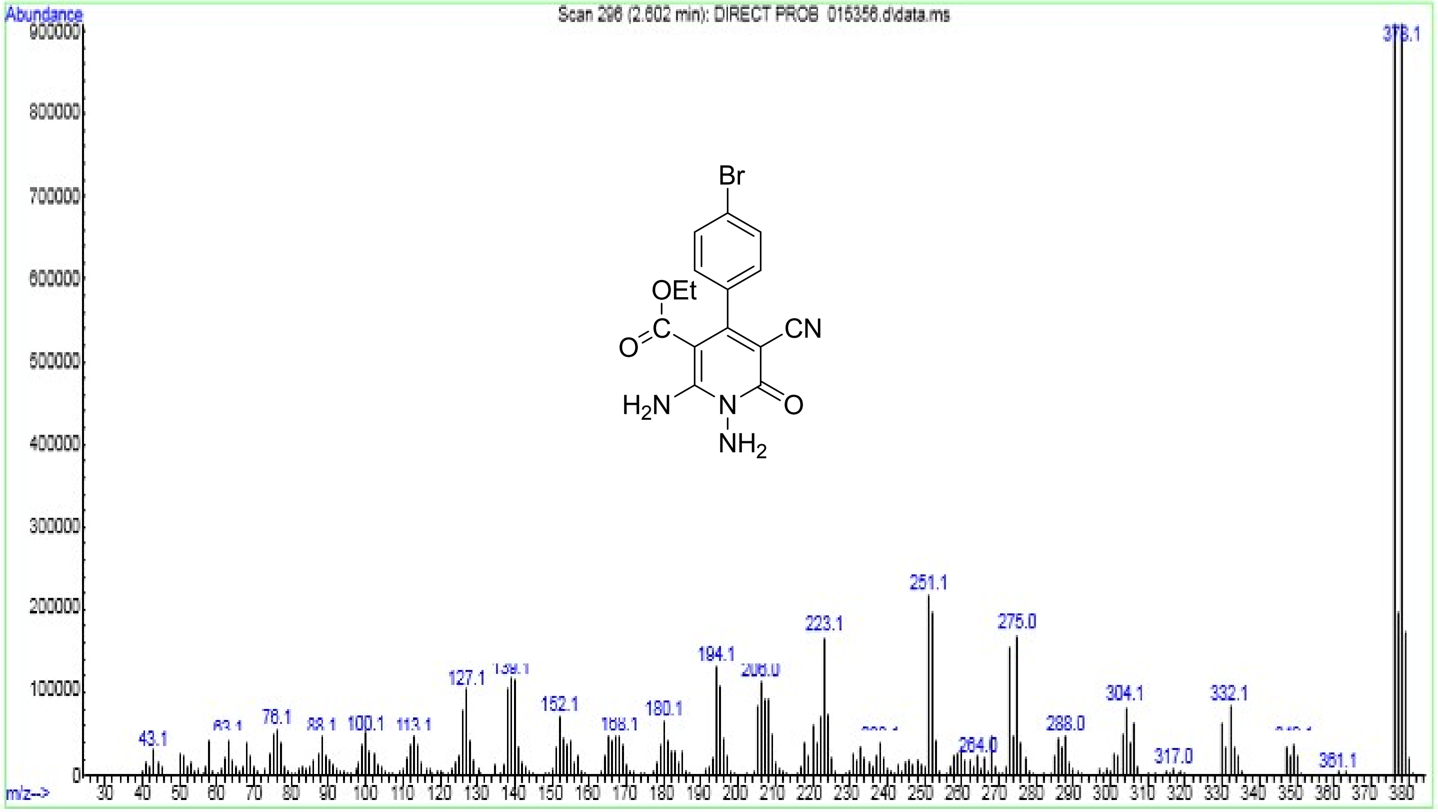
Abundance
Scan 298 (2.602 min): DIRECT PROB 015356.d\data.ms
900000
800000
700000
600000
500000
400000
300000
200000
100000
0
m/z-->
43.1
76.1
100.1
127.1
152.1
180.1
194.1
223.1
251.1
275.0
288.0
304.1
317.0
332.1
361.1
Br
OEt
CN
O
H2N
N
NH2

MS of 5b

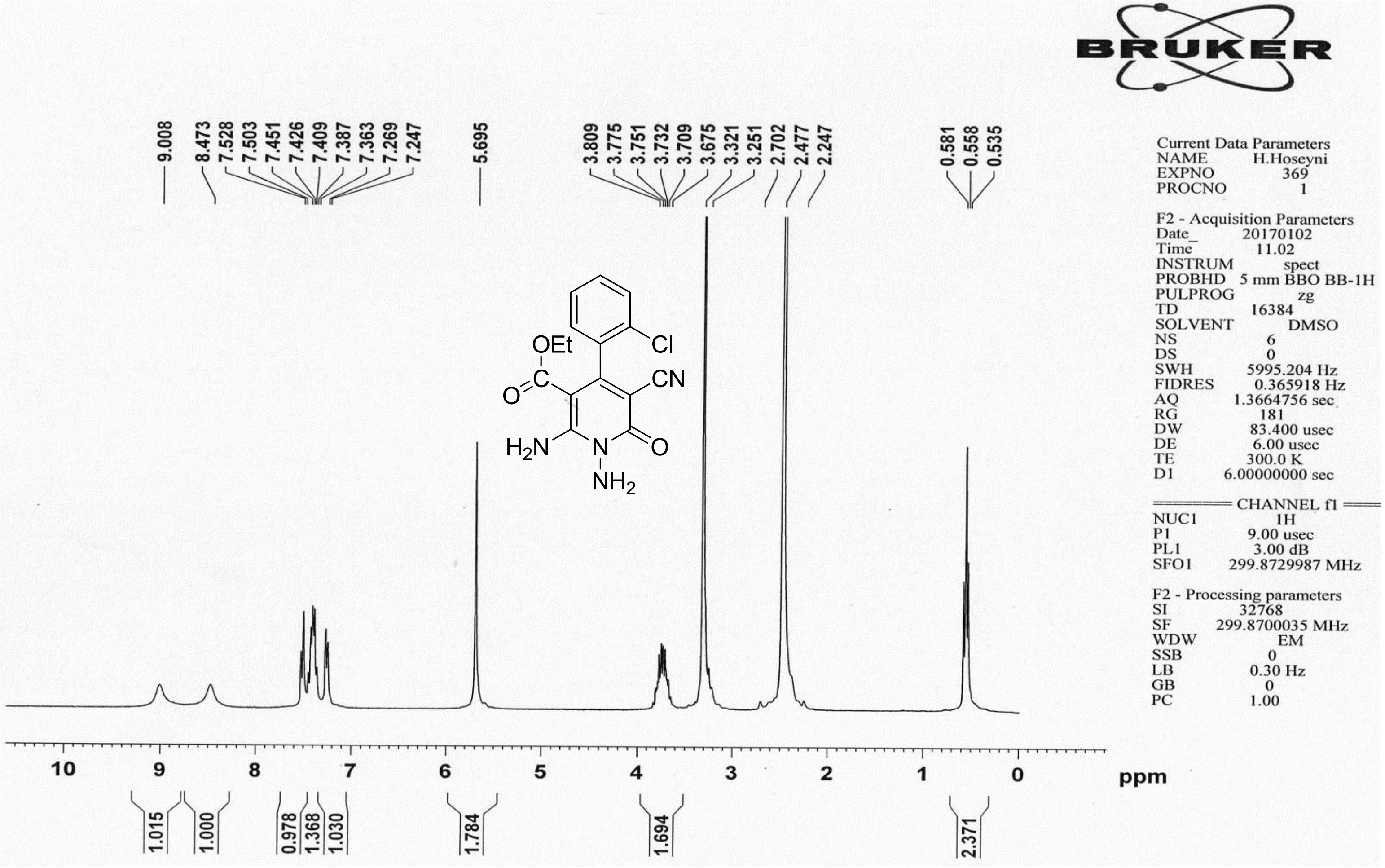
BRUKER
9.008
8.473
7.528
7.503
7.451
7.426
7.409
7.387
7.363
7.269
7.247
5.695
3.809
3.775
3.751
3.732
3.709
3.675
3.321
3.251
2.702
2.477
2.247
0.581
0.558
0.535
Current Data Parameters
NAME H.Hoseyni
EXPNO 369
PROCNO 1
F2 - Acquisition Parameters
Date_ 20170102
Time 11.02
INSTRUM spect
PROBHD 5 mm BBO BB-1H
PULPROG zg
TD 16384
SOLVENT DMSO
NS 6
DS 0
SWH 5995.204 Hz
FIDRES 0.365918 Hz
AQ 1.3664756 sec
RG 181
DW 83.400 usec
DE 6.00 usec
TE 300.0 K
D1 6.00000000 sec
CHANNEL f1
NUC1 1H
P1 9.00 usec
PL1 3.00 dB
SFO1 299.8729987 MHz
F2 - Processing parameters
SI 32768
SF 299.8700035 MHz
WDW EM
SSB 0
LB 0.30 Hz
GB 0
PC 1.00
OEt
Cl
CN
O
H2N
N
O
NH2
10 9 8 7 6 5 4 3 2 1 0
ppm
1.015
1.000
0.978
1.368
1.030
1.784
1.694
2.371

^{1}H NMR of 5c

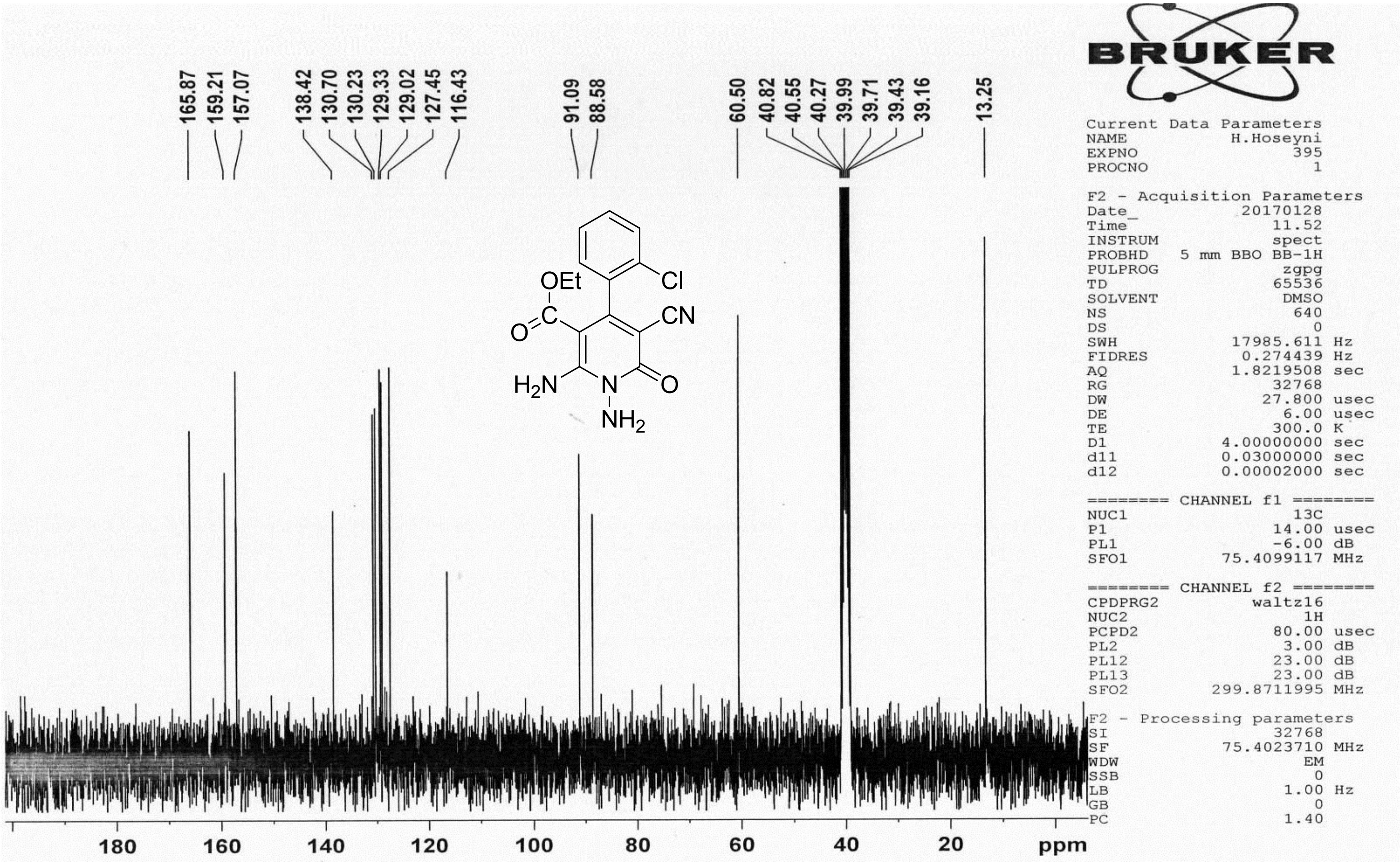
165.87
159.21
157.07
138.42
130.70
130.23
129.33
129.02
127.45
116.43
91.09
88.58
60.50
40.82
40.55
40.27
39.99
39.71
39.43
39.16
13.25
BRUKER
OEt
Cl
CN
O
H2N
N
O
NH2
Current Data Parameters
NAME H.Hoseyni
EXPNO 395
PROCNO 1
F2 - Acquisition Parameters
Date_ 20170128
Time 11.52
INSTRUM spect
PROBHD 5 mm BBO BB-1H
PULPROG zgpg
TD 65536
SOLVENT DMSO
NS 640
DS 0
SWH 17985.611 Hz
FIDRES 0.274439 Hz
AQ 1.8219508 sec
RG 32768
DW 27.800 usec
DE 6.00 usec
TE 300.0 K
D1 4.00000000 sec
d11 0.03000000 sec
d12 0.00002000 sec
======== CHANNEL f1 ========
NUC1 13C
P1 14.00 usec
PL1 -6.00 dB
SFO1 75.4099117 MHz
======== CHANNEL f2 ========
CPDPRG2 waltz16
NUC2 1H
PCPD2 80.00 usec
PL2 3.00 dB
PL12 23.00 dB
PL13 23.00 dB
SFO2 299.8711995 MHz
F2 - Processing parameters
SI 32768
SF 75.4023710 MHz
WDW EM
SSB 0
LB 1.00 Hz
GB 0
PC 1.40
180
160
140
120
100
80
60
40
20
ppm

^{13}C NMR of 5c

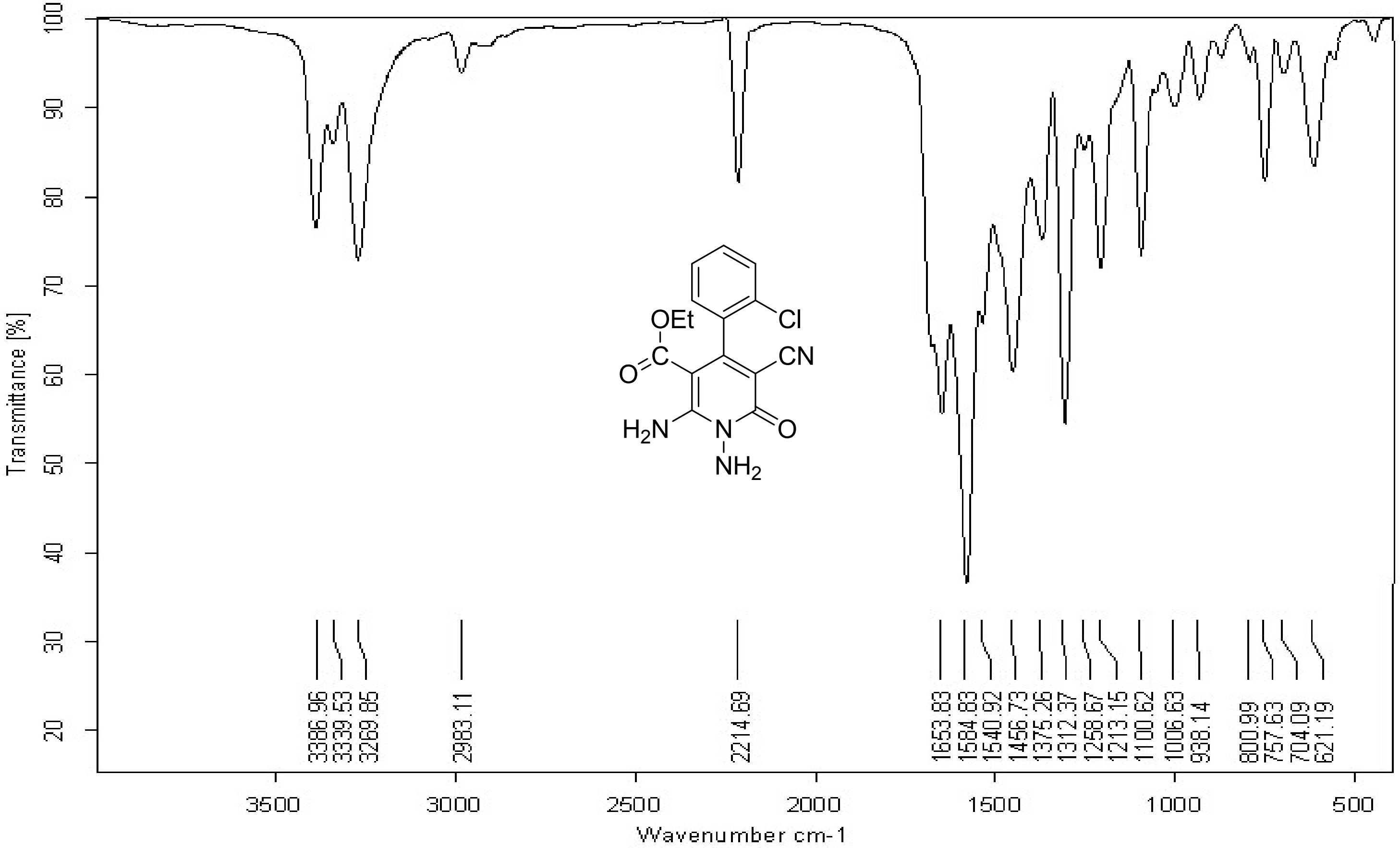
Transmittance [%]
100
90
80
70
60
50
40
30
20
Wavenumber cm-1
3500
3000
2500
2000
1500
1000
500
3386.96
3339.53
3269.85
2983.11
2214.69
1653.83
1584.83
1540.92
1456.73
1375.26
1312.37
1258.67
1213.15
1100.62
1006.63
938.14
800.99
757.63
704.09
621.19
OEt
Cl
CN
C
O
H_2N
N
O
NH_2

IR of 5c

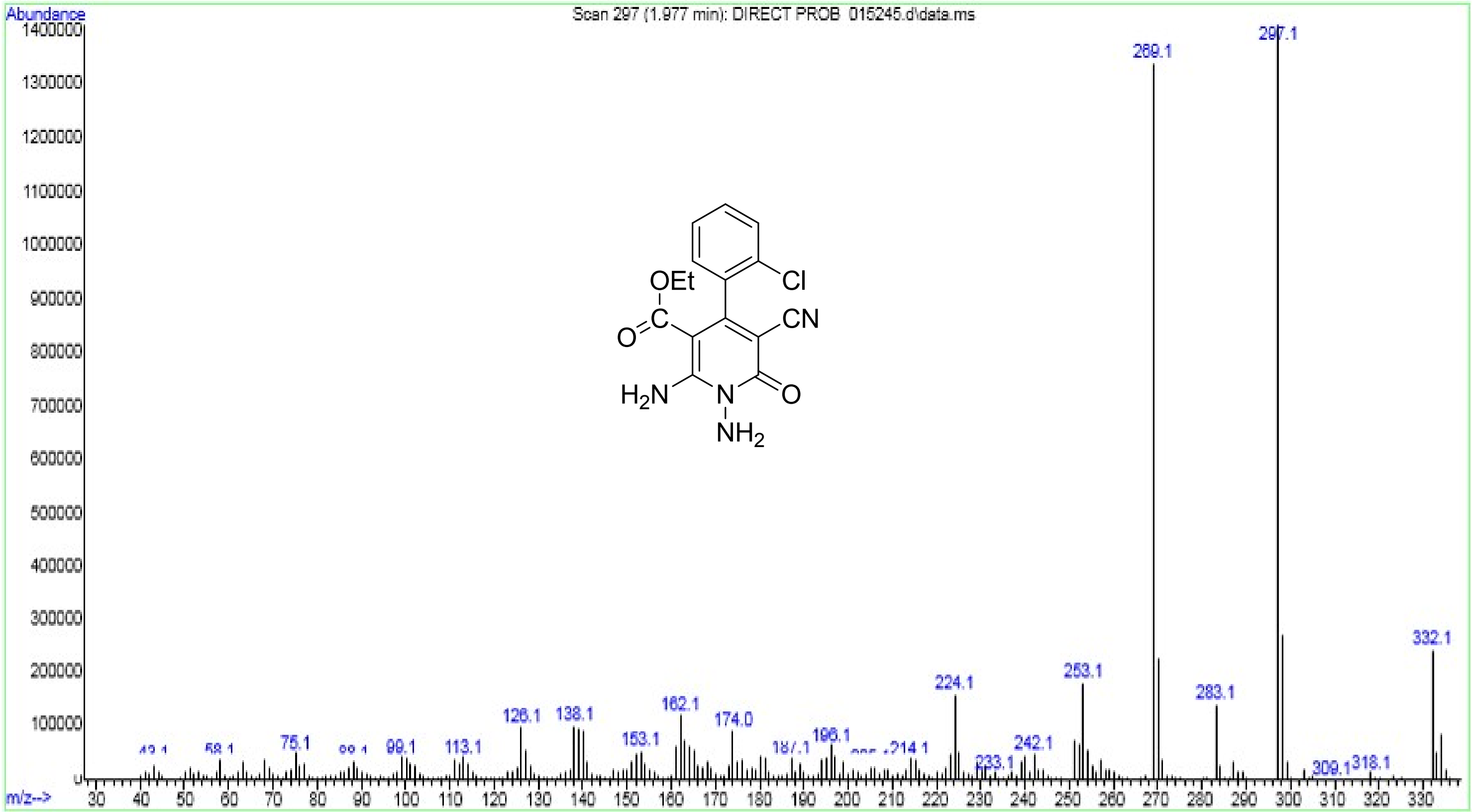
Abundance
Scan 297 (1.977 min): DIRECT PROB 015245.d\data.ms
1400000
1300000
1200000
1100000
1000000
900000
800000
700000
600000
500000
400000
300000
200000
100000
0
m/z-->
269.1
297.1
332.1
253.1
283.1
224.1
162.1
174.0
126.1
138.1
153.1
196.1
242.1
75.1
99.1
113.1
58.1
309.1
318.1
233.1
214.1
187.1
OEt
C
O
Cl
CN
H2N
N
O
NH2

MS of 5c

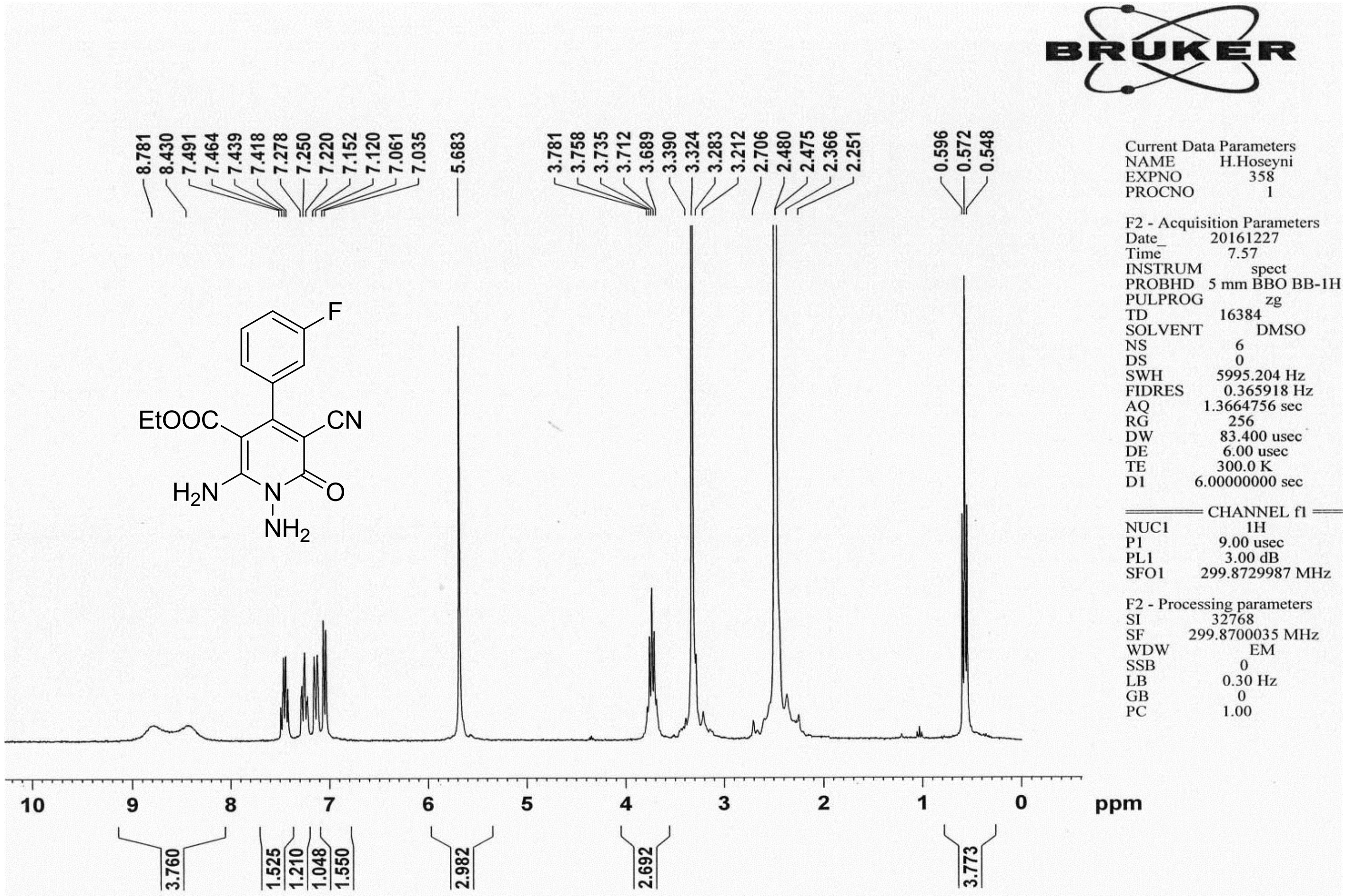
BRUKER
8.781
8.430
7.491
7.464
7.439
7.418
7.278
7.250
7.220
7.152
7.120
7.061
7.035
5.683
3.781
3.758
3.735
3.712
3.689
3.390
3.324
3.283
3.212
2.706
2.480
2.475
2.366
2.251
0.596
0.572
0.548
F
EtOOC
CN
H2N
N
O
NH2
Current Data Parameters
NAME H.Hoseyni
EXPNO 358
PROCNO 1
F2 - Acquisition Parameters
Date_ 20161227
Time 7.57
INSTRUM spect
PROBHD 5 mm BBO BB-1H
PULPROG zg
TD 16384
SOLVENT DMSO
NS 6
DS 0
SWH 5995.204 Hz
FIDRES 0.365918 Hz
AQ 1.3664756 sec
RG 256
DW 83.400 usec
DE 6.00 usec
TE 300.0 K
D1 6.00000000 sec
CHANNEL f1
NUC1 1H
P1 9.00 usec
PL1 3.00 dB
SFO1 299.8729987 MHz
F2 - Processing parameters
SI 32768
SF 299.8700035 MHz
WDW EM
SSB 0
LB 0.30 Hz
GB 0
PC 1.00
10
9
8
7
6
5
4
3
2
1
0
ppm
3.760
1.525
1.210
1.048
1.550
2.982
2.692
3.773

^{1}H NMR of 5d

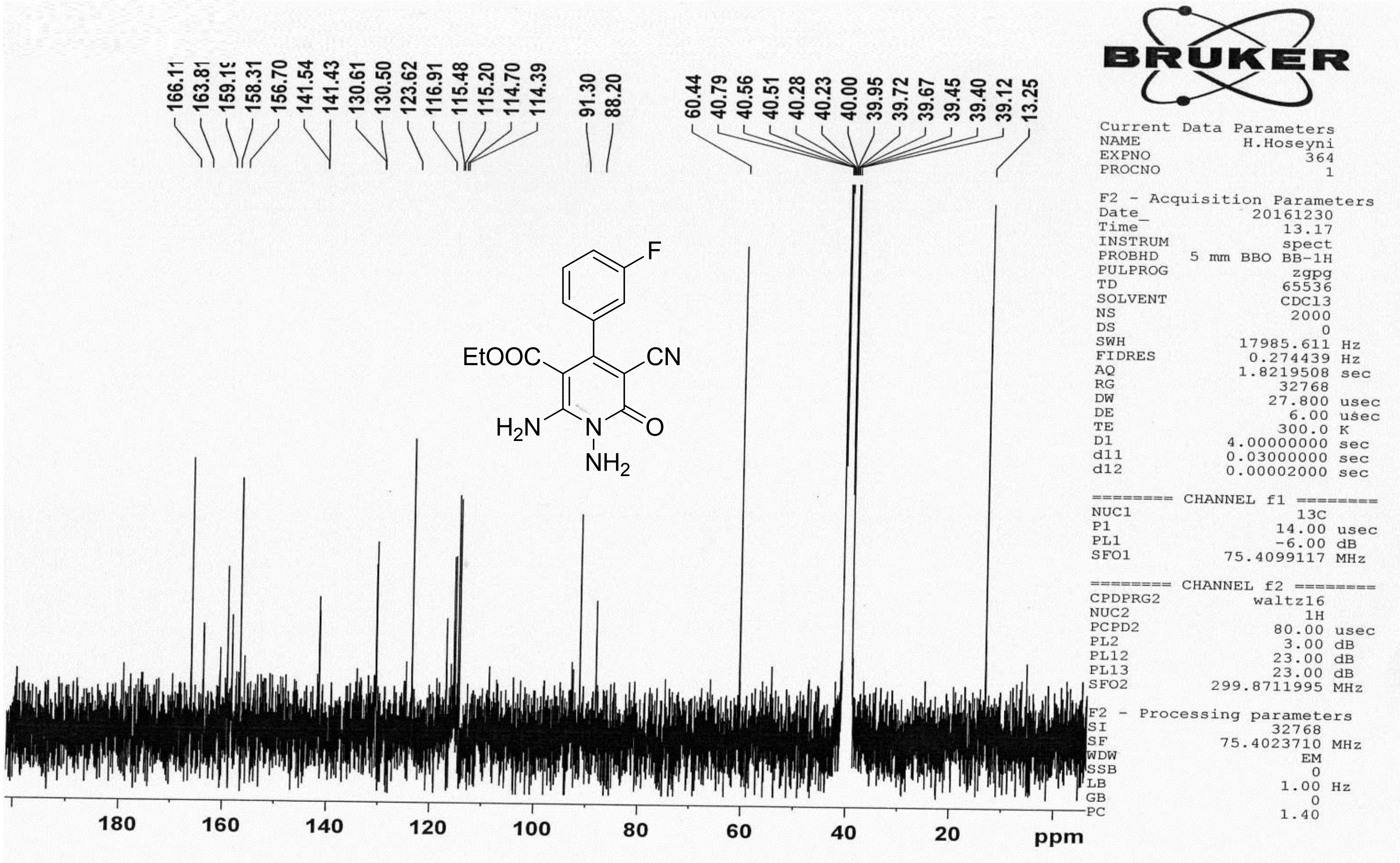

166.11
163.81
159.19
158.31
156.70
141.54
141.43
130.61
130.50
123.62
116.91
115.48
115.20
114.70
114.39
91.30
88.20
60.44
40.79
40.56
40.51
40.28
40.23
40.00
39.95
39.72
39.67
39.45
39.40
39.12
13.25
BRUKER
Current Data Parameters
NAME H.Hoseyni
EXPNO 364
PROCNO 1
F2 - Acquisition Parameters
Date_ 20161230
Time 13.17
INSTRUM spect
PROBHD 5 mm BBO BB-1H
PULPROG zgpg
TD 65536
SOLVENT CDCl3
NS 2000
DS 0
SWH 17985.611 Hz
FIDRES 0.274439 Hz
AQ 1.8219508 sec
RG 32768
DW 27.800 usec
DE 6.00 usec
TE 300.0 K
D1 4.00000000 sec
d11 0.03000000 sec
d12 0.00002000 sec
======== CHANNEL f1 ========
NUC1 13C
P1 14.00 usec
PL1 -6.00 dB
SFO1 75.4099117 MHz
======== CHANNEL f2 ========
CPDPRG2 waltz16
NUC2 1H
PCPD2 80.00 usec
PL2 3.00 dB
PL12 23.00 dB
PL13 23.00 dB
SFO2 299.8711995 MHz
F2 - Processing parameters
SI 32768
SF 75.4023710 MHz
WDW EM
SSB 0
LB 1.00 Hz
GB 0
PC 1.40
F
EtOOC
CN
H2N
N
O
NH2
180
160
140
120
100
80
60
40
20
ppm

^{13}C NMR of 5d

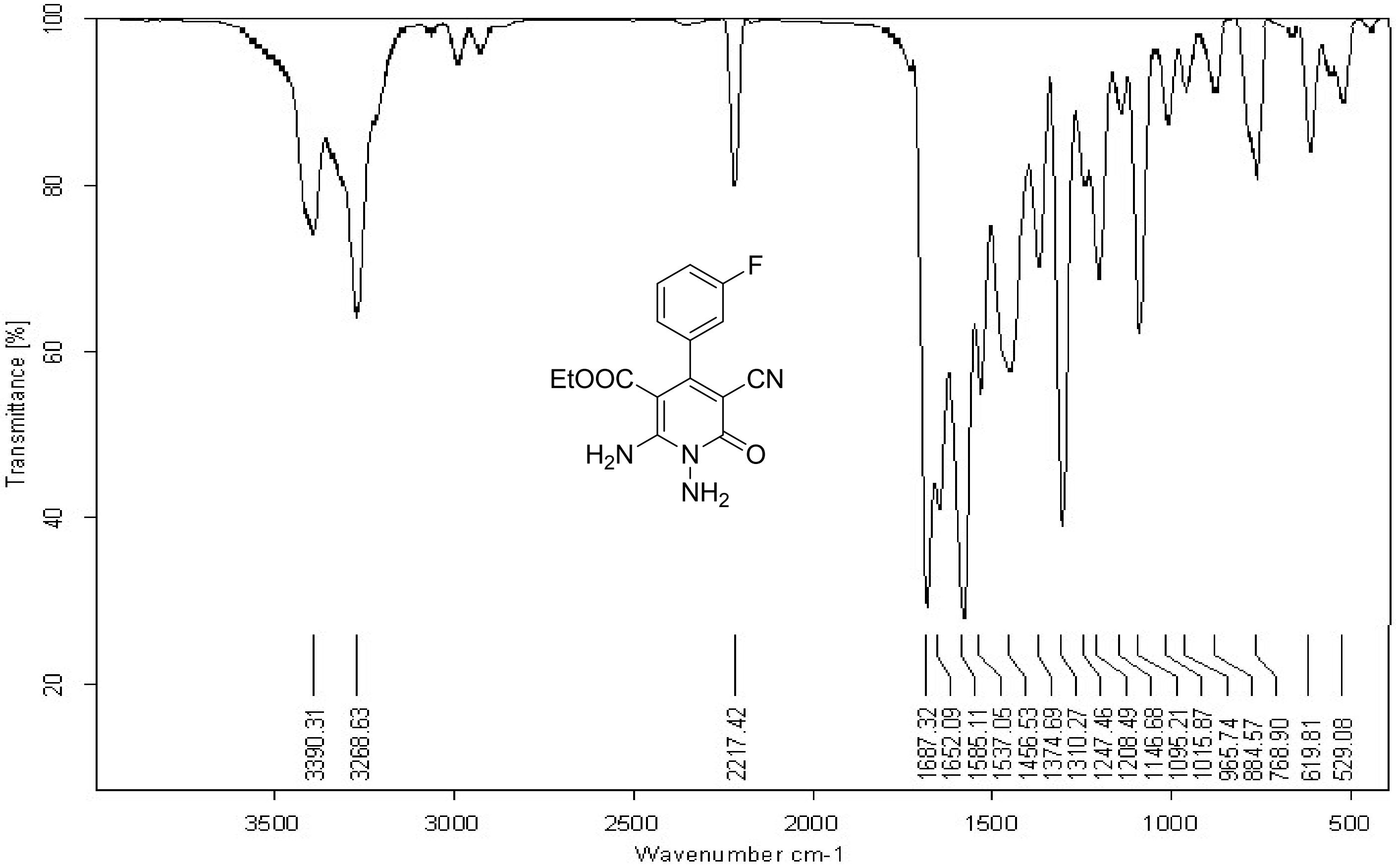

100
80
60
40
20
Transmittance [%]
3500
3000
2500
2000
1500
1000
500
Wavenumber cm-1
3390.31
3268.63
2217.42
1687.32
1652.09
1585.11
1537.05
1456.53
1374.69
1310.27
1247.46
1208.49
1146.68
1095.21
1015.87
965.74
884.57
768.90
619.81
529.08
F
EtOOC
CN
H2N
N
O
NH2

IR of 5d

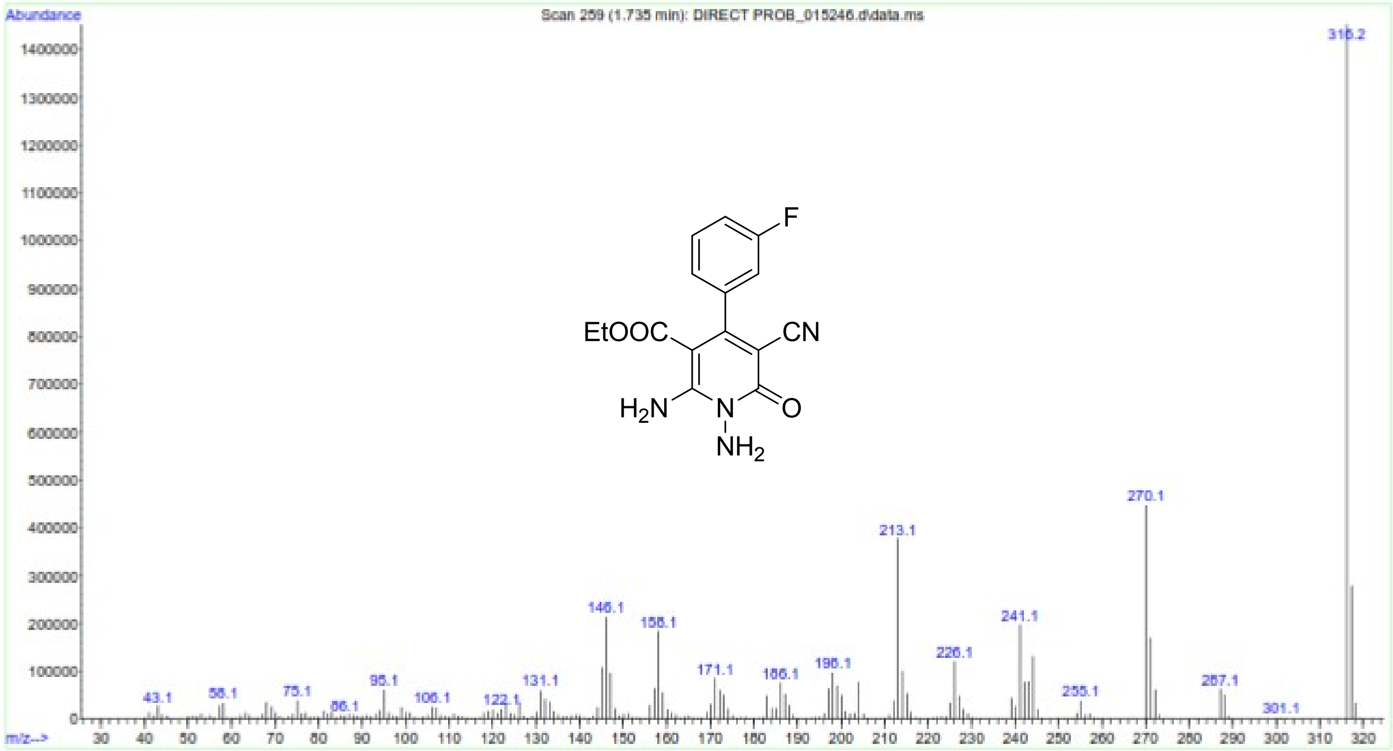
Abundance
Scan 259 (1.735 min): DIRECT PROB_015246.d\data.ms
1400000
1300000
1200000
1100000
1000000
900000
800000
700000
600000
500000
400000
300000
200000
100000
0
m/z-->
316.2
270.1
213.1
241.1
146.1
158.1
226.1
196.1
171.1
186.1
287.1
255.1
301.1
131.1
95.1
43.1
58.1
75.1
86.1
106.1
122.1
F
EtOOC
CN
H_2N
N
O
NH_2

MS of 5d

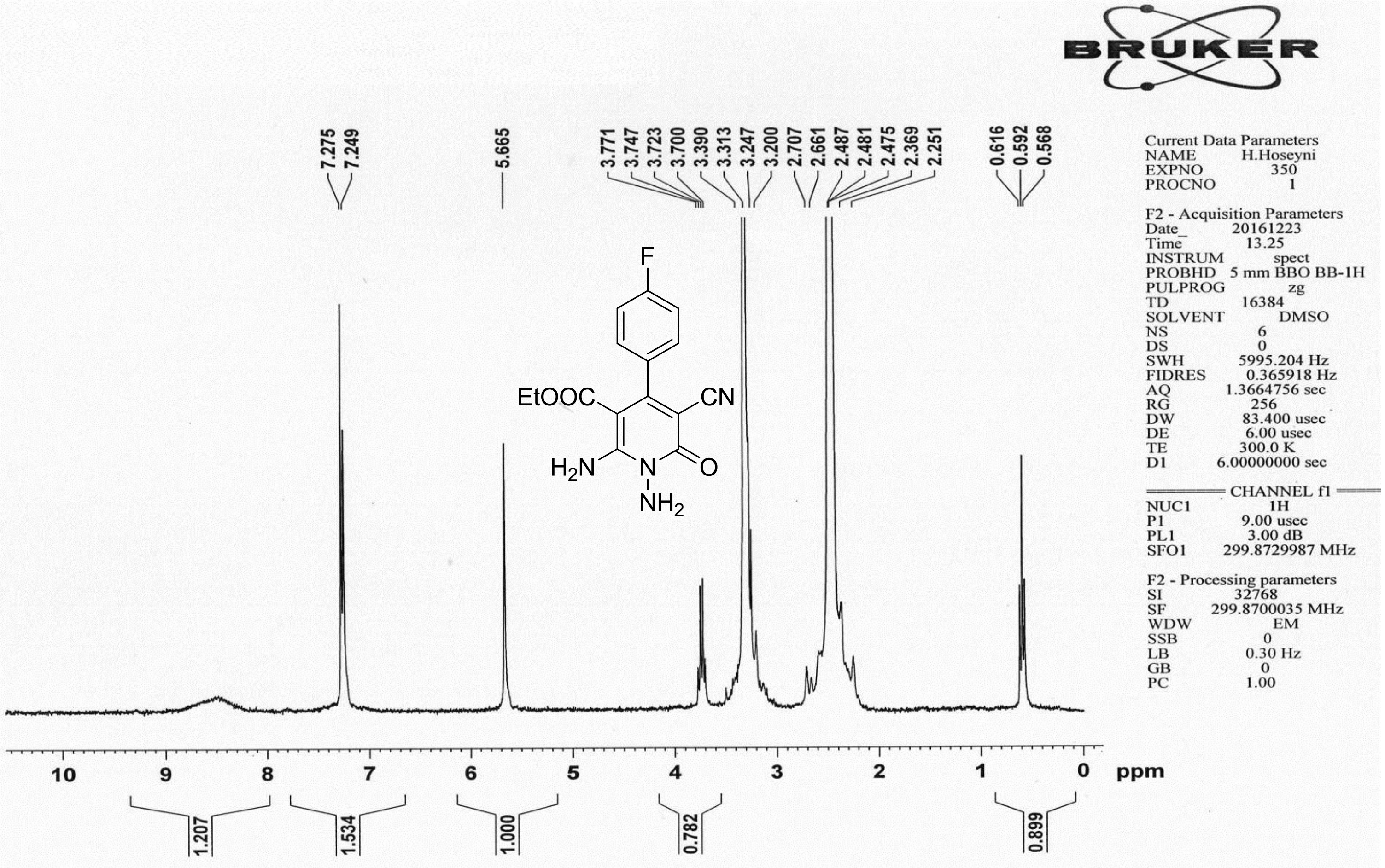

BRUKER
7.275
7.249
5.665
3.771
3.747
3.723
3.700
3.390
3.313
3.247
3.200
2.707
2.661
2.487
2.481
2.475
2.369
2.251
0.616
0.592
0.568
F
EtOOC
CN
H2N
N
O
NH2
Current Data Parameters
NAME H.Hoseyni
EXPNO 350
PROCNO 1
F2 - Acquisition Parameters
Date_ 20161223
Time 13.25
INSTRUM spect
PROBHD 5 mm BBO BB-1H
PULPROG zg
TD 16384
SOLVENT DMSO
NS 6
DS 0
SWH 5995.204 Hz
FIDRES 0.365918 Hz
AQ 1.3664756 sec
RG 256
DW 83.400 usec
DE 6.00 usec
TE 300.0 K
D1 6.00000000 sec
CHANNEL f1
NUC1 1H
P1 9.00 usec
PL1 3.00 dB
SFO1 299.8729987 MHz
F2 - Processing parameters
SI 32768
SF 299.8700035 MHz
WDW EM
SSB 0
LB 0.30 Hz
GB 0
PC 1.00
10 9 8 7 6 5 4 3 2 1 0 ppm
1.207
1.534
1.000
0.782
0.899

^{1}H NMR of 5e

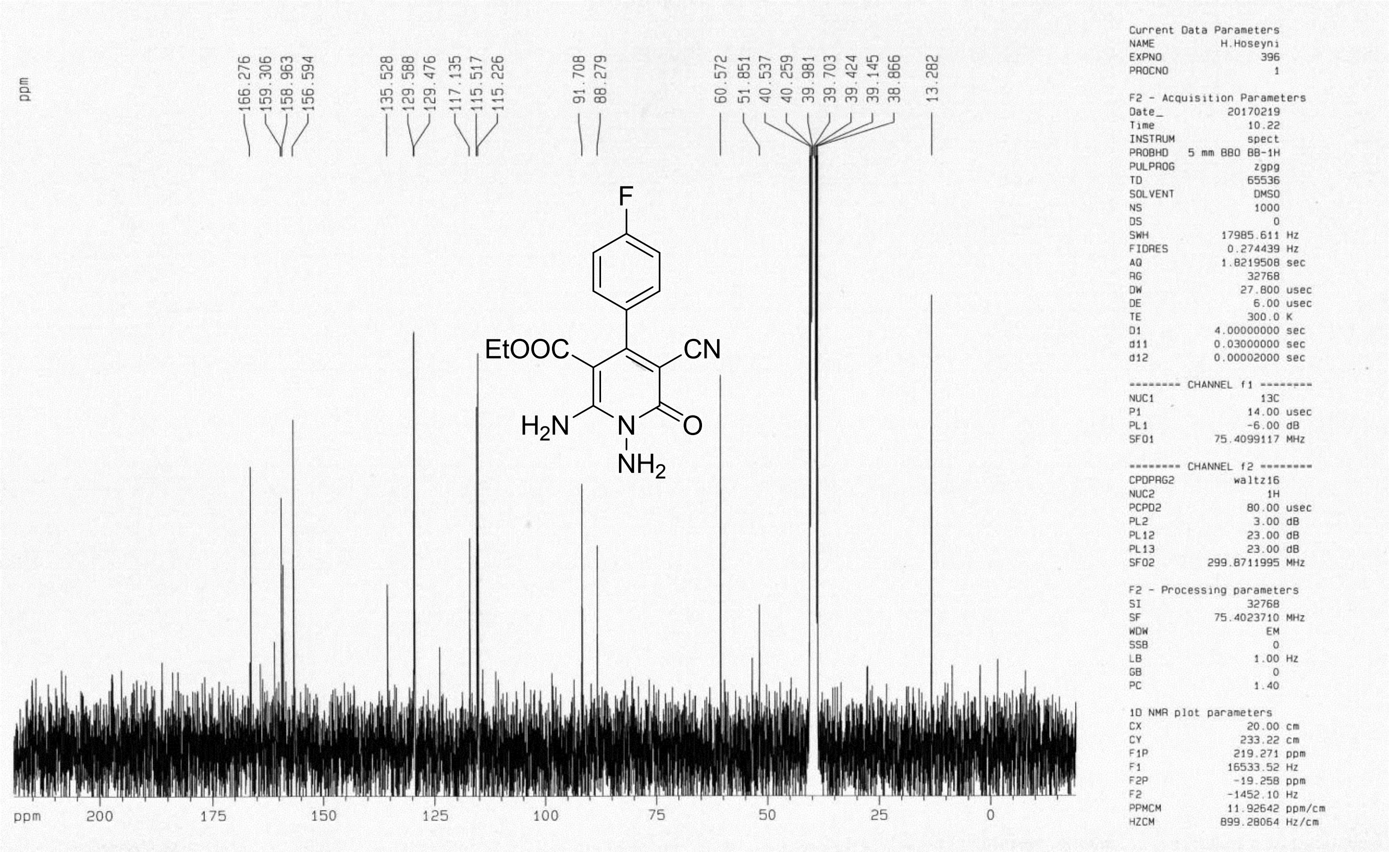

^{13}C NMR of 5e

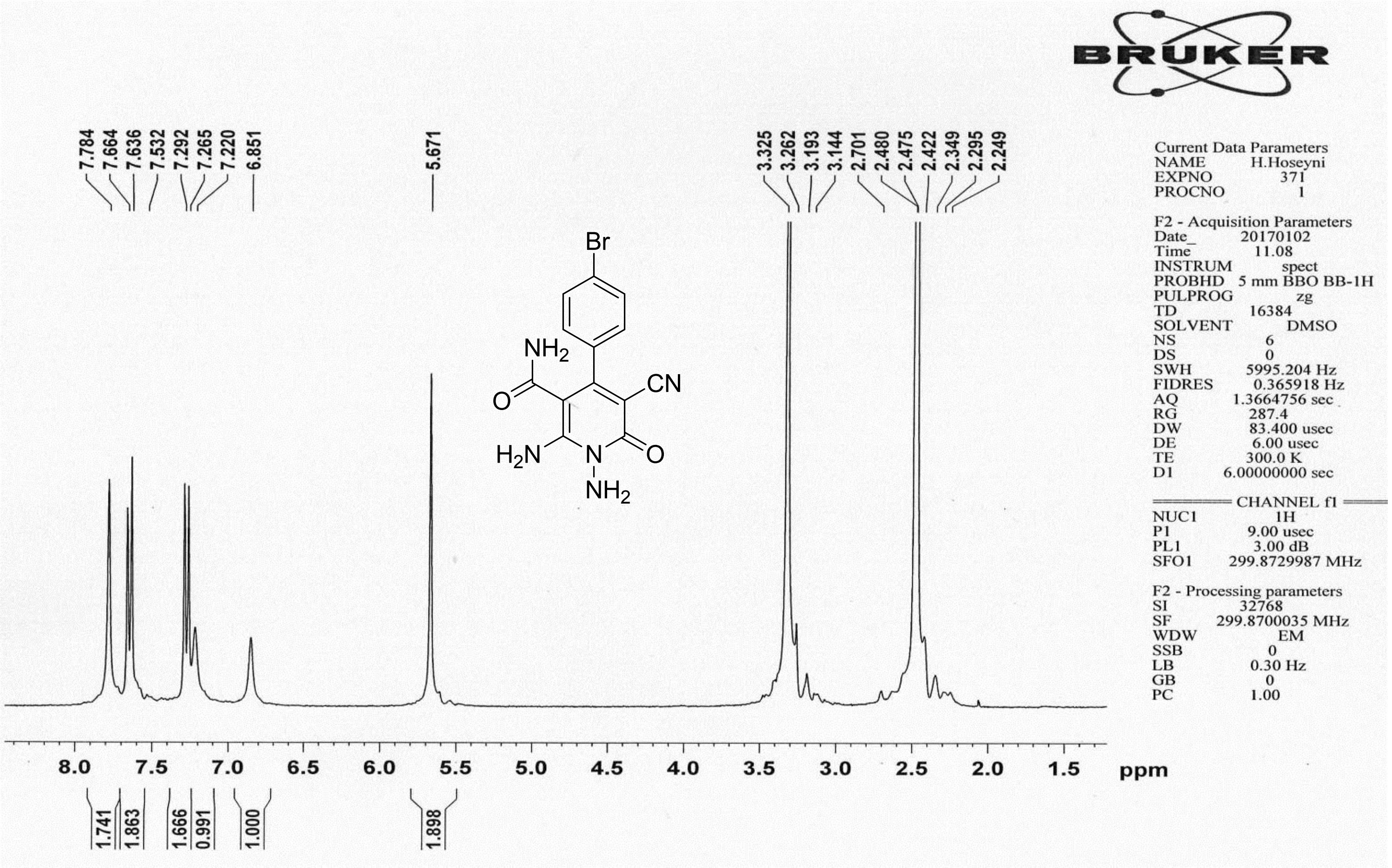
BRUKER
7.784
7.664
7.636
7.532
7.292
7.265
7.220
6.851
5.671
3.325
3.262
3.193
3.144
2.701
2.480
2.475
2.422
2.349
2.295
2.249
Br
NH2
CN
O
H2N
N
O
NH2
Current Data Parameters
NAME H.Hoseyni
EXPNO 371
PROCNO 1
F2 - Acquisition Parameters
Date_ 20170102
Time 11.08
INSTRUM spect
PROBHD 5 mm BBO BB-1H
PULPROG zg
TD 16384
SOLVENT DMSO
NS 6
DS 0
SWH 5995.204 Hz
FIDRES 0.365918 Hz
AQ 1.3664756 sec
RG 287.4
DW 83.400 usec
DE 6.00 usec
TE 300.0 K
D1 6.00000000 sec
CHANNEL f1
NUC1 1H
P1 9.00 usec
PL1 3.00 dB
SFO1 299.8729987 MHz
F2 - Processing parameters
SI 32768
SF 299.8700035 MHz
WDW EM
SSB 0
LB 0.30 Hz
GB 0
PC 1.00
8.0
7.5
7.0
6.5
6.0
5.5
5.0
4.5
4.0
3.5
3.0
2.5
2.0
1.5
ppm
1.741
1.863
1.666
0.991
1.000
1.898

^{1}H NMR of 6a

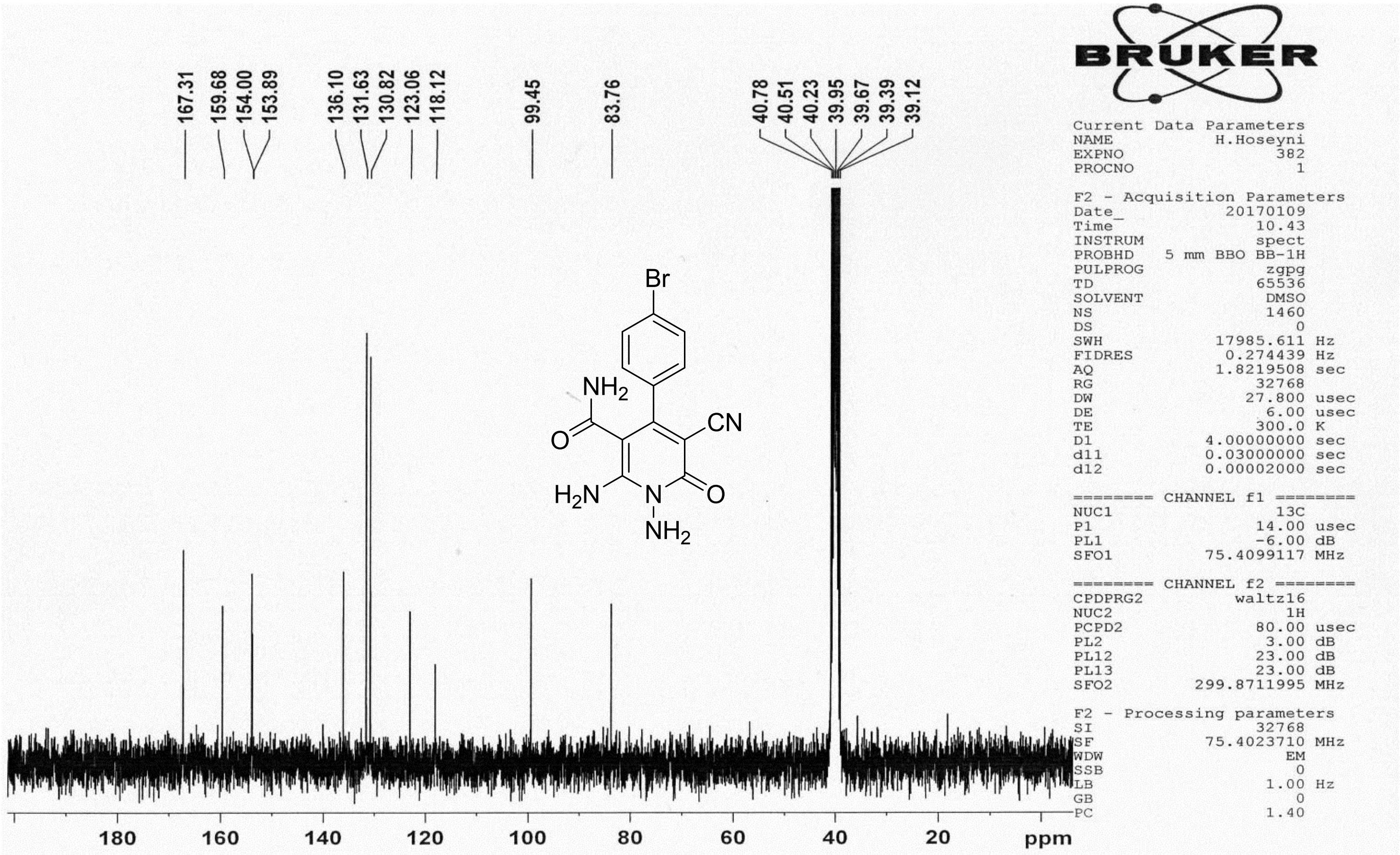

167.31
159.68
154.00
153.89
136.10
131.63
130.82
123.06
118.12
99.45
83.76
40.78
40.51
40.23
39.95
39.67
39.39
39.12
BRUKER
Current Data Parameters
NAME H.Hoseyni
EXPNO 382
PROCNO 1
F2 - Acquisition Parameters
Date_ 20170109
Time 10.43
INSTRUM spect
PROBHD 5 mm BBO BB-1H
PULPROG zgpg
TD 65536
SOLVENT DMSO
NS 1460
DS 0
SWH 17985.611 Hz
FIDRES 0.274439 Hz
AQ 1.8219508 sec
RG 32768
DW 27.800 usec
DE 6.00 usec
TE 300.0 K
D1 4.00000000 sec
d11 0.03000000 sec
d12 0.00002000 sec
======== CHANNEL f1 ========
NUC1 13C
P1 14.00 usec
PL1 -6.00 dB
SFO1 75.4099117 MHz
======== CHANNEL f2 ========
CPDPRG2 waltz16
NUC2 1H
PCPD2 80.00 usec
PL2 3.00 dB
PL12 23.00 dB
PL13 23.00 dB
SFO2 299.8711995 MHz
F2 - Processing parameters
SI 32768
SF 75.4023710 MHz
WDW EM
SSB 0
LB 1.00 Hz
GB 0
PC 1.40
Br
NH2
CN
O
H2N
N
O
NH2
180
160
140
120
100
80
60
40
20
ppm

^{13}C NMR of 6a

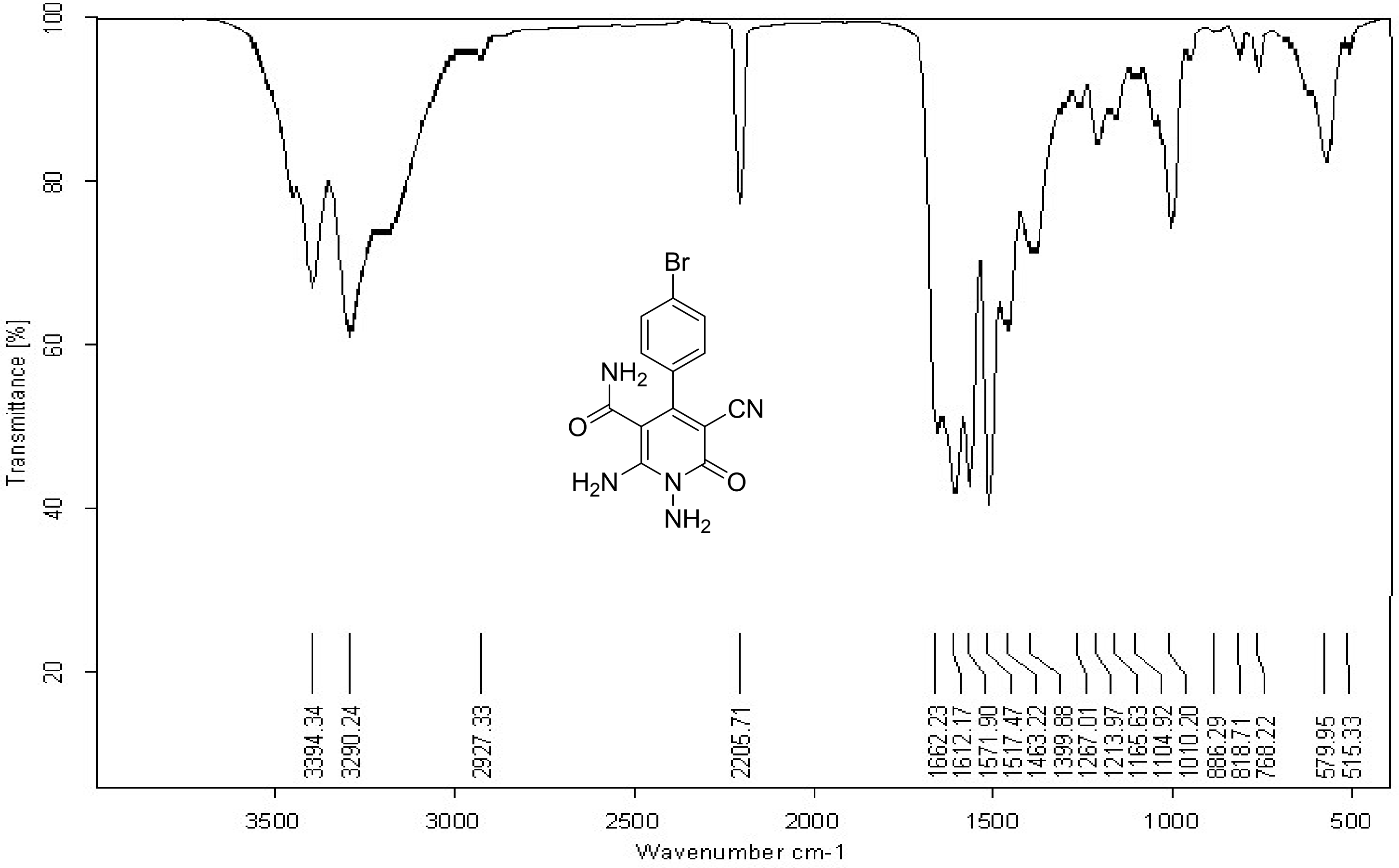
Transmittance [%]
100
80
60
40
20
Wavenumber cm-1
3500
3000
2500
2000
1500
1000
500
3394.34
3290.24
2927.33
2205.71
1662.23
1612.17
1571.90
1517.47
1463.22
1399.88
1267.01
1213.97
1165.63
1104.92
1010.20
886.29
818.71
768.22
579.95
515.33
Br
NH2
CN
O
H2N
N
O
NH2

IR of 6a

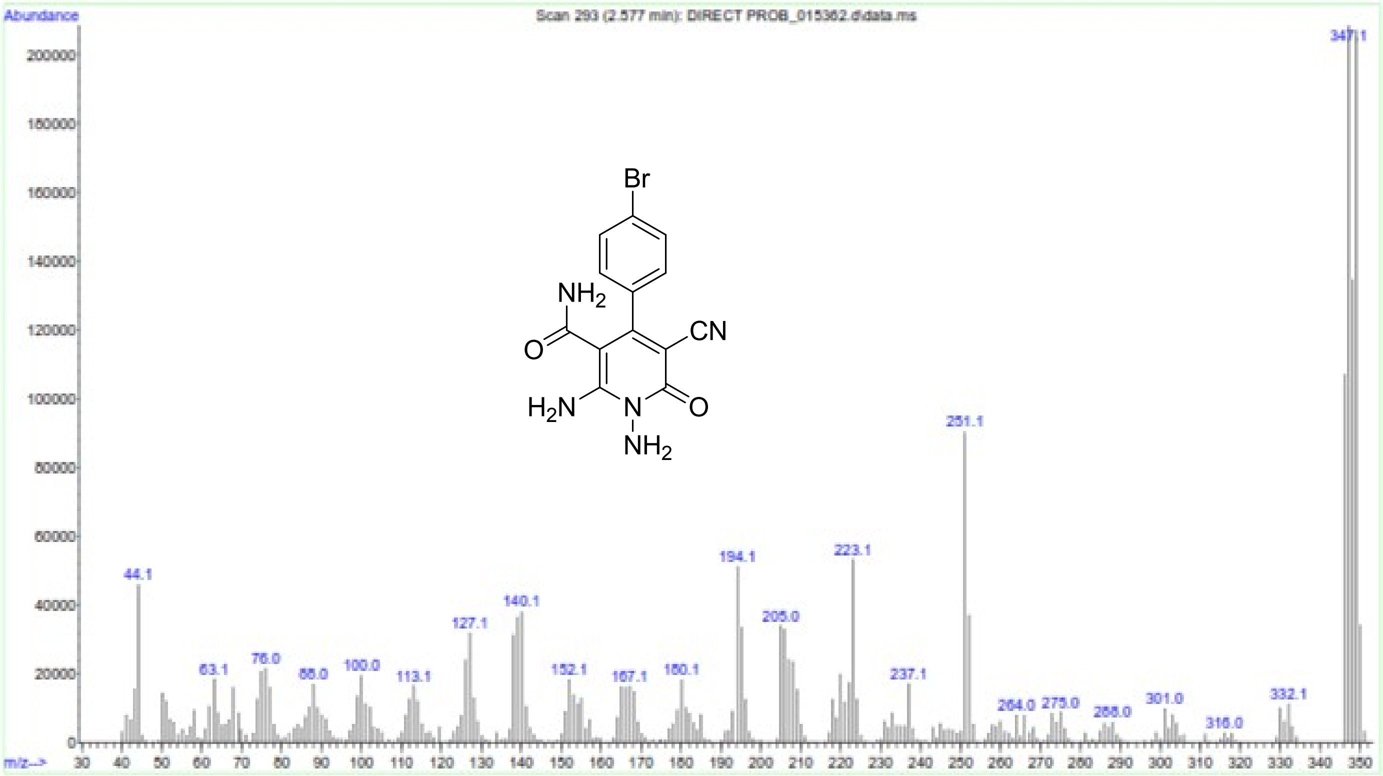

Abundance
Scan 293 (2.577 min): DIRECT PROB_015362.d\data.ms
m/z-->
Br
NH2
CN
O
H2N
N
O
NH2
347.1
251.1
223.1
194.1
205.0
140.1
127.1
44.1
237.1

MS of 6a

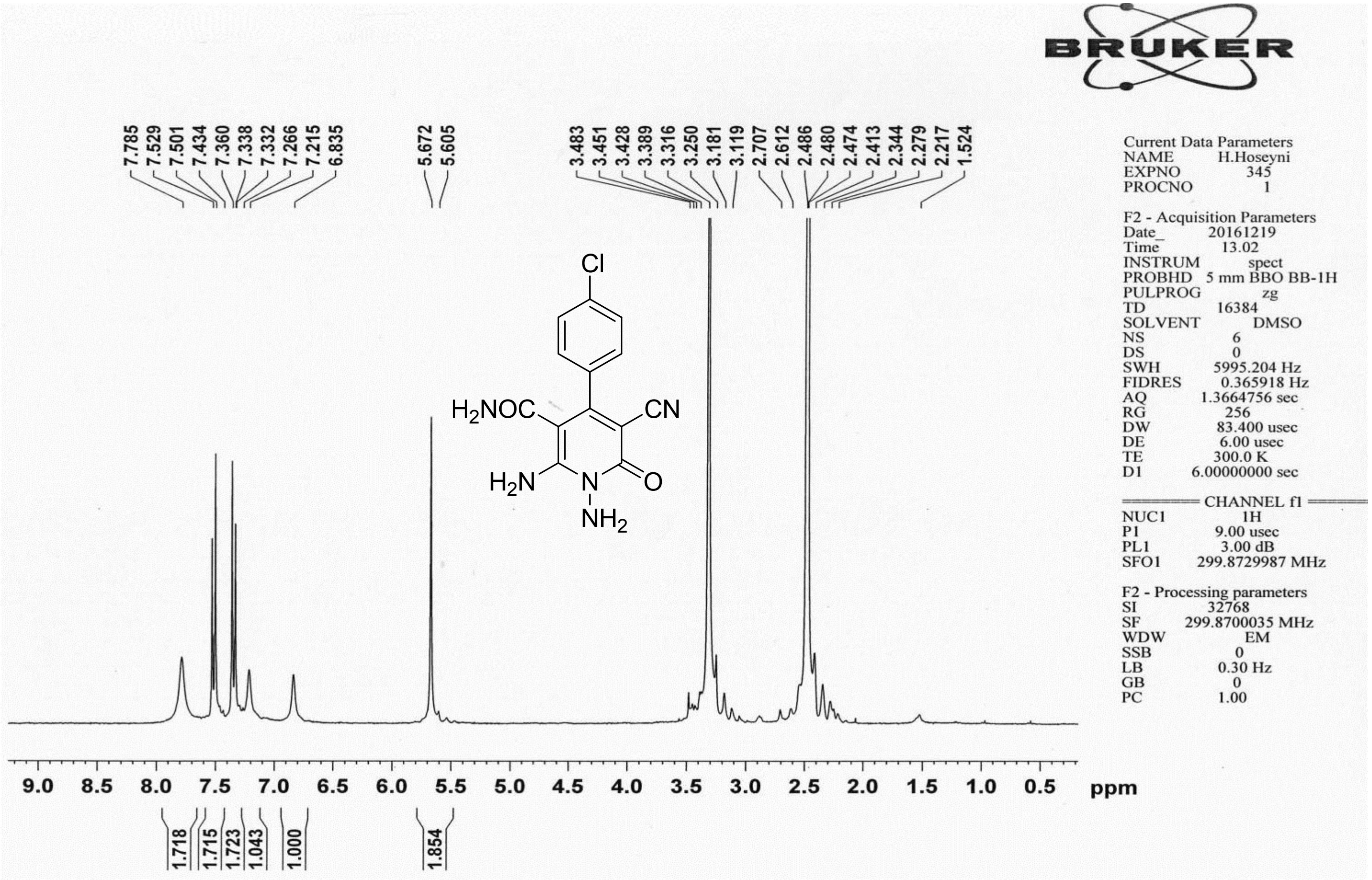
BRUKER
7.785
7.529
7.501
7.434
7.360
7.338
7.332
7.266
7.215
6.835
5.672
5.605
3.483
3.451
3.428
3.389
3.316
3.250
3.181
3.119
2.707
2.612
2.486
2.480
2.474
2.413
2.344
2.279
2.217
1.524
Cl
H2NOC
CN
H2N
N
O
NH2
Current Data Parameters
NAME H.Hoseyni
EXPNO 345
PROCNO 1
F2 - Acquisition Parameters
Date_ 20161219
Time_ 13.02
INSTRUM spect
PROBHD 5 mm BBO BB-1H
PULPROG zg
TD 16384
SOLVENT DMSO
NS 6
DS 0
SWH 5995.204 Hz
FIDRES 0.365918 Hz
AQ 1.3664756 sec
RG 256
DW 83.400 usec
DE 6.00 usec
TE 300.0 K
D1 6.00000000 sec
CHANNEL f1
NUC1 1H
P1 9.00 usec
PL1 3.00 dB
SFO1 299.8729987 MHz
F2 - Processing parameters
SI 32768
SF 299.8700035 MHz
WDW EM
SSB 0
LB 0.30 Hz
GB 0
PC 1.00
9.0 8.5 8.0 7.5 7.0 6.5 6.0 5.5 5.0 4.5 4.0 3.5 3.0 2.5 2.0 1.5 1.0 0.5 ppm
1.718
1.715
1.723
1.043
1.000
1.854

^{1}H NMR of 6b

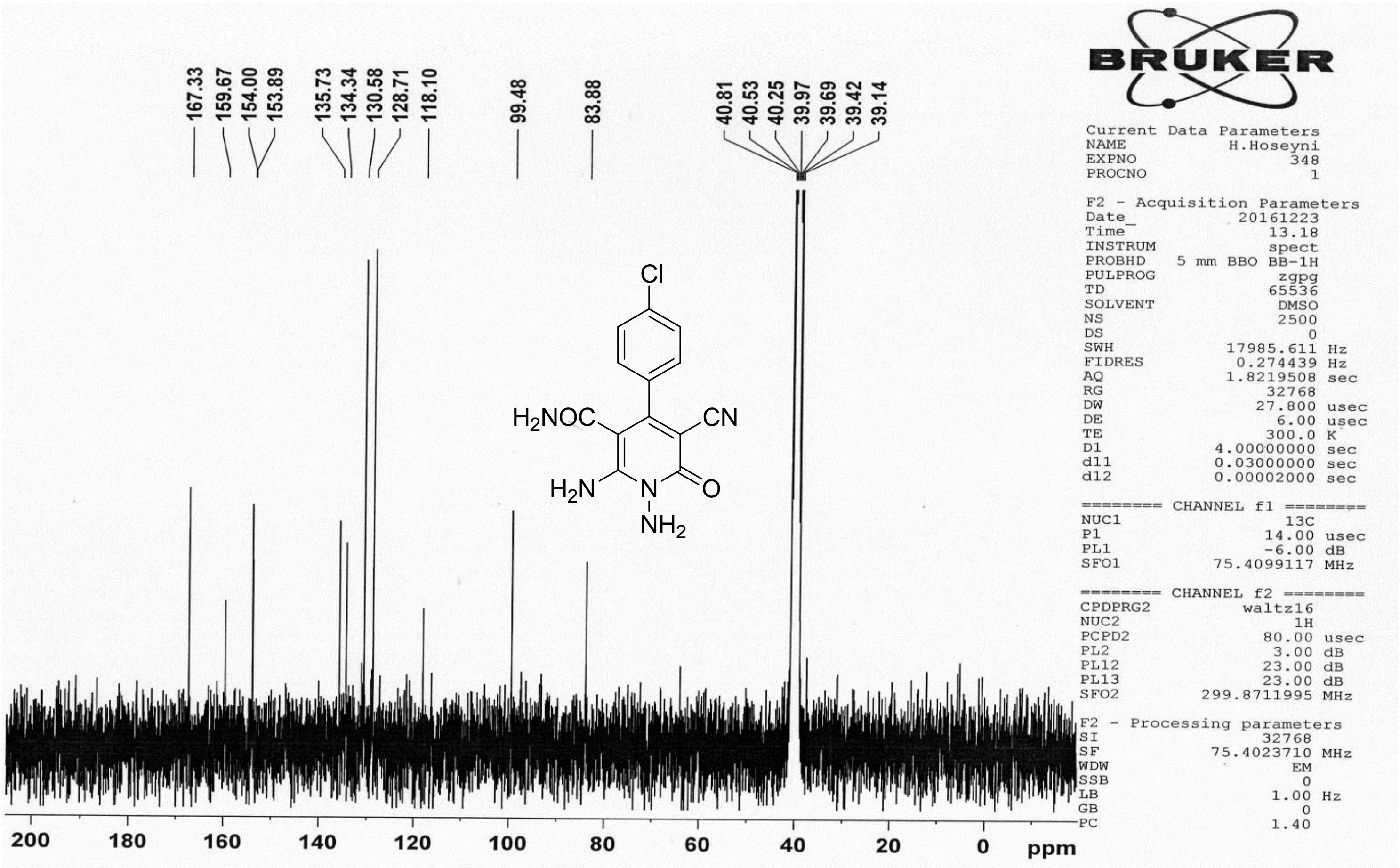
167.33
159.67
154.00
153.89
135.73
134.34
130.58
128.71
118.10
99.48
83.88
40.81
40.53
40.25
39.97
39.69
39.42
39.14
BRUKER
Current Data Parameters
NAME H.Hoseyni
EXPNO 348
PROCNO 1
F2 - Acquisition Parameters
Date_ 20161223
Time 13.18
INSTRUM spect
PROBHD 5 mm BBO BB-1H
PULPROG zgpg
TD 65536
SOLVENT DMSO
NS 2500
DS 0
SWH 17985.611 Hz
FIDRES 0.274439 Hz
AQ 1.8219508 sec
RG 32768
DW 27.800 usec
DE 6.00 usec
TE 300.0 K
D1 4.00000000 sec
d11 0.03000000 sec
d12 0.00002000 sec
======== CHANNEL f1 ========
NUC1 13C
P1 14.00 usec
PL1 -6.00 dB
SFO1 75.4099117 MHz
======== CHANNEL f2 ========
CPDPRG2 waltz16
NUC2 1H
PCPD2 80.00 usec
PL2 3.00 dB
PL12 23.00 dB
PL13 23.00 dB
SFO2 299.8711995 MHz
F2 - Processing parameters
SI 32768
SF 75.4023710 MHz
WDW EM
SSB 0
LB 1.00 Hz
GB 0
PC 1.40
Cl
H2NOC
CN
H2N
N
O
NH2
200 180 160 140 120 100 80 60 40 20 0 ppm

^{13}C NMR of 6b

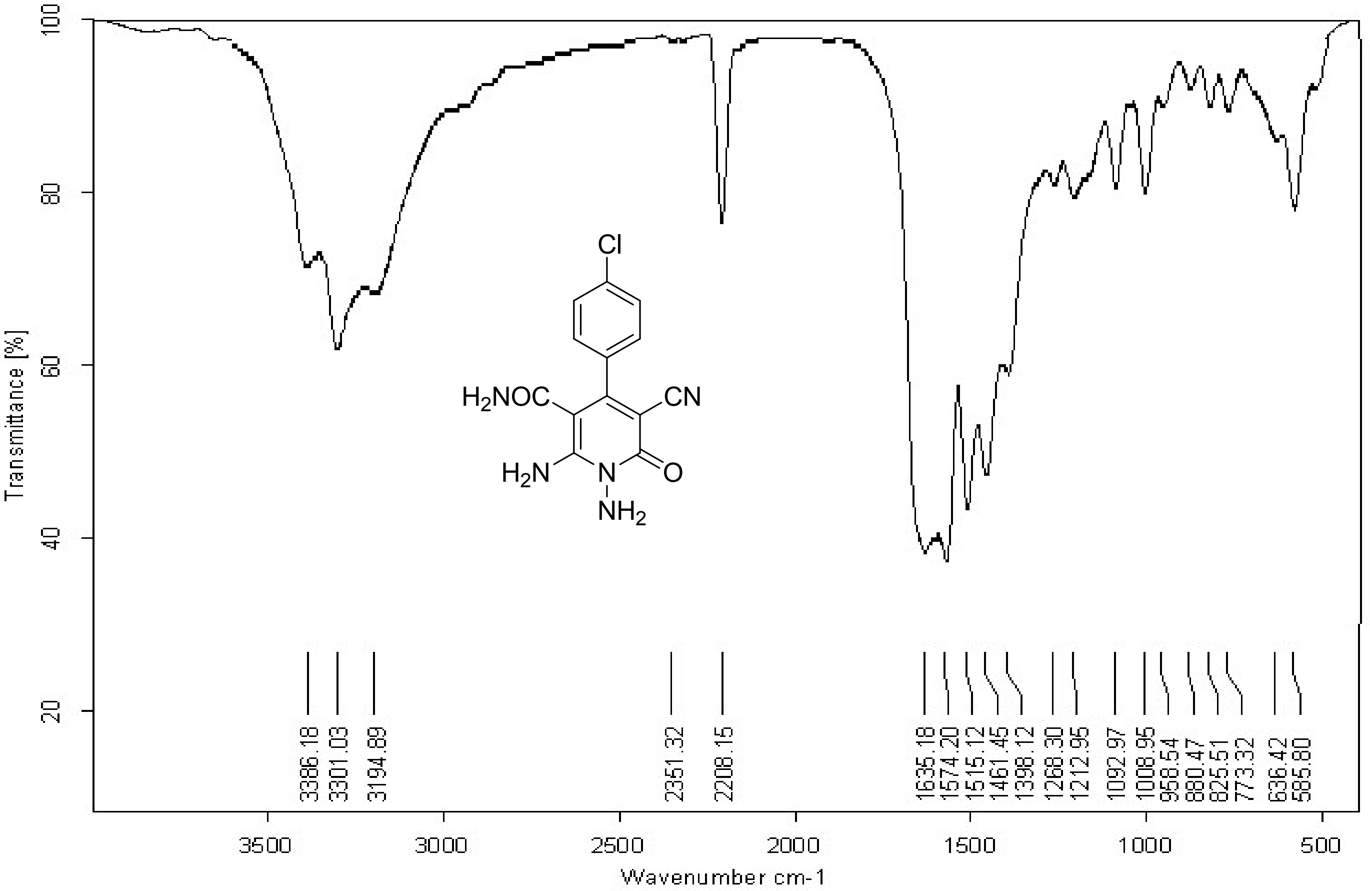
Transmittance [%]
Wavenumber cm-1
Cl
H_2NOC
CN
H_2N
N
O
NH_2
3386.18
3301.03
3194.89
2351.32
2208.15
1635.18
1574.20
1515.12
1461.45
1398.12
1268.30
1212.95
1092.97
1008.95
958.54
880.47
825.51
773.32
636.42
585.80

IR of 6b

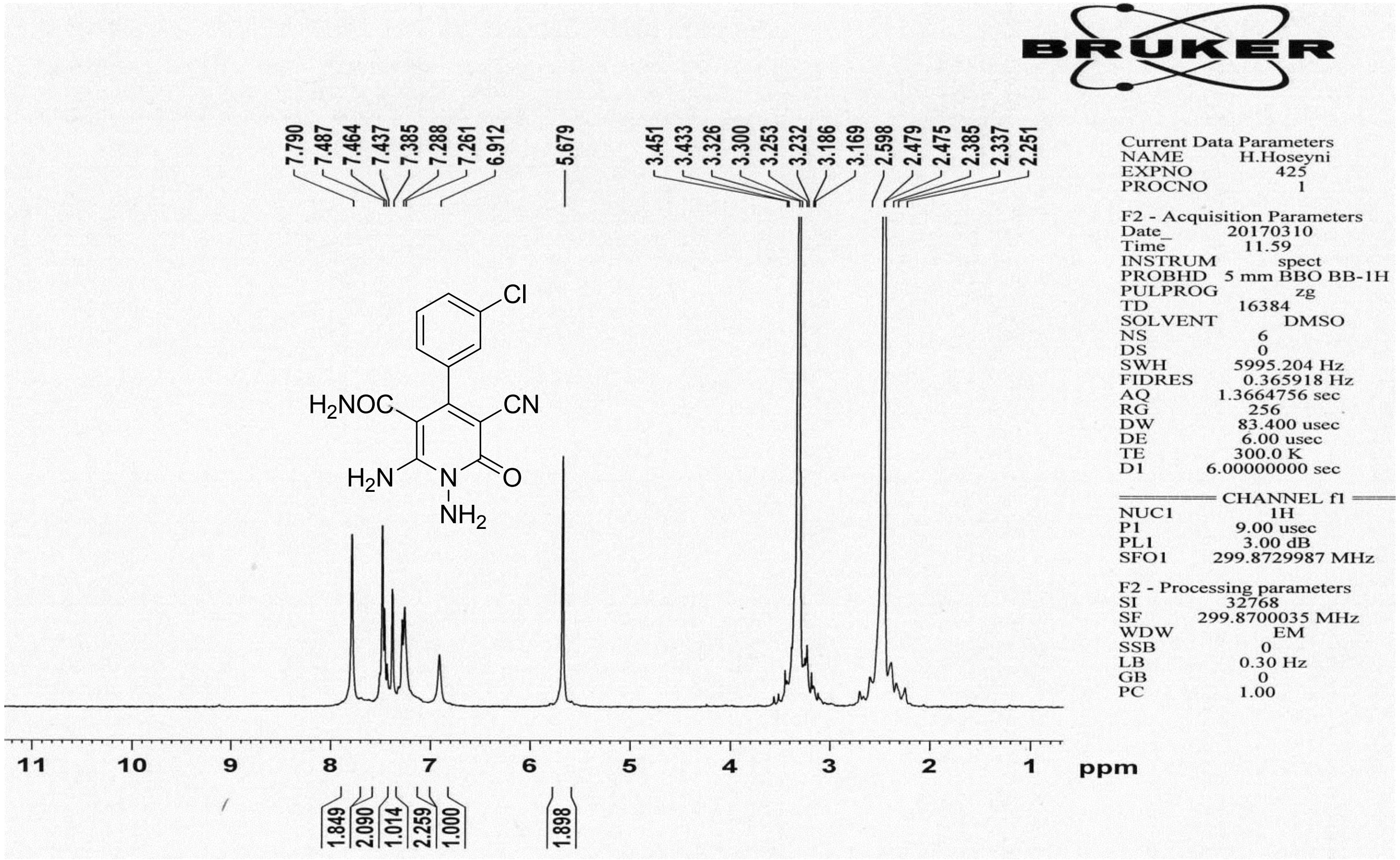
BRUKER
7.790
7.487
7.464
7.437
7.385
7.288
7.261
6.912
5.679
3.451
3.433
3.326
3.300
3.253
3.232
3.186
3.169
2.598
2.479
2.475
2.385
2.337
2.251
Current Data Parameters
NAME H.Hoseyni
EXPNO 425
PROCNO 1
F2 - Acquisition Parameters
Date_ 20170310
Time 11.59
INSTRUM spect
PROBHD 5 mm BBO BB-1H
PULPROG zg
TD 16384
SOLVENT DMSO
NS 6
DS 0
SWH 5995.204 Hz
FIDRES 0.365918 Hz
AQ 1.3664756 sec
RG 256
DW 83.400 usec
DE 6.00 usec
TE 300.0 K
D1 6.00000000 sec
CHANNEL f1
NUC1 1H
P1 9.00 usec
PL1 3.00 dB
SFO1 299.8729987 MHz
F2 - Processing parameters
SI 32768
SF 299.8700035 MHz
WDW EM
SSB 0
LB 0.30 Hz
GB 0
PC 1.00
Cl
H2NOC
CN
H2N
N
O
NH2
11 10 9 8 7 6 5 4 3 2 1 ppm
1.849
2.090
1.014
2.259
1.000
1.898

^{1}H NMR of 6c

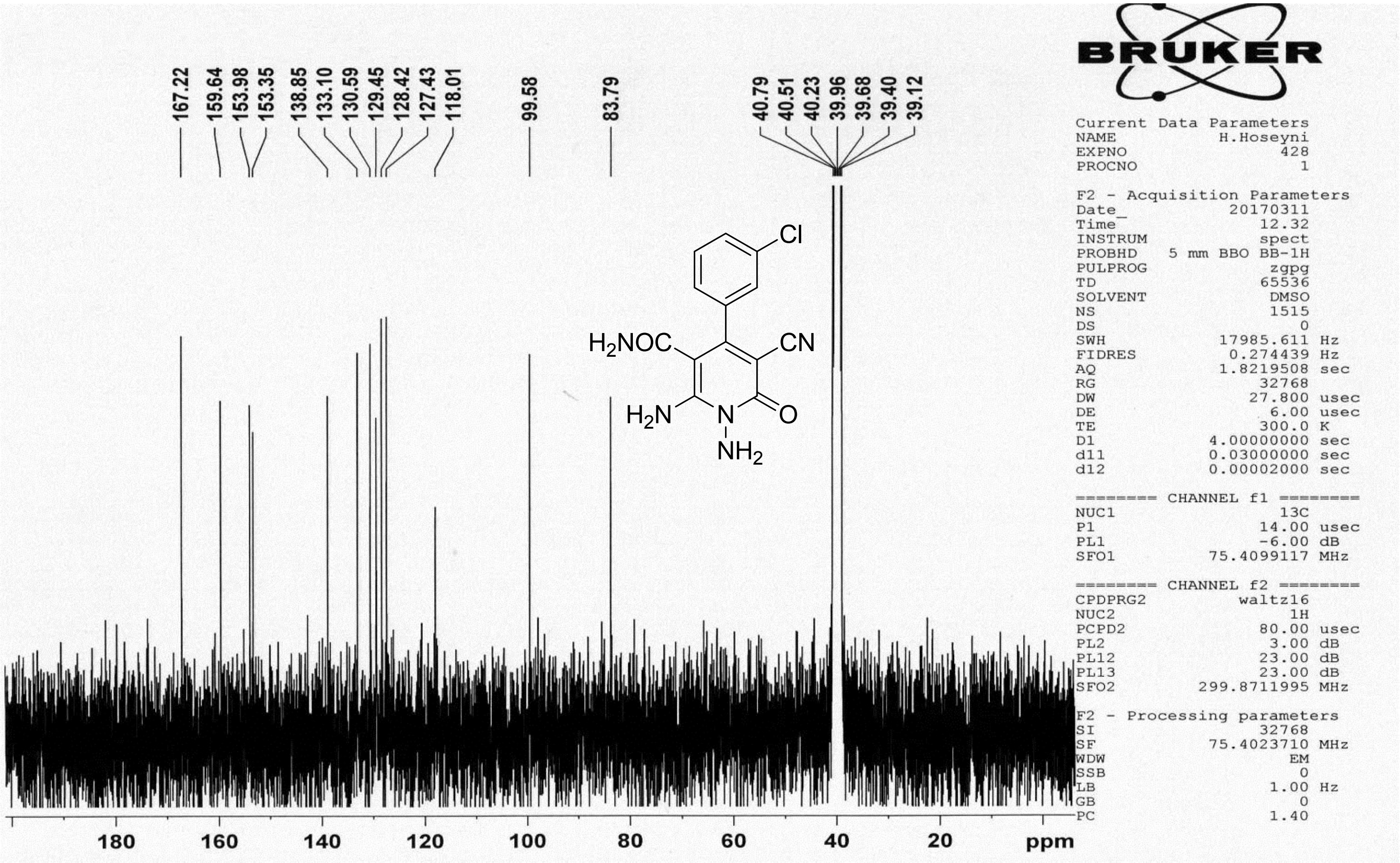
167.22
159.64
153.98
153.35
138.85
133.10
130.59
129.45
128.42
127.43
118.01
99.58
83.79
40.79
40.51
40.23
39.96
39.68
39.40
39.12
BRUKER
Current Data Parameters
NAME H.Hoseyni
EXPNO 428
PROCNO 1
F2 - Acquisition Parameters
Date_ 20170311
Time 12.32
INSTRUM spect
PROBHD 5 mm BBO BB-1H
PULPROG zgpg
TD 65536
SOLVENT DMSO
NS 1515
DS 0
SWH 17985.611 Hz
FIDRES 0.274439 Hz
AQ 1.8219508 sec
RG 32768
DW 27.800 usec
DE 6.00 usec
TE 300.0 K
D1 4.00000000 sec
d11 0.03000000 sec
d12 0.00002000 sec
======== CHANNEL f1 ========
NUC1 13C
P1 14.00 usec
PL1 -6.00 dB
SFO1 75.4099117 MHz
======== CHANNEL f2 ========
CPDPRG2 waltz16
NUC2 1H
PCPD2 80.00 usec
PL2 3.00 dB
PL12 23.00 dB
PL13 23.00 dB
SFO2 299.8711995 MHz
F2 - Processing parameters
SI 32768
SF 75.4023710 MHz
WDW EM
SSB 0
LB 1.00 Hz
GB 0
PC 1.40
Cl
H2NOC
CN
H2N
N
O
NH2
180
160
140
120
100
80
60
40
20
ppm

^{13}C NMR of 6c

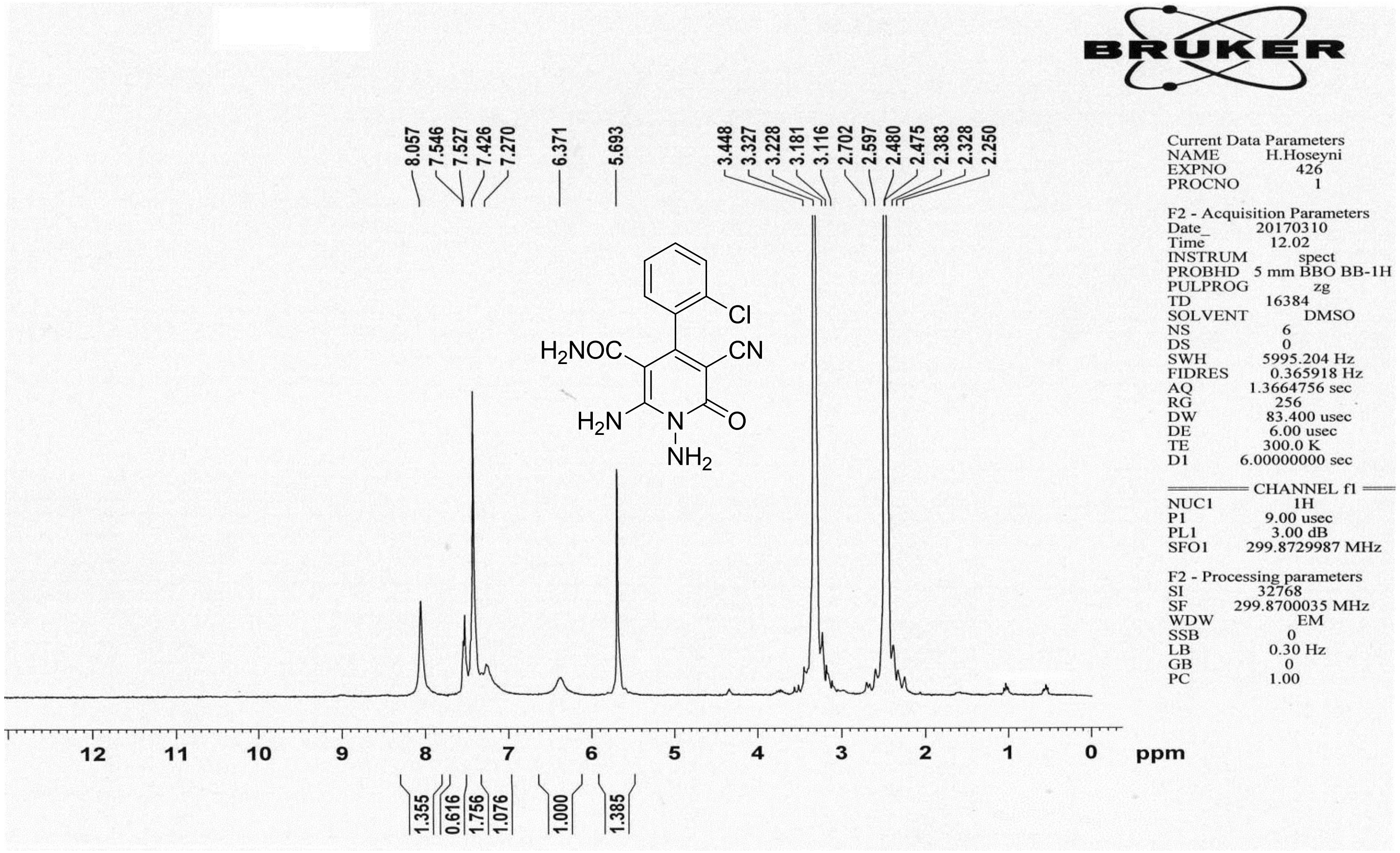
BRUKER
8.057
7.546
7.527
7.426
7.270
6.371
5.693
3.448
3.327
3.228
3.181
3.116
2.702
2.597
2.480
2.475
2.383
2.328
2.250
Current Data Parameters
NAME H.Hoseyni
EXPNO 426
PROCNO 1
F2 - Acquisition Parameters
Date_ 20170310
Time 12.02
INSTRUM spect
PROBHD 5 mm BBO BB-1H
PULPROG zg
TD 16384
SOLVENT DMSO
NS 6
DS 0
SWH 5995.204 Hz
FIDRES 0.365918 Hz
AQ 1.3664756 sec
RG 256
DW 83.400 usec
DE 6.00 usec
TE 300.0 K
D1 6.00000000 sec
CHANNEL f1
NUC1 1H
P1 9.00 usec
PL1 3.00 dB
SFO1 299.8729987 MHz
F2 - Processing parameters
SI 32768
SF 299.8700035 MHz
WDW EM
SSB 0
LB 0.30 Hz
GB 0
PC 1.00
Cl
H2NOC
CN
H2N
N
O
NH2
12
11
10
9
8
7
6
5
4
3
2
1
0
ppm
1.355
0.616
1.756
1.076
1.000
1.385

^{1}H NMR of 6d

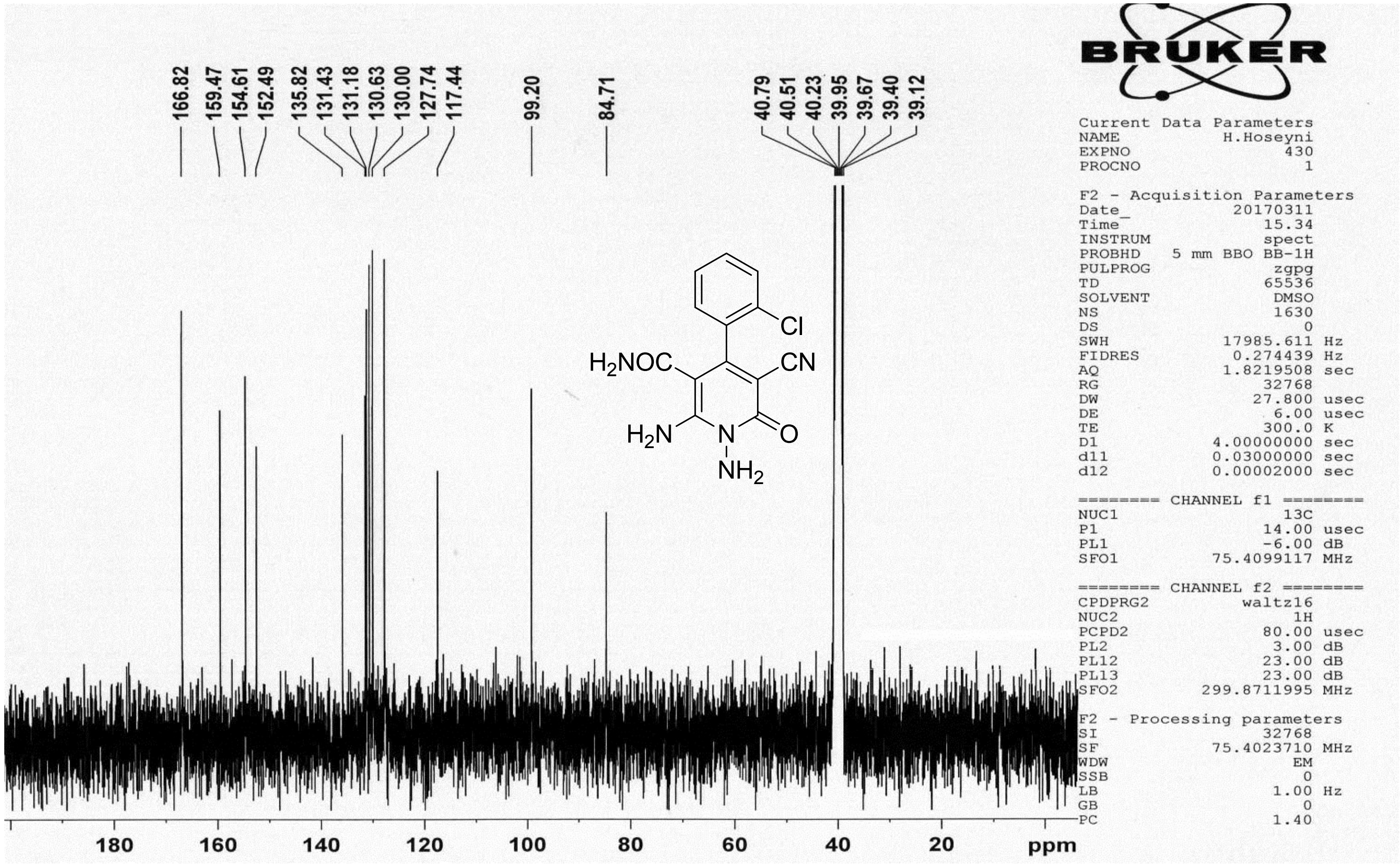
166.82
159.47
154.61
152.49
135.82
131.43
131.18
130.63
130.00
127.74
117.44
99.20
84.71
40.79
40.51
40.23
39.95
39.67
39.40
39.12
BRUKER
Current Data Parameters
NAME H.Hoseyni
EXPNO 430
PROCNO 1
F2 - Acquisition Parameters
Date_ 20170311
Time 15.34
INSTRUM spect
PROBHD 5 mm BBO BB-1H
PULPROG zgpg
TD 65536
SOLVENT DMSO
NS 1630
DS 0
SWH 17985.611 Hz
FIDRES 0.274439 Hz
AQ 1.8219508 sec
RG 32768
DW 27.800 usec
DE 6.00 usec
TE 300.0 K
D1 4.00000000 sec
d11 0.03000000 sec
d12 0.00002000 sec
======== CHANNEL f1 ========
NUC1 13C
P1 14.00 usec
PL1 -6.00 dB
SFO1 75.4099117 MHz
======== CHANNEL f2 ========
CPDPRG2 waltz16
NUC2 1H
PCPD2 80.00 usec
PL2 3.00 dB
PL12 23.00 dB
PL13 23.00 dB
SFO2 299.8711995 MHz
F2 - Processing parameters
SI 32768
SF 75.4023710 MHz
WDW EM
SSB 0
LB 1.00 Hz
GB 0
PC 1.40
Cl
H2NOC
CN
H2N
N
O
NH2
180
160
140
120
100
80
60
40
20
ppm

^{13}C NMR of 6d

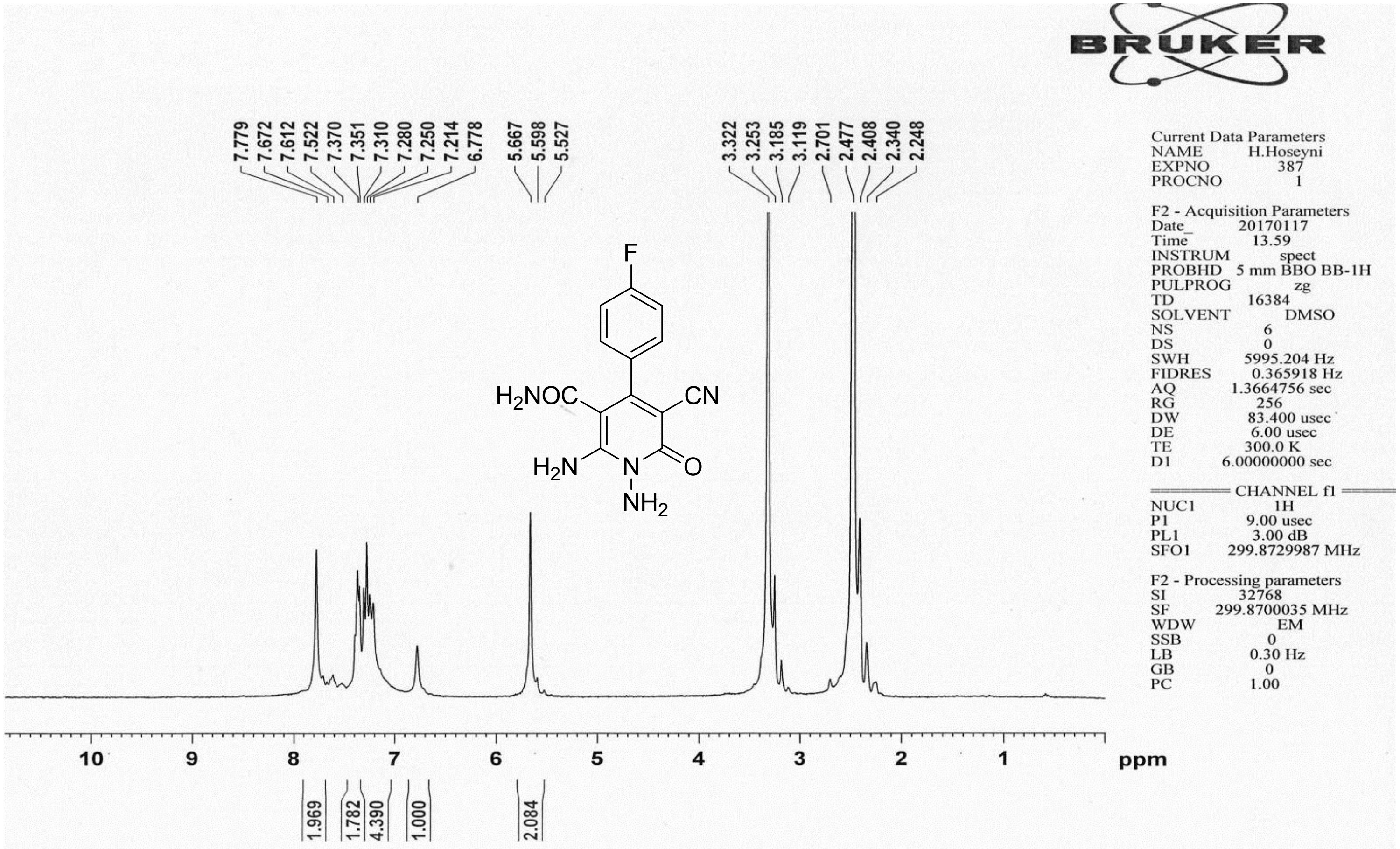

BRUKER
7.779
7.672
7.612
7.522
7.370
7.351
7.310
7.280
7.250
7.214
6.778
5.667
5.598
5.527
3.322
3.253
3.185
3.119
2.701
2.477
2.408
2.340
2.248
F
H2NOC
CN
H2N
N
O
NH2
Current Data Parameters
NAME H.Hoseyni
EXPNO 387
PROCNO 1
F2 - Acquisition Parameters
Date_ 20170117
Time 13.59
INSTRUM spect
PROBHD 5 mm BBO BB-1H
PULPROG zg
TD 16384
SOLVENT DMSO
NS 6
DS 0
SWH 5995.204 Hz
FIDRES 0.365918 Hz
AQ 1.3664756 sec
RG 256
DW 83.400 usec
DE 6.00 usec
TE 300.0 K
D1 6.00000000 sec
CHANNEL f1
NUC1 1H
P1 9.00 usec
PL1 3.00 dB
SFO1 299.8729987 MHz
F2 - Processing parameters
SI 32768
SF 299.8700035 MHz
WDW EM
SSB 0
LB 0.30 Hz
GB 0
PC 1.00
10
9
8
7
6
5
4
3
2
1
ppm
1.969
1.782
4.390
1.000
2.084

^{1}H NMR of 6e

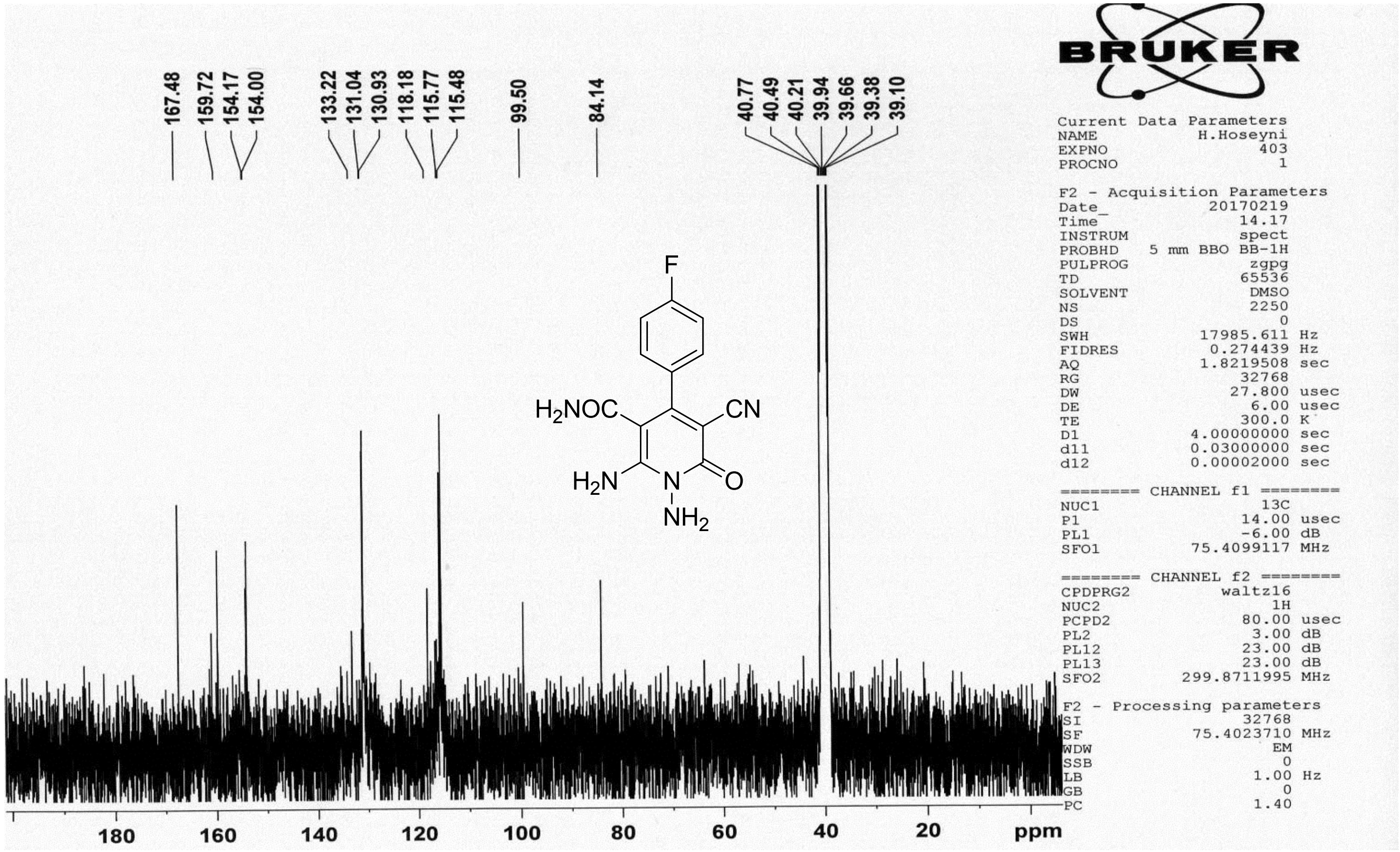
167.48
159.72
154.17
154.00
133.22
131.04
130.93
118.18
115.77
115.48
99.50
84.14
40.77
40.49
40.21
39.94
39.66
39.38
39.10
BRUKER
Current Data Parameters
NAME H.Hoseyni
EXPNO 403
PROCNO 1
F2 - Acquisition Parameters
Date_ 20170219
Time 14.17
INSTRUM spect
PROBHD 5 mm BBO BB-1H
PULPROG zgpg
TD 65536
SOLVENT DMSO
NS 2250
DS 0
SWH 17985.611 Hz
FIDRES 0.274439 Hz
AQ 1.8219508 sec
RG 32768
DW 27.800 usec
DE 6.00 usec
TE 300.0 K
D1 4.00000000 sec
d11 0.03000000 sec
d12 0.00002000 sec
======== CHANNEL f1 ========
NUC1 13C
P1 14.00 usec
PL1 -6.00 dB
SFO1 75.4099117 MHz
======== CHANNEL f2 ========
CPDPRG2 waltz16
NUC2 1H
PCPD2 80.00 usec
PL2 3.00 dB
PL12 23.00 dB
PL13 23.00 dB
SFO2 299.8711995 MHz
F2 - Processing parameters
SI 32768
SF 75.4023710 MHz
WDW EM
SSB 0
LB 1.00 Hz
GB 0
PC 1.40
F
H2NOC
CN
H2N
N
O
NH2
180
160
140
120
100
80
60
40
20
ppm

^{13}C NMR of 6e

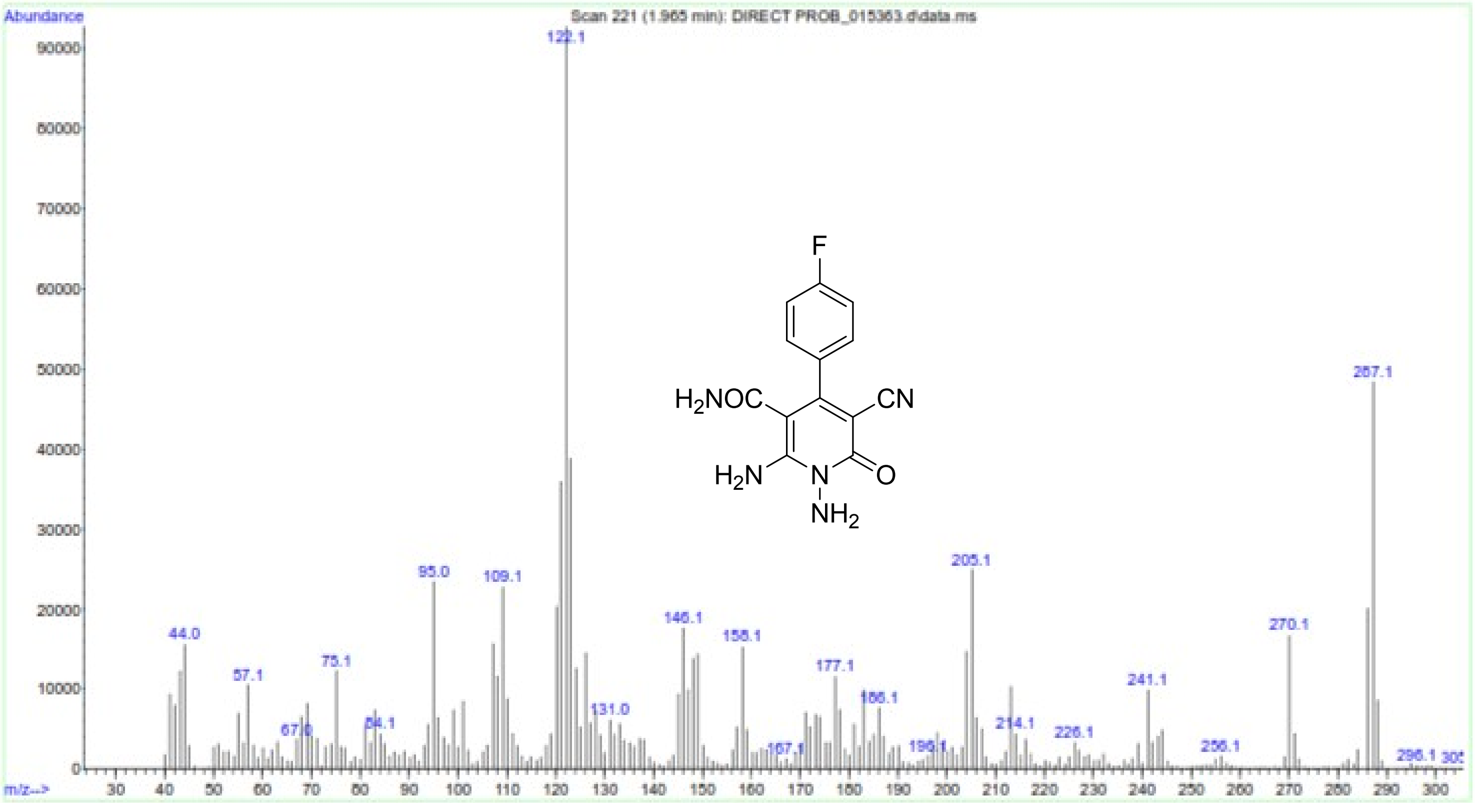
Abundance
Scan 221 (1.965 min): DIRECT PROB_015363.d\data.ms
122.1
287.1
205.1
95.0
109.1
146.1
158.1
270.1
44.0
57.1
75.1
177.1
241.1
186.1
131.0
214.1
226.1
256.1
296.1
m/z-->
F
H2NOC
CN
H2N
N
O
NH2

MS of 6e
